# Supplementary material for: C(sp3)–H sulfinylation of light hydrocarbons with sulfur dioxide via hydrogen atom transfer photocatalysis in flow
Source: Nat Commun. 2024 Jun 19;15:5246. doi: 10.1038/s41467-024-49322-w (PMC11186823; doi:10.1038/s41467-024-49322-w)
Supplement: Supplementary file 1 — Supplementary Information [file 41467_2024_49322_MOESM1_ESM.pdf]

Supplementary Information for

C(sp^3)–H sulfinylation of light hydrocarbons with sulfur dioxide *via* hydrogen atom transfer photocatalysis in flow

Dmitrii Nagornîi[±], Fabian Raymenants[±], Nikolaos Kaplaneris, Timothy Noël*

Flow Chemistry Group, Van 't Hoff Institute for Molecular Sciences (HIMS), University of Amsterdam, Amsterdam, The Netherlands.

[±] These authors contributed equally to this work.

*Correspondence to: T.Noel@uva.nl.

This PDF file includes:

Materials and Methods

Supplementary Figures 1 to 6

Supplementary Tables 1 to 9

NMR Data

References

Table of Contents

1. General Information 3
2. Reactor Design 4
   - 2.1 Flow Equipment 4
   - 2.2 Signify Eagle Reactor 5
3. Procedure for Optimization of Gas-Liquid Reactions in Flow 6
   - 3.1 Gas-liquid Reactions at Elevated Pressure with Loop Filling Method 6
   - 3.2 Calculation of Flow Rates for Desired Stoichiometric Ratio (Gas Equivalents) 7
4. Reaction Optimization: Photocatalytic Sulfinylation 9
   - 4.1 Residence Time Screening 9
   - 4.2 Catalyst Loading 10
   - 4.3 Gas Equivalents Screening 10
   - 4.4 Decatungstate Source Screening 11
   - 4.5 Reaction Concentration Screening 11
   - 4.6 Limiting Reagent Screening 12
   - 4.7 Residence Time Screening for Ethane 12
   - 4.8 Optimization for Methane 13
5. Experimental Procedures 15
   - 5.1 Isobutane 15
   - 5.2 *n*-Butane 15
   - 5.3 Propane 15
   - 5.4 Ethane 16
   - 5.5 Methane 16
   - 5.6 Telescope 17
   - 5.7 Scale-up 18
6. Identification of Reaction Intermediates 19
7. Characterization Data 22
   - 7.1 Starting Materials 22
   - 7.2 Isobutane 24
   - 7.3 *n*-Butane 26
   - 7.4 Propane 30
   - 7.5 Ethane 33
   - 7.6 Methane 35
   - 7.7 Functionalization 37
8. NMR Spectra 46
9. References 97

1. General Information

Materials. All reagents and solvents were used as received without further purification. Reagents and solvents were bought from Sigma Aldrich, TCI and Fluorochem. Sulfurous acid (ACS grade, 6.0% SO_2 min) was purchased from Thermo Scientific Chemicals and titrated according to a previously described literature procedure.[1] Geranyl bromide (95% purity) was purchased from Sigma Aldrich. Technical solvents were bought from VWR International and used as received. Isobutane gas with 3.5purity was purchased from Nippon gases, *n*-butane gas with 3.5purity was purchased from Praxair, propane gas with 2.5purity was purchased from Benegas, ethane gas with 3.5purity was purchased from Gerling and Holz and Co and methane gas with 4.5purity was purchased from Nippon gases. The TBADT and NaDT catalysts were prepared according to reported procedures.[2, 3] Disposable syringes were purchased from Laboratory Glass Specialist. Syringe pumps were purchased from Chemix Inc. model Fusion 200 Touch. All capillary tubing, microfluidic fittings and Back Pressure Regulators (BPR) were purchased from IDEX Health & Science. Product isolation was performed automatically, by a Biotage® Isolation Four, with Biotage® SNAP KP-Sil 4 or 10 g flash chromatography cartridges, or manually, using silica (P60, SILICYCLE). TLC analysis was performed using Silica on aluminum foils TLC plates (F254, SILICYCLE) with visualization under ultraviolet light (254 nm and 365 nm) or appropriate TLC staining (potassium permanganate). Organic solutions were concentrated under reduced pressure on a Büchi rotary evaporator (in vacuo at 40 ºC, ~5 mbar).

NMR spectroscopy. ^{1}H (400 and 300 MHz), ^{13}C (101 and 75 MHz) and ^{19}F (282 MHz) spectra were recorded at ambient temperature using Bruker AV 300-I and AV 400. ^{1}H NMR spectra are reported in parts per million (ppm) downfield relative to $CDCl_3$ (7.26 ppm) and all ^{13}C NMR spectra are reported in ppm relative to $CDCl_3$ (77.16 ppm) unless stated otherwise. The multiplicities of signals are designated by the following abbreviations: s (singlet), d (doublet), t (triplet), q (quartet), p (pentet), sext (sextet), m (multiplet), dd (doublet of doublets), dt (doublet of triplets), td (triplet of doublets), ddd (doublet of doublet of doublets). Coupling constants (J) are reported in hertz (Hz). NMR data was processed using the MestReNova 14 software package. Known products were characterized by comparing to the corresponding ^{1}H NMR, ^{13}C NMR with those available in the literature.

Mass spectrometry. High resolution mass spectra (HRMS) were collected on an AccuTOF GC v 4g, JMS-T100GCV Mass spectrometer (JEOL, Japan).

Determination of Regioisomeric and Diastereomeric Ratio. The regioisomeric and diastereomeric ratios were determined by ^{1}H NMR analysis of the crude reaction mixture through integration of diagnostic signals. For cases where the integration of diagnostic signals is not possible, the ratio is calculated after the purification step.

2. Reactor Design

2.1 Flow Equipment

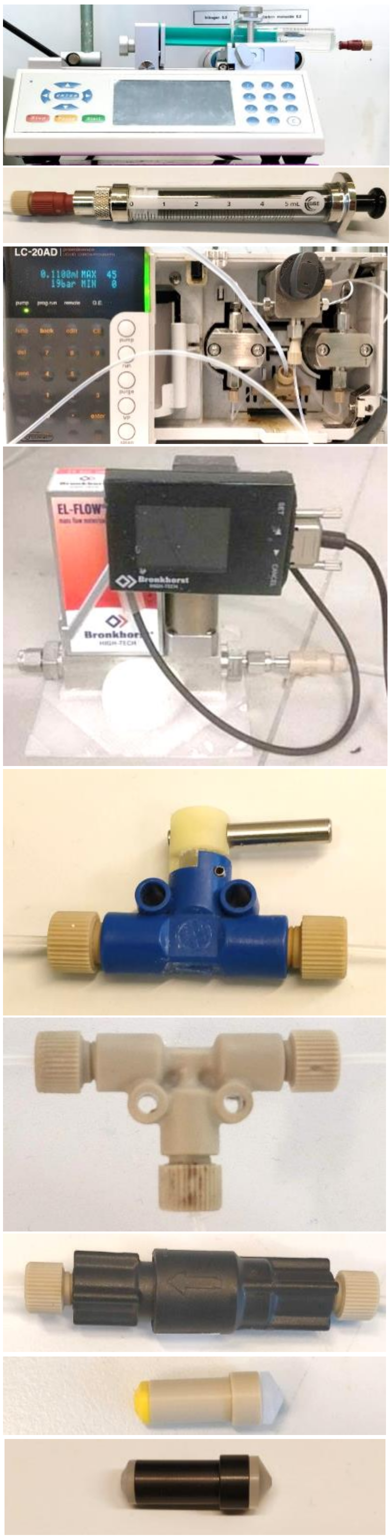

Syringe pump
(Chemyx Fusion 200)

Gastight syringe
(SGE Luer Lock 5

HPLC pump
(Shimadzu LC-20AD)

Mass Flow Controller
(Bronkhorst EL-FLOW)

Shut-off Valve
(IDEX P-783)

T-mixer
(IDEX P-712)

BPR holder
(IDEX P-789

BPR cartridge
(IDEX P-789)

Check valve cartridge
(IDEX CV-3000)

Supplementary Figure 1: Details of flow equipment used for the photocatalytic sulfinylation reactions.

2.2 Signify Eagle Reactor

A Signify photochemical reactor is used, consisting of a base assembly with six 365 nm UV-A chip-on-board light modules.[4] Each of these light source modules contain a fan and a heat sink to efficiently dissipate heat generated through the high power LEDs. Also, the head cap assembly contains blowers to cool the interior of the reactor system, to reduce undesired thermal side-reactions. The LED modules and chamber cooling blowers are connected to a driver box, allowing to set the current of each of the LED modules individually, as well as the rotation speed of the cooling blowers. The six LED modules (365 nm, max. 144 W combined optical output power) are positioned in a hexagonal form around an aluminum cylinder support (80 mm height, 75 mm diameter), which has the reactor coil wrapped around (FEP capillary tubing: 0.5 mm ID, 2 mL volume).

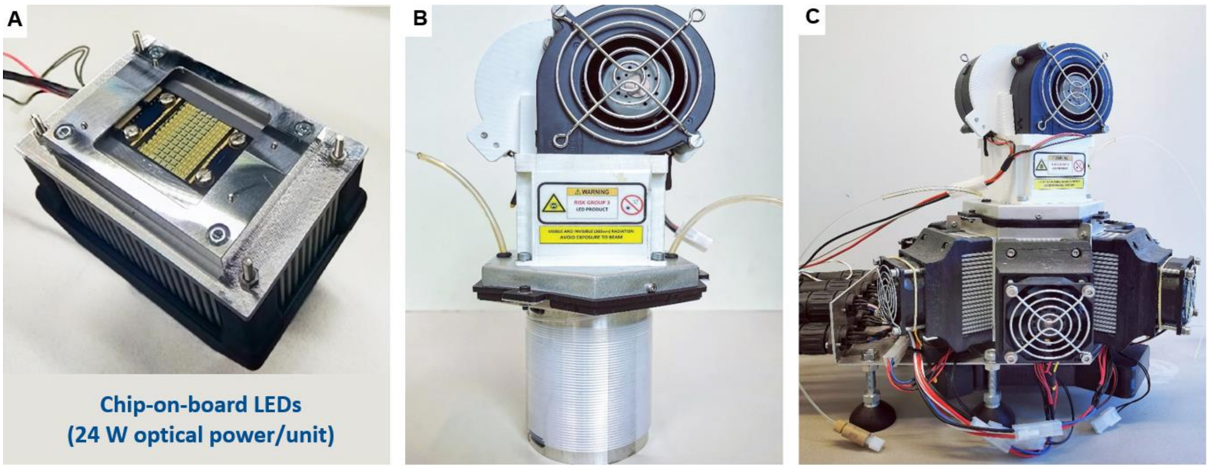

Supplementary Figure 2: Signify Eagle Reactor with six (A) chip-on-board LED modules, (B) head assembly with reactor coil, and (C) complete assembly with fans, heat sinks and LED modules.

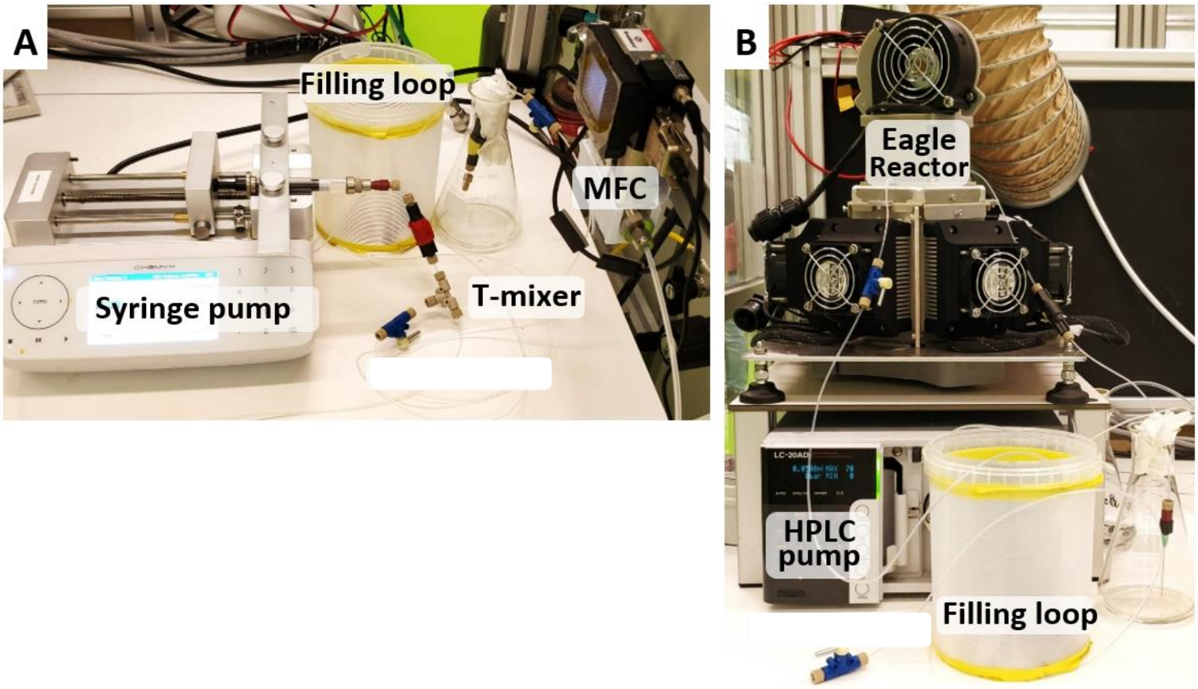

Supplementary Figure 3: Overview of Setup for (A) loop filling of gas- and liquid, (B) photochemical reaction with Signify Eagle Reactor.

3. Procedure for Optimization of Gas-Liquid Reactions in Flow

An elaborate description of the procedure for optimizing gas-liquid and gas-gas-liquid reactions in flow is described in the supporting information of our previous work on photocatalytic carbonylation of light and heavy hydrocarbons.[5] The relevant descriptions and calculations for this work are repeated here.

To ensure close contact between the gas and liquid reaction partners, reactions are performed at elevated pressures, above the liquefaction pressure of the light alkane gases at room temperature (butane: 3 bar, propane: 9 bar, ethane: 35 bar, methane: 46 bar). To work in continuous mode, the pressure of all input streams should be higher than the pressure at the outlet of the reactor. This means that all equipment, which is used for injecting a gas stream (gas cylinder, reducer of gas cylinder and MFC) and for injecting a liquid stream (syringe pump with gastight/disposable syringe, HPLC pump) should be operating at a higher pressure than the design pressure of the BPR and additional pressure drop of the reactor system. Given that the reducer of the gas cylinders is set to relatively low pressures (< 10 bar), all reactions are performed with a loop filling method.

3.1 Gas-liquid Reactions at Elevated Pressure with Loop Filling Method

For reactions above the maximum pressure of the liquid stream (syringe pump or syringe) or above the maximum pressure of the gas stream (pressure of the gas cylinder or reducer), a loop filling method is applied. With this method, the gas and liquid stream are first combined in a filling loop, then pressurized with a HPLC pump and finally injected into the reactor coil under the desired flow rate with the HPLC pump.

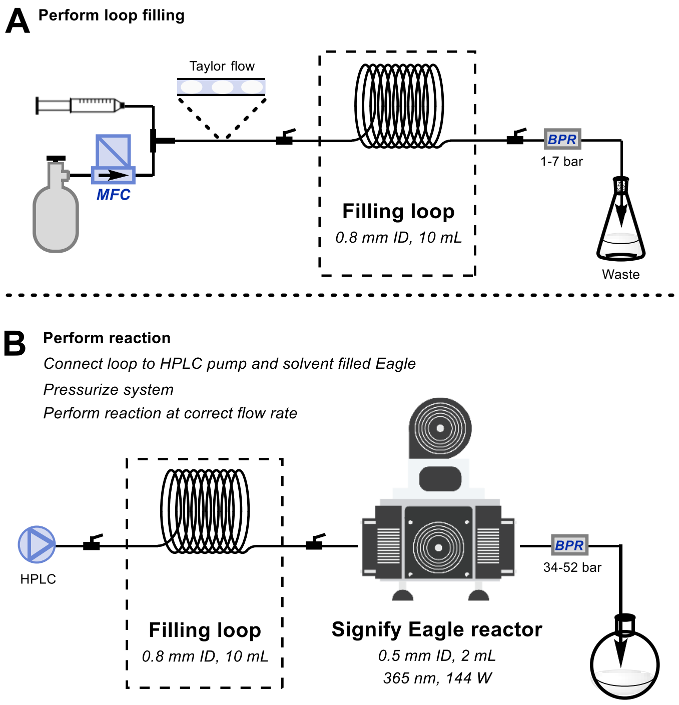

Supplementary Figure 4: Schematic representation of gas-liquid reactions performed with a loop filling method. (A) Loop filling of gas-liquid mixture, (B) Pressurizing system and reaction under correct flow rate. MFC: Mass Flow Controller, CV: Check-Valve, BPR: Back-Pressure Regulator.

The filling loop has shut-off valves connected both sides, allowing the user to contain the gas-liquid mixture inside the loop after injecting the complete reaction mixture. Once the gas-liquid mixture is contained in the filling loop, the outlet shut-off valve is connected to the solvent-filled photoreactor

with the desired BPR at the outlet, while the inlet shut-off valve is connected with a HPLC pump. The outlet valve towards the photoreactor can then slowly be opened, with the purpose to preserve the gas-liquid separation in separate slugs as much as possible. Next, solvent is pushed with the HPLC pump towards the filling loop and photoreactor, causing the capillary tubing between the HPLC pump and BPR to be pressurized. Consequently, the gas segments are compacted, the gas is partially solubilized and eventually liquefied. However, flow over the BPR only starts when the system has reached the design pressure of the BPR. Once this pressure is reached, the flow rate of the HPLC pump is adjusted to achieve the desired residence time. Reaction is then performed through irradiation of the solution inside the reactor and the reaction mixture is collected at the outlet.

3.2 Calculation of Flow Rates for Desired Stoichiometric Ratio (Gas Equivalents)

A tool for calculating gas equivalents was developed by our group to quickly determine the appropriate flow rates for gas-liquid reactions in flow. This tool can be accessed through the following link: https://noel-research-group-streamlit-problem-session-main-esrber.streamlit.app/.

Below the formulas are also deduced and the basic principles are explained, which are used to calculate the required flow rates for a desired stoichiometric ratio of gaseous reagents. For calculations of the required flow rates in continuous mode, see the supporting information of our previous work.[5]

With the loop filling method, the stoichiometric ratio, or the amount of gas equivalents, is determined by the ratio of the molar flow rates. Pressure (and volumes) are not relevant.

$$Eq_{gas} = \frac{Gas\ molar\ flow\ rate}{Substrate\ molar\ flow\ rate} = \frac{\dot{n}_{gas}}{\dot{n}_{substate}}$$

Supplementary equation 1

The molar flow rate of the substrate is:

$$\dot{n}_{substrate} = c_{substrate} \cdot \dot{v}_{liquid}$$

Supplementary equation 2

The volumetric flow rate of gas that needs to be applied on the MFC (display gives volumetric flow rate at STP) for a desired stoichiometric ratio can then be calculated as:

$$\dot{v}_{gas} = \frac{\dot{n}_{gas}}{\rho_{molar,gas}} = \frac{MW_{gas}}{\rho_{mass,gas}} \cdot \dot{n}_{gas}$$

Supplementary equation 3

For delivering gas to the flow system, a Bronkhorst EL-FLOW is used, a thermal Mass Flow Controller for gasses using the bypass principle. The provider calibrated the instrument using nitrogen and a conversion factor is included internally into the system, to provide flow rates for alkane gases at standard temperature and pressure (STP: 0°C, 1.013 bar). Other gasses can also be used, but a conversion factor needs to be applied by the user to account for differences in heat capacity and mass density at normal conditions.

For the loop filling method, absolute flow rates are not important. As the gas and liquid streams are mixed inside a filling loop first and not directly fed into the reactor, the absolute gas and liquid flow rates do not determine residence time.

As an example, to calculate the required gas flow rate that should be applied to the MFC to reach 5 equivalents of propane during loop filling, and choosing a substrate (SO_2) concentration of 0.2 M and liquid flow rate of 0.1 mL·min^{-1}, the above formulas can be applied:

$$\dot{v}_{gas} = \frac{MW_{Propane}}{\rho_{mass,Propane}} \cdot \dot{n}_{Propane} = \frac{MW_{Propane}}{\rho_{mass,Propane}} \cdot Eq_{Propane} \cdot c_{substrate} \cdot \dot{v}_{liquid}$$

Supplementary equation 4

$$\dot{v}_{gas} = \frac{44.1\ g \cdot mol^{-1}}{1.808\ g \cdot L^{-1}} \cdot 5 \cdot 0.2\ mol \cdot L^{-1} \cdot 0.1\ mL \cdot min^{-1} = 2.44\ mL_{n,Propane} \cdot min^{-1}$$

Supplementary equation 5

Under the abovementioned reaction conditions, a gas-to-liquid ratio $\dot{v}_{gas}/\dot{v}_{liquid}$ of 24.4:1 is thus required to achieve 5 equivalents of propane compared to SO_2.

4. Reaction Optimization: Photocatalytic Sulfinylation

The reaction between propane and SO_2 is chosen as model reaction and an initial screening of reaction parameters is performed in a Signify Eagle Reactor (365 nm, 144 W output power) as described above. At the outlet of the photoreactor, the intermediate sulfinic acid is trapped with benzyl bromide in the presence of base in a fed-batch approach.

4.1 Residence Time Screening

From our previous experience with photocatalytic functionalization of light alkanes, we decided to perform the reaction under increased pressure to ensure liquefaction of the gaseous reaction partner.[6]

To begin our investigation, a residence time screening is performed for the photocatalytic reaction between propane and SO_2 (Supplementary Table 1). Best result was observed with 1 h residence time, giving 48% ^{1}H NMR yield of product **1** after trapping of the sulfinate with benzyl bromide.

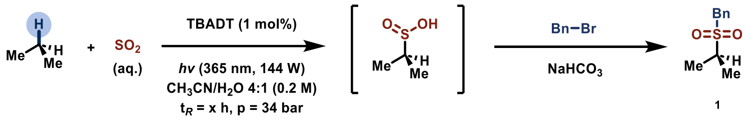

Supplementary Table 1: Residence Time Screening for the reaction of SO_2 and propane.

Entry[a]	t_R [h]	Yield[b]
1	0.5	41%
2	1	48%
3	2	46%
4	4	25%

[a] Reaction conditions: A stock solution is prepared with 0.4 mmol of SO_2 (1.0 M, aqueous solution) and 1 mol% of TBADT dissolved in a 1.6 mL of CH_3CN (resulting in a solution of CH_3CN/water 4:1, 0.2 M). The mixture is combined with propane gas (5 equiv. vs SO_2), pressurized (34 bar) and pushed over the photoreactor (365 nm, 144 W) with a varying residence time (0.5–2 h). The outflow of the reactor is collected in a vial containing benzyl bromide (0.6 mmol, 1.5 equiv.) and $NaHCO_3$ (84 mg, 1.0 mmol). The resulting mixture is stirred overnight and the amount of product **1** formed, is evaluated. [b] ^{1}H-NMR yields are calculated with an external standard (trichloroethylene).

4.2 Catalyst Loading

Next the influence of catalyst loading is investigated. 1 mol% catalyst loading gave the best result, with reduced yield of product **1** observed with lower and higher catalyst loading (Supplementary Table 2).

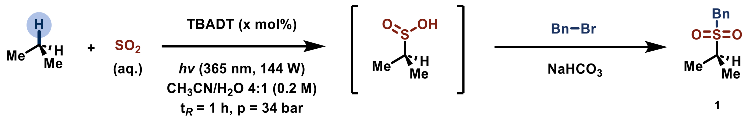

Supplementary Table 2: Catalyst loading screening for the reaction of SO_2 and propane.

Entry[a]	Catalyst loading [mol%]	Yield[b]
1	0.5	18%
2	1	48%
3	2	44%
4	3	34%

[a] Reaction conditions: A stock solution is prepared with 0.4 mmol of SO_2 (1.0 M, aqueous solution) and 0.5–3 mol% of TBADT dissolved in a 1.6 mL of CH_3CN (resulting in a solution of CH_3CN/water 4:1, 0.2 M). The mixture is combined with propane gas (5 equiv. vs SO_2), pressurized (34 bar) and pushed over the photoreactor (365 nm, 144 W) with a 1 h residence time. The outflow of the reactor is collected in a vial containing benzyl bromide (0.6 mmol, 1.5 equiv.) and $NaHCO_3$ (84 mg, 1.0 mmol). The resulting mixture is stirred overnight and the amount of product **1** formed, is evaluated. [b] ^{1}H-NMR yields are calculated with an external standard (trichloroethylene).

4.3 Gas Equivalents Screening

A screening of the gas equivalents showed optimal results with 5 equivalents of propane, with similar yield obtained with 10 equivalents and a significant drop in yield with 20 equivalents (Supplementary Table 3).

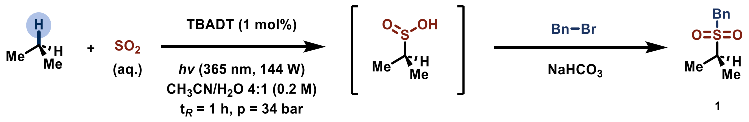

Supplementary Table 3: Gas equivalents screening for the reaction of SO_2 and propane.

Entry[a]	Gas equivalents [eq.]	Yield[b]
1	2.5	19%
2	5	48%
3	10	45%
4	20	20%

[a] Reaction conditions: A stock solution is prepared with 0.4 mmol of SO_2 (1.0 M, aqueous solution) and 1 mol% of TBADT dissolved in a 1.6 mL of CH_3CN (resulting in a solution of CH_3CN/water 4:1, 0.2

M). The mixture is combined with propane gas (2.5–20 equiv. vs SO_2), pressurized (34 bar) and pushed over the photoreactor (365 nm, 144 W) with a 1 h residence time. The outflow of the reactor is collected in a vial containing benzyl bromide (0.6 mmol, 1.5 equiv.) and $NaHCO_3$ (84 mg, 1.0 mmol). The resulting mixture is stirred overnight and the amount of product **1** formed, is evaluated. [b] ^{1}H-NMR yields are calculated with an external standard (trichloroethylene).

4.4 Decatungstate Source Screening

The counterion of the decatungstate photocatalyst plays a determining role in its solubility in the reaction mixture. By switching from tetrabutyl ammonium as counterion to sodium, a significant increase in yield was observed, leading to 59% ^{1}H NMR yield of product **1** with NaDT as photocatalyst (Supplementary Table 4).

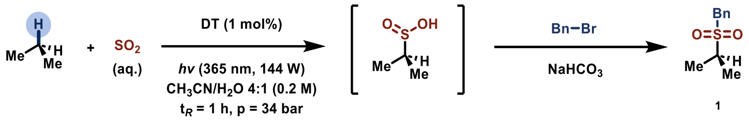

Supplementary Table 4: Decatungstate source screening for the reaction of SO_2 and propane.

Entry[a]	Decatungstate source	Yield[b]
1	TBADT	48%
2	NaDT	59%

[a] Reaction conditions: A stock solution is prepared with 0.4 mmol of SO_2 (1.0 M, aqueous solution) and 1 mol% of decatungstate catalyst dissolved in a 1.6 mL of CH_3CN (resulting in a solution of CH_3CN/water 4:1, 0.2 M). The mixture is combined with propane gas (5 equiv. vs SO_2), pressurized (34 bar) and pushed over the photoreactor (365 nm, 144 W) with a 1 h residence time. The outflow of the reactor is collected in a vial containing benzyl bromide (0.6 mmol, 1.5 equiv.) and $NaHCO_3$ (84 mg, 1.0 mmol). The resulting mixture is stirred overnight and the amount of product **1** formed, is evaluated. [b] ^{1}H-NMR yields are calculated with an external standard (trichloroethylene).

4.5 Reaction Concentration Screening

Next, we investigated the influence of lowering the concentration of SO_2. Lowering the SO_2 concentration to 0.1 M resulted in a reduced yield of 43% of product **1** (Supplementary Table 5).

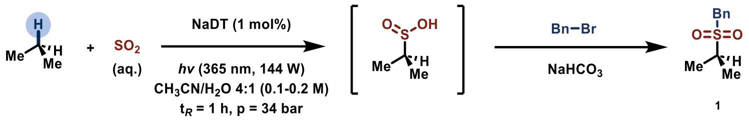

Supplementary Table 5: Screening concentration of SO_2 for the reaction of SO_2 and propane.

Entry[a]	Concentration SO_2 [M]	Yield[b]
1	0.2	59%
2	0.1	43%

[a] Reaction conditions: A stock solution is prepared with 0.2–0.4 mmol of SO_2 (1.0 M, aqueous solution) and 1 mol% of NaDT dissolved in a 1.6 mL of CH_3CN or 1.6 mL CH_3CN and 0.2 mL water (resulting in a solution of CH_3CN/water 4:1, 0.1–0.2 M). The mixture is combined with propane gas (5 equiv. vs SO_2), pressurized (34 bar) and pushed over the photoreactor (365 nm, 144 W) with a 1 h residence time. The outflow of the reactor is collected in a vial containing benzyl bromide (0.6 mmol, 1.5 equiv.) and $NaHCO_3$ (84 mg, 1.0 mmol). The resulting mixture is stirred overnight and the amount of product **1** formed, is evaluated. [b] ^{1}H-NMR yields are calculated with an external standard (trichloroethylene).

4.6 Limiting Reagent Screening

Due to the higher cost and lower abundance of the electrophilic reagent (benzyl bromide) in comparison to SO_2, the electrophile was chosen as the limiting reagent of the reaction, resulting in an isolated yield of 95% of product **1** (Supplementary Table 6).

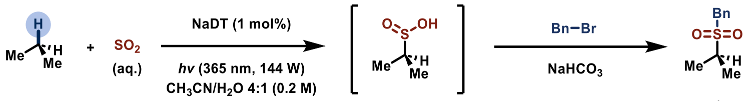

Supplementary Table 6: Limiting reagent screening for the reaction of SO_2 and propane.

Entry[a]	Limiting reagent	Yield[c]
1[a]	SO_2	59%
2[b]	BnBr	95%[d]

[a] Reaction conditions: A stock solution was prepared with 0.4 mmol of SO_2 (1.0 M, aqueous solution, 1 equiv.) and 1 mol% of NaDT catalyst dissolved in a 1.6 mL of CH_3CN (resulting in a solution of CH_3CN/water 4:1, 0.2 M). The mixture was combined with propane gas (5 equiv. vs SO_2), pressurized (34 bar) and pushed over the photoreactor (365 nm, 144 W) with a 1 h residence time. The outflow of the reactor was collected in a vial containing benzyl bromide (0.6 mmol, 1.5 equiv.) and $NaHCO_3$ (84 mg, 1.0 mmol). The resulting mixture was stirred overnight and the amount of product **1** formed, was evaluated. [b] Reaction conditions: A stock solution was prepared with 0.6 mmol of SO_2 (1.0 M, aqueous solution, 3 equiv.) and 1 mol% of NaDT catalyst dissolved in a 2.4 mL of CH_3CN (resulting in a solution of CH_3CN/water 4:1, 0.2 M). The mixture was combined with propane gas (5 equiv. vs SO_2), pressurized (34 bar) and pushed over the photoreactor (365 nm, 144 W) with a 1 h residence time. The outflow of the reactor was collected in a vial containing benzyl bromide (0.2 mmol, 1 equiv.) and $NaHCO_3$ (100 mg, 1.2 mmol). The resulting mixture was stirred overnight and the amount of product **1** formed, was evaluated. [c] ^{1}H-NMR yields are calculated with an external standard (trichloroethylene). [d] Isolated yield.

4.7 Residence Time Screening for Ethane

For the reaction with ethane gas, the residence time screening was repeated, showing optimal yield after 2 h residence time (Supplementary Table 7).

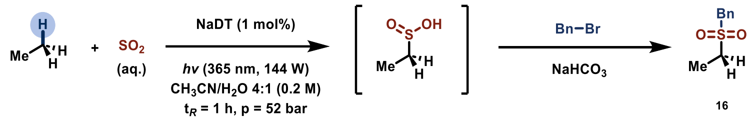

Supplementary Table 7: Residence Time Screening for the reaction of SO_2 and ethane.

Entry[a]	t_R [h]	Yield[b]
1	1	35%
2	2	62%
3	4	38%

[a] Reaction conditions: A stock solution is prepared with 0.4 mmol of SO_2 (1.0 M, aqueous solution) and 1 mol% of NaDT dissolved in a 1.6 mL of CH_3CN (resulting in a solution of CH_3CN/water 4:1, 0.2 M). The mixture is combined with ethane gas (5 equiv. vs SO_2), pressurized (52 bar) and pushed over the photoreactor (365 nm, 144 W) with a varying residence time (1-4 h). The outflow of the reactor is collected in a vial containing benzyl bromide (0.6 mmol, 1.5 equiv.) and $NaHCO_3$ (84 mg, 1.0 mmol). The resulting mixture is stirred overnight and the amount of product **16** formed, is evaluated. [b] ^{1}H-NMR yields are calculated with an external standard (trichloroethylene).

4.8 Optimization for Methane

Based on our previous experience with methane functionalization, we expected that we may need longer residence times and lower concentrations than for other light alkane gases. For the first trials, we therefore lowered the concentration of SO_2 and maintained the same amount of concentration of alkane gas and photocatalyst, resulting in the use of 10 equivalents of methane compared to the limiting reagent SO_2 and 2 mol% catalyst loading (Supplementary Table 8). After the second step with 1.5 equivalents of benzyl bromide, the product was detected in GCMS and characteristic peaks were observed in the ^{1}H NMR of the crude mixture. However, amounts were small, making quantification difficult. Changing CH_3CN for its deuterated alternative, did not result in a large increase in yield.

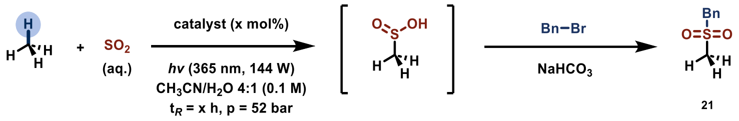

Supplementary Table 8: Residence Time Screening for the reaction of SO_2 and methane.

Entry[a]	t_R [h]	[SO_2]	Me eq.	Cat. loading	Solvent	Yield[b]
1	6	0.1 M	10	NaDT 2%	CH_3CN/H_2O 4:1	<5%
2	8	0.1 M	10	NaDT 2%	CD_3CN/H_2O 4:1	<5%
3	6	0.1 M	10	$FeCl_3.6H_2O$ 20%	CH_3CN/H_2O 4:1	<5%

[a] Reaction conditions: A stock solution is prepared with 0.2 mmol of SO_2 (1.0 M, aqueous solution) and 2 mol% of NaDT or 20 mol% $FeCl_3.6H_2O$ dissolved in a 1.6 mL of CH_3CN and 0.2 mL of water (resulting in a solution of CH_3CN/water 4:1, 0.1 M). The mixture is combined with methane gas (10 equiv. vs SO_2), pressurized (52 bar) and pushed over the photoreactor (365 nm, 144 W) with a varying

residence time (6–8 h). The outflow of the reactor is collected in a vial containing benzyl bromide (0.3 mmol, 1.5 equiv.) and $NaHCO_3$ (33 mg, 0.4 mmol). The resulting mixture is stirred overnight and the amount of product **21** formed, is evaluated. [b] ^{1}H-NMR yields are calculated with an external standard (trichloroethylene).

When benzyl bromide was used as the limiting reagent of the reaction, along with an increased amount of SO_2 (2 mmol), 8% of product **21** was obtained (Supplementary Table 9). The use of CD_3CN as reaction solvent led to a marginally better yield and a cleaner ^{1}H NMR spectrum. Higher catalyst loading did not improve the yield of the reaction. Finally, when a fresh and recently titrated SO_2 solution (1M) was used, a 52% ^{1}H NMR yield was observed. Further increasing the amount of SO_2 to 4 mmol, resulted in a ^{1}H NMR of 70% and an isolated yield of 63%.

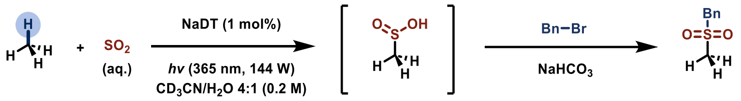

Supplementary Table 9: Residence Time Screening for the reaction of SO_2 and methane.

Entry[a]	Deviation	Yield[b]	Comment
1	CH_3CN/H_2O 4:1, 2 mmol SO_2	8%[c]	Significant side product formation
2	CD_3CN/H_2O 4:1	12%[c]	Cleaner crude ^{1}H NMR
3	4 mol% NaDT	11%[c]	
4	1 mol% NaDT, CD_3CN/H_2O 4:1, 2 mmol SO_2	52% (29%)[d]	Fresh SO_2 solution
5	4 mmol SO_2	70% (63%)[d]	

[a] Reaction conditions: A stock solution is prepared with 2-4 mmol of SO_2 (1.0 M, aqueous solution) and 1 mol% of NaDT dissolved in 8-16 mL of CD_3CN (resulting in a solution of CD_3CN/water 4:1, 0.2 M). The mixture is combined with methane gas (5 equiv. vs SO_2), pressurized (52 bar) and pushed over the photoreactor (365 nm, 144 W) with a 2 h residence time. The outflow of the reactor is collected in a vial containing benzyl bromide (0.2 mmol, 1 equiv.) and $NaHCO_3$ (600 mg, 7.1 mmol). The resulting mixture is stirred overnight and the amount of product **21** formed, is evaluated. [b] ^{1}H-NMR yields are calculated with an external standard (trichloroethylene). [c] Reactions performed with an old solution of SO_2 in water. [d] Isolated yield is noted in parentheses.

5. Experimental Procedures

5.1 Isobutane

To a nitrogen-purged, screw-capped vial, fitted with a rubber septum and charged with NaDT (14.65 mg, 6 µmol, 3 mol%) degassed CH_3CN is added (2.4 mL), followed by aqueous SO_2 (6 wt%, 0.6 mL, 0.6 mmol, 3 equiv.). The stock solution is charged in a gastight syringe, positioned in a syringe pump and combined with a stream of isobutane gas (70 mL, 3 mmol, 15 equiv.) through a T-mixer into a filling loop, with a liquid flow rate of 0.16 mL·min-1 and an isobutane gas flow rate of 4 mL·min-1. Next, the filling loop is connected to the reactor, the system is pressurized to 34 bar using an HPLC pump and the reaction mixture is pumped over the Signify Eagle reactor (365 nm, 144 W output power, FEP capillary: 0.5 mm ID, 1.5 mL) at a flow rate of 0.025 mL·min-1, resulting in a residence time of 1 h. The obtained reaction mixture is collected into a nitrogen-purged vial containing $NaHCO_3$ (100 mg, 1.2 mmol, 6 equiv.), alkyl bromide (0.2 mmol, 1 equiv.) and degassed CH_3CN (1 mL). The reaction mixture is stirred at room temperature during the collection of the outflow, and the stirring is continued at the specified temperature. Then, the reaction mixture is transferred to a separatory funnel, diluted with water (20 mL) and extracted with DCM (3x20 mL). The combined organic layers are dried over $MgSO_4$ and evaporated in vacuo. The residue is purified by column chromatography (*n*-pentane:AcOEt).

5.2 *n*-Butane

To a nitrogen-purged, screw-capped vial, fitted with a rubber septum and charged with NaDT (14.65 mg, 6 µmol, 3 mol%) degassed CH_3CN is added (2.4 mL), followed by aqueous SO_2 (6 wt%, 0.6 mL, 0.6 mmol, 3 equiv.). The stock solution is charged in a gastight syringe, positioned in a syringe pump and combined with a stream of butane gas (70 mL, 3 mmol, 15 equiv.) through a T-mixer into a filling loop, with a liquid flow rate of 0.16 mL·min-1 and a butane gas flow rate of 4 mL·min-1. Next, the filling loop is connected to the reactor, the system is pressurized to 34 bar using an HPLC pump and the reaction mixture is pumped over the Signify Eagle reactor (365 nm, 144 W output power, FEP capillary: 0.5 mm ID, 1.5 mL) at a flow rate of 0.025 mL·min-1, resulting in a residence time of 1 h. The obtained reaction mixture is collected into a nitrogen-purged vial containing $NaHCO_3$ (100 mg, 1.2 mmol, 6 equiv.), alkyl bromide (0.2 mmol, 1 equiv.) and degassed CH_3CN (1 mL). The reaction mixture is stirred at room temperature during the collection of the outflow, and the stirring is continued at the specified temperature. Then, the reaction mixture is transferred to a separatory funnel, diluted with water (20 mL) and extracted with DCM (3x20 mL). The combined organic layers are dried over $MgSO_4$ and evaporated in vacuo. The residue is purified by column chromatography (*n*-pentane:AcOEt).

5.3 Propane

To a nitrogen-purged, screw-capped vial, fitted with a rubber septum and charged with NaDT (14.65 mg, 6 µmol, 3 mol%) degassed CH_3CN is added (2.4 mL), followed by aqueous SO_2 (6 wt%, 0.6 mL, 0.6 mmol, 3 equiv.). The stock solution is charged in a gastight syringe, positioned in a syringe pump and combined with a stream of propane gas (73.5 mL, 3 mmol, 15 equiv.) through a T-mixer into a filling loop, with a liquid flow rate of 0.16 mL·min-1 and a propane gas flow rate of 4 mL·min-1. A BPR of 2.8 bar is used during the loop filling. Next, the filling loop is connected to the reactor, the

system is pressurized to 34 bar using an HPLC pump and the reaction mixture is pumped over the Signify Eagle reactor (365 nm, 144 W output power, FEP capillary: 0.5 mm ID, 1.5 mL) at a flow rate of 0.025 mL·min-1, resulting in a residence time of 1 h. The obtained reaction mixture is collected into a nitrogen-purged vial containing $NaHCO_3$ (100 mg, 1.2 mmol, 6 equiv.), alkyl bromide (0.2 mmol, 1 equiv.) and degassed CH_3CN (1 mL). The reaction mixture is stirred at room temperature during the collection of the outflow, and the stirring is continued at the specified temperature. Then, the reaction mixture is transferred to a separatory funnel, diluted with water (20 mL) and extracted with DCM (3x20 mL). The combined organic layers are dried over $MgSO_4$ and evaporated in vacuo. The residue is purified by column chromatography (*n*-pentane:AcOEt).

5.4 Ethane

To a nitrogen-purged, screw-capped vial, fitted with a rubber septum and charged with NaDT (14.65 mg, 6 µmol, 3 mol%) degassed CH_3CN is added (2.4 mL), followed by aqueous SO_2 (6 wt%, 0.6 mL, 0.6 mmol, 3 equiv.). The stock solution is charged in a gastight syringe, positioned in a syringe pump and combined with a stream of ethane gas (70.5 mL, 3 mmol, 15 equiv.) through a T-mixer into a filling loop, with a liquid flow rate of 0.16 mL·min-1 and an ethane gas flow rate of 4 mL·min-1. A BPR of 2.8 bar is used during the loop filling. Next, the filling loop is connected to the reactor, the system is pressurized to 52 bar using an HPLC pump and the reaction mixture is pumped over the Signify Eagle reactor (365 nm, 144 W output power, FEP capillary: 0.5 mm ID, 1.5 mL) at a flow rate of 0.0125 mL·min-1, resulting in a residence time of 2 h. The obtained reaction mixture is collected into a nitrogen-purged vial containing $NaHCO_3$ (100 mg, 1.2 mmol, 6 equiv.), alkyl bromide (0.2 mmol, 1 equiv.) and degassed CH_3CN (1 mL). The reaction mixture is stirred at room temperature during the collection of the outflow, and the stirring is continued at the specified temperature. Then, the reaction mixture is transferred to a separatory funnel, diluted with water (20 mL) and extracted with DCM (3x20 mL). The combined organic layers are dried over $MgSO_4$ and evaporated in vacuo. The residue is purified by column chromatography (*n*-pentane:AcOEt).

5.5 Methane

To a nitrogen-purged, screw-capped vial, fitted with a rubber septum and charged with NaDT (97.67 mg, 40 µmol, 20 mol%) degassed CH_3CN is added (16 mL), followed by aqueous SO_2 (6 wt%, 4.0 mL, 4.0 mmol, 20 equiv.). The stock solution is charged in a gastight syringe, positioned in a syringe pump and combined with a stream of methane gas (447.6 mL, 20 mmol, 100 equiv.) through a T-mixer into a filling loop, with a liquid flow rate of 0.16 mL·min-1 and a methane gas flow rate of 4 mL·min-1. A BPR of 2.8 bar is used during the loop filling. Next, the filling loop is connected to the reactor, the system is pressurized to 52 bar using an HPLC pump and the reaction mixture is pumped over the Signify Eagle reactor (365 nm, 144 W output power, FEP capillary: 0.5 mm ID, 1.5 mL) at a flow rate of 0.0125 mL·min-1, resulting in a residence time of 2 h. The obtained reaction mixture is collected into a nitrogen-purged vial containing $NaHCO_3$ (667 mg, 8.0 mmol, 40 equiv.), alkyl bromide (0.2 mmol, 1 equiv.) and degassed CH_3CN (1 mL). The reaction mixture is stirred at room temperature during the collection of the outflow, and the stirring is continued at the specified temperature. Then, the reaction mixture is transferred to a separatory funnel, diluted with water (20 mL) and extracted with DCM (3x20 mL). The combined organic layers are dried over $MgSO_4$ and evaporated in vacuo. The residue is purified by column chromatography (*n*-pentane:AcOEt).

5.6 Telescope

To a nitrogen-purged, screw-capped vial, fitted with a rubber septum and charged with NaDT (14.65 mg, 6 µmol, 3 mol%) degassed CH_3CN is added (2.4 mL), followed by aqueous SO_2 (6 wt%, 0.6 mL, 0.6 mmol, 3 equiv.). The SO_2 solution is charged in a gastight syringe, positioned in a syringe pump and combined with a stream of propane gas (73.5 mL, 3 mmol, 15 equiv.) through a T-mixer into a filling loop, with a liquid flow rate of 0.16 mL·min-1 and a propane gas flow rate of 4 mL·min-1. A BPR of 2.8 bar is used during the loop filling. Additionally, to a nitrogen-purged, screw-capped vial, fitted with a rubber septum and charged with benzyl bromide (34.2 mg, 23.8 µL, 0.2 mmol, 1 equiv.) and 2,6-lutidine (214 mg, 232.0 µL, 1.0 mmol, 10 equiv.) degassed CH_3CN is added (2 mL). The benzyl bromide solution was charged into a second filling loop, with one end connected to an HPLC pump and the other end connected to the outlet of the photoreactor through a T-mixer, with a shut-off valve positioned immediately before the T-mixer. Next, the SO_2 filling loop is connected to the photoreactor, the system is pressurized to 34 bar using an HPLC pump (while the shut-off valve is closed) and the reaction mixture is pumped over the Signify Eagle reactor (365 nm, 144 W output power, FEP capillary: 0.5 mm ID, 2.8 mL) at a flow rate of 0.046 mL·min-1, resulting in a residence time of 1 h. At the same time, the benzyl bromide filling loop is also pressurized to 34 bar using an HPLC pump. When the reaction mixture reaches the T-mixer, the shut-off valve is open and the benzyl bromide solution is pushed at a flow rate of 0.031 mL·min-1, resulting in 3:1 SO_2:benzyl bromide ratio and a total flow rate of 0.077 mL·min-1. This mixture is pumped into a final reactor loop (4.6 mL), resulting in a residence time of 1 h. Then, the collected outflow is transferred to a separatory funnel, diluted with water (20 mL) and extracted with DCM (3x20 mL). The combined organic layers are dried over $MgSO_4$ and evaporated in vacuo. The crude mixture was purified by flash column chromatography (100% *n*-pentane to *n*-pentane 80:20 AcOEt) to afford product **1a** and **1b** (80:20 ratio determined by ^{1}H NMR analysis of the crude reaction mixture), (34.1 mg, 86%) as a clear oil.

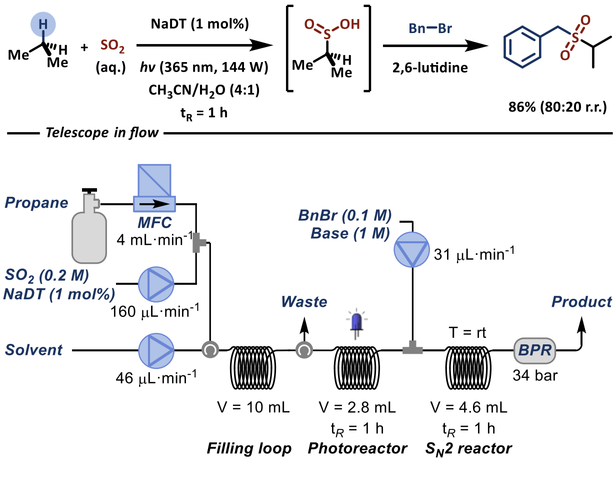

Supplementary Figure 5: Schematic representation of the setup for telescope reaction in flow.

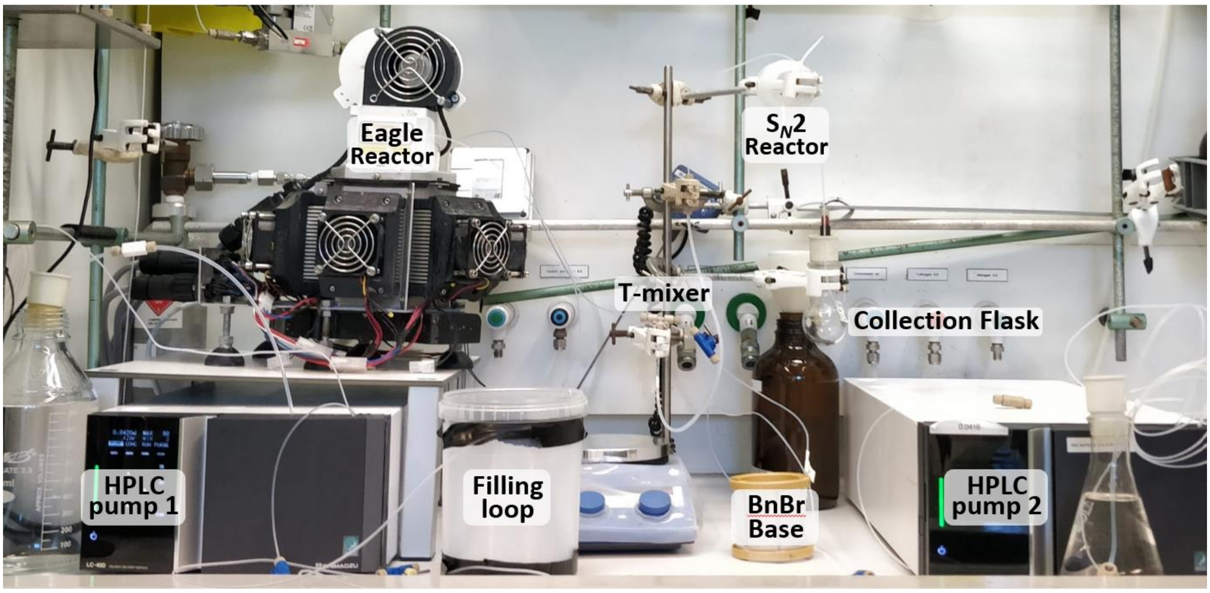

Supplementary Figure 6: Picture of setup for telescope reaction in flow.

5.7 Scale-up

To a nitrogen-purged, screw-capped vial, fitted with a rubber septum and charged with NaDT (73.25 mg, 30 µmol, 3 mol%) degassed CH_3CN is added (12 mL), followed by aqueous SO_2 (6 wt%, 3.0 mL, 3.0 mmol, 3 equiv.). The stock solution is charged in a gastight syringe, positioned in a syringe pump and combined with a stream of propane gas (367.5 mL, 15.0 mmol, 15 equiv.) through a T-mixer into a filling loop, with a liquid flow rate of 0.16 mL·min-1 and a propane gas flow rate of 4 mL·min-1. A BPR of 2.8 bar is used during the loop filling. Next, the filling loop is connected to the reactor, the system is pressurized to 34 bar using an HPLC pump and the reaction mixture is pumped over the Signify Eagle reactor (365 nm, 144 W output power, FEP capillary: 0.5 mm ID, 1.5 mL) at a flow rate of 0.025 mL·min-1, resulting in a residence time of 1 h. The obtained reaction mixture is collected into a nitrogen-purged flask containing $NaHCO_3$ (500 mg, 6 mmol, 6 equiv.), S1 (356 mg ,1 mmol, 1 equiv.) and degassed CH_3CN (5 mL). The reaction mixture is stirred at room temperature during the collection of the outflow, and then for 1 hour at 60 °C. Then, the reaction mixture is transferred to a separatory funnel, diluted with water (40 mL) and extracted with DCM (3x40 mL). The combined organic layers are dried over $MgSO_4$ and evaporated in vacuo. The crude mixture was purified by flash column chromatography (100% *n*-pentane to *n*-pentane 75:25 AcOEt) to afford product **15a** and **15b** as an inseparable mixture of regioisomers (80:20 ratio determined by ^{1}H NMR analysis of the crude reaction mixture), (314.1 mg, 82%) as a white solid.

6. Identification of Reaction Intermediates

To identify the reaction intermediates, a mixture of propane and SO_2 were reacted according to the General Procedure for propane, with the outflow of the Signify Eagle reactor collected in a vial without further functionalization (no benzyl bromide and base added). The crude was evaporated in vacuo and dissolved in D_2O for NMR analysis. The ^1H NMR spectrum revealed a mixture of sulfinic acid and sulfinate salt of the corresponding isopropyl and *n*-propyl adducts (Supplementary Figure 7).

Isopropane sulfinic acid

^1H NMR (400 MHz, D_2O) δ 2.96 (hept, J = 6.9 Hz, 1H), 1.20 (d, J = 6.8 Hz, 5H).

n-propane sulfinic acid

^1H NMR (400 MHz, D_2O) δ 2.85 – 2.77 (m, 2H), 1.76 – 1.60 (m, 2H), 0.93 (t, J = 7.5 Hz, 3H).

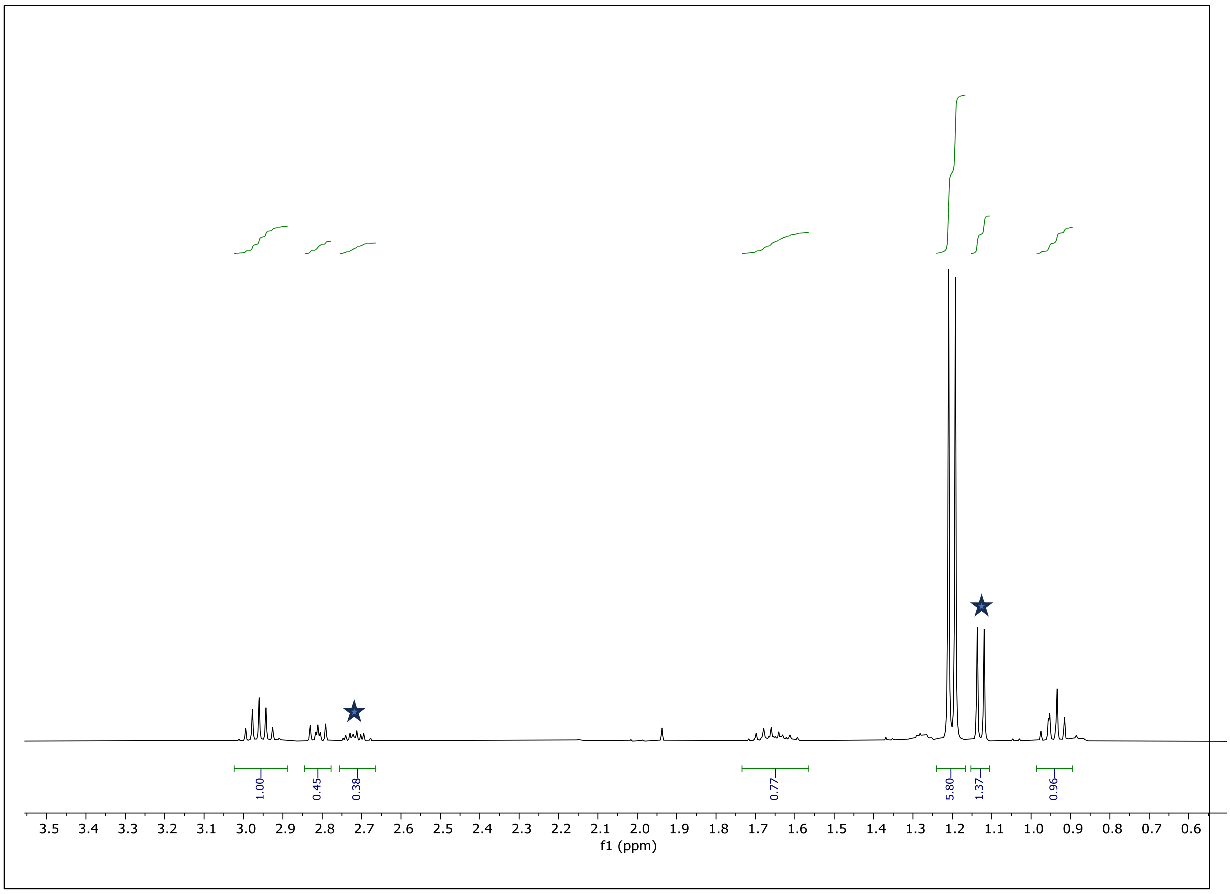

Supplementary Figure 7: Crude ^1H NMR of the reaction between propane and SO_2 (peaks denoted with asterisk correspond to sulfinate species)

The intermediate sulfinic acid was isolated by acidifying the crude mixture 1M HCl and extracted with MTBE, dried and evaporated in vacuo. The ^1H and ^{13}C NMR analysis confirmed the presence of isopropyl and *n*-propyl sulfinic acid, by comparing the spectra with those available in literature (Supplementary Figure 8 and 9).[7] Additionally, GC–MS analysis further confirmed the molecular mass of the intermediates, in accordance with previous literature.[8]

Isobutane sulfinic acid

^{1}H NMR (400 MHz, D_2O) δ 2.96 (hept, J = 6.9 Hz, 1H), 1.20 (d, J = 6.8 Hz, 5H).

^{13}C NMR (101 MHz, D_2O) δ 51.0, 16.6.

GCMS (EI+) (m/z): $[M]^+$ calcd. for $C_3H_8O_2S$, 108.0; found: 108.0.

***n*-propane sulfinic acid**

^{1}H NMR (400 MHz, D_2O) δ 2.85 – 2.77 (m, 2H), 1.76 – 1.60 (m, 2H), 0.93 (t, J = 7.5 Hz, 3H).

^{13}C NMR (101 MHz, D_2O) δ 52.8, 17.7, 12.3.

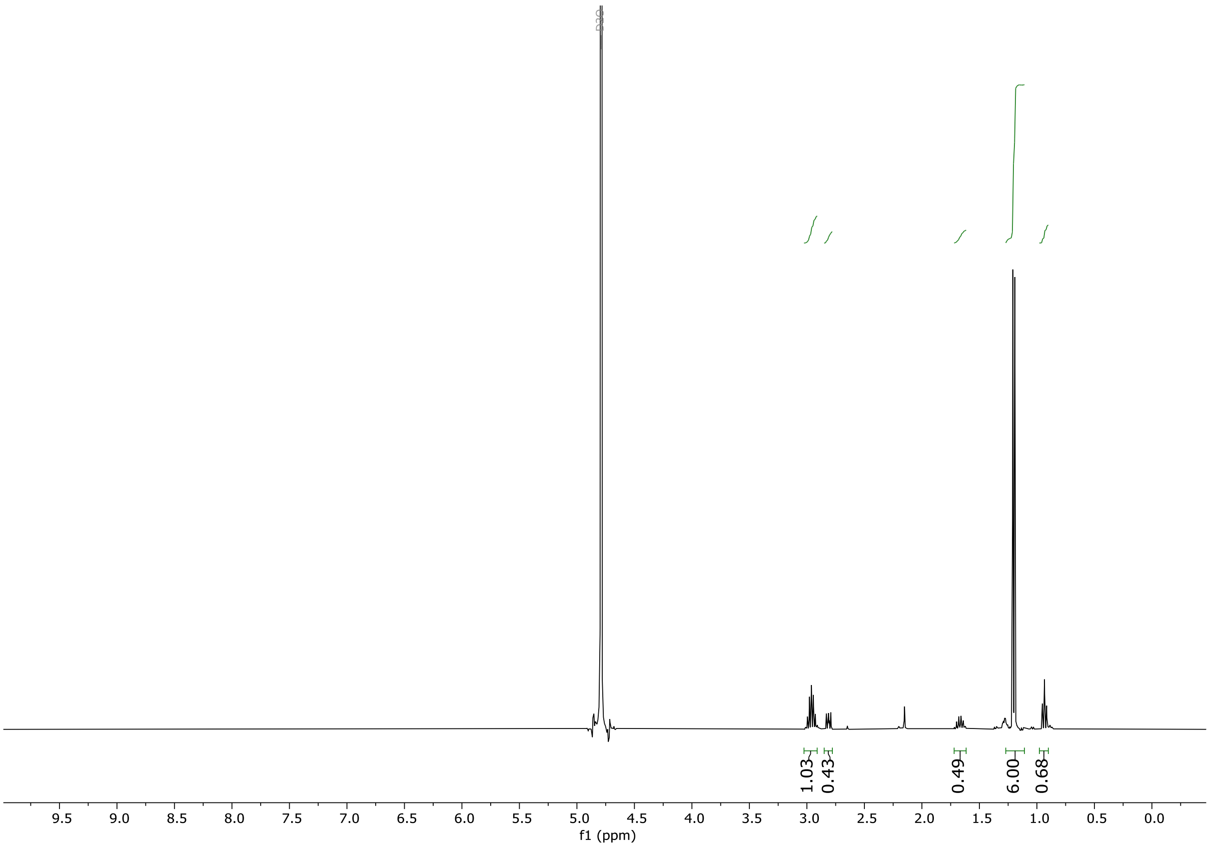

Supplementary Figure 8: ^{1}H NMR of the reaction between propane and SO_2, after acidification

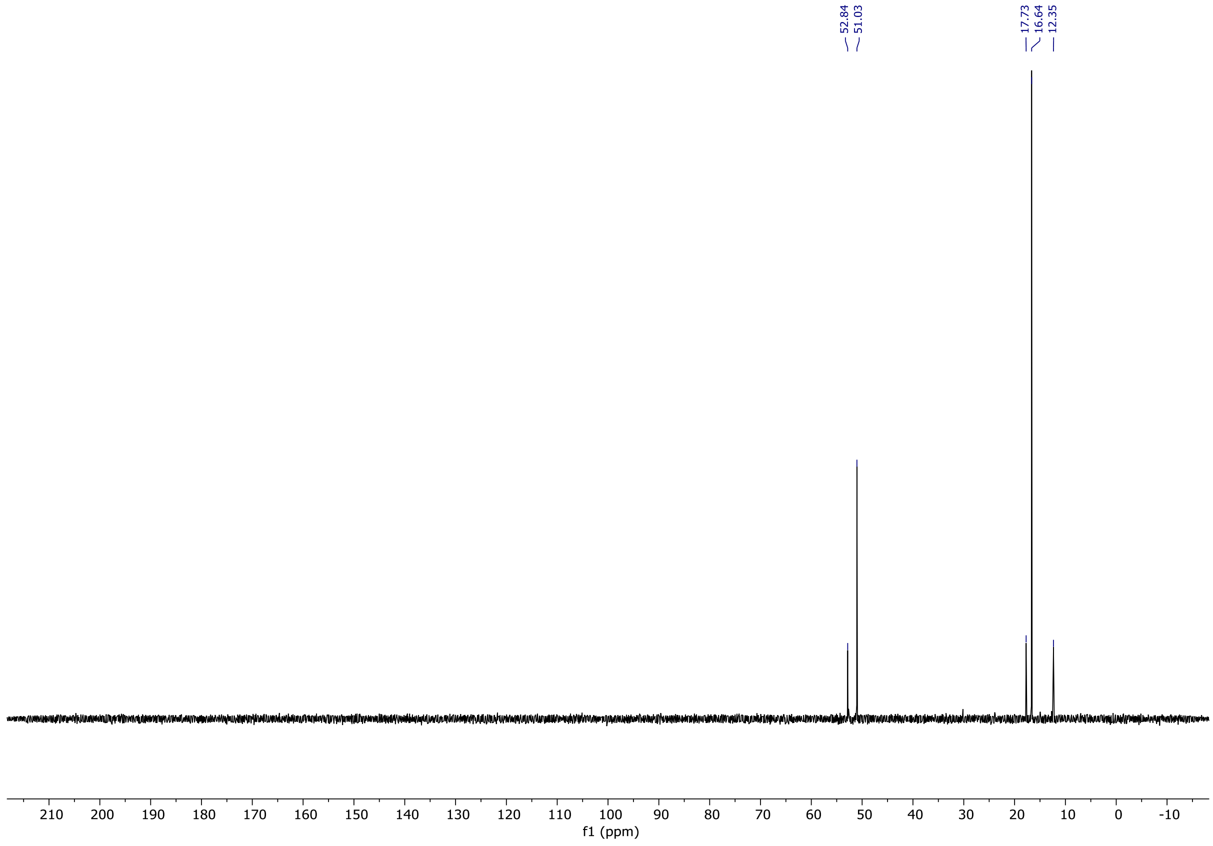

Supplementary Figure 9: ^{13}C NMR of the reaction between propane and SO_2, after acidification

7. Characterization Data

7.1 Starting Materials

3-(bromomethyl)-2-cyclopropyl-4-(4-fluorophenyl)quinoline (S1)

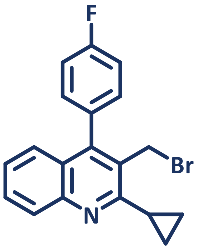

S1

In an oven-dried flask, phosphorus tribromide (0.8 mL, 8.5 mmol) is added slowly to a solution of (2-cyclopropyl-4-(4-fluorophenyl)quinolin-3-yl)methanol (1.25 g, 4.25 mmol) in DCM (30 mL) at 0°C. After stirring for 4 hours at 0°C, 150 mL of water was added. The mixture was brought to a pH of 8 by adding a saturated solution of sodium bicarbonate. The mixture was transferred to a separatory funnel and extracted with AcOEt (3x150 mL). The organic layer was dried over $MgSO_4$ and evaporated in vacuo, affording product **S1** (1.467g, 97%) as a white solid. The spectroscopic data are consistent with those reported previously.[9]

^{1}H NMR (300 MHz, $CDCl_3$) δ 7.99 (d, J = 8.4 Hz, 1H), 7.69 – 7.57 (m, 1H), 7.40 – 7.24 (m, 6H), 4.59 (s, 2H), 2.59 – 2.44 (m, 1H), 1.45 – 1.34 (m, 2H), 1.21 – 1.07 (m, 2H).

Bis(4-(methoxycarbonyl)phenyl)iodonium trifluoromethanesulfonate (S2)

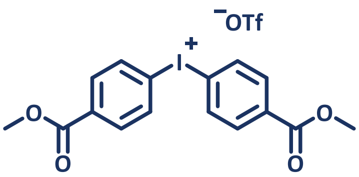

S2

In an oven-dried flask, mCPBA (1.23 g, 5.5 mmol, 77 w%) and methyl 4-iodobenzoate (1.310 g, 5 mmol) were dissolved in DCM (20 mL). Then, BF_3•OEt_2 (1.6 mL, 9.4 mmol) was added and stirred for 1 hour at room temperature. The mixture was cooled to 0°C and 4-methoxycarbonylphenylboronic acid (0.99 g, 5.5 mmol) was added and stirred for 15 minutes and slowly warmed up to room temperature where it was stirred for 1 hour. The mixture was re-cooled to 0°C and TfOH (0.45 mL, 5.1 mmol) was added dropwise and allowed to reach room temperature where it was stirred for 2 hours. The mixture was concentrated in vacuo and passed through a plug of silica with 5% MeOH:DCM eluent (200 mL). This was concentrated and Et_2O was added and stirred for 30 minutes. The precipitate was collected to afford S2 (1.137 g, 42%) as a white solid. The spectroscopic data are consistent with those reported previously.[10]

^{1}H NMR (300 MHz, DMSO–d_6) δ 8.45 – 8.34 (m, 4H), 8.09 – 7.99 (m, 4H), 3.86 (s, 6H).

methyl (2-bromoacetyl)-*D*-tryptophanate (S3)

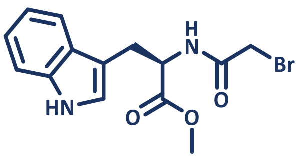

S3

In an oven-dried flask, Et_3N (1.67 mL, 12 mmol, 3 equiv.) was added to *D*-tryptophan methyl ester hydrochloride (1.01 g, 4 mmol, 1 equiv.) in DCM (20 mL). After stirring for 10 minutes, the flask was cooled to 0 °C and bromoacetyl bromide (0.44 mL, 5 mmol, 1.25 equiv.) was added dropwise. The flask was warmed to room temperature and the reaction mixture was stirred overnight. After reaction completion, the mixture was transferred to a separatory funnel and washed with water (20 mL) and brine (20 mL), then dried over $MgSO_4$ and evaporated in vacuo. The crude mixture was purified by flash column chromatography (100% *n*-pentane to *n*-pentane 50:50 AcOEt) to afford product **S3** (2.3:1 mixture of rotamers, 1.00 g, 74%) as a white solid.

Major rotamer

^{1}H NMR (400 MHz, DMSO–d_6) δ 10.89 (s, 1H), 8.73 (d, J = 7.5 Hz, 1H), 7.48 (d, J = 7.8 Hz, 1H), 7.34 (d, J = 8.0 Hz, 1H), 7.15 (s, 1H), 7.07 (t, J = 7.5 Hz, 1H), 6.99 (t, J = 7.4 Hz, 1H), 4.60 – 4.48 (m, 1H), 3.90 (s, 2H), 3.60 (s, 3H), 3.25 – 3.02 (m, 2H).

^{13}C NMR (101 MHz, DMSO–d_6) δ 171.8, 166.0, 136.1, 127.0, 123.7, 121.0, 118.5, 117.9, 111.4, 109.0, 53.4, 51.9, 29.0, 26.9.

Minor rotamer

^{1}H NMR (400 MHz, DMSO–d_6) δ 10.89 (s, 1H), 8.62 (d, J = 7.5 Hz, 1H), 7.48 (d, J = 7.8 Hz, 1H), 7.34 (d, J = 8.0 Hz, 1H), 7.15 (s, 1H), 7.07 (t, J = 7.5 Hz, 1H), 6.99 (t, J = 7.4 Hz, 1H), 4.60 – 4.48 (m, 1H), 4.09 (s, 2H), 3.60 (s, 3H), 3.25 – 3.02 (m, 2H).

^{13}C NMR (101 MHz, DMSO–d_6) δ 171.8, 165.9, 136.1, 127.0, 123.7, 121.0, 118.5, 117.9, 111.4, 109.0, 53.4, 52.0, 42.2, 26.9.

HRMS (ESI+) (m/z): $[M+H]^+$ calcd. for $C_{14}H_{16}BrN_2O_3$, 339.0339; found: 339.0342.

(*E*)-5-(6-((4-fluorobenzoyl)oxy)hex-1-en-1-yl)-5*H*-thianthren-5-ium tetrafluoroborate (S4)

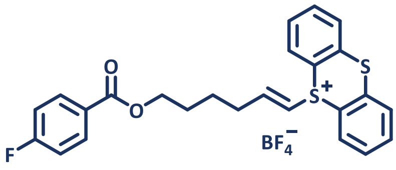

S4

Under ambient atmosphere, a 100 mL round-bottom flask equipped with a magnetic stir bar was charged with hex-5-en-1-yl 4-fluorobenzoate (1.11 g, 5.00 mmol, 1.00 equiv.), thianthrene S-oxide (1.20 g, 5.17 mmol, 1.03 equiv.), and CH_3CN (10 mL, c = 0.25 M). After cooling to 0 °C, trifluoroacetic anhydride (1.05 mL, 15 mmol, 3.0 equiv.) was added dropwise within 30 seconds, followed by dropwise addition of HOTf (260 µL, 5.9 mmol, 1.2 equiv.) within 10 seconds. After stirring the lilac mixture at 0 °C for 60 min followed by stirring at 25 °C for 30 min, the resulting purple mixture was concentrated under reduced pressure and subsequently diluted with CH_2Cl_2 (50 mL). The CH_2Cl_2 solution was poured onto a saturated aqueous $NaHCO_3$ solution (ca. 100 mL). The combined mixture was poured into a separatory funnel, and the layers were separated. The CH_2Cl_2 layer was collected, and the aqueous layer was further extracted with CH_2Cl_2 (2 × ca. 50 mL). The combined CH_2Cl_2 solution was washed with aqueous $NaBF_4$ solution (2 × ca. 100 mL, 10 % w/w). The CH_2Cl_2 layer was dried over Na_2SO_4, filtered, and the solvent was removed under reduced pressure. The residue was purified by flash column chromatography (100:1 CH_2Cl_2/*i*-PrOH to 15:1 CH_2Cl_2/*i*-PrOH) to afford product **S4** (*E*/*Z* = 5.3/1, 1.36 g, 52% yield) as white solid.

E-isomer

^{1}H NMR (400 MHz, $CDCl_3$) δ 8.26 (d, J = 7.9 Hz, 2H), 7.93 (dd, J = 8.5, 5.5 Hz, 2H), 7.76 (d, J = 7.9 Hz, 2H), 7.76 – 7.56 (m, 5H), 7.02 (t, J = 8.6 Hz, 2H), 6.53 (d, J = 14.8 Hz, 1H), 4.16 (t, J = 6.2 Hz, 2H), 2.27 (q, J = 7.3 Hz, 2H), 1.69 – 1.46 (m, 4H).

^{13}C NMR (101 MHz, $CDCl_3$) δ 165.8 (d, J = 253.7 Hz), 165.7, 156.4, 135.6, 134.5, 133.8, 132.2 (d, J = 9.4 Hz), 130.3 (d, J = 20.2 Hz), 126.49 (d, J = 2.9 Hz), 120.5, 115.7, 115.5, 109.9, 64.4, 33.0, 28.1, 24.0.

^{19}F NMR (282 MHz, $CDCl_3$) δ -105.66 – -105.77 (m), 150.54 – -150.58 (m), 150.60 – -150.65 (m).

Z-isomer

^{1}H NMR (400 MHz, $CDCl_3$) δ 8.35 (d, J = 8.0 Hz, 2H), 8.06 – 7.99 (m, 2H), 7.85 – 7.79 (m, 2H), 7.77 – 7.57 (m, 4H), 7.33 – 7.18 (m, 1H), 7.12 – 7.00 (m, 2H), 6.71 (d, J = 8.7 Hz, 1H), 4.31 (t, J = 6.3 Hz, 2H), 2.88 (q, J = 7.5 Hz, 2H), 1.94 – 1.79 (m, 4H).

^{13}C NMR (101 MHz, $CDCl_3$) δ 165.8 (d, J = 253.7 Hz), 165.7, 155.2, 135.7, 134.4, 133.4, 132.2 (d, J = 9.2 Hz), 130.3 (d, J = 20.2 Hz), 128.8, 127.8, 126.5 (d, J = 3.0 Hz), 121.2, 111.8, 64.6, 30.0, 28.2, 25.0.

^{19}F NMR (282 MHz, $CDCl_3$) δ -105.66 – -105.77 (m), 150.54 – -150.58 (m), 150.60 – -150.65 (m).

HRMS (ESI+) (m/z): [M-BF^4]+ calcd. for $C_{25}H_{22}FO_2S_2$ 437.1040; found: 437.1046.

7.2 Isobutane

((*tert*-butylsulfonyl)methyl)benzene (2a) and ((isobutylsulfonyl)methyl)benzene (2b)

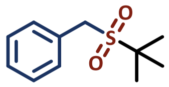

2a

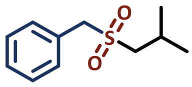

2b

Prepared according to the general procedure for isobutane, using aqueous SO_2 (6 wt%, 0.6 mL, 0.6 mmol, 3 equiv.), NaDT (14.65 mg, 0.006 mmol, 3 mol%), isobutane (70 mL, 3 mmol, 15 equiv.) and benzyl bromide (34.2 mg, 23.8 µL, 0.2 mmol, 1 equiv.). After all the outflow from the Signify Eagle reactor was collected, the reaction mixture was stirred for 1 hour at 60 °C. The crude mixture was purified by flash column chromatography (100% *n*-pentane to *n*-pentane 80:20 AcOEt) to afford product **2a** and **2b** (87:13 ratio determined by ^{1}H NMR analysis of the crude reaction mixture), (34.3 mg + 5.1 mg, 93%) as a white solid. The spectroscopic data are consistent with those reported previously.[11]

Regioisomer **2a**

^{1}H NMR (400 MHz, $CDCl_3$) δ 7.48 – 7.33 (m, 5H), 4.19 (s, 2H), 1.43 (s, 9H).

^{13}C NMR (101 MHz, $CDCl_3$) δ 131.3, 128.8, 127.3, 60.0, 52.9, 23.9.

HRMS (FD+) (m/z): $[M]^+$ calcd. for $C_{11}H_{16}O_2S$, 212.0866; found: 212.0907.

Regioisomer **2b**

^{1}H NMR (300 MHz, $CDCl_3$) δ 7.40 (s, 5H), 4.21 (s, 2H), 2.72 (d, J = 6.6 Hz, 2H), 2.40 – 2.25 (m, 1H), 1.08 (d, J = 6.6 Hz, 6H).

^{13}C NMR (75 MHz, $CDCl_3$) δ 130.7, 129.2, 129.1, 128.4, 60.9, 58.5, 23.6, 22.9.

HRMS (FD+) (m/z): $[M]^+$ calcd. for $C_{11}H_{16}O_2S$, 212.0866; found: 212.0903.

2-((*tert*-butylsulfonyl)methyl)pyridine (3a) and 2-((isobutylsulfonyl)methyl)pyridine (3b)

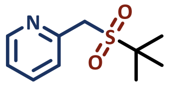

3a

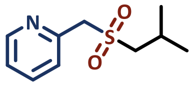

3b

Prepared according to the general procedure for isobutane, using aqueous SO_2 (6 wt%, 0.6 mL, 0.6 mmol, 3 equiv.), NaDT (14.65 mg, 0.006 mmol, 3 mol%), isobutane (70 mL, 3 mmol, 15 equiv.) and 2-(bromomethyl)pyridine hydrobromide (50.6 mg, 0.2 mmol, 1 equiv.). After all the outflow from the Signify Eagle reactor was collected, the reaction mixture was stirred for 1 hour at 60 °C. The crude mixture was purified by flash column chromatography (100% *n*-pentane to *n*-pentane 40:60 AcOEt) to afford product **3a** and **3b** (86:14 ratio determined by ^{1}H NMR analysis of the crude reaction mixture), (33.3 mg, 78%) as a white solid.

Regioisomer **3a**

^{1}H NMR (300 MHz, $CDCl_3$) δ 8.59 (ddd, J = 4.9, 1.8, 0.9 Hz, 1H), 7.72 (td, J = 7.6, 1.9 Hz, 1H), 7.60 (dt, J = 7.6, 1.1 Hz, 1H), 7.28 (ddd, J = 7.6, 4.9, 1.3 Hz, 1H), 4.46 (s, 2H), 1.39 (s, 9H).

^{13}C NMR (75 MHz, $CDCl_3$) δ 149.7, 149.3, 136.9, 126.2, 123.6, 60.9, 56.3, 23.7.

Regioisomer **3b**

^{1}H NMR (300 MHz, $CDCl_3$) δ 8.60 (ddd, J = 4.9, 1.9, 1.0 Hz, 1H), 7.81 – 7.68 (m, 1H), 7.51 (dt, J = 7.8, 1.1 Hz, 1H), 7.31 (ddd, J = 7.6, 4.9, 1.2 Hz, 1H), 4.39 (s, 2H), 2.90 (d, J = 6.6 Hz, 2H), 2.36 (m, 1H), 1.09 (d, J = 6.6 Hz, 6H).

^{13}C NMR (75 MHz, $CDCl_3$) δ 149.9, 149.8, 137.3, 126.1, 123.7, 62.8, 59.4, 23.6, 22.8.

HRMS (ESI+) (m/z): $[M+H]^+$ calcd. for $C_{10}H_{16}NO_2S$, 214.0896; found: 214.0901.

(*E*)-1-(*tert*-butylsulfonyl)-3,7-dimethylocta-2,6-diene (4a) and (*E*)-1-(isobutylsulfonyl)-3,7-dimethylocta-2,6-diene (4b))

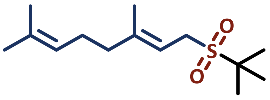

4a

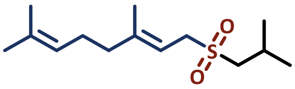

4b

Prepared according to the general procedure for isobutane, using aqueous SO_2 (6 wt%, 0.6 mL, 0.6 mmol, 3 equiv.), NaDT (14.65 mg, 0.006 mmol, 3 mol%), isobutane (70 mL, 3 mmol, 15 equiv.) and geranyl bromide (43.4 mg, 39.7 µL, 0.2 mmol, 1 equiv.). After all the outflow from the Signify Eagle reactor was collected, the reaction mixture was stirred at room temperature overnight. The crude mixture was purified by flash column chromatography (100% *n*-pentane to *n*-pentane 80:20 AcOEt) to afford product **4a** and **4b** as an inseparable mixture of regioisomers (89:11 ratio determined by ^{1}H NMR analysis of the crude reaction mixture), (25.3 mg, 49%) as a slightly yellow oil.

Regioisomer **4a**

^{1}H NMR (400 MHz, $CDCl_3$) δ 5.33 (t, J = 7.6 Hz, 1H), 5.07 (t, J = 7.5 Hz, 1H), 3.70 (d, J = 7.7 Hz, 2H), 2.15 – 2.08 (m, 4H), 1.72 (s, 3H), 1.66 (s, 3H), 1.58 (s, 3H), 1.41 (s, 9H).

^{13}C NMR (101 MHz, $CDCl_3$) δ 145.4, 132.1, 123.6, 109.9, 59.7, 47.1, 39.9, 26.3, 25.8, 23.8, 17.8, 17.0.

Regioisomer **4b**

1H NMR (400 MHz, $CDCl_3$) δ 5.44 – 5.36 (m, 1H), 5.11 – 5.01 (m, 1H), 3.70 (d, J = 7.7 Hz, 2H), 2.77 (d, J = 6.5 Hz, 1H), 2.39 – 2.25 (m, 1H), 2.13 – 2.08 (m, 4H), 1.72 (s, 3H), 1.66 (s, 3H), 1.58 (s, 3H), 1.10 (d, J = 6.7 Hz, 6H).

Overlapping of peaks and low concentration avoids the unambiguous ^{13}C NMR characterization of the minor regioisomer **4b**.

HRMS (FD+) (m/z): $[M]^+$ calcd. for $C_{14}H_{26}O_2S$, 258.1648; found: 258.1650.

methyl (2-(*tert*-butylsulfonyl)acetyl)-*D*-tryptophanate (5a) and methyl (2-(isobutylsulfonyl)acetyl)-*D*-tryptophanate (5b)

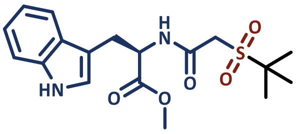

5a

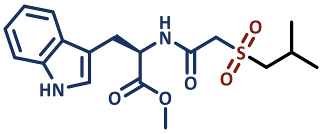

5b

Prepared according to the general procedure for isobutane, using aqueous SO_2 (6 wt%, 0.6 mL, 0.6 mmol, 3 equiv.), NaDT (14.65 mg, 0.006 mmol, 3 mol%), isobutane (70 mL, 3 mmol, 15 equiv.) and S3 (68 mg, 0.2 mmol, 1 equiv.). After all the outflow from the Signify Eagle reactor was collected, the reaction mixture was stirred for 1 hour at 60 °C. The crude mixture was purified by flash column chromatography (100% *n*-pentane to *n*-pentane 50:50 AcOEt) to afford product **5a** as a single regioisomer (47.2 mg, 62%, 90:10 ratio determined by ^{1}H NMR analysis of the crude reaction mixture) as a yellow solid.

Regioisomer **5a**

^{1}H NMR (400 MHz, $CDCl_3$) δ 8.40 (brs, 1H), 7.52 (d, J = 7.9 Hz, 1H), 7.39 (d, J = 7.5 Hz, 1H), 7.32 (dd, J = 7.9, 1.0 Hz, 1H), 7.20 – 7.05 (m, 3H), 4.90 (dt, J = 7.6, 5.7 Hz, 1H), 3.77 (s, 2H), 3.65 (s, 3H), 3.32 (m, 2H), 1.33 (s, 9H).

^{13}C NMR (101 MHz, $CDCl_3$) δ 171.7, 161.2, 136.3, 127.3, 123.8, 122.2, 119.6, 118.5, 111.4, 109.2, 61.5, 53.2, 52.9, 52.5, 27.6, 23.4.

HRMS (ESI+) (m/z): $[M+H]^+$ calcd. for $C_{18}H_{25}N_2O_5S$, 381.1479; found: 381.1476.

3-((*tert*-butylsulfonyl)methyl)-2-cyclopropyl-4-(4-fluorophenyl)quinoline (6a) and 2-cyclopropyl-4-(4-fluorophenyl)-3-((isobutylsulfonyl)methyl)quinoline (6b)

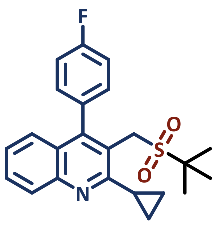

6a

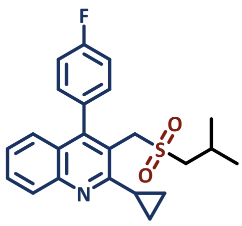

6b

Prepared according to the general procedure for isobutane, using aqueous SO_2 (6 wt%, 0.6 mL, 0.6 mmol, 3 equiv.), NaDT (14.65 mg, 0.006 mmol, 3 mol%), isobutane (70 mL, 3 mmol, 15 equiv.) and S1 (71.2 mg, 0.2 mmol, 1 equiv.). After all the outflow from the Signify Eagle reactor was collected, the reaction mixture was stirred for 1 hour at 60 °C. The crude mixture was purified by flash column chromatography (100% *n*-pentane to *n*-pentane 75:25 AcOEt) to afford product **6a** and **6b** as an inseparable mixture of regioisomers (82:18 ratio determined by ^{1}H NMR analysis of the crude reaction mixture), (58 mg, 73%) as a white solid.

Regioisomer **6a**

^{1}H NMR (300 MHz, $CDCl_3$) δ 8.01 (d, J = 8.4 Hz, 1H), 7.66 (ddd, J = 8.4, 6.8, 1.4 Hz, 1H), 7.45 – 7.18 (m, 6H), 4.56 (s, 2H), 2.82 (ddd, J = 13.0, 8.1, 4.9 Hz, 1H), 1.43 – 1.21 (m, 11H), 1.13 (dd, J = 8.0, 3.3 Hz, 2H).

^{13}C NMR (75 MHz, $CDCl_3$) δ 162.7 (d, J = 247.9 Hz), 162.4, 150.0, 147.5, 132.3 (d, J = 3.7 Hz), 132.2 (d, J = 6.9 Hz), 129.8, 129.1, 126.7, 126.4, 125.8, 118.2, 115.4 (d, J = 21.5 Hz), 60.1, 45.9, 23.3, 15.6, 10.3.

^{19}F NMR (282 MHz, $CDCl_3$) δ -113.44.

Regioisomer **6b**

^{1}H NMR (300 MHz, $CDCl_3$) δ 8.01 (d, J = 8.4 Hz, 1H), 7.66 (ddd, J = 8.4, 6.8, 1.4 Hz, 1H), 7.48 – 7.14 (m, 6H), 4.61 (s, 2H), 2.88 – 2.76 (m, 1H), 2.71 (d, J = 6.6 Hz, 2H), 2.27 – 2.14 (m, 1H), 1.39 – 1.31 (m, 2H), 1.13 (dd, J = 8.0, 3.3 Hz, 2H), 1.04 (d, J = 6.7 Hz, 6H).

Overlapping of peaks and low concentration avoids the unambiguous ^{13}C NMR characterization of the minor regioisomer **6b**.

^{19}F NMR (282 MHz, $CDCl_3$) δ -112.83.

HRMS (ESI+) (m/z): $[M+H]^+$ calcd. for $C_{23}H_{25}FNO_2S$, 398.1585; found: 398.1584.

7.3 *n*-Butane

((*sec*-butylsulfonyl)methyl)benzene (7a) and ((butylsulfonyl)methyl)benzene (7b)

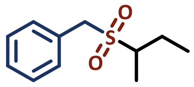

7a

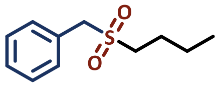

7b

Prepared according to the general procedure for butane, using aqueous SO_2 (6 wt%, 0.6 mL, 0.6 mmol, 3 equiv.), NaDT (14.65 mg, 0.006 mmol, 3 mol%), butane (70 mL, 3 mmol, 15 equiv.) and benzyl bromide (34.2 mg, 23.8 µL, 0.2 mmol, 1 equiv.). After all the outflow from the Signify Eagle reactor was collected, the reaction mixture was stirred for 1 hour at 60 °C. The crude mixture was purified by flash column chromatography (100% *n*-pentane to *n*-pentane 80:20 AcOEt) to afford product **7a** and **7b** as an inseparable mixture of regioisomers (82:18 ratio determined by ^{1}H NMR analysis of the crude reaction mixture), (36 mg, 85%) as a clear oil.

Regioisomer **7a**

^{1}H NMR (400 MHz, $CDCl_3$) δ 7.44 – 7.33 (m, 5H), 4.20 (s, 2H), 2.79 – 2.68 (m, 1H), 2.03 (m, 1H), 1.56 (m, 1H), 1.35 (d, J = 6.9 Hz, 3H), 0.99 (t, J = 7.5 Hz, 3H).

^{13}C NMR (101 MHz, $CDCl_3$) δ 130.6, 129.0, 128.9, 127.9, 57.1, 56.4, 22.1, 12.2, 11.0.

Regioisomer **7b**

^{1}H NMR (400 MHz, $CDCl_3$) δ 7.44 – 7.33 (m, 5H), 4.20 (s, 2H), 2.81 (m, 2H), 1.77 (m, 2H), 1.40 (m, 2H), 0.90 (t, J = 7.5 Hz, 3H).

^{13}C NMR (101 MHz, $CDCl_3$) δ 130.5, 129.1, 129.0, 128.2, 59.4, 50.8, 23.7, 21.7, 13.5.

HRMS (EI+) (m/z): $[M]^+$ calcd. for $C_{11}H_{16}O_2S$, 212.0866; found: 212.0867.

2-((*sec*-butylsulfonyl)methyl)pyridine (8a) and 2-((butylsulfonyl)methyl)pyridine (8b)

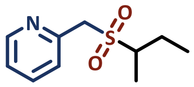

8a

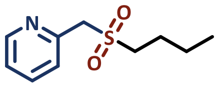

8b

Prepared according to the general procedure for butane, using aqueous SO_2 (6 wt%, 0.6 mL, 0.6 mmol, 3 equiv.), NaDT (14.65 mg, 0.006 mmol, 3 mol%), butane (70 mL, 3 mmol, 15 equiv.) and 2-(bromomethyl)pyridine hydrobromide (50.6 mg, 0.2 mmol, 1 equiv.). After all the outflow from the Signify Eagle reactor was collected, the reaction mixture was stirred for 1 hour at 60 °C. The crude mixture was purified by flash column chromatography (100% *n*-pentane to *n*-pentane 40:60 AcOEt) to afford product **8a** and **8b** as an inseparable mixture of regioisomers (83:17 ratio determined by ^{1}H NMR analysis of the crude reaction mixture), (38.4 mg, 90%) as a clear oil.

Regioisomer **8a**

^{1}H NMR (400 MHz, $CDCl_3$) δ 8.55 (d, J = 4.3, 1H), 7.70 (m, 1H), 7.53 – 7.44 (m, 1H), 7.32 – 7.22 (m, 1H), 4.46 – 4.33 (m, 2H), 2.86 (m, 1H), 2.06 (m, 1H), 1.63 – 1.47 (m, 1H), 1.36 (d, J = 7.0, 3H), 0.97 (t, J = 7.5, 3H).

^{13}C NMR (101 MHz, $CDCl_3$) δ 149.8, 149.6, 137.1, 126.0, 123.5, 58.7, 57.8, 21.8, 12.1, 11.0.

Regioisomer **8b**

^{1}H NMR (400 MHz, $CDCl_3$) δ 8.55 (d, J = 4.3, 1H), 7.70 (m, 1H), 7.53 – 7.44 (m, 1H), 7.32 – 7.22 (m, 1H), 4.37 (s, 2H), 2.99 – 2.91 (m, 2H), 1.86 – 1.73 (m, 2H), 1.46 – 1.38 (m, 2H), 0.89 (t, J = 7.4 Hz, 3H).

^{13}C NMR (101 MHz, $CDCl_3$) δ 149.8, 149.6, 137.2, 126.0, 123.6, 61.3, 51.7, 23.7, 21.7, 13.5.

HRMS (EI+) (m/z): $[M+H]^+$ calcd. for $C_{10}H_{16}NO_2S$, 214.0896; found: 214.0901.

(*E*)-1-(*sec*-butylsulfonyl)-3,7-dimethylocta-2,6-diene (9a) and (*E*)-1-(butylsulfonyl)-3,7-dimethylocta-2,6-diene (9b)

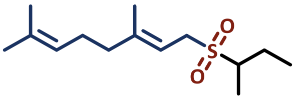

9a

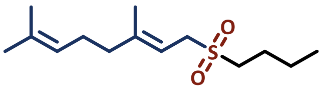

9b

Prepared according to the general procedure for butane, using aqueous SO_2 (6 wt%, 0.6 mL, 0.6 mmol, 3 equiv.), NaDT (14.65 mg, 0.006 mmol, 3 mol%), butane (70 mL, 3 mmol, 15 equiv.) and geranyl bromide (43.4 mg, 39.7 µL, 0.2 mmol, 1 equiv.). After all the outflow from the Signify Eagle reactor was collected, the reaction mixture was stirred at room temperature overnight. The crude mixture was purified by flash column chromatography (100% *n*-pentane to *n*-pentane 80:20 AcOEt) to afford product **9a** and **9b** as an inseparable mixture of regioisomers (87:13 ratio determined by ^1H NMR analysis of the crude reaction mixture), (26.8 mg, 52%) as a slightly yellow oil.

Regioisomer **9a**

^1H NMR (400 MHz, $CDCl_3$) δ 5.34 – 5.23 (m, 1H), 5.08 – 5.00 (m, 1H), 3.69 (d, J = 7.7 Hz, 2H), 2.94 – 2.81 (m, 1H), 2.14 – 2.08 (m, 4H), 2.07 – 1.97 (m, 1H), 1.72 (s, 3H), 1.66 (s, 3H), 1.59 (s, 3H), 1.57 – 1.50 (m, 1H), 1.34 (d, J = 6.9 Hz, 3H), 1.03 (t, J = 7.5 Hz, 3H).

^{13}C NMR (101 MHz, $CDCl_3$) δ 145.5, 132.2, 123.5, 110.4, 57.3, 50.4, 39.8, 26.2, 25.8, 22.1, 17.8, 16.9, 12.2, 11.2.

Regioisomer **9b**

^1H NMR (400 MHz, $CDCl_3$) δ 5.34 – 5.23 (m, 1H), 5.08 – 5.00 (m, 1H), 3.69 (d, J = 7.7 Hz, 2H), 2.14 – 2.08 (m, 4H), 1.83 – 1.73 (m, 2H), 1.72 (s, 3H), 1.66 (s, 3H), 1.59 (s, 3H), 1.48 – 1.40 (m, 2H), 0.94 (t, J = 7.3 Hz, 3H).

^{13}C NMR (101 MHz, $CDCl_3$) δ 145.8, 132.3, 123.4, 110.7, 53.0, 51.0, 39.8, 26.2, 25.8, 23.8, 21.9, 17.8, 16.8, 13.6.

HRMS (EI+) (m/z): $[M]^+$ calcd. for $C_{14}H_{26}O_2S$, 258.1648; found: 258.1653.

methyl (2-(*sec*-butylsulfonyl)acetyl)-*D*-tryptophanate (10a) and methyl (2-(butylsulfonyl)acetyl)-*D*-tryptophanate (10b)

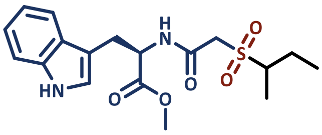

10a

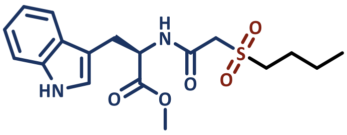

10b

Prepared according to the general procedure for butane, using aqueous SO_2 (6 wt%, 0.6 mL, 0.6 mmol, 3 equiv.), NaDT (14.65 mg, 0.006 mmol, 3 mol%), butane (70 mL, 3 mmol, 15 equiv.) and S3 (68 mg, 0.2 mmol, 1 equiv.). After all the outflow from the Signify Eagle reactor was collected, the reaction mixture was stirred for 1 hour at 60 °C. The crude mixture was purified by flash column chromatography (100% *n*-pentane to *n*-pentane 50:50 AcOEt) to afford product **10a** and **10b** as an inseparable mixture of isomers (80 [dr 1:1]:20 ratio determined by ^1H NMR analysis of the crude reaction mixture), (48 mg, 63%) as a clear oil.

Regioisomer **10a** and Diastereoisomer **10a’**

^{1}H NMR (400 MHz, $CDCl_3$) δ 8.21 (s, 1H), 7.53 (d, J = 7.8 Hz, 1H), 7.38 – 7.32 (m, 1H), 7.22 – 7.05 (m, 4H), 4.94 – 4.84 (m, 1H), 3.85 – 3.71 (m, 2H), 3.71 – 3.67 (m, 3H), 3.42 – 3.25 (m, 2H), 3.14 – 2.91 (m, 1H), 2.03 – 1.89 (m, 1H), 1.56 – 1.43 (m, 1H), 1.35 – 1.23 (m, 3H), 1.02 – 0.89 (m, 3H).

^{13}C NMR (101 MHz, $CDCl_3$) δ 171.7 (2C), 161.2 (2C), 136.3, 136.3, 127.3, 127.3, 123.6, 123.6, 122.4, 122.4, 119.8, 119.8, 118.5 (2C), 111.4 (2C), 109.4, 109.3, 59.5, 59.4, 56.2, 56.1, 53.2, 53.2, 52.7 (2C), 27.4 (2C), 22.0, 21.8, 12.2, 11.9, 10.9, 10.9.

Regioisomer **10b**

^{1}H NMR (400 MHz, $CDCl_3$) δ 8.21 (s, 1H), 7.53 (d, J = 7.8 Hz, 1H), 7.38 – 7.32 (m, 1H), 7.22 – 7.05 (m, 4H), 4.94 – 4.84 (m, 1H), 3.77 (s, 2H), 3.69 (s, 3H), 3.42 – 3.25 (m, 2H), 3.14 – 2.91 (m, 2H), 1.81 – 1.68 (m, 2H), 1.43 – 1.34 (m, 2H), 0.93 – 0.89 (m, 3H).

^{13}C NMR (101 MHz, $CDCl_3$) δ 171.7, 161.3, 136.3, 127.3, 123.6, 122.4, 119.8, 118.5, 111.4, 109.2, 58.6, 53.2, 53.0, 52.7, 27.4, 23.8, 21.6, 13.5.

HRMS (EI+) (m/z): $[M]^+$ calcd. for $C_{18}H_{24}N_2O_5S$, 380.1400; found: 380.1410.

3-((*sec*-butylsulfonyl)methyl)-2-cyclopropyl-4-(4-fluorophenyl)quinoline (11a) and 3-((butylsulfonyl)methyl)-2-cyclopropyl-4-(4-fluorophenyl)quinoline (11b)

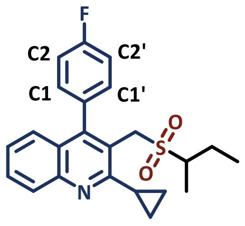

11a

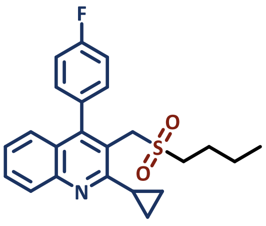

11b

Prepared according to the general procedure for butane, using aqueous SO_2 (6 wt%, 0.6 mL, 0.6 mmol, 3 equiv.), NaDT (14.65 mg, 0.006 mmol, 3 mol%), butane (70 mL, 3 mmol, 15 equiv.) and S1 (71.2 mg, 0.2 mmol, 1 equiv.). After all the outflow from the Signify Eagle reactor was collected, the reaction mixture was stirred for 1 hour at 60 °C. The crude mixture was purified by flash column chromatography (100% *n*-pentane to *n*-pentane 75:25 AcOEt) to afford product **11a** and **11b** as an inseparable mixture of regioisomers (72:24 ratio determined by ^{1}H NMR analysis of the crude reaction mixture), (43.7 mg, 55%) as a white solid.

Regioisomer **11a**

^{1}H NMR (400 MHz, $CDCl_3$) δ 7.89 (m, 1H), 7.59 – 7.50 (m, 1H), 7.32 – 7.19 (m, 3H), 7.19 – 7.09 (m, 3H), 4.43 (s, 2H), 2.66 – 2.54 (m, 2H), 1.78 (dddd, J = 15.1, 11.2, 6.9, 3.6 Hz, 1H), 1.37 – 1.20 (m, 3H), 1.09 (d, J = 6.9 Hz, 3H), 1.03 (dd, J = 8.2, 4.2 Hz, 2H), 0.87 (t, J = 7.5 Hz, 3H).

^{13}C NMR (101 MHz, $CDCl_3$) δ 162.7 (d, J = 248.0 Hz), 162.2, 149.3, 147.5, 132.3 (d, J = 3.8 Hz), 132.0 (d, J = 7.7 Hz, C1 and C1'), 129.8, 129.1, 126.5, 126.4, 125.8, 118.5, 115.5 (d, J = 21.4 Hz), 115.5 (d, J = 21.4 Hz) 61.1, 49.8, 22.3, 15.7, 12.5, 11.2, 10.3.

Due to the hindered rotation of the biaryl motif, C1 and C1' are not equivalent. In addition, C2 and C2' are not equivalent.

^{19}F NMR (282 MHz, $CDCl_3$) δ -113.12.

Regioisomer **11b**

^{1}H NMR (400 MHz, $CDCl_3$) δ 7.89 (m, 1H), 7.59 – 7.50 (m, 1H), 7.32 – 7.19 (m, 3H), 7.19 – 7.09 (m, 3H), 4.51 (s, 2H), 2.74 – 2.68 (m, 2H), 2.66 – 2.54 (m, 1H), 1.53 – 1.42 (m, 2H), 1.37 – 1.20 (m, 4H), 1.03 (dd, J = 8.2, 4.2 Hz, 2H), 0.80 (t, J = 7.3 Hz, 3H).

^{13}C NMR (101 MHz, $CDCl_3$) δ 162.8 (d, J = 248.0 Hz), 162.0, 149.0, 147.6, 132.2 (d, J = 3.6 Hz), 132.0 (d, J = 7.7 Hz), 129.9, 129.1, 126.5, 126.4, 125.9, 118.9, 115.7 (d, J = 20.7 Hz), 54.4, 53.6, 23.9, 21.7, 15.8, 13.5, 10.5.

^{19}F NMR (282 MHz, $CDCl_3$) δ -112.78.

HRMS (ESI+) (m/z): $[M+H]^+$ calcd. for $C_{23}H_{25}FNO_2S$, 398.1585; found: 398.1589.

7.4 Propane

((isopropylsulfonyl)methyl)benzene (1a) and ((propylsulfonyl)methyl)benzene (1b)

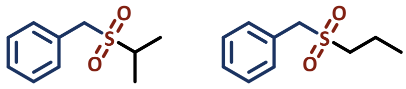

1a **1b**

Prepared according to the general procedure for propane, using aqueous SO_2 (6 wt%, 0.6 mL, 0.6 mmol, 3 equiv.), NaDT (14.65 mg, 0.006 mmol, 3 mol%), propane (73.5 mL, 3 mmol, 15 equiv.) and benzyl bromide (34.2 mg, 23.8 µL, 0.2 mmol, 1 equiv.). After all the outflow from the Signify Eagle reactor was collected, the reaction mixture was stirred for 1 hour at 60 °C. The crude mixture was purified by flash column chromatography (100% *n*-pentane to *n*-pentane 80:20 AcOEt) to afford product **1a** and **1b** (81:19 ratio determined by ^{1}H NMR analysis of the crude reaction mixture), (37.7 mg, 95%) as a clear oil.

Regioisomer **1a**

^{1}H NMR (400 MHz, $CDCl_3$) δ 7.46 – 7.35 (m, 5H), 4.22 (s, 2H), 3.00 (hept, J = 6.9 Hz, 1H), 1.37 (d, J = 6.9 Hz, 6H).

^{13}C NMR (101 MHz, $CDCl_3$) δ 130.7, 129.1, 129.0, 128.0, 56.3, 51.1, 15.4.

Regioisomer **1b**

1H NMR (300 MHz, $CDCl_3$) δ 7.47 – 7.32 (m, 5H), 4.21 (s, 2H), 2.85 – 2.73 (m, 2H), 1.93 – 1.74 (m, 2H), 1.02 (t, J = 7.4 Hz, 3H).

^{13}C NMR (101 MHz, $CDCl_3$) δ 130.6, 129.1, 128.2, 59.6, 52.8, 15.6, 13.2.

HRMS (EI+) (m/z): $[M]^+$ calcd. for $C_{10}H_{14}O_2S$, 198.0709; found: 198.0715.

2-((isopropylsulfonyl)methyl)pyridine (12a) and 2-((propylsulfonyl)methyl)pyridine (12b)

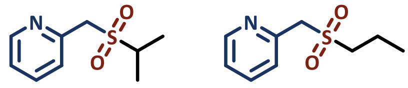

12a **12b**

Prepared according to the general procedure for propane, using aqueous SO_2 (6 wt%, 0.6 mL, 0.6 mmol, 3 equiv.), NaDT (14.65 mg, 0.006 mmol, 3 mol%), propane (73.5 mL, 3 mmol, 15 equiv.) and 2-(bromomethyl)pyridine hydrobromide (50.6 mg, 0.2 mmol, 1 equiv.). After all the outflow from the Signify Eagle reactor was collected, the reaction mixture was stirred for 1 hour at 60 °C. The crude mixture was purified by flash

column chromatography (100% *n*-pentane to *n*-pentane 40:60 AcOEt) to afford product **12a** and **12b** as an inseparable mixture of regioisomers (80:20 ratio determined by ^{1}H NMR analysis of the crude reaction mixture), (36 mg, 90%) as a slightly yellow oil.

Regioisomer **12a**

^{1}H NMR (400 MHz, $CDCl_3$) δ 8.58 (m, 1H), 7.79 – 7.67 (m, 1H), 7.51 (m, 1H), 7.34 – 7.27 (m, 1H), 4.41 (s, 2H), 3.14 (hept, J = 6.9 Hz, 1H), 1.39 (d, J = 6.9 Hz, 6H).

^{13}C NMR (101 MHz, $CDCl_3$) δ 149.9, 149.7, 137.2, 126.1, 123.6, 58.5, 51.9, 15.2.

Regioisomer **12b**

^{1}H NMR (400 MHz, $CDCl_3$) δ 8.58 (m, 1H), 7.79 – 7.67 (m, 1H), 7.51 (m, 1H), 7.34 – 7.27 (m, 1H), 4.38 (s, 2H), 2.99 – 2.91 (m, 2H), 1.95 – 1.81 (m, 2H), 1.03 (t, J = 7.4 Hz, 3H).

^{13}C NMR (101 MHz, $CDCl_3$) δ 149.9, 149.7, 137.3, 126.1, 123.7, 61.5, 53.7, 15.7, 13.2.

HRMS (EI+) (m/z): $[M]^+$ calcd. for $C_9H_{13}NO_2S$, 199.0662; found: 199.0670.

(*E*)-1-(isopropylsulfonyl)-3,7-dimethylocta-2,6-diene (13a) and (*E*)-3,7-dimethyl-1-(propylsulfonyl)octa-2,6-diene (13b)

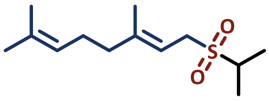

13a

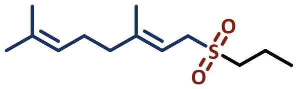

13b

Prepared according to the general procedure for propane, using aqueous SO_2 (6 wt%, 0.6 mL, 0.6 mmol, 3 equiv.), NaDT (14.65 mg, 0.006 mmol, 3 mol%), propane (70 mL, 3 mmol, 15 equiv.) and geranyl bromide (43.4 mg, 39.7 µL, 0.2 mmol, 1 equiv.). After all the outflow from the Signify Eagle reactor was collected, the reaction mixture was stirred at room temperature overnight. The crude mixture was purified by flash column chromatography (100% *n*-pentane to *n*-pentane 80:20 AcOEt) to afford product **13a** and **13b** as an inseparable mixture of regioisomers (83:17 ratio determined by 1H NMR analysis of the crude reaction mixture), (33.3 mg, 68%) as a slightly yellow oil.

Regioisomer **13a**

^{1}H NMR (400 MHz, $CDCl_3$) δ 5.28 (t, J = 7.8, 1H), 5.07 – 5.00 (m, 1H), 3.70 (d, J = 7.8 Hz, 2H), 3.13 (hept, J = 6.9 Hz, 1H), 2.15 – 2.09 (m, 4H), 1.73 (s, 3H), 1.67 (s, 3H), 1.60 (s, 3H), 1.36 (d, J = 6.9 Hz, 6H).

^{13}C NMR (101 MHz, $CDCl_3$) δ 145.6, 132.2, 123.5, 110.4, 51.2, 50.2, 39.8, 26.2, 25.8, 17.8, 16.9, 15.2.

Regioisomer **13b**

^{1}H NMR (400 MHz, $CDCl_3$) δ 5.33 – 5.25 (m, 1H), 5.07 – 5.00 (m, 1H), 3.68 (d, J = 7.8, 2H), 2.93 – 2.80 (m, 2H), 2.15 – 2.09 (m, 4H), 1.92 – 1.78 (m, 2H), 1.73 (s, 3H), 1.67 (s, 3H), 1.60 (s, 3H), 1.07 (t, J = 7.4 Hz, 3H).

^{13}C NMR (101 MHz, $CDCl_3$) δ 145.8, 132.3, 123.4, 110.7, 53.1, 52.9, 39.8, 26.2, 25.8, 17.8, 16.8, 15.7, 13.3.

HRMS (EI+) (m/z): $[M]^+$ calcd. for $C_{13}H_{24}O_2S$, 244.1492; found: 244.1493.

methyl (2-(isopropylsulfonyl)acetyl)-*D*-tryptophanate (14a) and methyl (2-(propylsulfonyl)acetyl)-*D*-tryptophanate (14b)

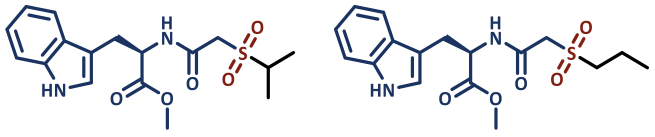

14a **14b**

Prepared according to the general procedure for propane, using aqueous SO_2 (6 wt%, 0.6 mL, 0.6 mmol, 3 equiv.), NaDT (14.65 mg, 0.006 mmol, 3 mol%), propane (73.5 mL, 3 mmol, 15 equiv.) and S3 (68 mg, 0.2 mmol, 1 equiv.). After all the outflow from the Signify Eagle reactor was collected, the reaction mixture was stirred for 1 hour at 60 °C. The crude mixture was purified by flash column chromatography (100% *n*-pentane to *n*-pentane 50:50 AcOEt) to afford product **14a** and **14b** as an inseparable mixture of regioisomers (82:18 ratio determined by ^{1}H NMR analysis of the crude reaction mixture), (57.3 mg, 78%) as a slightly yellow oil.

Regioisomer **14a**

^{1}H NMR (400 MHz, $CDCl_3$) δ 8.38 (s, 1H), 7.51 (d, J = 7.8 Hz, 1H), 7.32 (d, J = 8.0 Hz, 1H), 7.22 – 7.13 (m, 2H), 7.13 – 7.06 (m, 2H), 4.90 (q, J = 5.8 Hz, 1H), 3.79 – 3.69 (m, 2H), 3.67 (s, 3H), 3.32 (d, J = 5.7 Hz, 2H), 3.29 – 3.19 (m, 1H), 1.29 (t, J = 7.5 Hz, 6H).

^{13}C NMR (101 MHz, $CDCl_3$) δ 171.8, 161.3, 136.2, 127.3, 123.8, 122.2, 119.7, 118.4, 111.5, 109.1, 55.6, 53.5, 53.1, 52.6, 27.3, 15.2, 14.8.

Regioisomer **14b**

^{1}H NMR (400 MHz, $CDCl_3$) δ 8.38 (s, 1H), 7.51 (d, J = 7.8 Hz, 1H), 7.32 (d, J = 8.0 Hz, 1H), 7.22 – 7.13 (m, 2H), 7.13 – 7.06 (m, 2H), 4.90 (q, J = 5.8 Hz, 1H), 3.79 – 3.69 (m, 2H), 3.67 (s, 3H), 3.32 (d, J = 5.7 Hz, 2H), 3.04 – 2.97 (m, 2H), 1.84 – 1.71 (m, 2H), 0.98 (t, J = 7.4 Hz, 3H).

^{13}C NMR (101 MHz, $CDCl_3$) δ 171.8, 161.4, 136.2, 127.3, 123.9, 122.2, 119.7, 118.4, 111.5, 109.0, 58.4, 55.6, 54.8, 52.6, 27.3, 15.7, 13.0.

HRMS (EI+) (m/z): $[M]^+$ calcd. for $C_{17}H_{22}N_2O_5S$, 366.1244; found: 366.1254.

2-cyclopropyl-4-(4-fluorophenyl)-3-((isopropylsulfonyl)methyl)quinoline (15a) and 2-cyclopropyl-4-(4-fluorophenyl)-3-((propylsulfonyl)methyl)quinoline (15b)

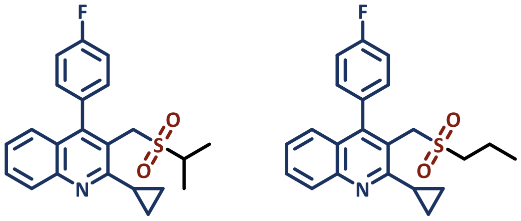

15a **15b**

Prepared according to the general procedure for propane, using aqueous SO_2 (6 wt%, 0.6 mL, 0.6 mmol, 3 equiv.), NaDT (14.65 mg, 0.006 mmol, 3 mol%), propane (73.5 mL, 3 mmol, 15 equiv.) and S1 (71.2 mg, 0.2 mmol, 1 equiv.). After all the outflow from the Signify Eagle reactor was collected, the reaction mixture was stirred for 1 hour at 60 °C. The crude mixture was purified by flash column chromatography (100% *n*-pentane to *n*-pentane 75:25 AcOEt) to afford product **15a** and **15b** as an inseparable mixture of regioisomers (76:24 ratio determined by ^{1}H NMR analysis of the crude reaction mixture), (63.6 mg, 83%) as a white solid.

Regioisomer **15a**

^{1}H NMR (400 MHz, $CDCl_3$) δ 7.88 (m, 1H), 7.53 (m, 1H), 7.33 – 7.18 (m, 3H), 7.18 – 7.06 (m, 3H), 4.43 (s, 2H), 2.83 (h, J = 6.9 Hz, 1H), 2.67 – 2.59 (m, 1H), 1.33 – 1.18 (m, 2H), 1.10 (d, J = 6.9 Hz, 6H), 1.02 (dd, J = 8.0, 3.5 Hz, 2H).

^{13}C NMR (101 MHz, $CDCl_3$) δ 162.7 (d, J = 248.1 Hz), 162.2, 149.3, 147.5, 132.2 (d, J = 3.5 Hz), 132.0 (d, J = 7.7 Hz), 129.8, 129.0, 126.5, 126.4, 125.8, 118.5, 115.5 (d, J = 21.4 Hz), 54.9, 49.5, 15.7, 15.4, 10.3.

^{19}F NMR (282 MHz, $CDCl_3$) δ -113.02.

Regioisomer **15b**

^{1}H NMR (400 MHz, $CDCl_3$) δ 7.88 (m, 1H), 7.53 (m, 1H), 7.33 – 7.18 (m, 3H), 7.18 – 7.06 (m, 3H), 4.49 (s, 2H), 2.59 – 2.51 (m, 2H), 2.59 – 2.51 (m, 1H), 1.62 – 1.48 (m, 2H), 1.33 – 1.18 (m, 2H), 1.02 (dd, J = 8.0, 3.5 Hz, 2H), 0.87 (t, J = 7.4 Hz, 3H).

^{13}C NMR (101 MHz, $CDCl_3$) δ 162.7 (d, J = 248.1 Hz), 162.0, 149.0, 147.5, 132.1 (d, J = 3.5 Hz), 132.0 (d, J = 7.7 Hz), 129.8, 129.0, 126.5, 126.4, 125.9, 118.8, 115.6 (d, J = 21.5 Hz), 56.3, 53.6, 15.7, 15.4, 13.1, 10.5.

^{19}F NMR (282 MHz, $CDCl_3$) δ -112.77.

HRMS (ESI+) (m/z): $[M+H]^+$ calcd. for $C_{22}H_{23}FNO_2S$, 384.1428; found: 384.1434.

7.5 Ethane

((ethylsulfonyl)methyl)benzene (16)

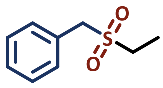

16

Prepared according to the general procedure for ethane, using aqueous SO_2 (6 wt%, 0.6 mL, 0.6 mmol, 3 equiv.), NaDT (14.65 mg, 0.006 mmol, 3 mol%), ethane (70.5 mL, 3 mmol, 15 equiv.) and benzyl bromide (34.2 mg, 23.8 µL, 0.2 mmol, 1 equiv.). After all the outflow from the Signify Eagle reactor was collected, the reaction mixture was stirred for 1 hour at 60 °C. The crude mixture was purified by flash column chromatography (100% *n*-pentane to *n*-pentane 80:20 AcOEt) to afford product **16** (32 mg, 87%) as a white solid.

^{1}H NMR (400 MHz, CDCl3) δ 7.44 – 7.35 (m, 5H), 4.21 (s, 2H), 2.85 (q, J = 7.5 Hz, 2H), 1.34 (t, J = 7.5 Hz, 3H).

^{13}C NMR (101 MHz, CDCl3) δ 130.6, 129.1, 129.1, 128.2, 58.8, 45.5, 6.5.

HRMS (EI+) (m/z): $[M]^+$ calcd. for $C_9H_{12}O_2S$, 184.0553; found: 184.0561.

2-((ethylsulfonyl)methyl)pyridine (17)

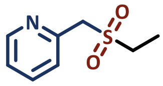

17

Prepared according to the general procedure for ethane, using aqueous SO_2 (6 wt%, 0.6 mL, 0.6 mmol, 3 equiv.), NaDT (14.65 mg, 0.006 mmol, 3 mol%), ethane (70.5 mL, 3 mmol, 15 equiv.) and 2-(bromomethyl)pyridine hydrobromide (50.6 mg, 0.2 mmol, 1 equiv.). After all the outflow from the Signify Eagle reactor was collected, the reaction mixture was stirred for 1 hour at 60 °C. The crude mixture was purified by flash column chromatography (100% *n*-pentane to *n*-pentane 40:60 AcOEt) to afford product **17** (31.5 mg, 85%) as a white solid.

^{1}H NMR (300 MHz, $CDCl_3$) δ 8.57 (ddd, J = 5.0, 1.9, 1.1 Hz, 1H), 7.72 (td, J = 7.7, 1.9 Hz, 1H), 7.48 (dt, J = 7.7, 1.1 Hz, 1H), 7.28 (ddd, J = 7.7, 5.0, 1.1 Hz, 1H), 4.37 (s, 2H), 2.99 (q, J = 7.5 Hz, 2H), 1.37 (t, J = 7.5 Hz, 3H).

^{13}C NMR (101 MHz, $CDCl_3$) δ 149.9, 149.6, 137.2, 126.0, 123.6, 60.5, 46.4, 6.5.

HRMS (EI+) (m/z): $[M]^+$ calcd. for $C_8H_{11}NO_2S$, 185.0505; found: 185.0511.

(*E*)-1-(ethylsulfonyl)-3,7-dimethylocta-2,6-diene (18)

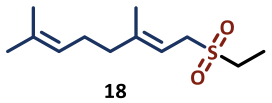

18

Prepared according to the general procedure for ethane, using aqueous SO_2 (6 wt%, 0.6 mL, 0.6 mmol, 3 equiv.), NaDT (14.65 mg, 0.006 mmol, 3 mol%), ethane (70.5 mL, 3 mmol, 15 equiv.) and geranyl bromide (43.4 mg, 39.7 µL, 0.2 mmol, 1 equiv.). After all the outflow from the Signify Eagle reactor was collected, the reaction mixture was stirred at room temperature overnight. The crude mixture was purified by flash column chromatography (100% *n*-pentane to *n*-pentane 80:20 AcOEt) to afford product **18** (19 mg, 41%) as a clear oil.

^{1}H NMR (300 MHz, $CDCl_3$) δ 5.30 (tq, J = 7.8, 1.3 Hz, 1H), 5.10 – 4.98 (m, 1H), 3.69 (d, J = 7.8 Hz, 2H), 2.92 (m, 2H), 2.12 (m, 4H), 1.73 (s, 3H), 1.67 (s, 3H), 1.59 (s, 3H), 1.36 (t, J = 7.5 Hz, 3H).

^{13}C NMR (101 MHz, $CDCl_3$) δ 145.9, 132.3, 123.4, 110.7, 52.4, 45.4, 39.7, 26.2, 25.8, 17.8, 16.8, 6.4.

HRMS (EI+) (m/z): $[M]^+$ calcd. for $C_{12}H_{22}O_2S$, 230.1335; found: 230.1331.

methyl (2-(ethylsulfonyl)acetyl)-*D*-tryptophanate (19)

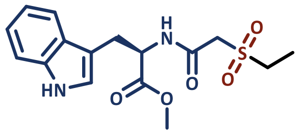

19

Prepared according to the general procedure for ethane, using aqueous SO_2 (6 wt%, 0.6 mL, 0.6 mmol, 3 equiv.), NaDT (14.65 mg, 0.006 mmol, 3 mol%), ethane (70.5 mL, 3 mmol, 15 equiv.) and S3 (68 mg, 0.2 mmol, 1 equiv.). After all the outflow from the Signify Eagle reactor was collected, the reaction mixture was stirred for 1 hour at 60 °C. The crude mixture was purified by flash column chromatography (100% *n*-pentane to *n*-pentane 50:50 AcOEt) to afford product **19** (57 mg, 81%) as a clear oil.

^{1}H NMR (300 MHz, $CDCl_3$) δ 8.35 (s, 1H), 7.51 (d, J = 7.7 Hz, 1H), 7.32 (d, J = 7.8 Hz, 1H), 7.22 – 7.04 (m, 4H), 4.96 – 4.84 (m, 1H), 3.72 (s, 2H), 3.67 (s, 3H), 3.32 (d, J = 5.6 Hz, 2H), 3.05 (q, J = 7.5 Hz, 2H), 1.30 (t, J = 7.5 Hz, 3H).

^{13}C NMR (75 MHz, $CDCl_3$) δ 171.9, 161.4, 136.2, 127.3, 123.8, 122.3, 119.7, 118.4, 111.5, 109.0, 57.6, 53.2, 52.7, 47.7, 27.3, 6.5.

HRMS (EI+) (m/z): $[M]^+$ calcd. for $C_{16}H_{20}N_2O_5S$, 352.1087; found: 352.1104.

2-cyclopropyl-3-((ethylsulfonyl)methyl)-4-(4-fluorophenyl)quinoline (20)

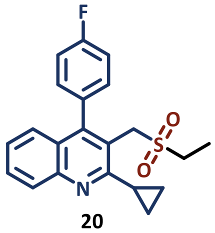

20

Prepared according to the general procedure for ethane, using aqueous SO_2 (6 wt%, 0.6 mL, 0.6 mmol, 3 equiv.), NaDT (14.65 mg, 0.006 mmol, 3 mol%), ethane (70.5 mL, 3 mmol, 15 equiv.) and S1 (71.2 mg, 0.2 mmol, 1 equiv.). After all the outflow from the Signify Eagle reactor was collected, the reaction mixture was stirred for 1 hour at 60 °C. The crude mixture was purified by flash column chromatography (100% *n*-pentane to *n*-pentane 75:25 AcOEt) to afford product **20** (66 mg, 89%) as a white solid.

^{1}H NMR (400 MHz, $CDCl_3$) δ 7.98 (d, J = 8.4 Hz, 1H), 7.64 (ddd, J = 8.4, 6.8, 1.4 Hz, 1H), 7.39 – 7.28 (m, 3H), 7.28 – 7.18 (m, 3H), 4.58 (s, 2H), 2.83 (q, J = 7.4 Hz, 2H), 2.73 – 2.60 (m, 1H), 1.40 – 1.29 (m, 2H), 1.18 (t, J = 7.4 Hz, 3H), 1.12 (dd, J = 8.1, 3.4 Hz, 2H).

^{13}C NMR (101 MHz, $CDCl_3$) δ 162.8 (d, J = 248.6 Hz), 162.0, 149.1, 147.5, 132.1 (d, J = 3.6 Hz), 132.0 (d, J = 7.8 Hz), 129.9, 129.1, 126.4, 126.4, 125.9, 118.8, 115.7 (d, J = 21.5 Hz), 52.8, 48.9, 15.8, 10.5, 6.6.

^{19}F NMR (282 MHz, $CDCl_3$) δ -112.70.

HRMS (ESI+) (m/z): $[M+H]^+$ calcd. for $C_{21}H_{21}FNO_2S$, 370.1272; found: 370.1284.

7.6 Methane

((methylsulfonyl)methyl)benzene (21)

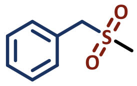

21

Prepared according to the general procedure for methane, using aqueous SO_2 (6 wt%, 4 mL, 4 mmol, 20 equiv.), NaDT (97.67 mg, 0.040 mmol, 20 mol%), methane (447.6 mL, 20 mmol, 100 equiv.) and benzyl bromide (34.2 mg, 23.8 µL, 0.2 mmol, 1 equiv.). After all the outflow from the Signify Eagle reactor was collected, the reaction mixture was stirred for 1 hour at 60 °C. The crude mixture was purified by flash column chromatography (100% *n*-pentane to *n*-pentane 80:20 AcOEt) to afford product **21** (21.4 mg, 63 %) as a white solid. The spectroscopic data are consistent with those reported previously.[12]

^{1}H NMR (400 MHz, $CDCl_3$) δ 7.41 (s, 5H), 4.25 (s, 2H), 2.76 (s, 3H).

^{13}C NMR (101 MHz, $CDCl_3$) δ 130.6, 129.3, 128.4, 61.4, 39.1.

HRMS (FD+) (m/z): $[M]^+$ calcd. for $C_8H_{10}O_2S$, 170.0396; found: 170.0402.

2-((methylsulfonyl)methyl)pyridine (22)

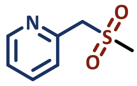

22

Prepared according to the general procedure for methane, using aqueous SO_2 (6 wt%, 4 mL, 4 mmol, 20 equiv.), NaDT (97.67 mg, 0.040 mmol, 20 mol%), methane (447.6 mL, 20 mmol, 100 equiv.) and 2-(bromomethyl)pyridine hydrobromide (50.6 mg, 0.2 mmol, 1 equiv.). After all the outflow from the Signify Eagle reactor was collected, the reaction mixture was stirred for 1 hour at 60 °C. The crude mixture was purified by flash column chromatography (100% *n*-pentane to *n*-pentane 40:60 AcOEt) to afford product **22** (19.5 mg, 57 %) as a white solid.

^{1}H NMR (400 MHz, $CDCl_3$) δ 8.62 (dt, J = 5.0, 1.1 Hz, 1H), 7.79 (td, J = 7.7, 1.9 Hz, 1H), 7.52 (d, J = 7.7 Hz, 1H), 7.38 – 7.32 (m, 1H), 4.45 (s, 2H), 2.93 (s, 3H).

^{13}C NMR (101 MHz, $CDCl_3$) δ 149.7, 149.5, 137.7, 126.2, 123.9, 63.1, 40.2.

HRMS (EI+) (m/z): $[M]^+$ calcd. for $C_7H_9NO_2S$, 171.0349; found: 171.0361.

(*E*)-3,7-dimethyl-1-(methylsulfonyl)octa-2,6-diene (23)

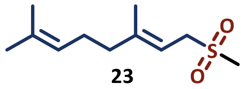

23

Prepared according to a modified version of the general procedure for methane, using aqueous SO_2 (6 wt%, 6 mL, 6 mmol, 30 equiv.), NaDT (146.5 mg, 0.060 mmol, 30 mol%), methane (671.4 mL, 30 mmol, 150 equiv.) and geranyl bromide (43.4 mg, 39.7 µL, 0.2 mmol, 1 equiv.). After all the outflow from the Signify Eagle reactor was collected in a flask containing $NaHCO_3$ (900 mg, 10.8 mmol, 54 equiv.), the reaction was stirred for 1 hour. Then, the solvent was evaporated in vacuo and ethanol (5 mL) was added and the flask was sonicated for 5 minutes. The supernatant was transferred to another vial and the process was repeated. The combined supernatants were evaporated in vacuo. Then, geranyl bromide was added, followed by a 4:1 mixture of CH_3CN/H_2O. The reaction was stirred for 1 hour at 60 °C. Then, the reaction mixture is transferred to a separatory funnel, diluted with water (20 mL) and extracted with DCM (3x20 mL). The combined organic layers were dried over $MgSO_4$ and evaporated in vacuo. The crude mixture was purified by flash column chromatography (100% *n*-pentane to *n*-pentane 80:20 AcOEt) to afford product **23** (11.7 mg, 27 %) as a clear oil. The spectroscopic data are consistent with those reported previously.[13]

^1H NMR (300 MHz, $CDCl_3$) δ 5.41 – 5.28 (m, 1H), 5.11 – 4.99 (m, 1H), 3.72 (d, J = 7.9 Hz, 2H), 2.80 (s, 3H), 2.14 (m, 4H), 1.74 (s, 3H), 1.67 (s, 3H), 1.60 (s, 3H).

^{13}C NMR (75 MHz, $CDCl_3$) δ 146.2, 132.4, 123.4, 111.0, 54.8, 39.7, 39.0, 26.2, 25.8, 17.8, 16.8.

HRMS (EI+) (m/z): $[M]^+$ calcd. for $C_{11}H_{20}O_2S$, 216.1179; found: 216.1183.

methyl (2-(methylsulfonyl)acetyl)-*D*-tryptophanate (24)

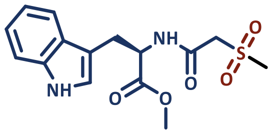

24

Prepared according to a modified version of the general procedure for methane, using aqueous SO_2 (6 wt%, 4 mL, 4 mmol, 20 equiv.), NaDT (97.67 mg, 0.040 mmol, 20 mol%), methane (447.6 mL, 20 mmol, 100 equiv.) and S3 (68 mg, 0.2 mmol, 1 equiv.). After all the outflow from the Signify Eagle reactor was collected in a flask containing $NaHCO_3$ (667 mg, 8 mmol, 40 equiv.), the reaction was stirred for 1 hour. Then, the solvent was evaporated in vacuo and ethanol (5 mL) was added and the flask was sonicated for 5 minutes. The supernatant was transferred to another vial and the process was repeated. The combined supernatants were evaporated in vacuo. Then, S3 was added, followed by a 4:1 mixture of CH_3CN/H_2O. The reaction was stirred for 1 hour at 60 °C. Then, the reaction mixture is transferred to a separatory funnel, diluted with water (20 mL) and extracted with DCM (3x20 mL). The combined organic layers were dried over $MgSO_4$ and evaporated in vacuo. The crude mixture was purified by flash column chromatography (100% *n*-pentane to *n*-pentane 50:50 AcOEt) to afford product **24** (51.3 mg, 76 %) as a clear oil.

^1H NMR (300 MHz, $CDCl_3$) δ 8.43 (s, 1H), 7.49 (d, J = 7.7 Hz, 1H), 7.30 (d, J = 7.7 Hz, 1H), 7.22 – 7.04 (m, 4H), 4.97 – 4.84 (m, 1H), 3.79 – 3.66 (m, 2H), 3.66 (s, 3H), 3.31 (d, J = 5.5 Hz, 2H), 2.90 (s, 3H).

^{13}C NMR (101 MHz, $CDCl_3$) δ 171.9, 161.5, 136.1, 127.3, 123.9, 122.2, 119.7, 118.4, 111.5, 108.9, 60.1, 53.2, 52.7, 41.1, 27.3.

HRMS (EI+) (m/z): $[M]^+$ calcd. for $C_{15}H_{18}N_2O_5S$, 338.0931; found: 338.0951.

2-cyclopropyl-4-(4-fluorophenyl)-3-((methylsulfonyl)methyl)quinoline (25)

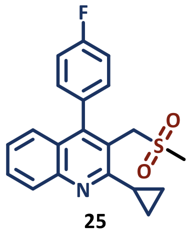

25

Prepared according to the general procedure for methane, using aqueous SO_2 (6 wt%, 4 mL, 4 mmol, 20 equiv.), NaDT (97.67 mg, 0.040 mmol, 20 mol%), methane (447.6 mL, 20 mmol, 100 equiv.) and S1 (71.2 mg, 0.2 mmol, 1 equiv.). After all the outflow from the Signify Eagle reactor was collected, the reaction mixture was stirred for 1 hour at 60 °C. The crude mixture was purified by flash column chromatography (100% *n*-pentane to *n*-pentane 75:25 AcOEt) to afford product **25** (34.8 mg, 49 %) as a white solid.

^1H NMR (300 MHz, $CDCl_3$) δ 7.98 (d, J = 8.4 Hz, 1H), 7.66 (ddd, J = 8.4, 6.8, 1.4 Hz, 1H), 7.42 – 7.30 (m, 3H), 7.30 – 7.19 (m, 3H), 4.67 (s, 2H), 2.75 (s, 3H), 2.68 – 2.53 (m, 1H), 1.37 (s, 2H), 1.14 (dd, J = 8.0, 3.2 Hz, 2H).

^{13}C NMR (101 MHz, $CDCl_3$) δ 162.8 (d, J = 248.9 Hz), 161.8, 149.0, 147.7, 132.1 (d, J = 3.5 Hz), 132.0 (d, J = 8.0 Hz), 130.1, 129.2, 126.5, 126.4, 126.0, 119.0, 115.9 (d, J = 21.5 Hz), 55.8, 42.2, 15.8, 10.8.

^{19}F NMR (282 MHz, $CDCl_3$) δ -112.49

HRMS (EI+) (m/z): $[M]^+$ calcd. for $C_{20}H_{18}FNO_2S$, 355.1037; found: 355.1037.

7.7 Functionalization

2-(ethylsulfonyl)benzo[*d*]thiazole (26)

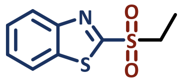

26

To a nitrogen-purged, screw-capped vial, fitted with a rubber septum and charged with NaDT (14.65 mg, 0.006 mmol, 3 mol%) degassed CH_3CN is added (2.4 mL), followed by aqueous SO_2 (6 wt%, 0.6 mL, 0.6 mmol, 3 equiv.). The stock solution is charged in a gastight syringe, positioned in a syringe pump and combined with a stream of ethane gas (70.5 mL, 3 mmol, 15 equiv.) through a T-mixer into a filling loop, with a liquid flow rate of 0.16 mL·min-1 and a ethane gas flow rate of 4 mL·min-1. A BPR of 2.8 bar is used during the loop filling. Next, the filling loop is connected to the reactor, the system is pressurized to 52 bar and the reaction mixture is pumped over the Signify Eagle reactor (365 nm, 144 W output power, FEP capillary: 0.5 mm ID, 1.5 mL) at a flow rate of 0.0125 mL·min-1, resulting in a residence time of 2 h. The obtained reaction mixture is collected into a nitrogen-purged vial containing $NaHCO_3$ (100 mg, 1.2 mmol, 6 equiv.) and degassed CH_3CN (1 mL). The vial is stirred at rt during the collection of the outflow from the Signify Eagle reactor. After all the outflow is collected, the mixture is stirred for an additional 30 min. The solvent was evaporated in vacuo and 2-chlorobenzothiazole (26 uL, 33.9 mg, 0.2 mmol, 1 equiv.) and DMA (5 mL) were added to the vial. The vial was purged with nitrogen and heated to 100 °C for 16 h. The mixture was then transferred to a separatory funnel, diluted with water (20 mL) and extracted with EtOAc (3x20 mL). The combined organic layers were dried over $MgSO_4$ and evaporated in vacuo. The crude mixture was purified by flash column chromatography (100% *n*-pentane to 20%

AcOEt in *n*-pentane) to afford product **26** (34.5 mg, 76%) as a white solid. The spectroscopic data are consistent with those reported previously.[14]

^{1}H NMR (400 MHz, $CDCl_3$) δ 8.28 – 8.21 (m, 1H), 8.08 – 8.01 (m, 1H), 7.71 – 7.57 (m, 2H), 3.57 (q, J = 7.4 Hz, 2H), 1.47 (t, J = 7.4 Hz, 3H).

^{13}C NMR (101 MHz, $CDCl_3$) δ 165.3, 152.7, 136.8, 128.0, 127.6, 125.4, 122.3, 49.3, 7.1.

HRMS (ESI+) (m/z): $[M+H]^+$ calcd. for $C_9H_{10}NO_2S_2$, 228.0147; found: 228.0158.

1-(5-(ethylsulfonyl)thiophen-2-yl)ethan-1-one (27)

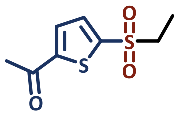

27

To a nitrogen-purged, screw-capped vial, fitted with a rubber septum and charged with NaDT (14.65 mg, 0.006 mmol, 3 mol%) degassed CH_3CN is added (2.4 mL), followed by aqueous SO_2 (6 wt%, 0.6 mL, 0.6 mmol, 3 equiv.). The stock solution is charged in a gastight syringe, positioned in a syringe pump and combined with a stream of ethane gas (70.5 mL, 3 mmol, 15 equiv.) through a T-mixer into a filling loop, with a liquid flow rate of 0.16 mL·min-1 and a ethane gas flow rate of 4 mL·min-1. A BPR of 2.8 bar is used during the loop filling. Next, the filling loop is connected to the reactor, the system is pressurized to 52 bar and the reaction mixture is pumped over the Signify Eagle reactor (365 nm, 144 W output power, FEP capillary: 0.5 mm ID, 1.5 mL) at a flow rate of 0.0125 mL·min-1, resulting in a residence time of 2 h. The obtained reaction mixture is collected into a nitrogen-purged vial containing $NaHCO_3$ (100 mg, 1.2 mmol, 6 equiv.) and degassed CH_3CN (1 mL). The vial is stirred at rt during the collection of the outflow from the Signify Eagle reactor. After all the outflow is collected, the mixture is stirred for an additional 30 min. The solvent was evaporated in vacuo and 2-acetyl-5-bromothiophene (41 mg, 0.2 mmol, 1 equiv.) and DMSO (3 mL) were added to the vial. The vial was purged with nitrogen and heated to 110 °C for 20 h. The mixture was then transferred to a separatory funnel, diluted with EtOAc (20 mL) and washed with brine (4x20 mL). The organic layer was dried over $MgSO_4$ and evaporated in vacuo. The crude mixture was purified by flash column chromatography (100% *n*-pentane to 20% AcOEt in *n*-pentane) to afford product **27** (27.5 mg, 63%) as a slightly yellow oil.

^{1}H NMR (300 MHz, $CDCl_3$) δ 7.65 (m, 2H), 3.23 (q, J = 7.4 Hz, 2H), 2.59 (s, 3H), 1.34 (t, J = 7.4 Hz, 3H).

^{13}C NMR (75 MHz, $CDCl_3$) δ 190.4, 150.8, 146.0, 134.1, 131.4, 52.0, 27.0, 7.8.

HRMS (ES+) (m/z): $[M]^+$ calcd. for $C_8H_{10}O_3S_2$, 218.0066; found: 218.0061.

(ethylsulfonyl)benzene (28)

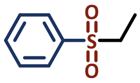

28

To a nitrogen-purged, screw-capped vial, fitted with a rubber septum and charged with NaDT (14.65 mg, 0.006 mmol, 3 mol%) degassed CH_3CN is added (2.4 mL), followed by aqueous SO_2 (6 wt%, 0.6 mL, 0.6 mmol, 3 equiv.). The stock solution is charged in a gastight syringe, positioned in a syringe pump and combined with a stream of ethane gas (70.5 mL, 3 mmol, 15 equiv.) through a T-mixer into a filling loop, with a liquid flow rate of 0.16 mL·min-1 and an ethane gas flow rate of 4 mL·min-1. A BPR of 2.8 bar is used during the loop filling. Next, the filling loop is connected to the reactor, the system is pressurized to 52 bar using an HPLC pump and the reaction mixture is pumped over the Signify Eagle reactor (365 nm, 144 W output power, FEP capillary: 0.5 mm ID, 1.5 mL) at a flow rate of 0.0125 mL·min-1, resulting in a residence time of 2 h. The obtained reaction mixture is collected into a nitrogen-purged vial containing $NaHCO_3$ (100 mg, 1.2 mmol, 6 equiv.), diphenyliodonium triflate (86 mg, 0.2 mmol, 1 equiv.) and degassed CH_3CN (1 mL). The vial is heated to 60 °C during the collection of the outflow from the Signify Eagle reactor.

After all the outflow is collected, the vial was heated for an additional 18h. Then, the reaction mixture is transferred to a separatory funnel, diluted with water (20 mL), and extracted with DCM (3x20 mL). The combined organic layers were dried over $MgSO_4$ and evaporated in vacuo. The crude mixture was purified by flash column chromatography (100% *n*-pentane to 20% AcOEt in *n*-pentane) to afford product **28** (26.8 mg, 79%) as a yellow solid. The spectroscopic data are consistent with those reported previously.[15]

^{1}H NMR (400 MHz, $CDCl_3$) δ 7.90 (dd, J = 7.5, 1.5 Hz, 2H), 7.65 (t, J = 7.5 Hz, 1H), 7.56 (t, J = 7.5 Hz, 2H), 3.11 (q, J = 7.4 Hz, 2H), 1.26 (t, J = 7.4 Hz, 3H).

^{13}C NMR (101 MHz, $CDCl_3$) δ 138.6, 133.7, 129.3, 128.3, 50.6, 7.5.

HRMS (EI+) (m/z): $[M]^+$ calcd. for $C_8H_{10}O_2S$, 170.0396; found: 170.0407.

methyl 4-(ethylsulfonyl)benzoate (29)

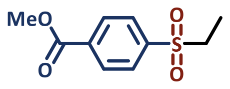

29

To a nitrogen-purged, screw-capped vial, fitted with a rubber septum and charged with NaDT (14.65 mg, 0.006 mmol, 3 mol%) degassed CH_3CN is added (2.4 mL), followed by aqueous SO_2 (6 wt%, 0.6 mL, 0.6 mmol, 3 equiv.). The stock solution is charged in a gastight syringe, positioned in a syringe pump and combined with a stream of ethane gas (70.5 mL, 3 mmol, 15 equiv.) through a T-mixer into a filling loop, with a liquid flow rate of 0.16 mL·min-1 and an ethane gas flow rate of 4 mL·min-1. A BPR of 2.8 bar is used during the loop filling. Next, the filling loop is connected to the reactor, the system is pressurized to 52 bar using an HPLC pump and the reaction mixture is pumped over the Signify Eagle reactor (365 nm, 144 W output power, FEP capillary: 0.5 mm ID, 1.5 mL) at a flow rate of 0.0125 mL·min-1, resulting in a residence time of 2 h. The obtained reaction mixture is collected into a nitrogen-purged vial containing $NaHCO_3$ (100 mg, 1.2 mmol, 6 equiv.) and degassed CH_3CN (1 mL). The vial is stirred at rt during the collection of the outflow from the Signify Eagle reactor. After all the outflow is collected, bis(4-(methoxycarbonyl)phenyl)iodonium triflate (109.2 mg, 0.2 mmol, 1 equiv.) is added and the vial is heated to 60 °C for 18h. Then, the reaction mixture is transferred to a separatory funnel, diluted with water (20 mL) and extracted with DCM (3x20 mL). The combined organic layers are dried over $MgSO_4$ and evaporated in vacuo. The crude mixture was purified by flash column chromatography (100% *n*-pentane to 20% AcOEt in *n*-pentane) to afford product **29** (30.9 mg, 68%) as a white solid. The spectroscopic data are consistent with those reported previously.[16]

^{1}H NMR (400 MHz, $CDCl_3$) δ 8.26 – 8.16 (d, J = 8.1 Hz, 2H), 8.03 – 7.92 (d, J = 8.1 Hz, 2H), 3.95 (s, 3H), 3.13 (q, J = 7.4 Hz, 2H), 1.27 (t, J = 7.4 Hz, 3H).

^{13}C NMR (101 MHz, $CDCl_3$) δ 165.5, 142.4, 134.9, 130.4, 128.4, 52.8, 50.6, 7.4.

HRMS (EI+) (m/z): $[M]^+$ calcd. for $C_{10}H_{12}O_4S$, 228.0451; found: 228.0460.

3-(ethylsulfonyl)-1-phenylpyrrolidine-2,5-dione (30)

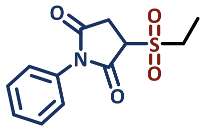

30

To a nitrogen-purged, screw-capped vial, fitted with a rubber septum and charged with NaDT (14.65 mg, 0.006 mmol, 3 mol%) degassed CH_3CN is added (2.4 mL), followed by aqueous SO_2 (6 wt%, 0.6 mL, 0.6 mmol, 3 equiv.). The stock solution is charged in a gastight syringe, positioned in a syringe pump and combined with a stream of ethane gas (70.5 mL, 3 mmol, 15 equiv.) through a T-mixer into a filling loop, with a liquid flow rate of 0.16 mL·min-1 and an ethane gas flow rate of 4 mL·min-1. A BPR of 2.8 bar is used during the loop filling. Next, the filling loop is connected to the reactor, the system is pressurized to 52 bar using an HPLC pump and the reaction mixture is pumped over the Signify Eagle

reactor (365 nm, 144 W output power, FEP capillary: 0.5 mm ID, 1.5 mL) at a flow rate of 0.0125 mL·min-1, resulting in a residence time of 2 h. The obtained reaction mixture is collected into a nitrogen-purged vial containing $NaHCO_3$ (100 mg, 1.2 mmol, 6 equiv.) and degassed CH_3CN (1 mL). The vial is stirred was rt during the collection of the outflow from the Signify Eagle reactor. After all the outflow was collected, the solvent is evaporated in vacuo. Then, *N*-phenyl maleimide (34.6 mg, 0.2 mmol, 1 equiv.) and 2M HCl (1 mL) was added, and the reaction mixture was stirred for 72h at room temperature. After the reaction was complete, the reaction mixture is transferred to a separatory funnel, diluted with water (20 mL) and extracted with AcOEt (3x20 mL). The combined organic layers are dried over $MgSO_4$ and evaporated in vacuo. The crude mixture was purified by flash column chromatography (100% *n*-pentane to 40% AcOEt in *n*-pentane) to afford product **30** (29.9 mg, 56%) as a clear oil. The spectroscopic data are consistent with those reported previously.[17]

^1H NMR (300 MHz, $CDCl_3$) δ 7.56 – 7.37 (m, 3H), 7.32 – 7.22 (m, 2H), 4.38 (dd, J = 9.7, 3.9 Hz, 1H), 3.62 – 3.38 (m, 3H), 3.26 – 3.11 (dd, J = 19.2, 9.7, 1H), 1.52 (t, J = 7.4 Hz, 3H).

^{13}C NMR (75 MHz, $CDCl_3$) δ 172.2, 168.9, 131.1, 129.5, 129.4, 126.5, 59.4, 47.3, 27.9, 6.5.

HRMS (EI+) (m/z): $[M]^+$ calcd. for $C_{12}H_{13}NO_4S$, 267.0560; found: 267.0569.

5-(ethylsulfonyl)hex-5-en-1-yl 4-fluorobenzoate (31)

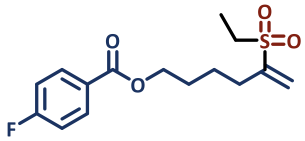

31

To a nitrogen-purged, screw-capped vial, fitted with a rubber septum and charged with NaDT (14.65 mg, 0.006 mmol, 3 mol%) degassed CH_3CN is added (2.4 mL), followed by aqueous SO_2 (6 wt%, 0.6 mL, 0.6 mmol, 3 equiv.). The stock solution is charged in a gastight syringe, positioned in a syringe pump and combined with a stream of ethane gas (70.5 mL, 3 mmol, 15 equiv.) through a T-mixer into a filling loop, with a liquid flow rate of 0.16 mL·min-1 and an ethane gas flow rate of 4 mL·min-1. A BPR of 2.8 bar is used during the loop filling. Next, the filling loop is connected to the reactor, the system is pressurized to 52 bar using an HPLC pump and the reaction mixture is pumped over the Signify Eagle reactor (365 nm, 144 W output power, FEP capillary: 0.5 mm ID, 1.5 mL) at a flow rate of 0.0125 mL·min-1, resulting in a residence time of 2 h. The obtained reaction mixture was collected in a nitrogen-purged vial containing $NaHCO_3$ (100 mg, 1.2 mmol, 6 equiv.) and degassed CH_3CN (1 mL). The vial is stirred at rt during the collection of the outflow from the Signify Eagle reactor. After all the outflow was collected, the solvent was evaporated in vacuo. Then, S4 (104.8 mg, 0.2 mmol, 1 equiv.) and DCE (2 mL) was added, and the reaction mixture is stirred for 17h at 50 °C. After the reaction is complete, the solvent was evaporated in vacuo and the crude mixture was purified by flash column chromatography (100% *n*-pentane to 20% AcOEt in *n*-pentane) to afford product **31** (44.1 mg, 70%) as a clear oil.

^1H NMR (400 MHz, $CDCl_3$) δ 8.07 – 7.98 (m, 2H), 7.14 – 7.04 (m, 2H), 6.24 (s, 1H), 5.86 – 5.81 (m, 1H), 4.34 (t, J = 6.2 Hz, 2H), 2.97 (q, J = 7.4 Hz, 2H), 2.51 – 2.42 (m, 2H), 1.91 – 1.70 (m, 4H), 1.28 (t, J = 7.4 Hz, 3H).

^{13}C NMR (101 MHz, $CDCl_3$) δ 165.8 (d, J = 253.9 Hz), 165.6, 147.9, 132.1 (d, J = 9.2 Hz), 126.5 (d, J = 3.0 Hz), 125.5, 115.6 (d, J = 22.0 Hz), 64.5, 46.7, 29.5, 28.2, 24.4, 7.0.

^{19}F NMR (282 MHz, $CDCl_3$) δ -105.63.

HRMS (EI+) (m/z): $[M]^+$ calcd. for $C_{15}H_{19}FO_4S$, 314.0983; found: 314.0993.

1-(ethylsulfonyl)-1*H*-benzo[*d*]imidazole (32)

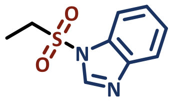

32

To a nitrogen-purged, screw-capped vial, fitted with a rubber septum and charged with NaDT (14.65 mg, 0.006 mmol, 3 mol%) degassed CH_3CN is added (2.4 mL), followed by aqueous SO_2 (6 wt%, 0.6 mL, 0.6 mmol, 3 equiv.). The stock solution is charged in a gastight syringe, positioned in a syringe pump and combined with a stream of ethane gas (70.5 mL, 3 mmol, 15 equiv.) through a T-mixer into a filling loop, with a liquid flow rate of 0.16 mL·min-1 and an ethane gas flow rate of 4 mL·min-1. A BPR of 2.8 bar is used during the loop filling. Next, the filling loop is connected to the reactor, the system is pressurized to 52 bar using an HPLC pump and the reaction mixture is pumped over the Signify Eagle reactor (365 nm, 144 W output power, FEP capillary: 0.5 mm ID, 1.5 mL) at a flow rate of 0.0125 mL·min-1, resulting in a residence time of 2 h. The obtained reaction mixture is collected into a nitrogen-purged vial containing $NaHCO_3$ (100 mg, 1.2 mmol, 6 equiv.) and degassed CH_3CN (1 mL). The vial is stirred at rt during the collection of the outflow from the Signify Eagle reactor. After all the outflow is collected, the vial is cooled to 0 °C and *N*-chlorosuccinimide (40 mg, 0.3 mmol, 1.5 equiv.) is added. The reaction mixture is allowed to warm up to rt and stirred for another 30 min. The mixture is transferred to a separatory funnel, diluted with brine (20 mL) and extracted with DCM (3x20 mL). The combined organic layers are dried over $MgSO_4$ and evaporated in vacuo. The flask was purged with nitrogen and the residue was dissolved in THF (4 mL) and benzimidazole (23.6 mg, 0.2 mmol, 1 equiv.) was added, followed by Et_3N (56 uL, 0.4 mmol, 2 equiv.). The mixture was stirred at rt for 24 h, after which it was transferred to a separatory funnel, diluted with 0.1 M HCl (20 mL) and extracted with DCM (3x20 mL). The combined organic layers are dried over $MgSO_4$ and evaporated in vacuo. The crude mixture was purified by flash column chromatography (100% *n*-pentane to 40% AcOEt in *n*-pentane) to afford product **32** (28.6 mg, 68%) as a clear oil.

^{1}H NMR (300 MHz, $CDCl_3$) δ 8.25 (s, 1H), 7.92 – 7.76 (m, 2H), 7.52 – 7.37 (m, 2H), 3.43 (q, J = 7.4 Hz, 2H), 1.31 (t, J = 7.4 Hz, 3H).

^{13}C NMR (101 MHz, $CDCl_3$) δ 144.1, 141.7, 131.2, 125.9, 125.1, 121.4, 112.3, 49.5, 8.1.

HRMS (FD+) (m/z): $[M]^+$ calcd. for $C_9H_{10}N_2O_2S$, 210.0458; found: 210.0469.

***tert*-butyl 2-((ethylsulfonyl)oxy)-7-azaspiro[3.5]nonane-7-carboxylate (33)**

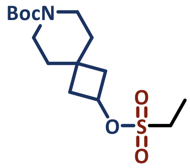

33

To a nitrogen-purged, screw-capped vial, fitted with a rubber septum and charged with NaDT (14.65 mg, 0.006 mmol, 3 mol%) degassed CH_3CN is added (2.4 mL), followed by aqueous SO_2 (6 wt%, 0.6 mL, 0.6 mmol, 3 equiv.). The stock solution is charged in a gastight syringe, positioned in a syringe pump and combined with a stream of ethane gas (70.5 mL, 3 mmol, 15 equiv.) through a T-mixer into a filling loop, with a liquid flow rate of 0.16 mL·min-1 and a ethane gas flow rate of 4 mL·min-1. A BPR of 2.8 bar is used during the loop filling. Next, the filling loop is connected to the reactor, the system is pressurized to 52 bar and the reaction mixture is pumped over the Signify Eagle reactor (365 nm, 144 W output power, FEP capillary: 0.5 mm ID, 1.5 mL) at a flow rate of 0.0125 mL·min-1, resulting in a residence time of 2 h. The obtained reaction mixture is collected into a nitrogen-purged vial containing $NaHCO_3$ (100 mg, 1.2 mmol, 6 equiv.) and degassed CH_3CN (1 mL). The vial is stirred at rt during the collection of the outflow from the Signify Eagle reactor. After all the outflow is collected, the vial is cooled to 0 °C and *N*-chlorosuccinimide (40 mg, 0.3 mmol, 1.5 equiv.) is added. The reaction mixture is allowed to warm up to rt and stirred for another 30 min. The mixture is transferred to a separatory funnel, diluted with brine (20 mL) and extracted with DCM (3x20 mL). The combined organic layers are dried over $MgSO_4$ and evaporated in vacuo. The flask was purged with nitrogen and the residue was dissolved in dry DCM (6 mL), and *tert*-butyl 2-hydroxy-7-azaspiro[3.5]nonane-7-carboxylate (48.3 mg, 0.2 mmol, 1 equiv.) was added at 0 °C, followed by Et_3N (56 uL, 0.4 mmol, 2 equiv.). The mixture was allowed to warm up to rt and stirred for 18 h, after which

it was transferred to a separatory funnel, diluted with water (20 mL) and extracted with DCM (3x20 mL). The combined organic layers are dried over $MgSO_4$ and evaporated in vacuo. The crude mixture was purified by flash column chromatography (100% *n*-pentane to 20% AcOEt in *n*-pentane) to afford product **33** (29.8 mg, 45%) as a slightly yellow oil.

^{1}H NMR (400 MHz, $CDCl_3$) δ 5.01 (p, J = 7.3 Hz, 1H), 3.36 – 3.25 (m, 4H), 3.07 (q, J = 7.4 Hz, 2H), 2.44 – 2.34 (m, 2H), 2.10 – 2.00 (m, 2H), 1.59 – 1.47 (m, 4H), 1.43 (s, 9H), 1.39 (t, J = 7.4 Hz, 3H).

^{13}C NMR (101 MHz, $CDCl_3$) δ 154.9, 79.6, 71.0, 45.7, 40.3, 39.1, 36.0, 32.0, 28.5, 8.2.

HRMS (FD+) (m/z): $[M]^+$ calcd. for $C_{15}H_{27}NO_5S$, 333.1604; found: 333.1605.

1-(6-chloropyridazin-3-yl)piperidin-4-yl ethanesulfonate (34) (via RSO_2Cl)

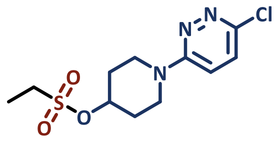

34

To a nitrogen-purged, screw-capped vial, fitted with a rubber septum and charged with NaDT (14.65 mg, 0.006 mmol, 3 mol%) degassed CH_3CN is added (2.4 mL), followed by aqueous SO_2 (6 wt%, 0.6 mL, 0.6 mmol, 3 equiv.). The stock solution is charged in a gastight syringe, positioned in a syringe pump and combined with a stream of ethane gas (70.5 mL, 3 mmol, 15 equiv.) through a T-mixer into a filling loop, with a liquid flow rate of 0.16 mL·min-1 and a ethane gas flow rate of 4 mL·min-1. A BPR of 2.8 bar is used during the loop filling. Next, the filling loop is connected to the reactor, the system is pressurized to 52 bar and the reaction mixture is pumped over the Signify Eagle reactor (365 nm, 144 W output power, FEP capillary: 0.5 mm ID, 1.5 mL) at a flow rate of 0.0125 mL·min-1, resulting in a residence time of 2 h. The obtained reaction mixture is collected into a nitrogen-purged vial containing $NaHCO_3$ (100 mg, 1.2 mmol, 6 equiv.) and degassed CH_3CN (1 mL). The vial is stirred at rt during the collection of the outflow from the Signify Eagle reactor. After all the outflow is collected, the vial is cooled to 0 °C and *N*-chlorosuccinimide (40 mg, 0.3 mmol, 1.5 equiv.) is added. The reaction mixture is allowed to warm up to rt and stirred for another 30 min. The mixture is transferred to a separatory funnel, diluted with brine (20 mL) and extracted with DCM (3x20 mL). The combined organic layers are dried over $MgSO_4$ and evaporated in vacuo. The flask was purged with nitrogen and the residue was dissolved in dry DCM (6 mL), and 1-(6-chloropyridazin-3-yl)piperidin-4-ol (42.7 mg, 0.2 mmol, 1 equiv.) was added at 0 °C, followed by Et_3N (56 uL, 0.4 mmol, 2 equiv.). The mixture was allowed to warm up to rt and stirred for 18 h, after which it was transferred to a separatory funnel, diluted with water (20 mL) and extracted with DCM (3x20 mL). The combined organic layers are dried over $MgSO_4$ and evaporated in vacuo. The crude mixture was purified by flash column chromatography (100% *n*-pentane to 20% AcOEt in *n*-pentane) to afford product **34** (18.8 mg, 31%) as a white solid.

^{1}H NMR (300 MHz, $CDCl_3$) δ 7.22 (d, J = 9.5 Hz, 1H), 6.94 (d, J = 9.5 Hz, 1H), 5.06 – 4.92 (m, 1H), 3.91 (ddd, J = 11.5, 7.4, 3.8 Hz, 2H), 3.59 (ddd, J = 13.6, 7.7, 3.8 Hz, 2H), 3.16 (q, J = 7.4 Hz, 2H), 2.16 – 2.03 (m, 2H), 2.03 – 1.89 (m, 2H), 1.44 (t, J = 7.4 Hz, 3H).

^{13}C NMR (101 MHz, $CDCl_3$) δ 158.7, 147.1, 129.0, 115.6, 76.7, 46.2, 42.3, 31.2, 8.3.

HRMS (ESI+) (m/z): $[M+H]^+$ calcd. for $C_{11}H_{17}ClN_3O_3S$, 306.0674; found: 306.0680.

1-(6-chloropyridazin-3-yl)piperidin-4-yl ethanesulfonate (34) (via RSO_2F)

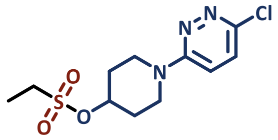

34

To a nitrogen-purged, screw-capped vial, fitted with a rubber septum and charged with NaDT (14.65 mg, 0.006 mmol, 3 mol%) degassed CH_3CN is added (2.4 mL), followed by aqueous SO_2 (6 wt%, 0.6 mL, 0.6 mmol, 3 equiv.). The stock solution is charged in a gastight syringe, positioned in a syringe pump and combined with a stream of ethane gas (70.5 mL, 3 mmol, 15 equiv.) through a T-mixer into a filling loop, with a liquid flow rate of 0.16 mL·min-1 and a ethane gas flow rate of 4 mL·min-1. A BPR of 2.8 bar is used during the loop filling. Next, the filling loop is connected to the reactor, the system is pressurized to 52 bar and the reaction mixture is pumped over the Signify Eagle reactor (365 nm, 144 W output power, FEP capillary: 0.5 mm ID, 1.5 mL) at a flow rate of 0.0125 mL·min-1, resulting in a residence time of 2 h. The obtained reaction mixture is collected into a nitrogen-purged vial containing $NaHCO_3$ (100 mg, 1.2 mmol, 6 equiv.) and degassed CH_3CN (1 mL). The vial is stirred at rt during the collection of the outflow from the Signify Eagle reactor. After all the outflow is collected and Selectfluor (212 mg, 0.6 mmol, 3 equiv.) is added. The reaction mixture is stirred for 2 h. The mixture is transferred to a separatory funnel, diluted with brine (20 mL) and extracted with DCM (3x20 mL). The combined organic layers are dried over $MgSO_4$ and evaporated in vacuo. The flask was purged with nitrogen and the residue was dissolved in dry DCM (6 mL), and 1-(6-chloropyridazin-3-yl)piperidin-4-ol (42.7 mg, 0.2 mmol, 1 equiv.) was added at 0 °C, followed by Et_3N (56 uL, 0.4 mmol, 2 equiv.). The mixture was allowed to warm up to rt and stirred for 48 h, after which it was transferred to a separatory funnel, diluted with water (20 mL) and extracted with DCM (3x20 mL). The combined organic layers are dried over $MgSO_4$ and evaporated in vacuo. The crude mixture was purified by flash column chromatography (100% *n*-pentane to 20% AcOEt in *n*-pentane) to afford product **34** (12.2 mg, 20%) as a white solid. The analysis is consistent with the previously characterized product (*vide supra*).

2-chloro-11-(4-(ethylsulfonyl)piperazin-1-yl)dibenzo[*b*,*f*][1,4]oxazepane (35)

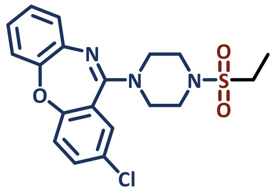

35

To a nitrogen-purged, screw-capped vial, fitted with a rubber septum and charged with NaDT (14.65 mg, 0.006 mmol, 3 mol%) degassed CH_3CN is added (2.4 mL), followed by aqueous SO_2 (6 wt%, 0.6 mL, 0.6 mmol, 3 equiv.). The stock solution is charged in a gastight syringe, positioned in a syringe pump and combined with a stream of ethane gas (70.5 mL, 3 mmol, 15 equiv.) through a T-mixer into a filling loop, with a liquid flow rate of 0.16 mL·min-1 and an ethane gas flow rate of 4 mL·min-1. A BPR of 2.8 bar is used during the loop filling. Next, the filling loop is connected to the reactor, the system is pressurized to 52 bar using an HPLC pump and the reaction mixture is pumped over the Signify Eagle reactor (365 nm, 144 W output power, FEP capillary: 0.5 mm ID, 1.5 mL) at a flow rate of 0.0125 mL·min-1, resulting in a residence time of 2 h. The obtained reaction mixture is collected into a nitrogen-purged vial containing $NaHCO_3$ (100 mg, 1.2 mmol, 6 equiv.) and degassed CH_3CN (1 mL). The vial is stirred at rt during the collection of the outflow from the Signify Eagle reactor. After all the outflow is collected, the vial is cooled to 0 °C and *N*-chlorosuccinimide (40 mg, 0.3 mmol, 1.5 equiv.) is added. The reaction mixture is allowed to warm up to rt and stirred for another 30 min. The mixture is transferred to a separatory funnel, diluted with brine (20 mL) and extracted with DCM (3x20 mL). The combined organic layers are dried over $MgSO_4$ and evaporated in vacuo. The flask was purged with nitrogen and the residue was dissolved in THF (4 mL) and Amoxapine (62.7 mg, 0.2 mmol, 1 equiv.) was added. The mixture was stirred at rt for 24 h, after which it was transferred to a separatory funnel, diluted with 0.1 M HCl (20 mL) and extracted with DCM (3x20 mL). The combined organic layers are dried over $MgSO_4$ and evaporated in vacuo. The crude mixture was purified by flash column chromatography (100% *n*-pentane to 40% AcOEt in *n*-pentane) to afford product **35** (37.6 mg, 46%) as a white solid.

^{1}H NMR (400 MHz, $CDCl_3$) δ 7.42 (dd, J = 8.6, 2.6 Hz, 1H), 7.30 (d, J = 2.6 Hz, 1H), 7.20 (d, J = 8.6 Hz, 1H), 7.17 – 7.06 (m, 3H), 7.06 – 6.98 (m, 1H), 3.71 – 3.55 (m, 4H), 3.49 – 3.42 (m, 4H), 2.99 (q, J = 7.4 Hz, 2H), 1.40 (t, J = 7.4 Hz, 3H).

^{13}C NMR (101 MHz, $CDCl_3$) δ 159.5, 158.5, 151.8, 139.8, 133.0, 130.6, 128.9, 127.2, 126.0, 125.2, 124.8, 123.0, 120.3, 47.6, 45.5, 44.1, 7.9.

HRMS (EI+) (m/z): $[M]^+$ calcd. for $C_{19}H_{20}ClN_3O_3S$, 405.0908; found: 405.0916.

N-(((1R,4aS,10aR)-7-isopropyl-1,4a-dimethyl-1,2,3,4,4a,9,10,10a-octahydrophenanthren-1-yl)methyl)ethanesulfonamide (36)

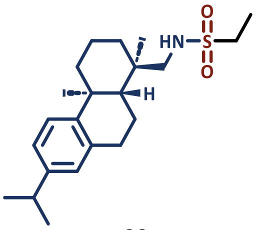

36

To a nitrogen-purged, screw-capped vial, fitted with a rubber septum and charged with NaDT (14.65 mg, 0.006 mmol, 3 mol%) degassed CH_3CN is added (2.4 mL), followed by aqueous SO_2 (6 wt%, 0.6 mL, 0.6 mmol, 3 equiv.). The stock solution is charged in a gastight syringe, positioned in a syringe pump and combined with a stream of ethane gas (70.5 mL, 3 mmol, 15 equiv.) through a T-mixer into a filling loop, with a liquid flow rate of 0.16 mL·min-1 and an ethane gas flow rate of 4 mL·min-1. A BPR of 2.8 bar is used during the loop filling. Next, the filling loop is connected to the reactor, the system is pressurized to 52 bar using an HPLC pump and the reaction mixture is pumped over the Signify Eagle reactor (365 nm, 144 W output power, FEP capillary: 0.5 mm ID, 1.5 mL) at a flow rate of 0.0125 mL·min-1, resulting in a residence time of 2 h. The obtained reaction mixture is collected into a nitrogen-purged vial containing $NaHCO_3$ (100 mg, 1.2 mmol, 6 equiv.) and degassed CH_3CN (1 mL). The vial is stirred at rt during the collection of the outflow from the Signify Eagle reactor. After all the outflow is collected, the vial is cooled to 0 °C and *N*-chlorosuccinimide (40 mg, 0.3 mmol, 1.5 equiv.) is added. The reaction mixture is allowed to warm up to rt and stirred for another 30 min. The mixture is transferred to a separatory funnel, diluted with brine (20 mL) and extracted with DCM (3x20 mL). The combined organic layers are dried over $MgSO_4$ and evaporated in vacuo. The flask was purged with nitrogen and the residue was dissolved in DCM (5 mL) and (+)-Dehydroabietylamine (57.1 mg, 0.2 mmol, 1 equiv.) was added, followed by Et_3N (56 uL, 0.4 mmol, 2 equiv.). The mixture was stirred at rt for 24 h, after which it was transferred to a separatory funnel, diluted with 0.1 M HCl (20 mL) and extracted with DCM (3x20 mL). The combined organic layers are dried over $MgSO_4$ and evaporated in vacuo. The crude mixture was purified by flash column chromatography (100% *n*-pentane to 40% AcOEt in *n*-pentane) to afford product **36** (20.5 mg, 27%) as a colorless oil.

^{1}H NMR (400 MHz, $CDCl_3$) δ 7.16 (d, J = 8.2 Hz, 1H), 7.00 (d, J = 2.0 Hz, 1H), 6.89 (d, J = 2.0 Hz, 1H), 4.35 (t, J = 6.9 Hz, 1H), 3.05 – 2.95 (m, 3H), 2.94 – 2.77 (m, 4H), 2.34 – 2.24 (m, 1H), 1.80 – 1.65 (m, 4H), 1.51 (dd, J = 11.2, 3.6 Hz, 1H), 1.47 – 1.36 (m, 2H), 1.33 (m, 4H), 1.23 (s, 3H), 1.22 (s, 6H), 0.96 (s, 3H).

^{13}C NMR (101 MHz, $CDCl_3$) δ 146.9, 145.7, 134.5, 126.8, 124.1, 123.8, 53.9, 46.7, 44.9, 38.2, 37.4, 37.0, 35.8, 33.4, 29.8, 25.2, 24.0, 23.9, 18.8, 18.5, 18.4, 8.3.

HRMS (EI+) (m/z): $[M]^+$ calcd. for $C_{22}H_{35}NO_2S$, 377.2383; found: 377.2404.

Ethyl methyl sulfone (S5)

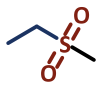

S5

To a nitrogen-purged, screw-capped vial, fitted with a rubber septum and charged with NaDT (14.65 mg, 0.006 mmol, 3 mol%) degassed CH_3CN is added (2.4 mL), followed by aqueous SO_2 (6 wt%, 0.6 mL, 0.6 mmol, 3 equiv.). The stock solution is charged in a gastight syringe, positioned in a syringe pump and combined with a stream of ethane gas (70.5 mL, 3 mmol, 15 equiv.) through a T-mixer into a filling loop, with a liquid flow rate of 0.16 mL·min-1 and a ethane gas flow rate of 4 mL·min-1. A BPR of 2.8 bar is used during the loop filling. Next, the filling loop is connected to the reactor, the system is pressurized to 52 bar and the reaction mixture is pumped over the Signify Eagle reactor (365 nm, 144 W output power, FEP capillary: 0.5 mm ID, 1.5 mL) at a flow rate of 0.0125 mL·min-1, resulting in a residence time of 2 h. The obtained reaction mixture is collected into a nitrogen-purged vial containing $NaHCO_3$ (100 mg, 1.2 mmol, 6 equiv.) and degassed CH_3CN (1 mL). The vial is stirred at rt during the collection of the outflow from the Signify Eagle reactor. After all the outflow is collected, methyl iodide (28.4 mg, 0.2 mmol, 1 equiv.) is added and the mixture is stirred overnight. Then, the reaction mixture is transferred to a separatory funnel, diluted with water (20 mL) and extracted with DCM (3x20 mL). The combined organic layers are dried over $MgSO_4$ and evaporated in vacuo. The crude mixture was purified by flash column chromatography (100% *n*-pentane to 20% AcOEt in *n*-pentane) to afford product **S5** (6.5 mg, 30%) as a colorless oil.

^{1}H NMR (400 MHz, $CDCl_3$) δ 3.04 (q, J = 7.5 Hz, 2H), 2.89 (s, 3H), 1.43 (t, J = 7.5 Hz, 3H).

^{13}C NMR (101 MHz, $CDCl_3$) δ 49.2, 39.7, 7.2.

GCMS (EI+) (m/z): $[M]^+$ calcd. for $C_3H_8O_2S$, 108.0; found: 108.0.

8. NMR Spectra

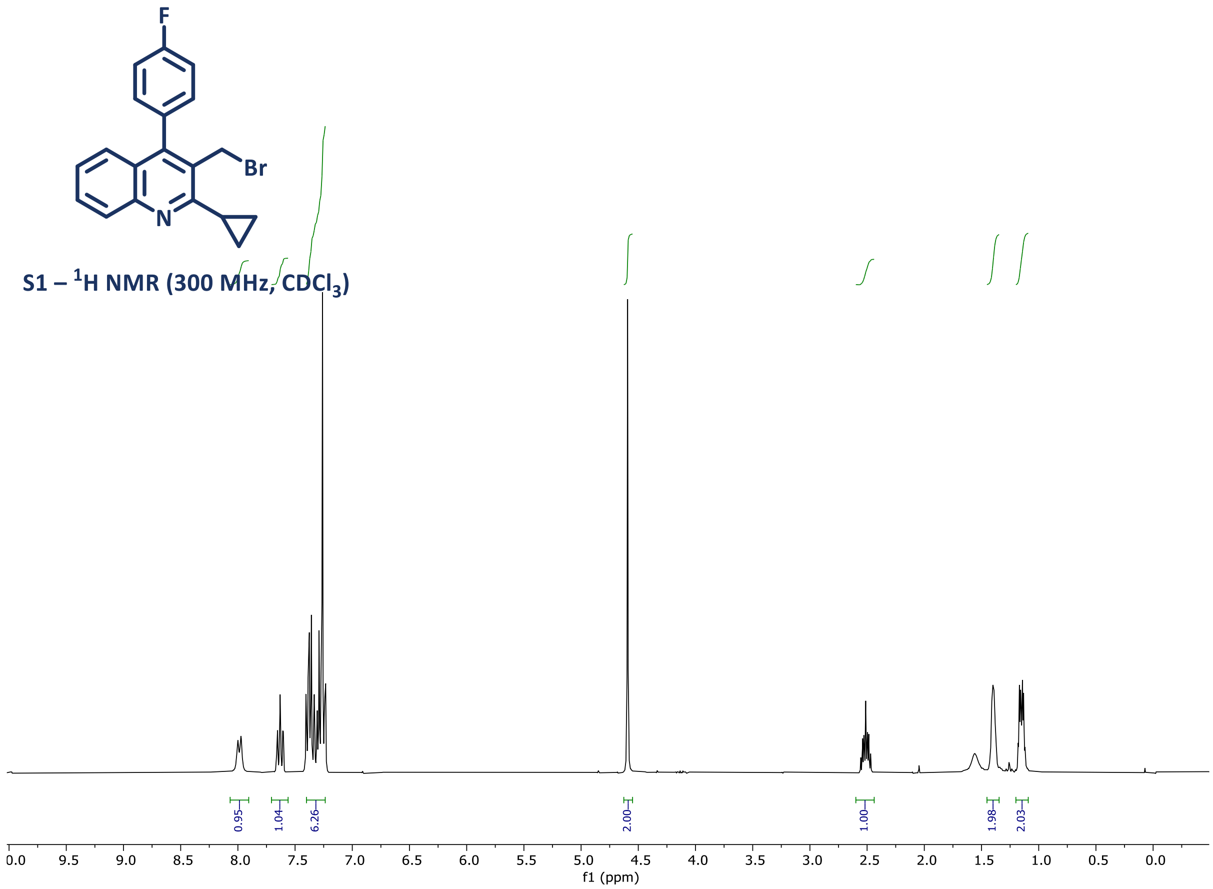

S1 – ^{1}H NMR (300 MHz, $CDCl_3$)

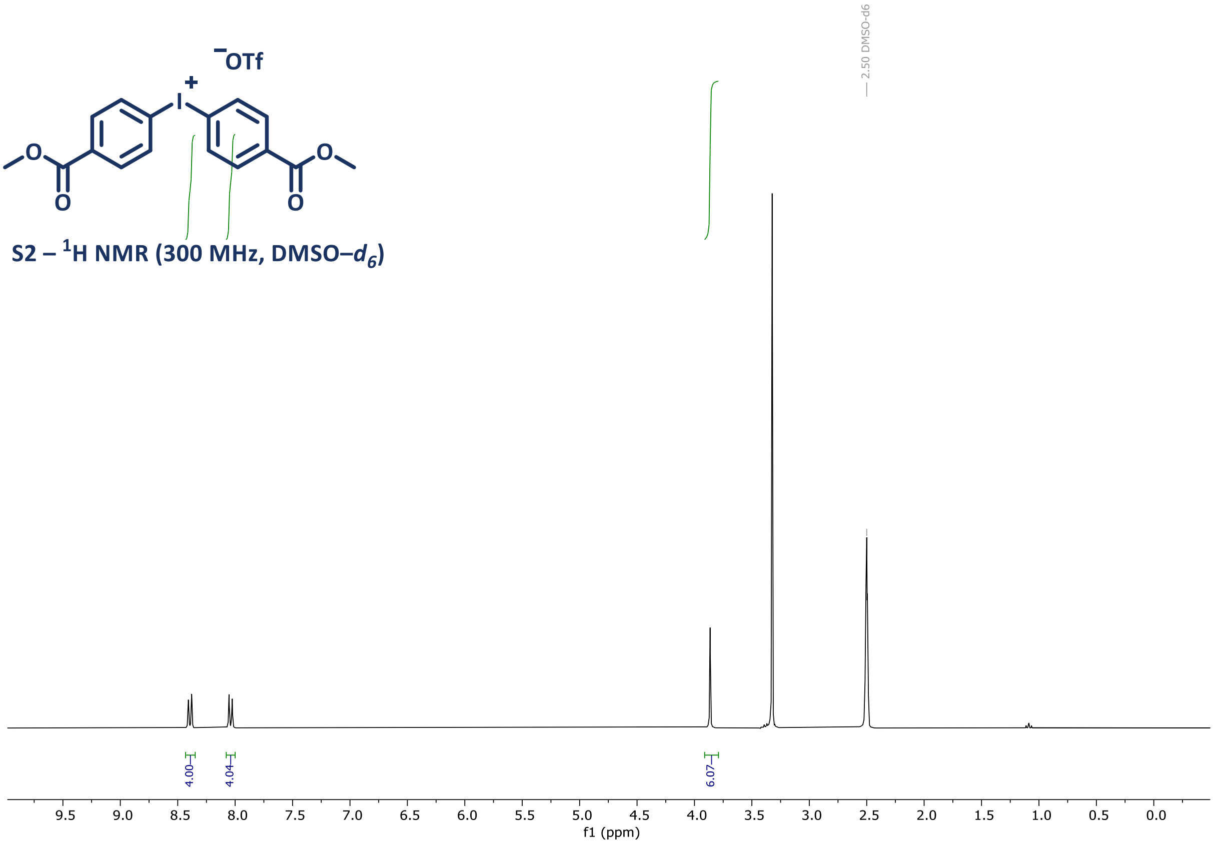

S2 – ^{1}H NMR (300 MHz, DMSO–d_6)

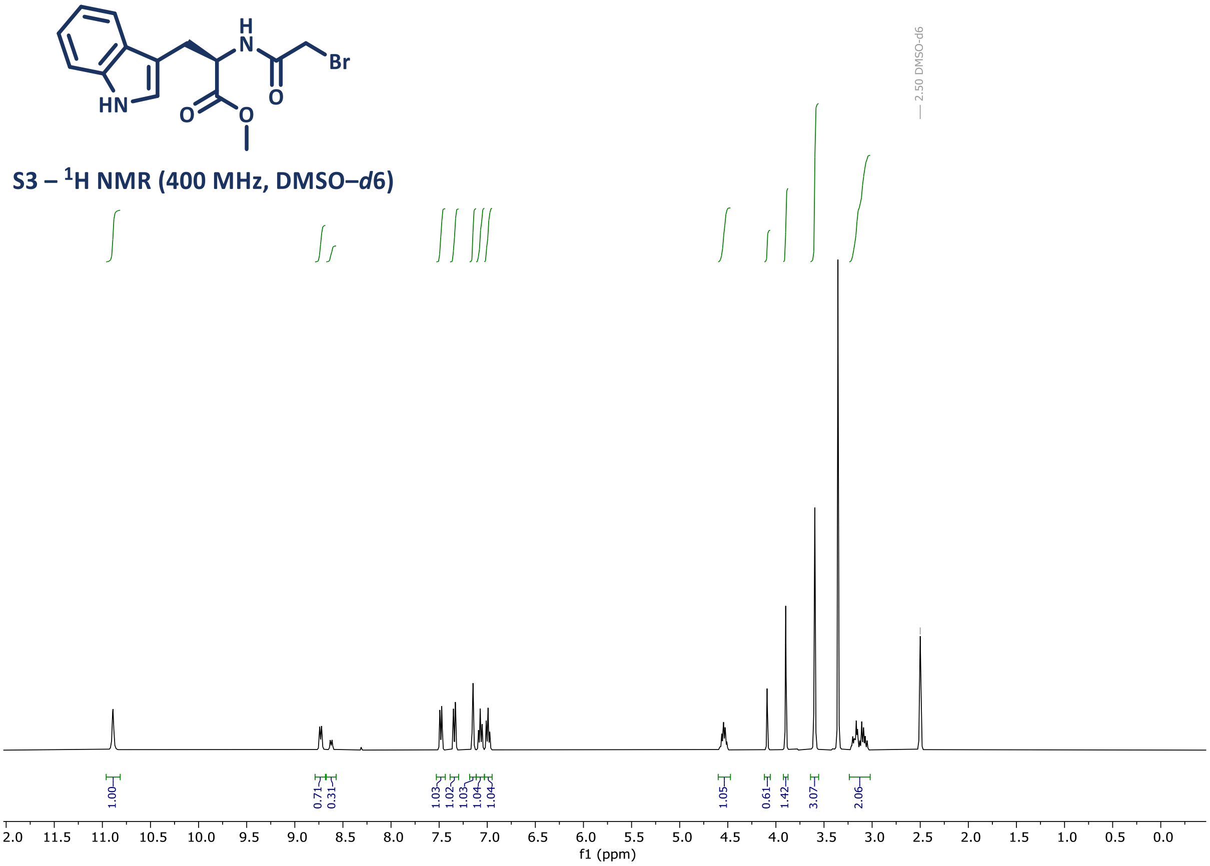

S3 – ^{1}H NMR (400 MHz, DMSO–*d6*)

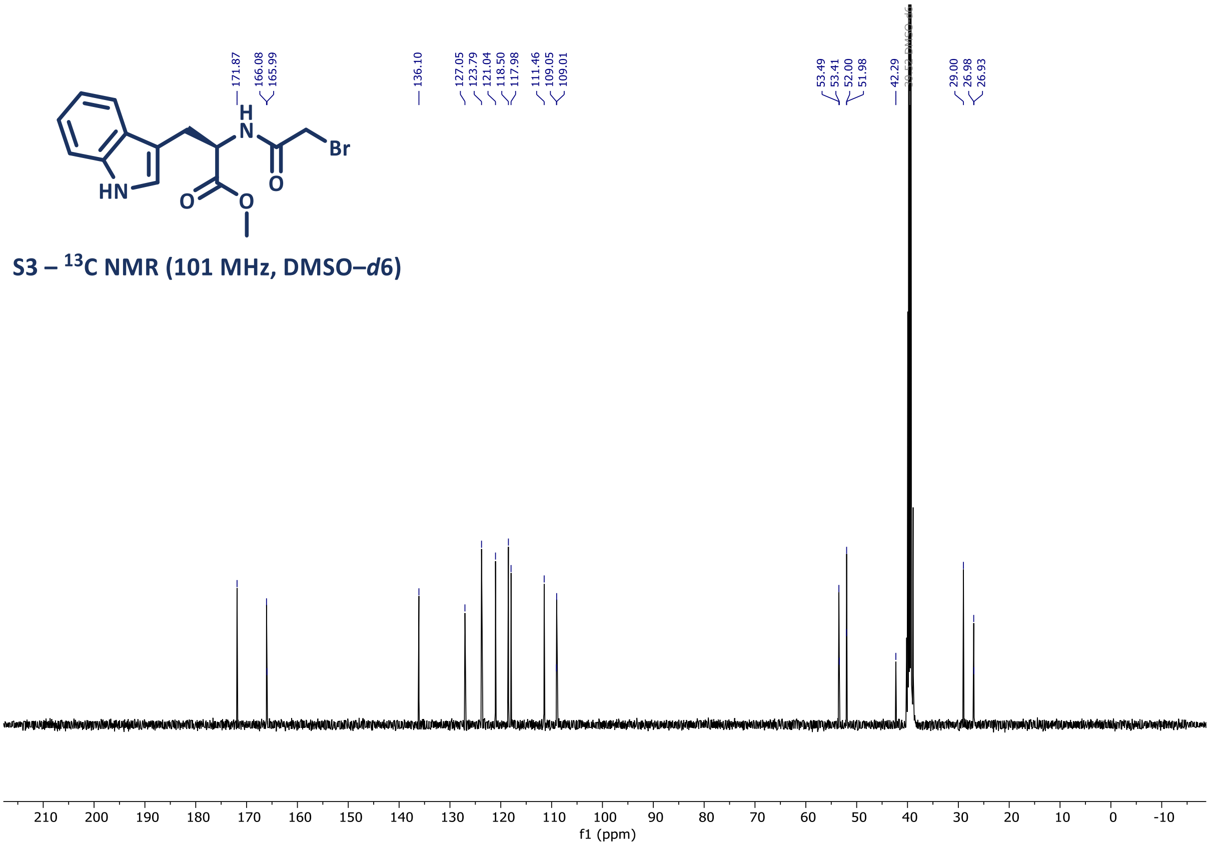

S3 – ^{13}C NMR (101 MHz, DMSO–*d6*)

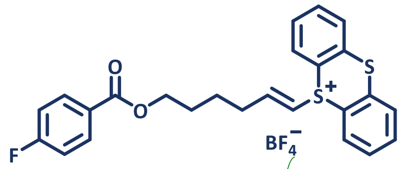

S4 – ^{1}H NMR (400 MHz, $CDCl_3$)

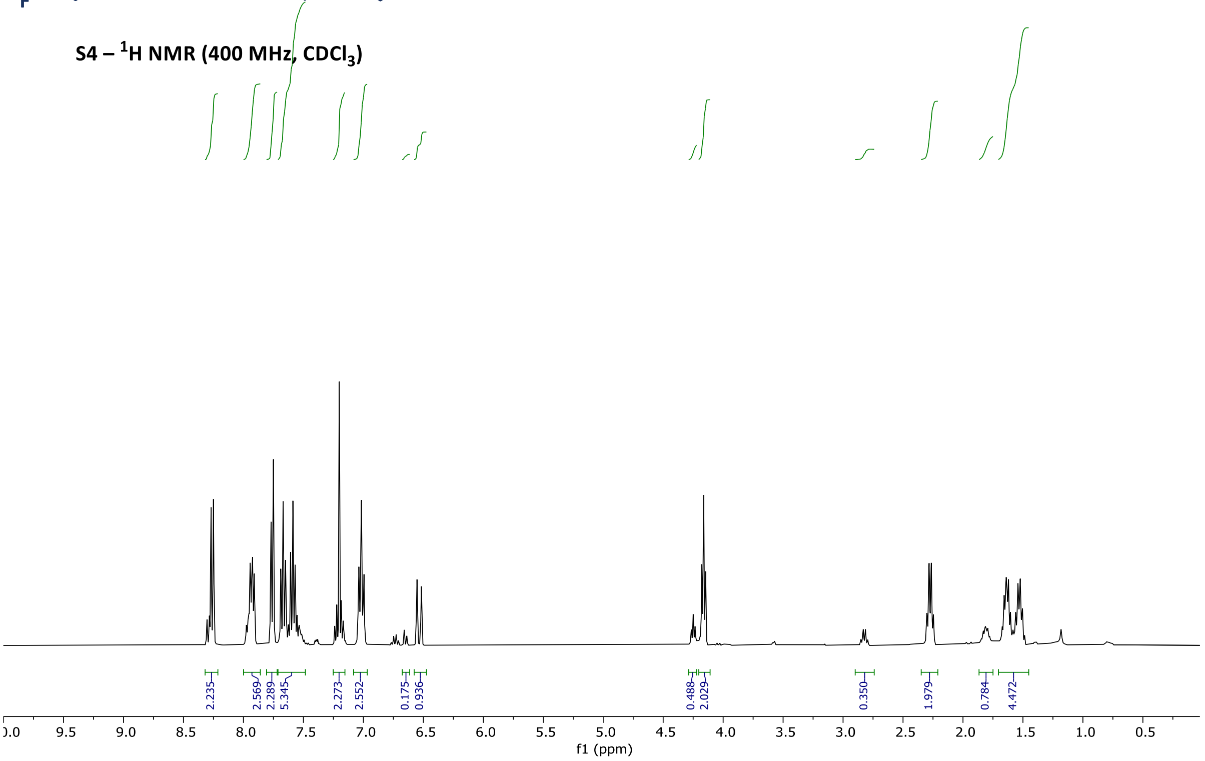

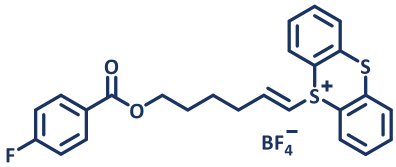

S4 – ^{13}C NMR (101 MHz, $CDCl_3$)

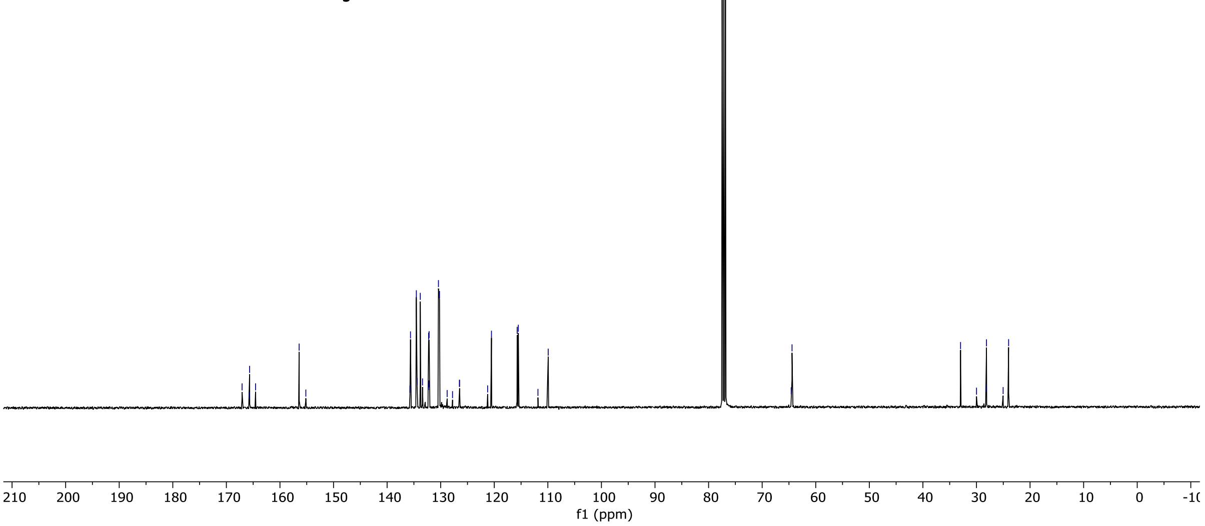

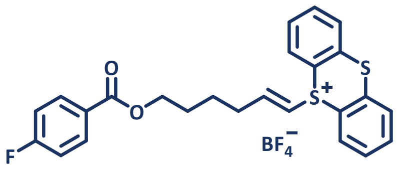

S4 – ^{19}F NMR (282 MHz, $CDCl_3$)

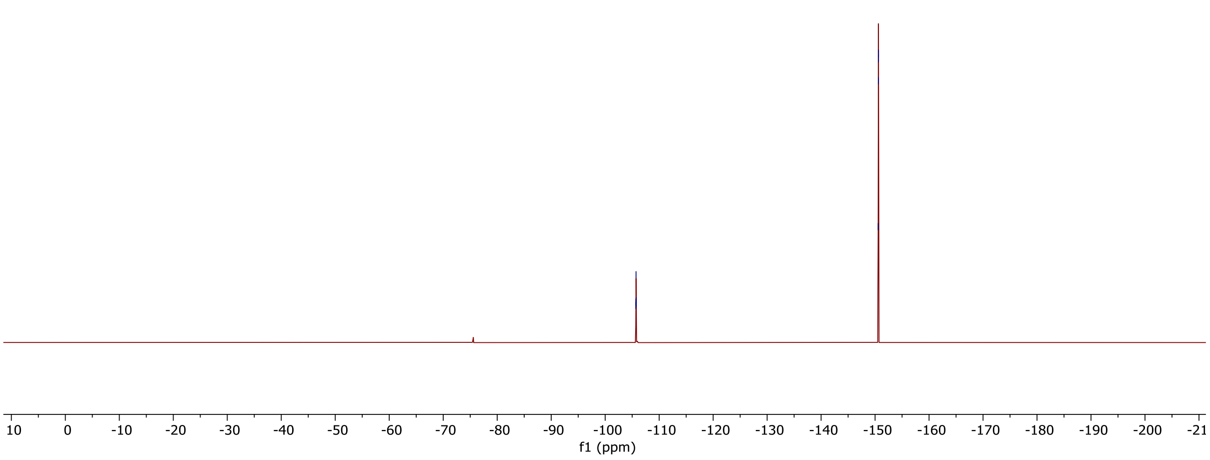

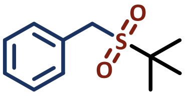

2a – ^{1}H NMR (400 MHz, $CDCl_3$)

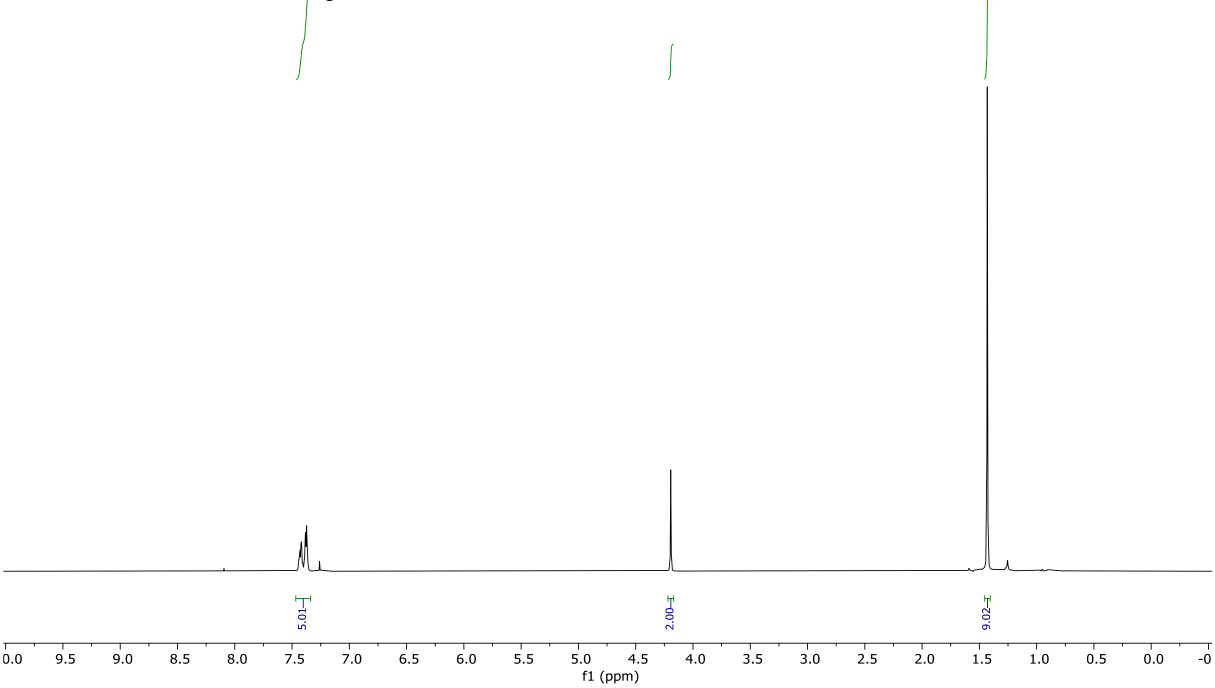

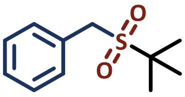

2a – ^{13}C NMR (101 MHz, $CDCl_3$)

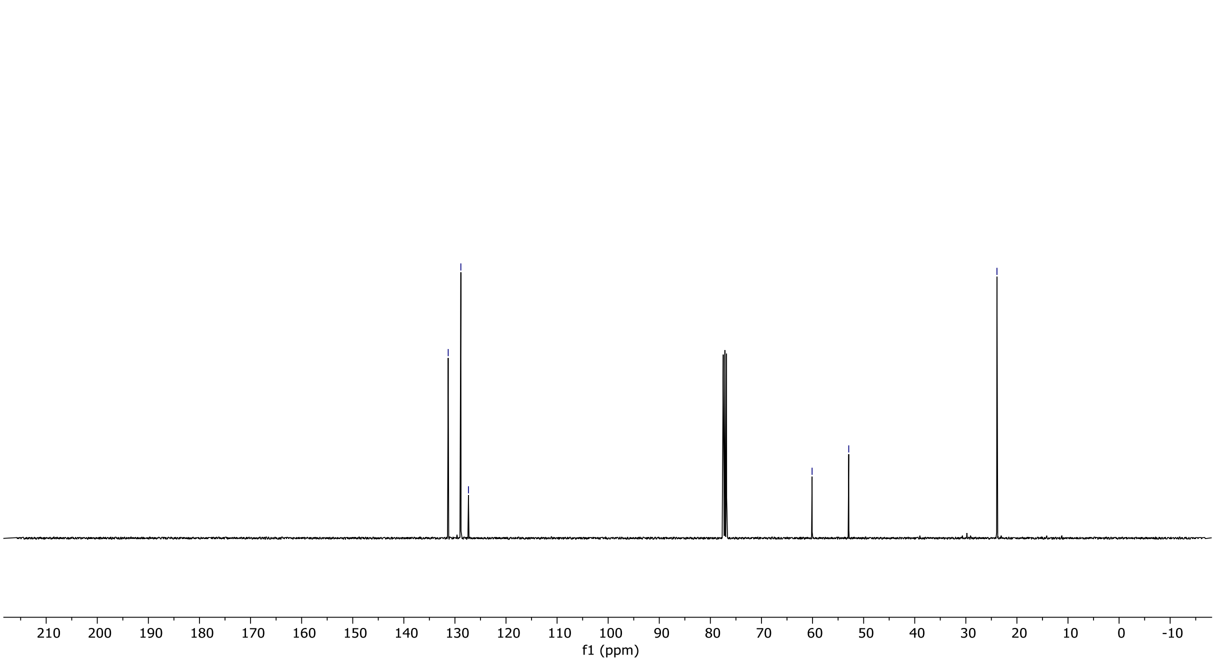

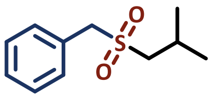

2b – ^{1}H NMR (300 MHz, $CDCl_3$)

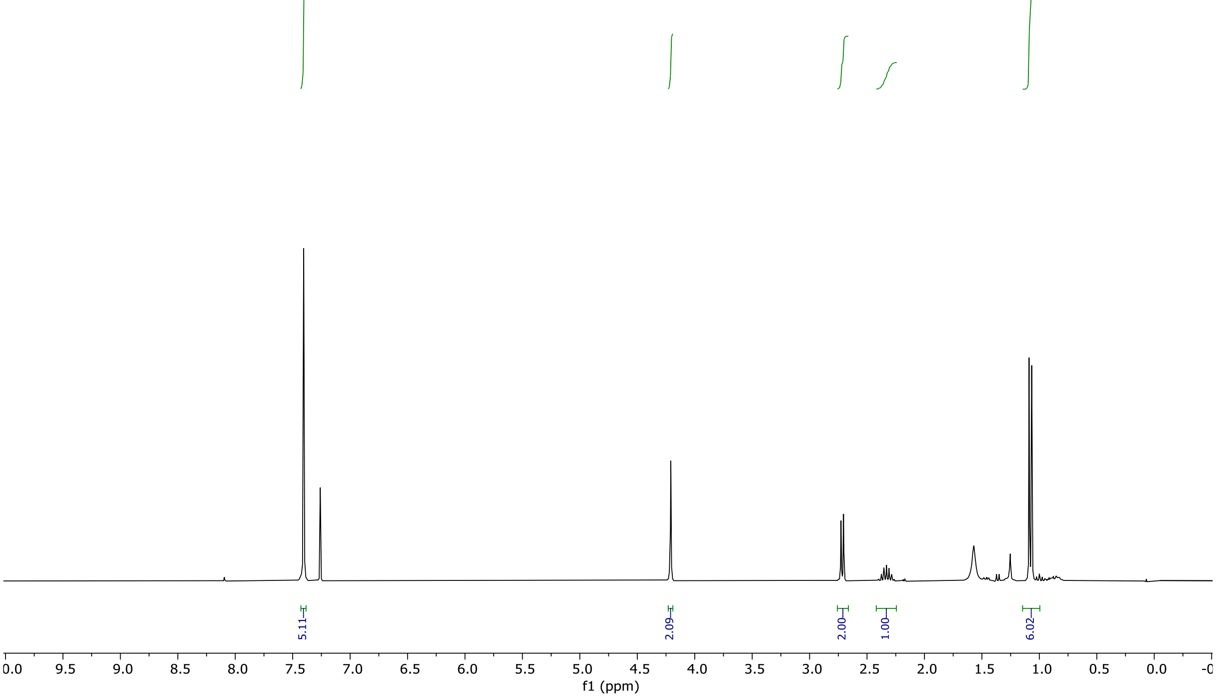

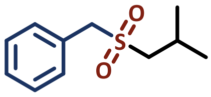

2b – ^{13}C NMR (75 MHz, $CDCl_3$)

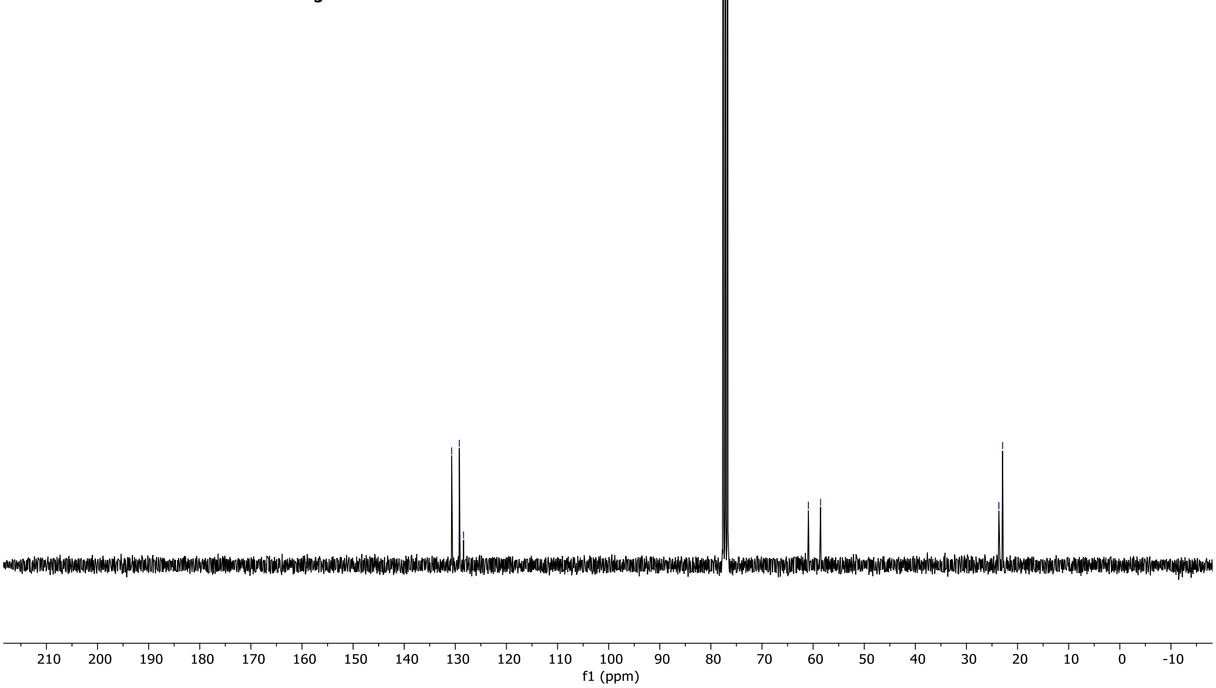

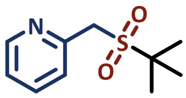

3a – ^{1}H NMR (300 MHz, $CDCl_3$)

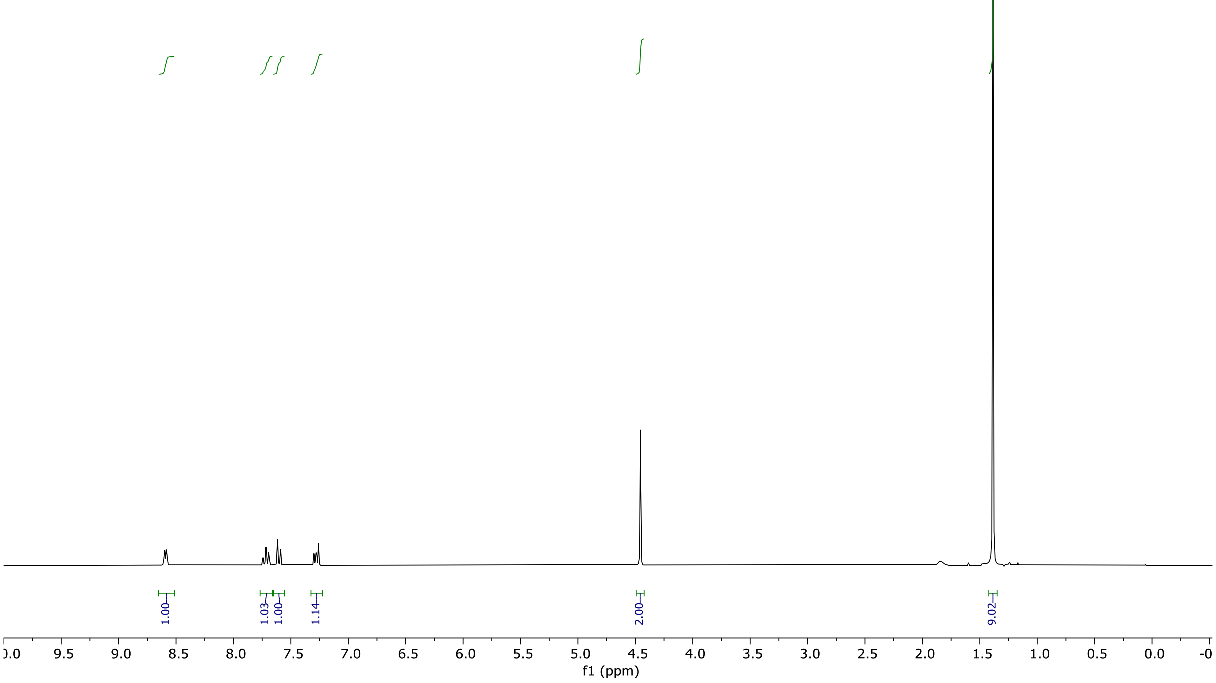

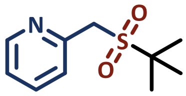

3a – ^{13}C NMR (75 MHz, $CDCl_3$)

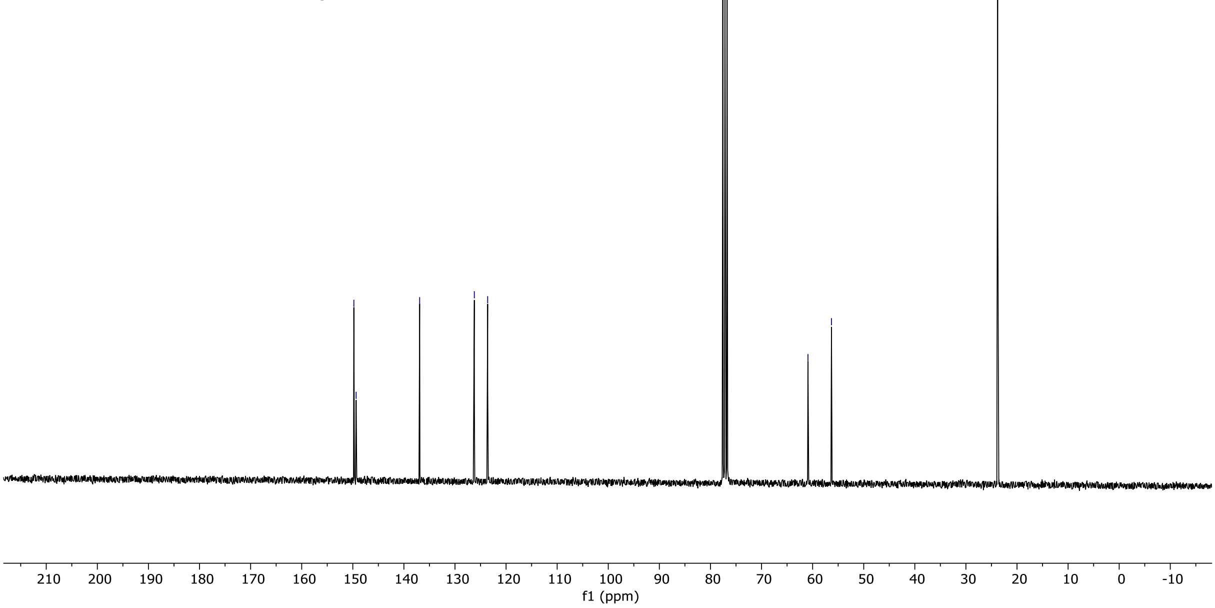

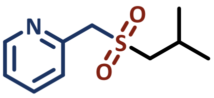

3b – ^{1}H NMR (300 MHz, $CDCl_3$)

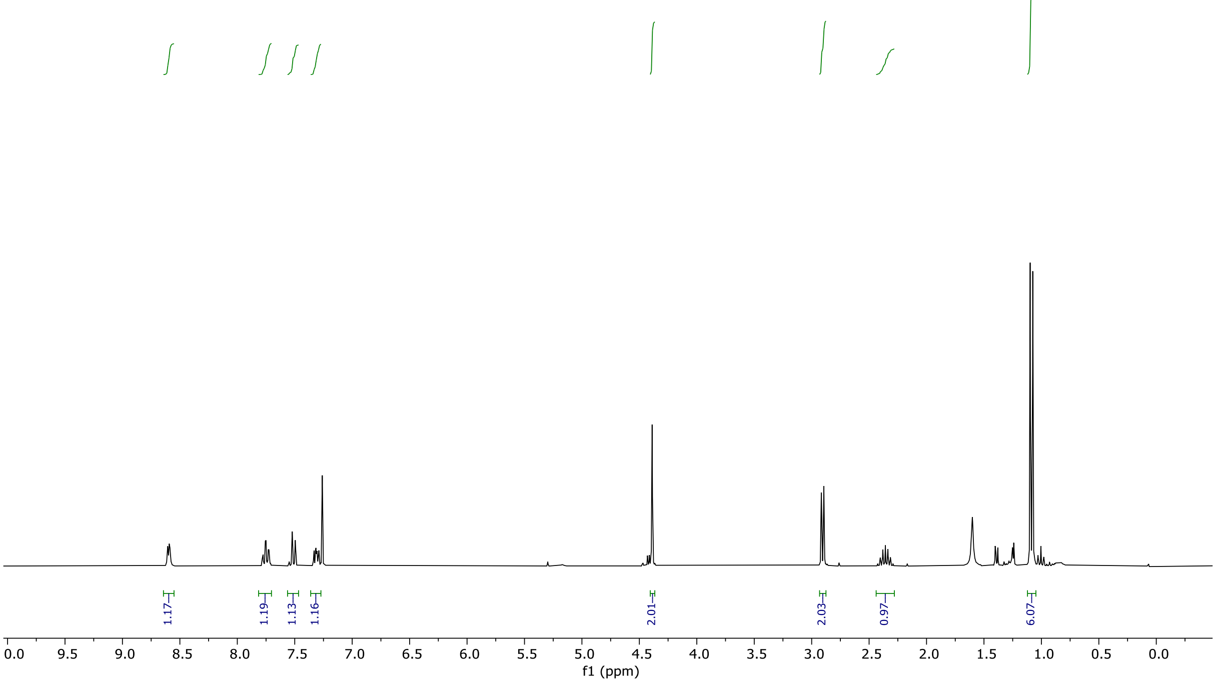

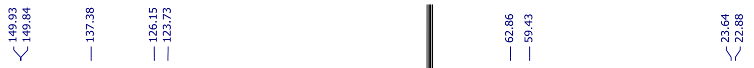

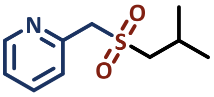

3b – ^{13}C NMR (75 MHz, $CDCl_3$)

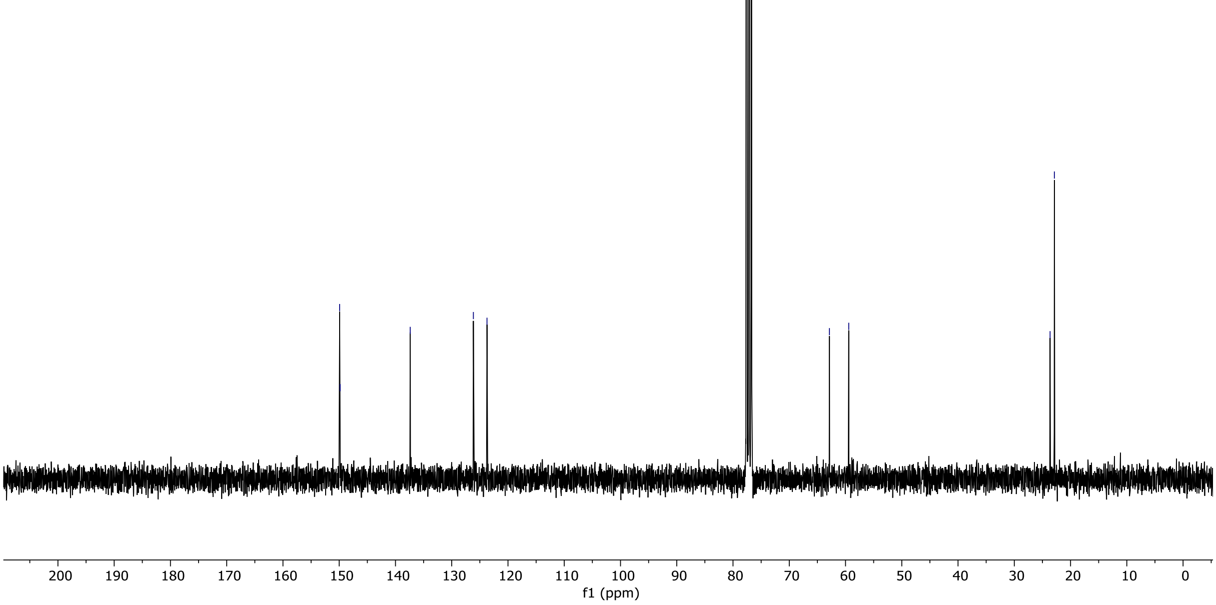

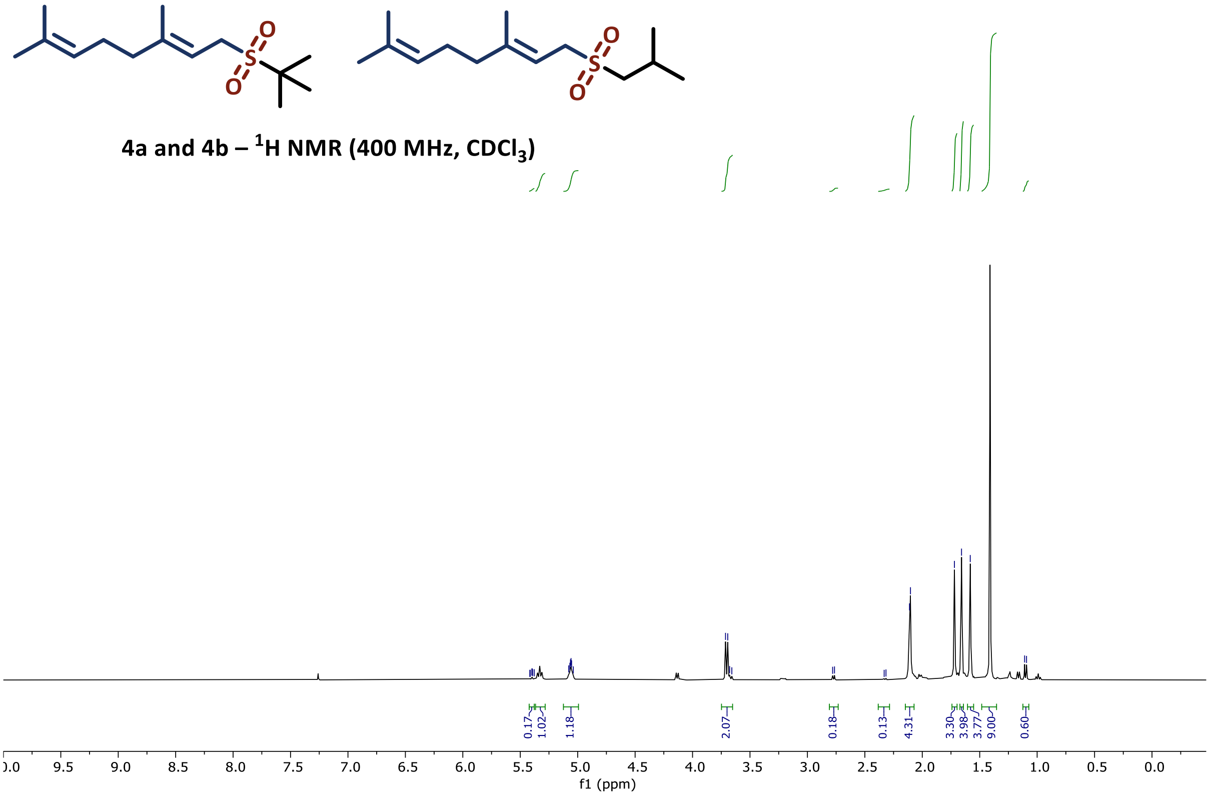

4a and 4b – ^{1}H NMR (400 MHz, $CDCl_3$)

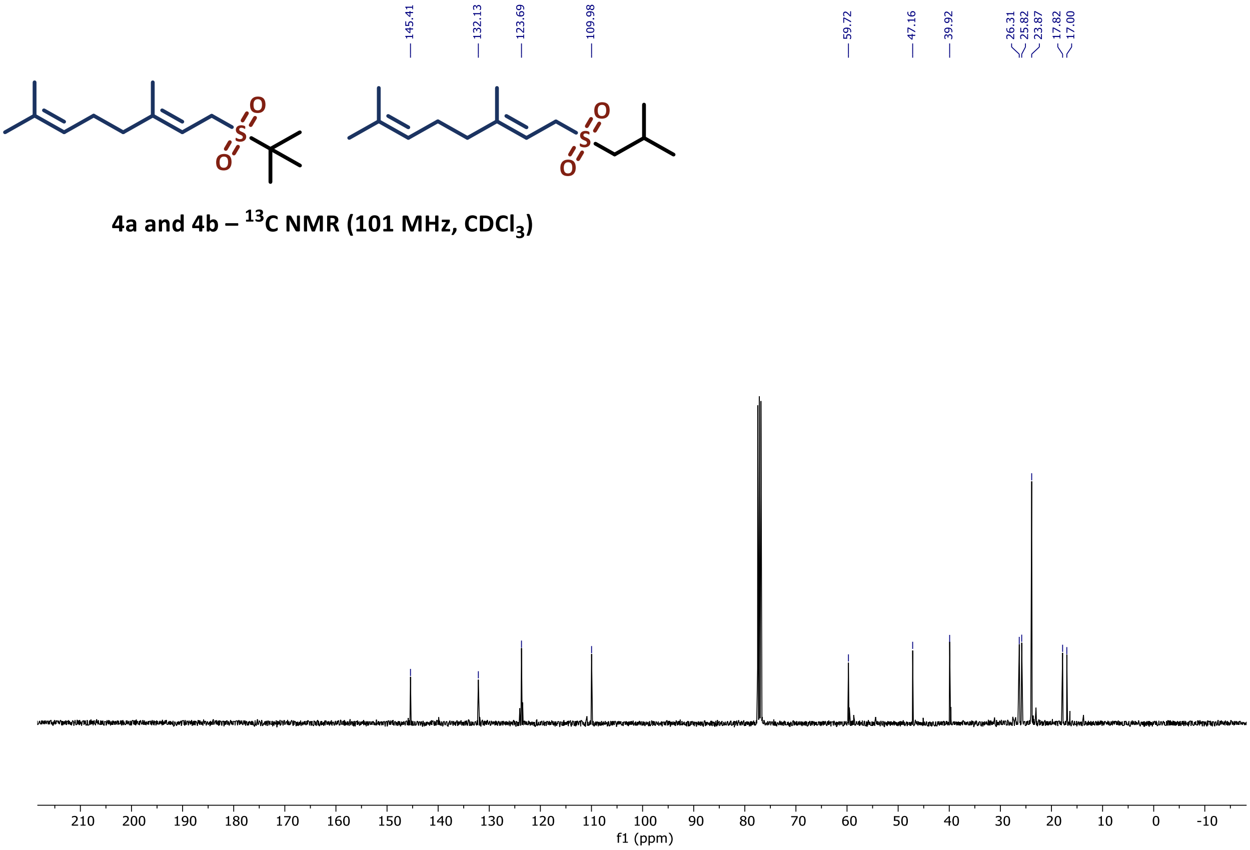

4a and 4b – ^{13}C NMR (101 MHz, $CDCl_3$)

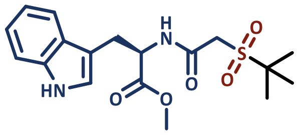

5a – ^{1}H NMR (400 MHz, CDCl$_3$)

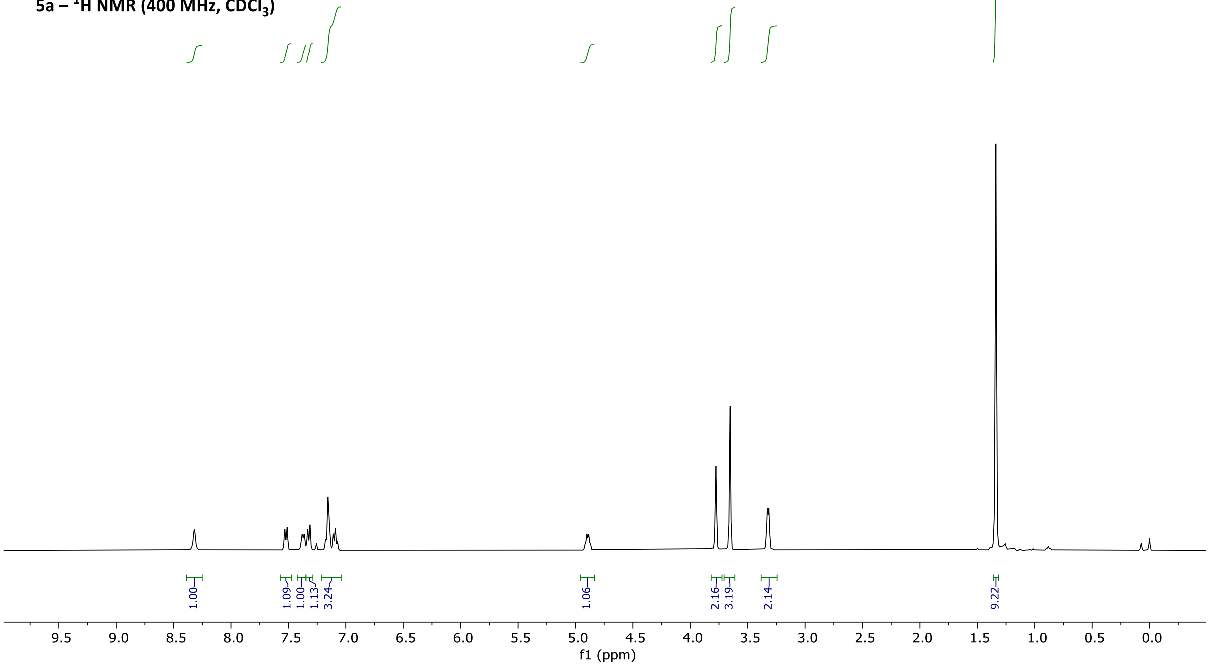

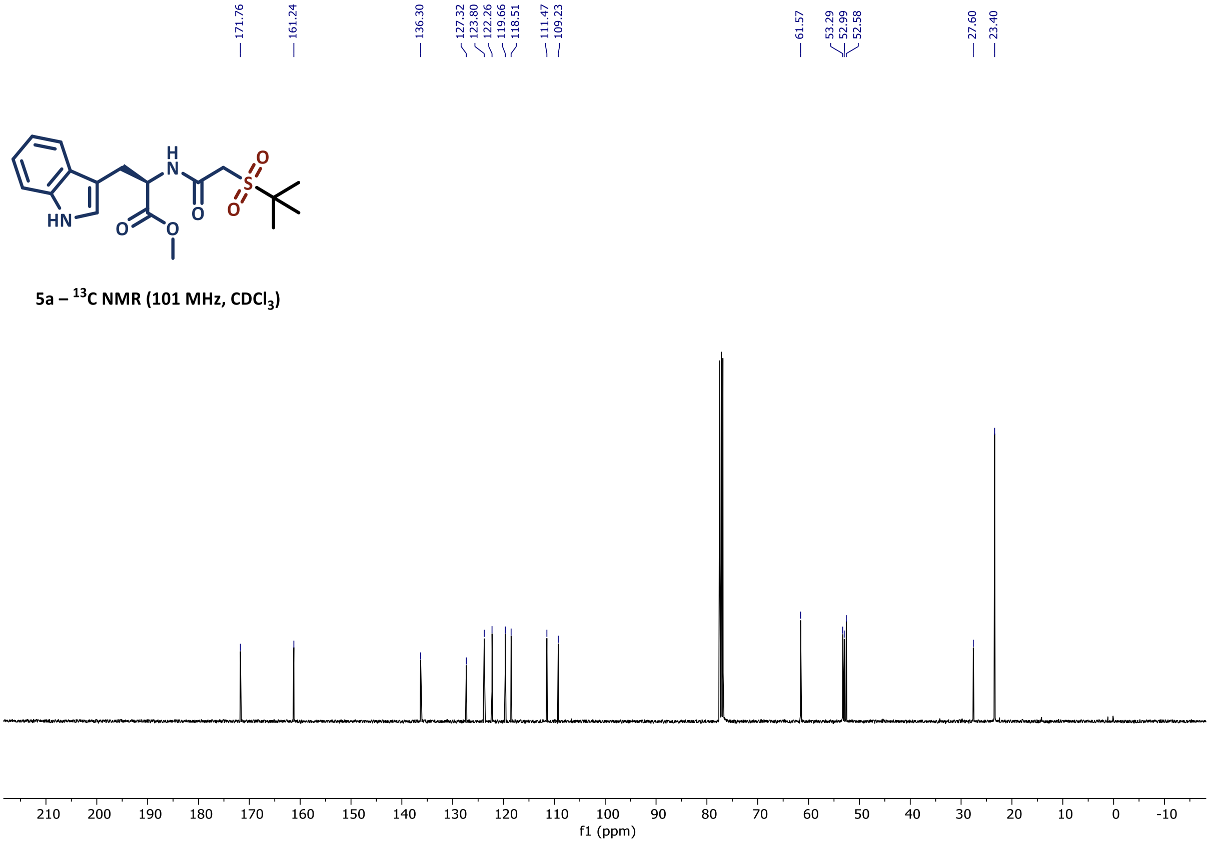

5a – ^{13}C NMR (101 MHz, CDCl$_3$)

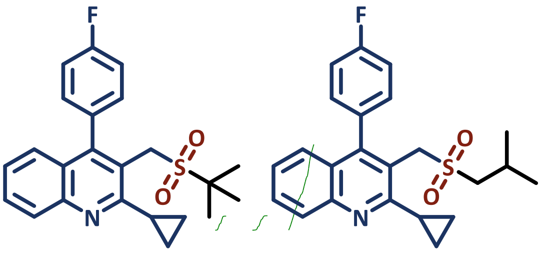

6a and 6b – ^{1}H NMR (300 MHz, $CDCl_3$)

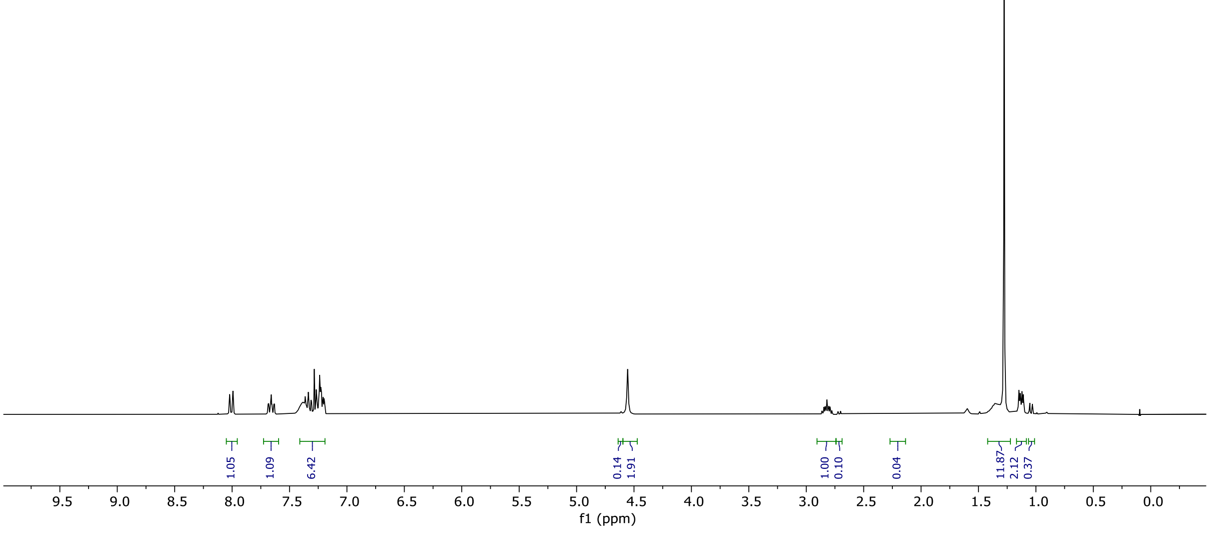

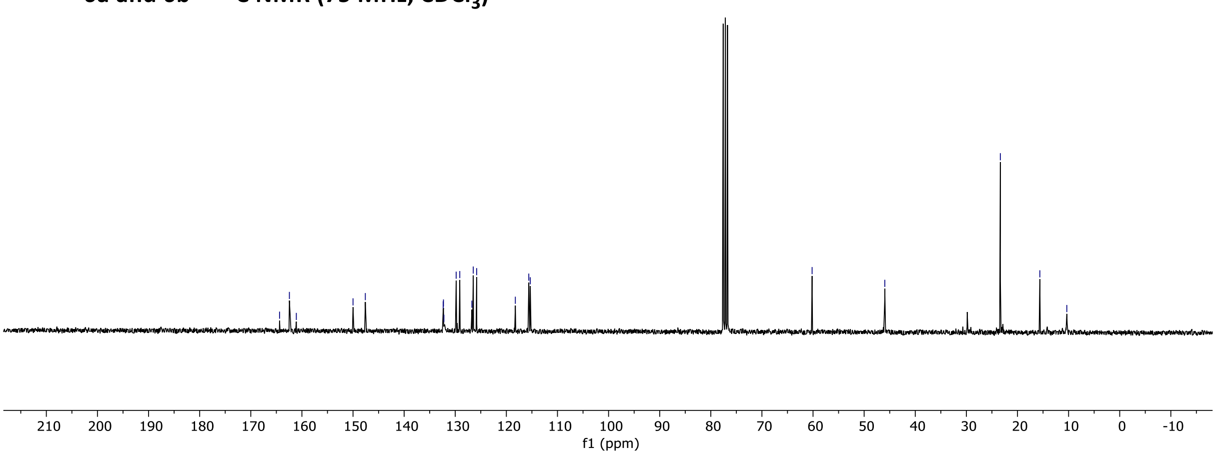

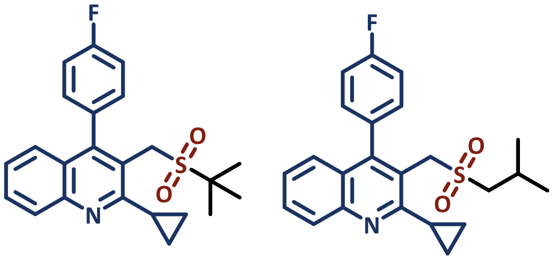

6a and 6b – ^{13}C NMR (75 MHz, $CDCl_3$)

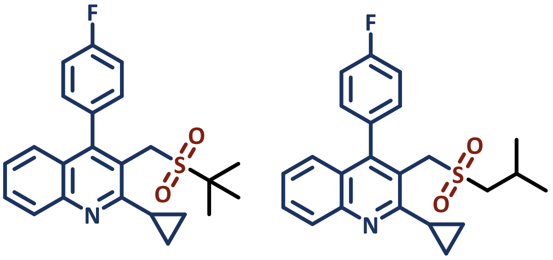

6a and 6b – ^{19}F NMR (282 MHz, $CDCl_3$)

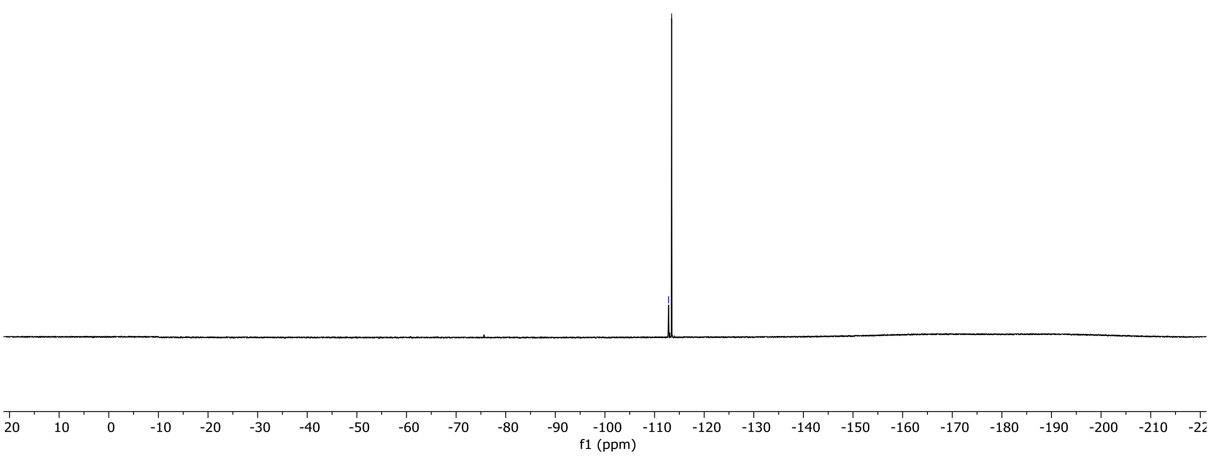

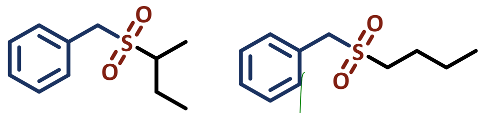

7a and 7b – ^{1}H NMR (400 MHz, $CDCl_3$)

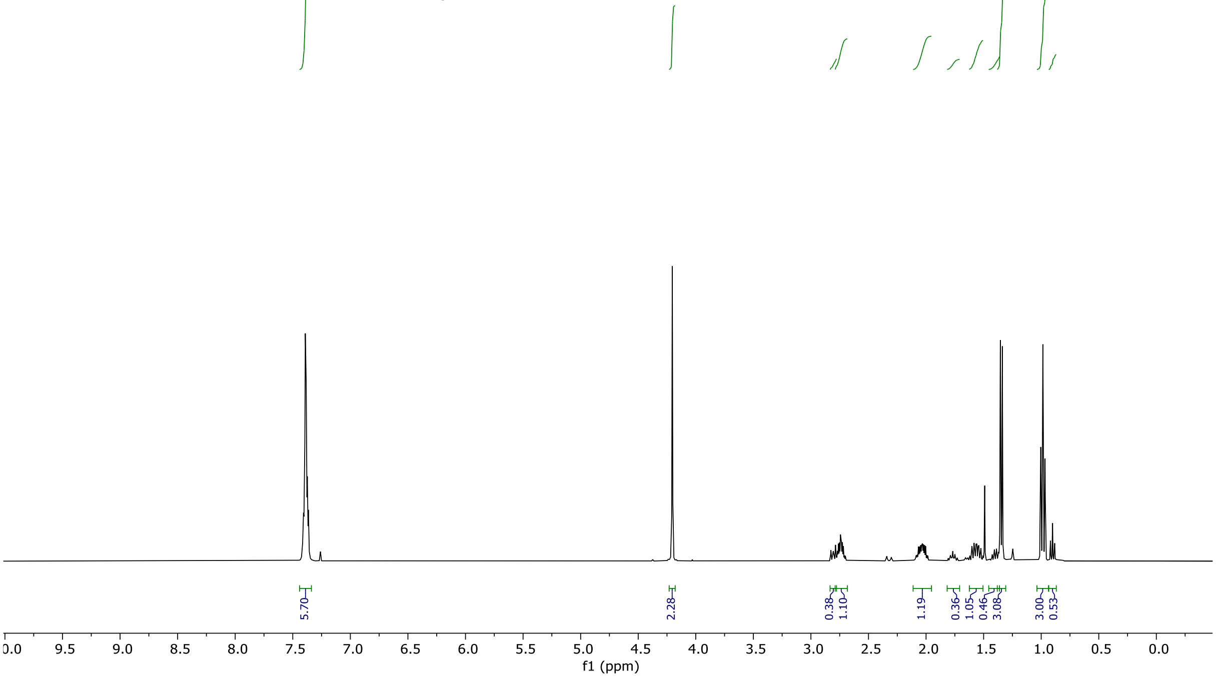

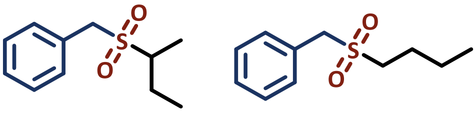

7a and 7b – ^{13}C NMR (101 MHz, $CDCl_3$)

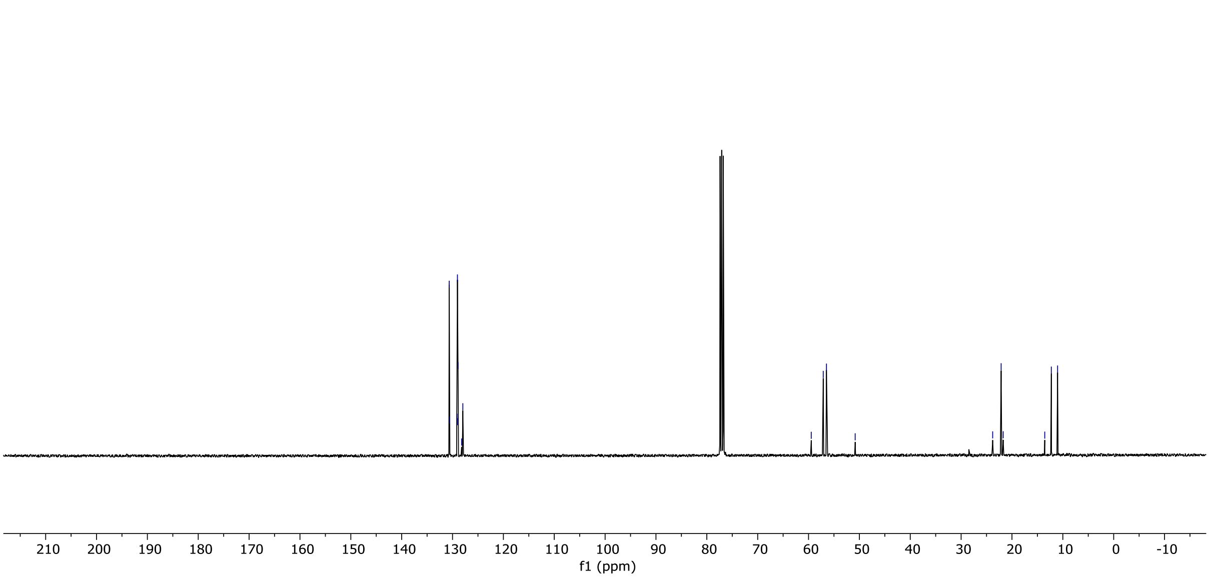

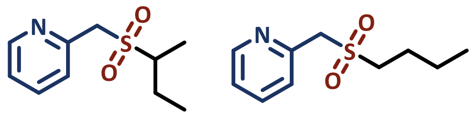

8a and 8b – ^{1}H NMR (400 MHz, $CDCl_3$)

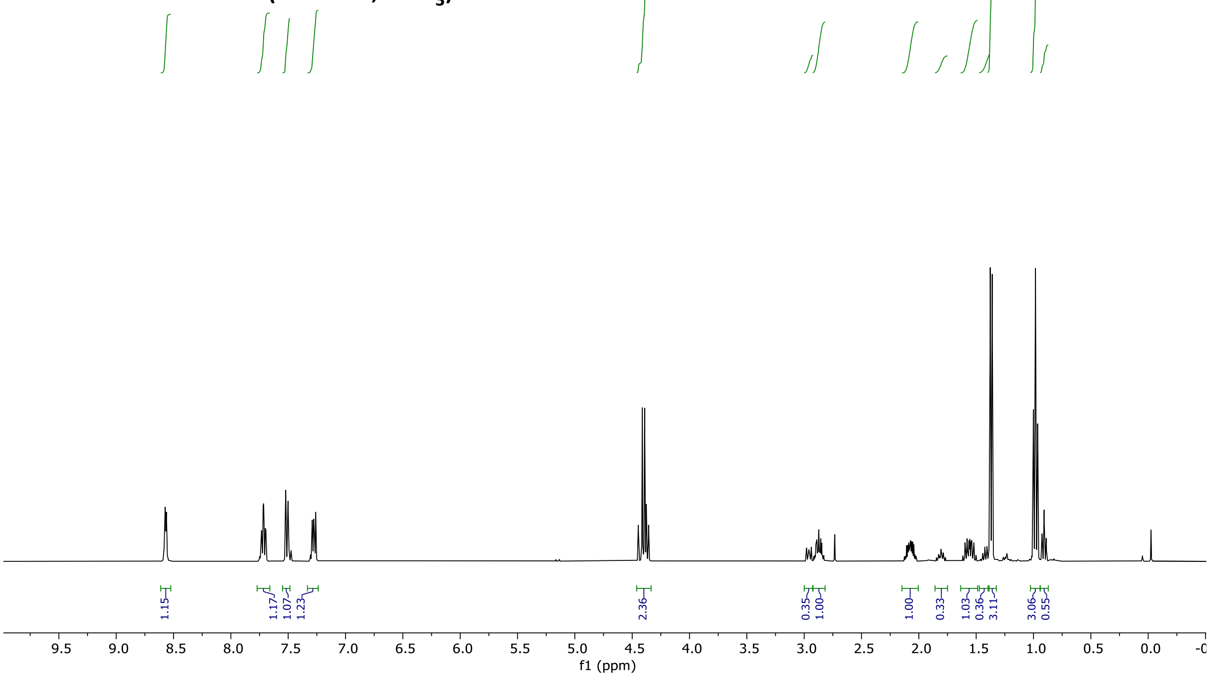

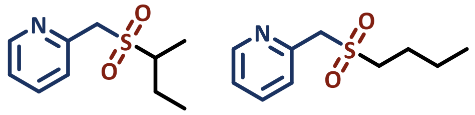

8a and 8b – ^{13}C NMR (101 MHz, $CDCl_3$)

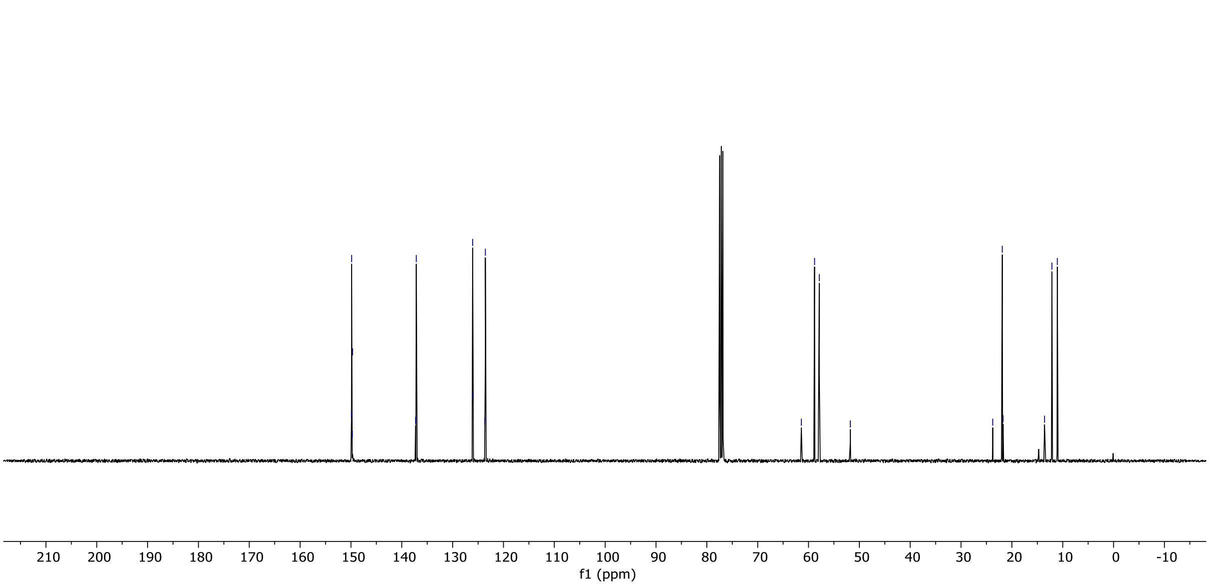

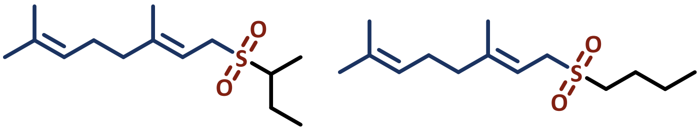

9a and 9b – ^{1}H NMR (400 MHz, $CDCl_3$)

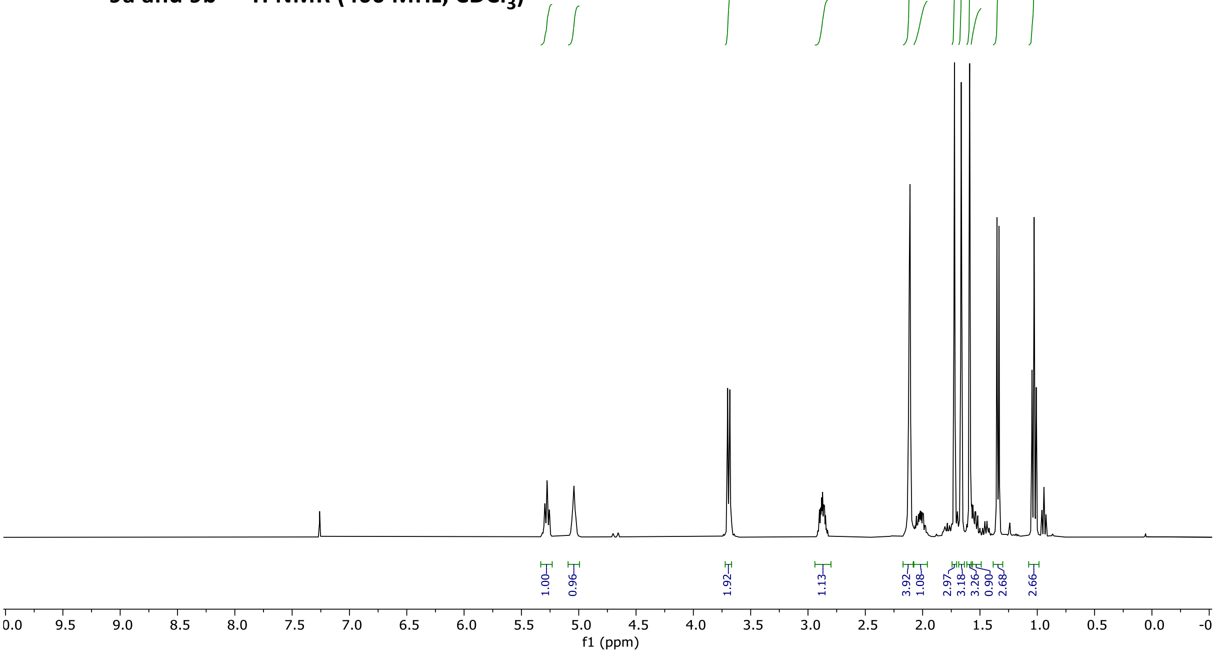

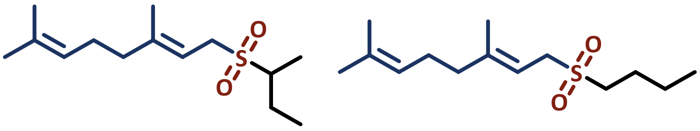

9a and 9b – ^{13}C NMR (101 MHz, $CDCl_3$)

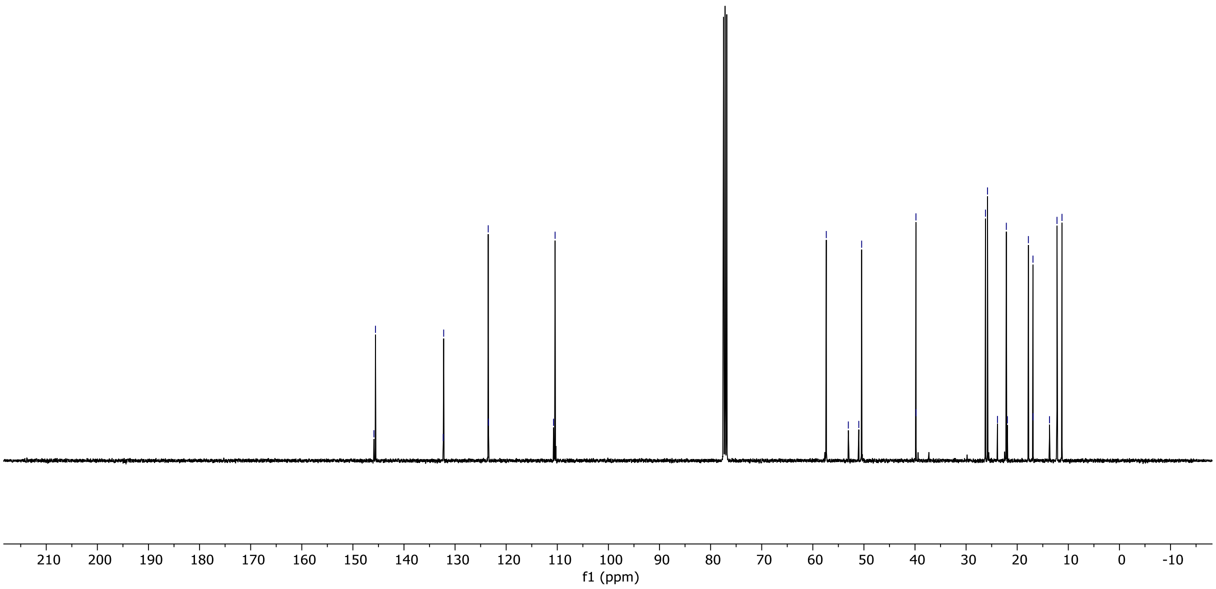

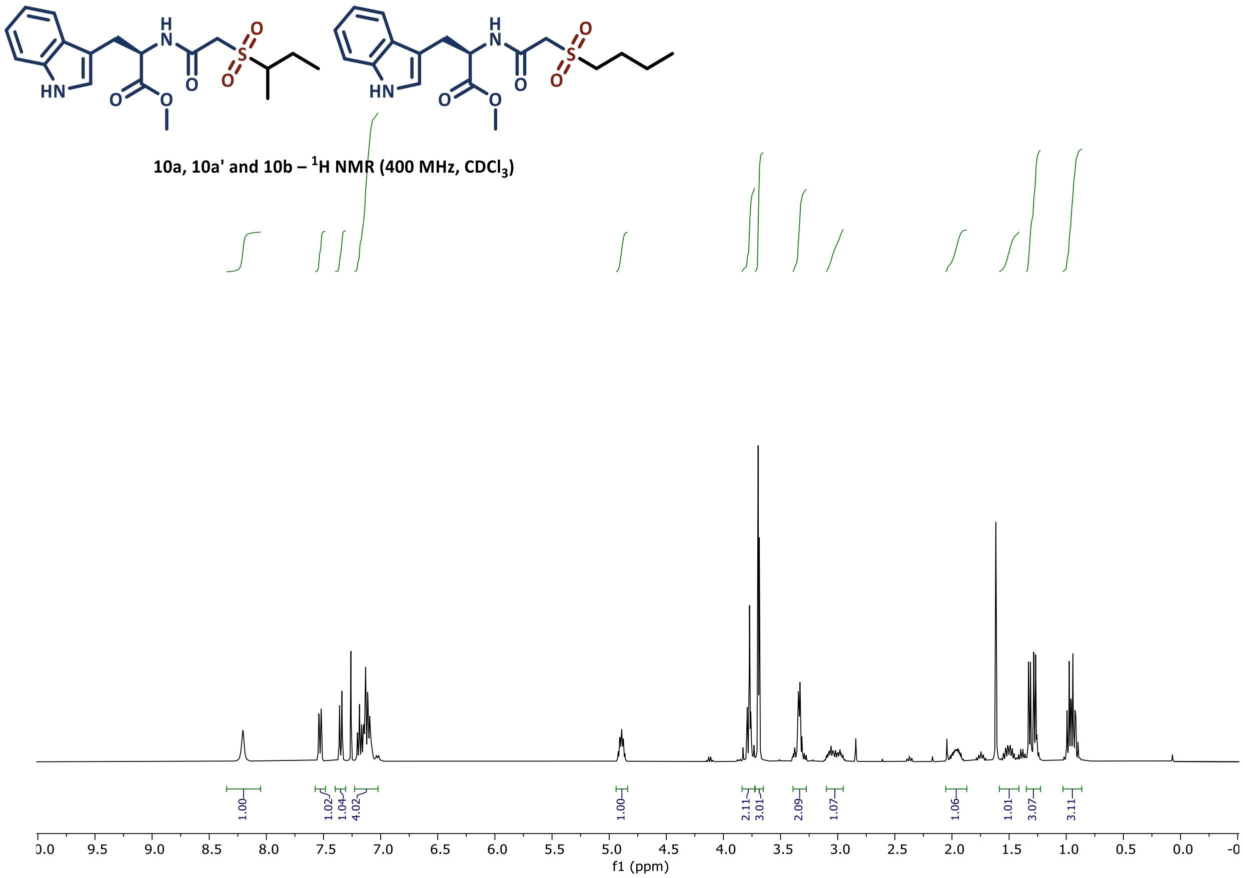

10a, 10a' and 10b – ^{1}H NMR (400 MHz, $CDCl_3$)

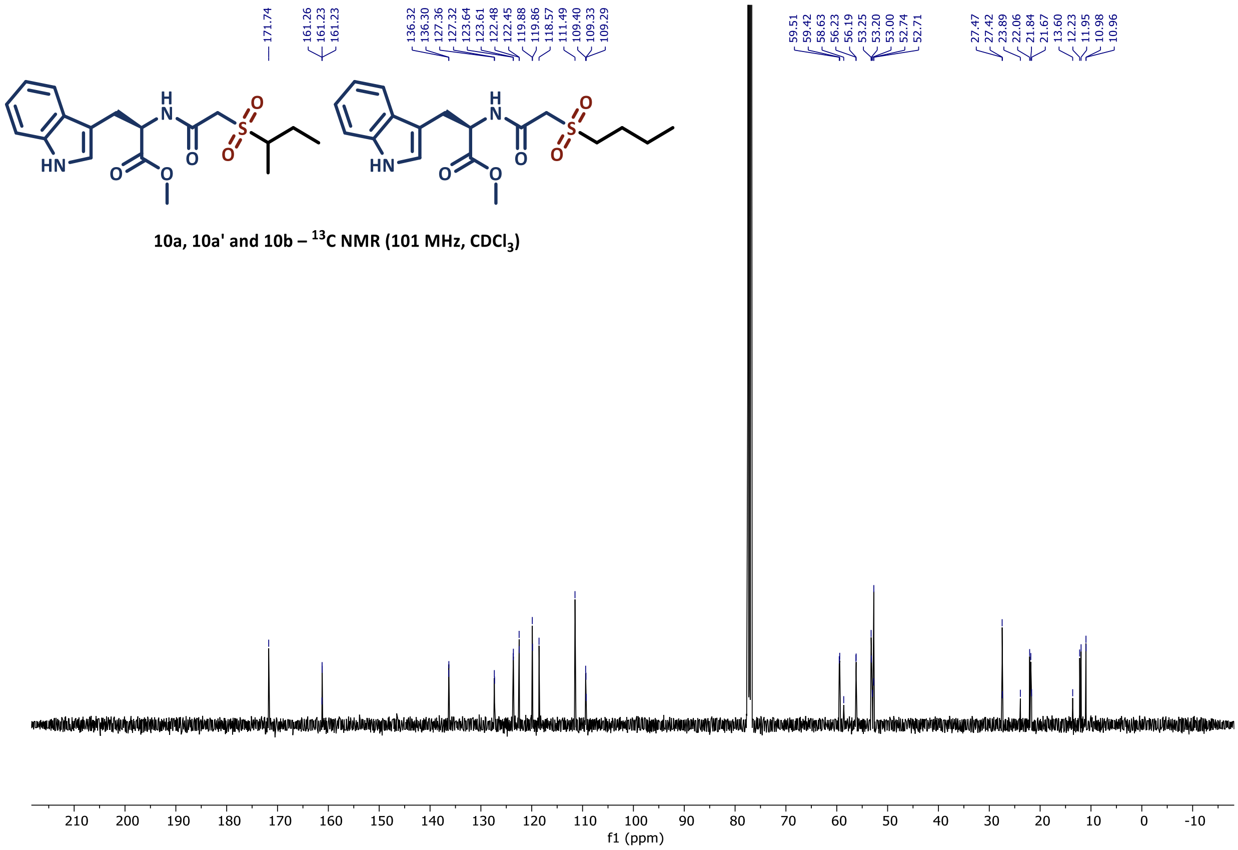

10a, 10a' and 10b – ^{13}C NMR (101 MHz, $CDCl_3$)

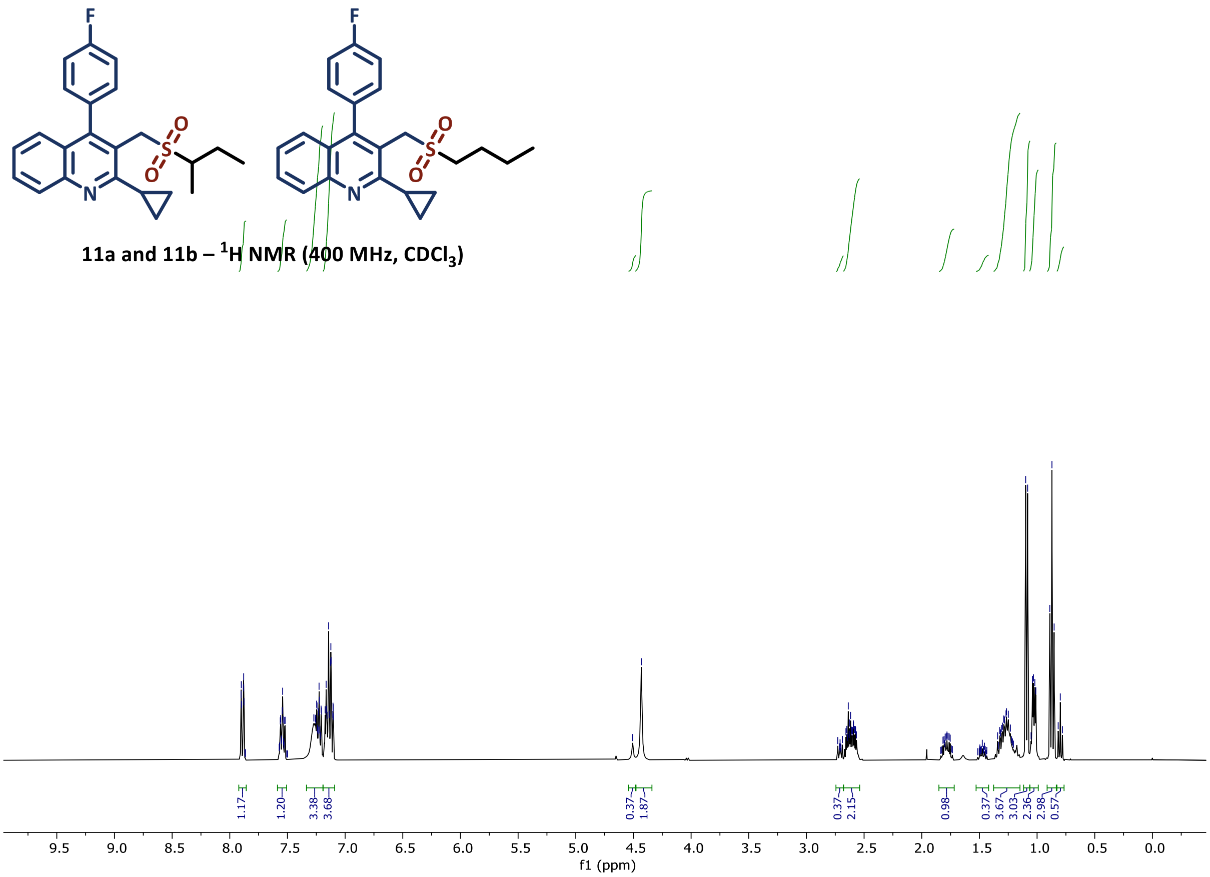

11a and 11b – ^{1}H NMR (400 MHz, $CDCl_3$)

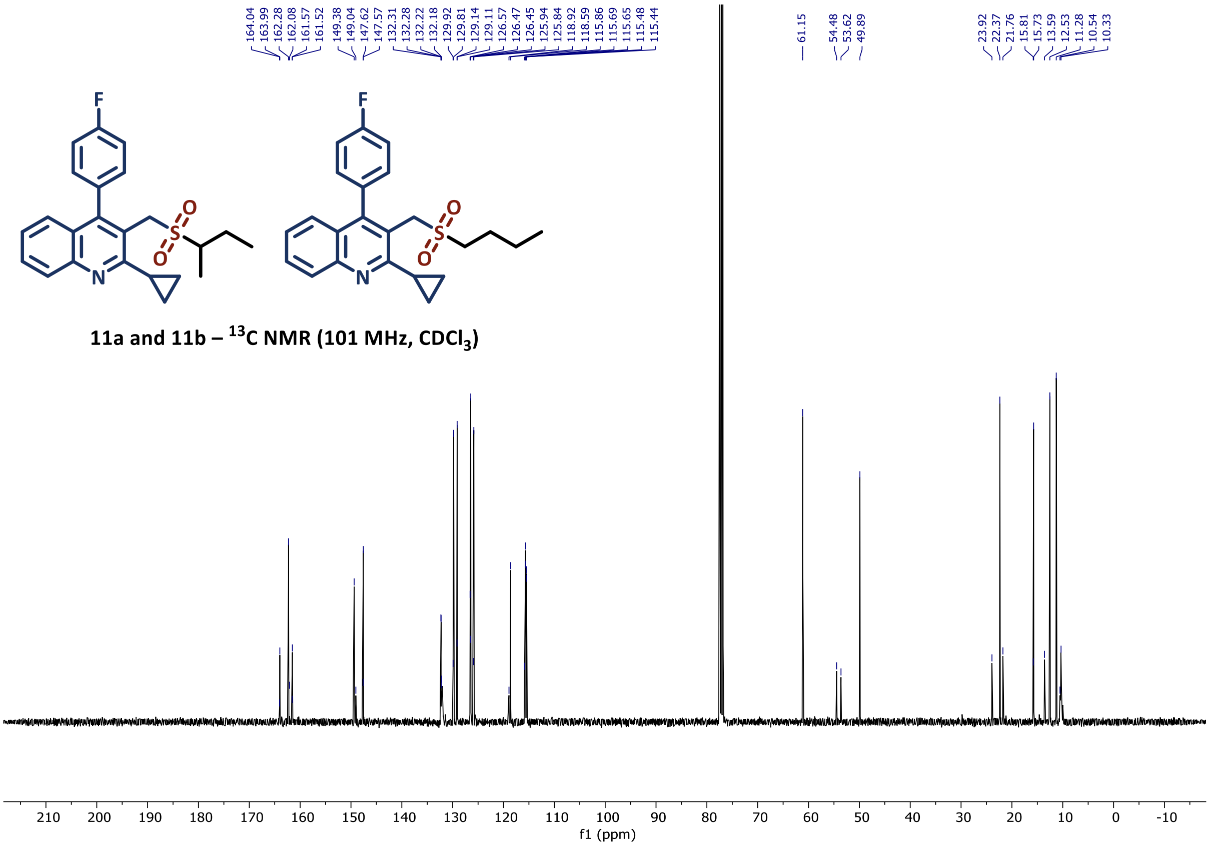

11a and 11b – ^{13}C NMR (101 MHz, $CDCl_3$)

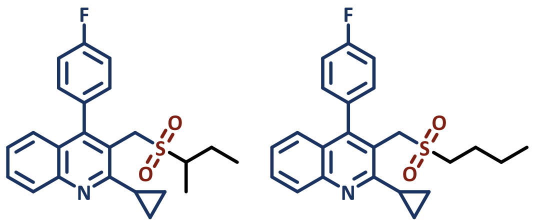

11a and 11b – ^{19}F NMR (282 MHz, $CDCl_3$)

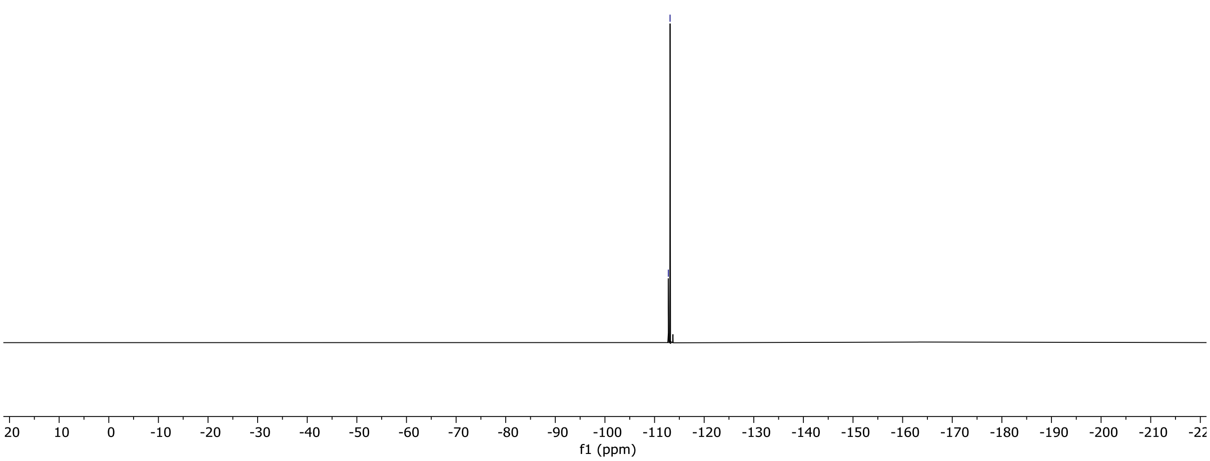

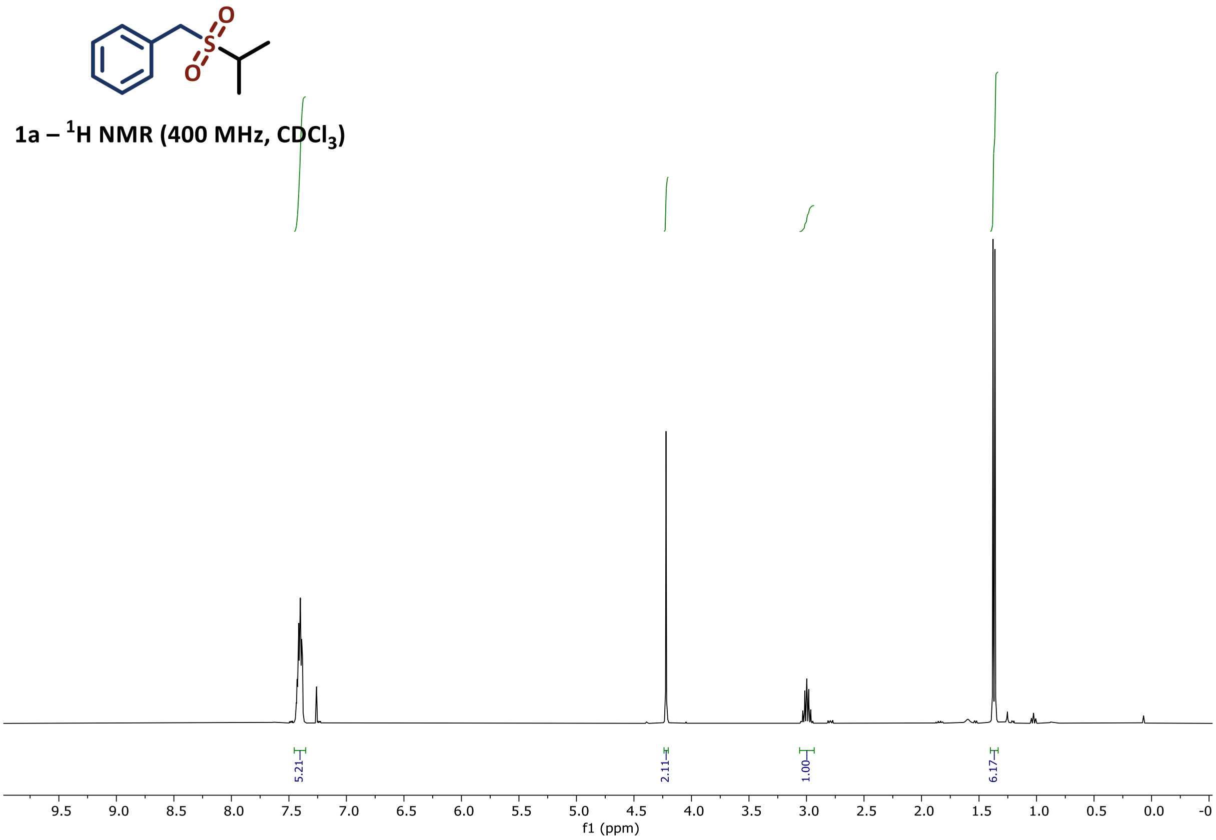

1a – ^{1}H NMR (400 MHz, $CDCl_3$)

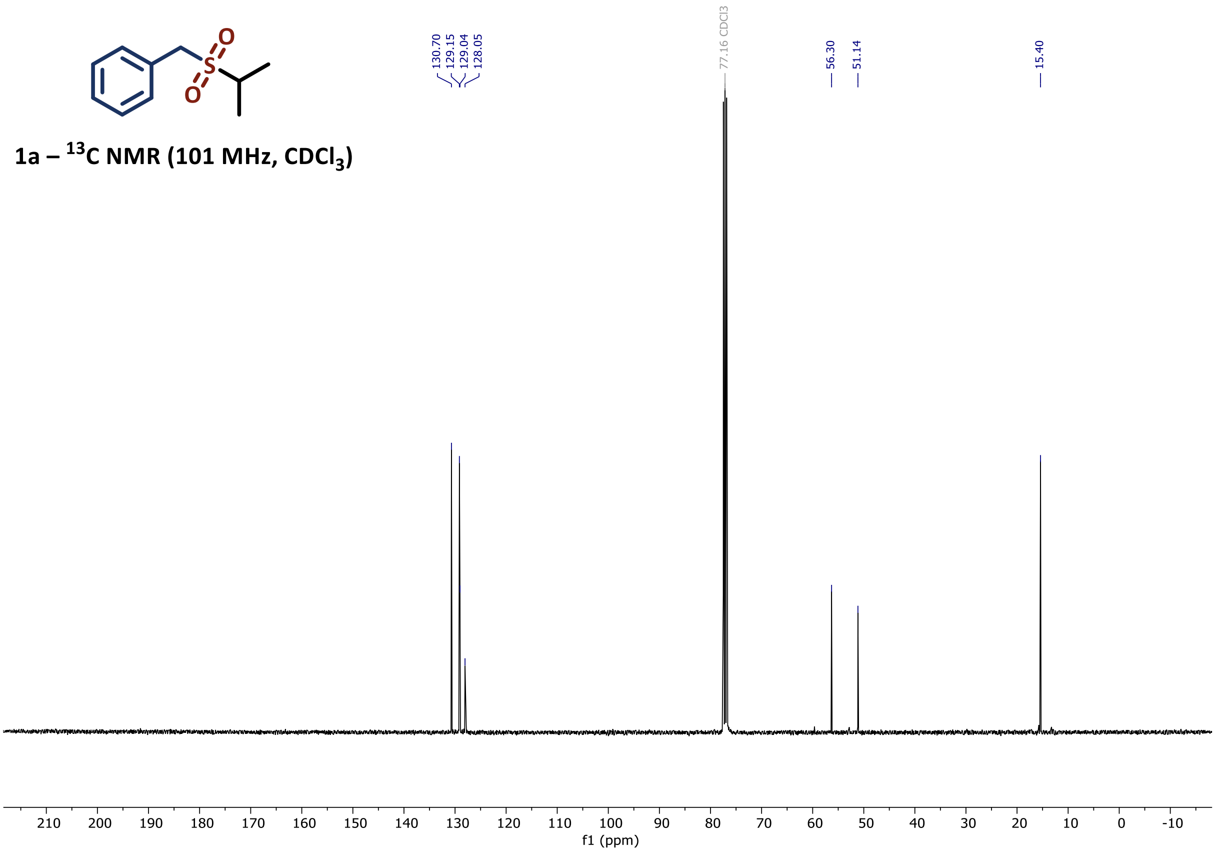

1a – ^{13}C NMR (101 MHz, $CDCl_3$)

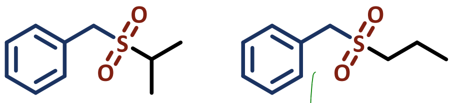

1a and 1b – ^{1}H NMR (300 MHz, $CDCl_3$)

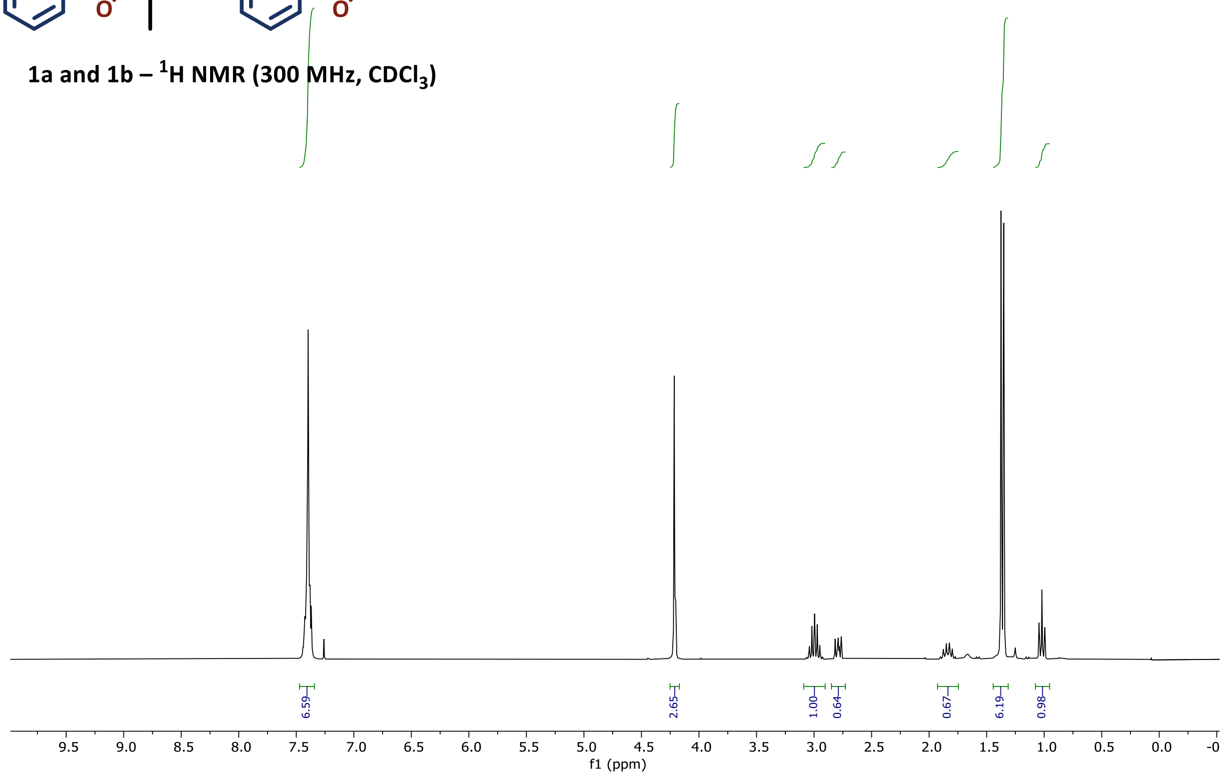

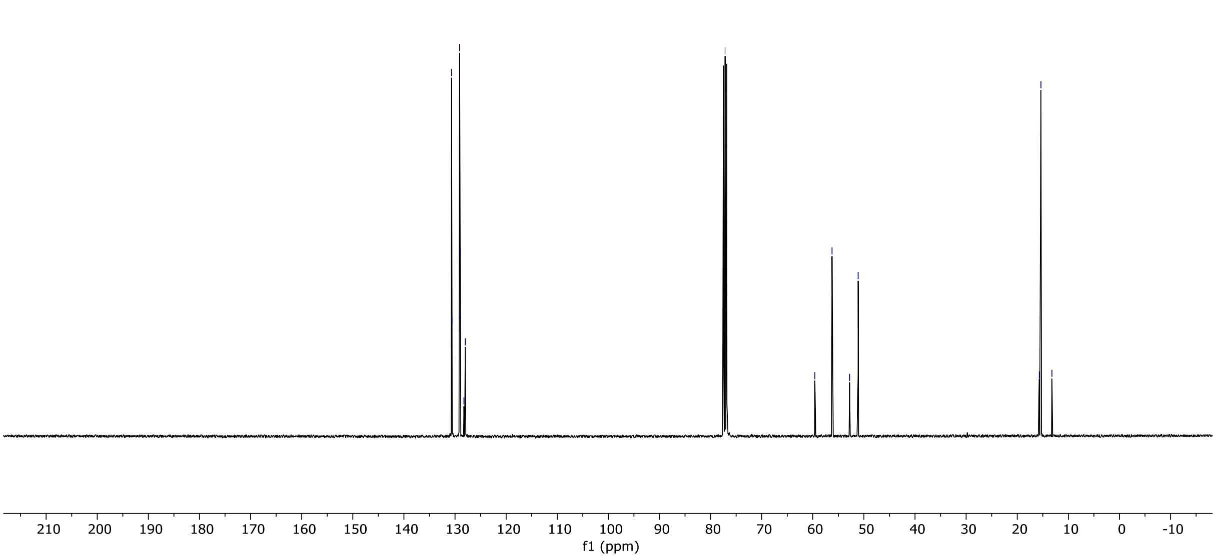

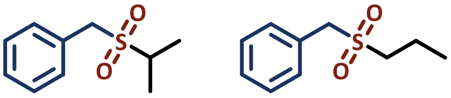

1a and 1b – ^{13}C NMR (101 MHz, $CDCl_3$)

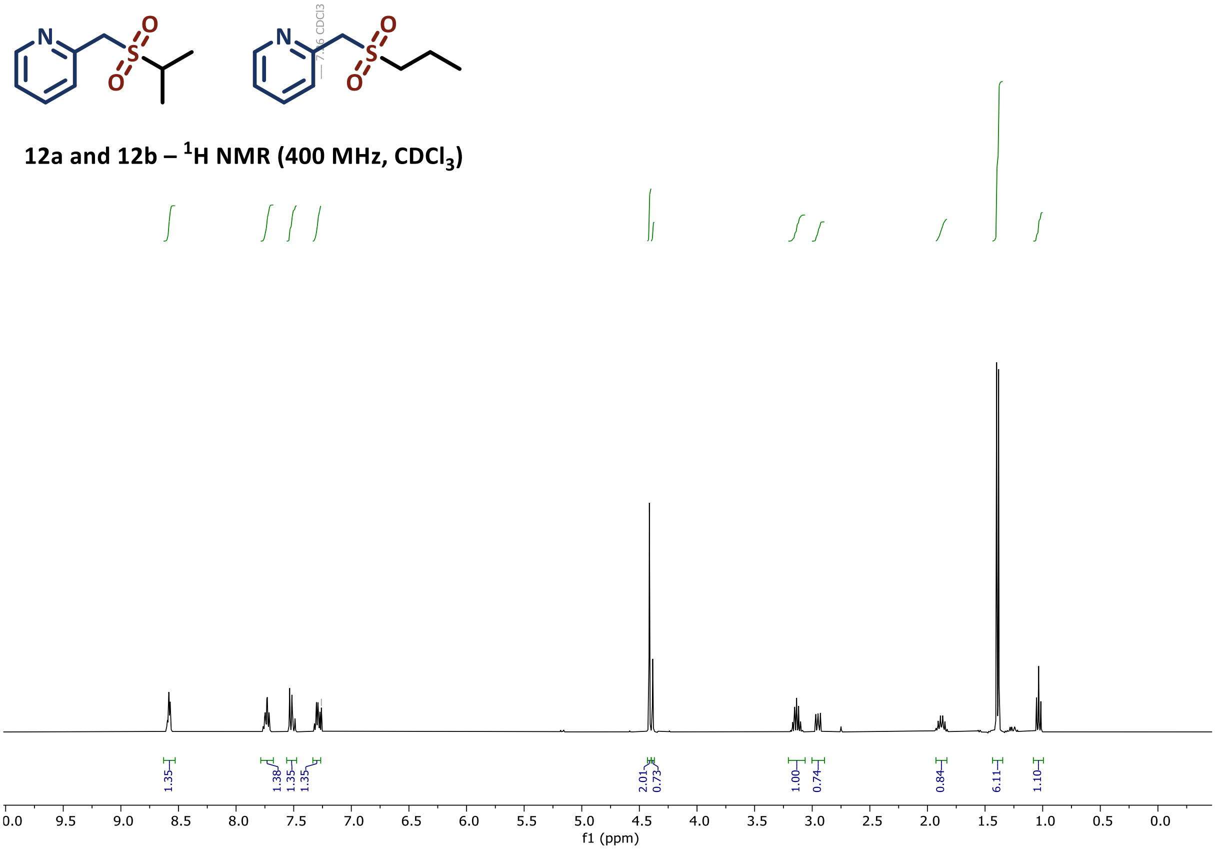

12a and 12b – ^{1}H NMR (400 MHz, $CDCl_3$)

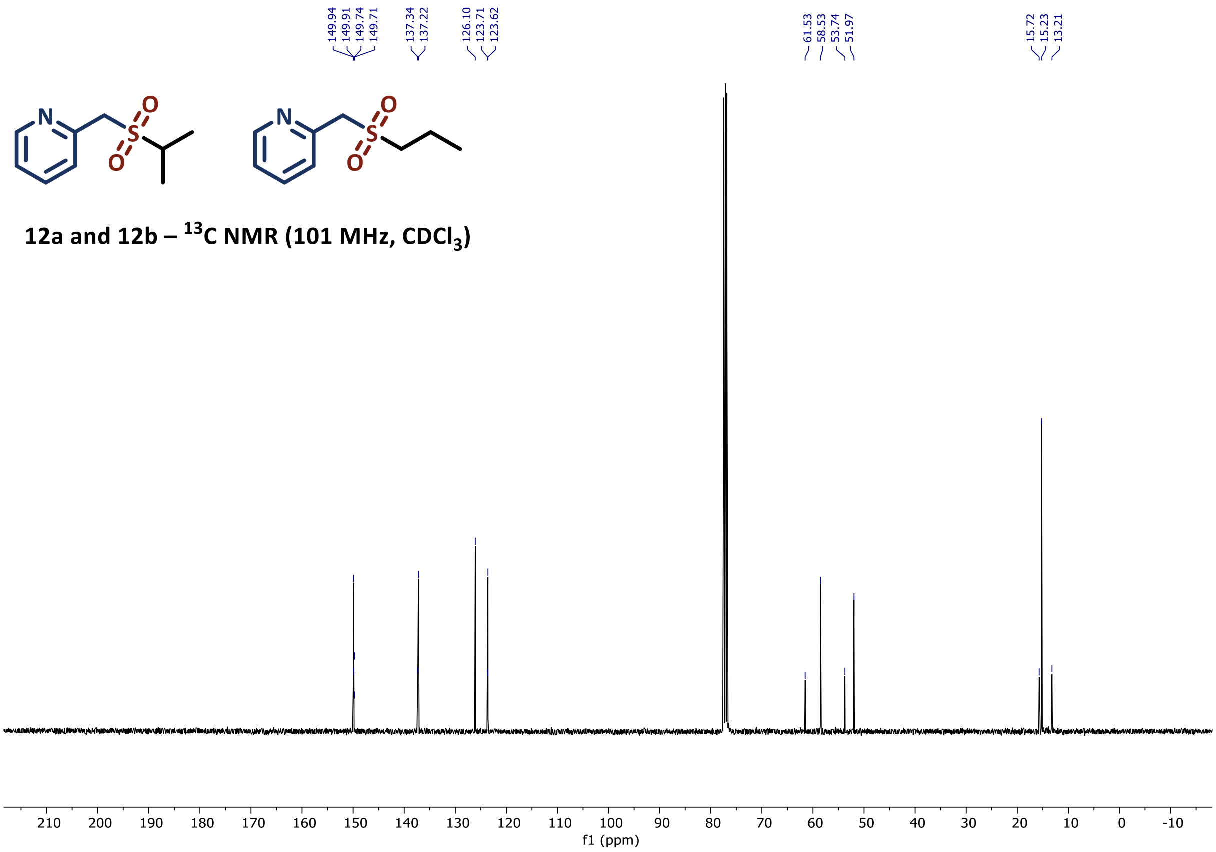

12a and 12b – ^{13}C NMR (101 MHz, $CDCl_3$)

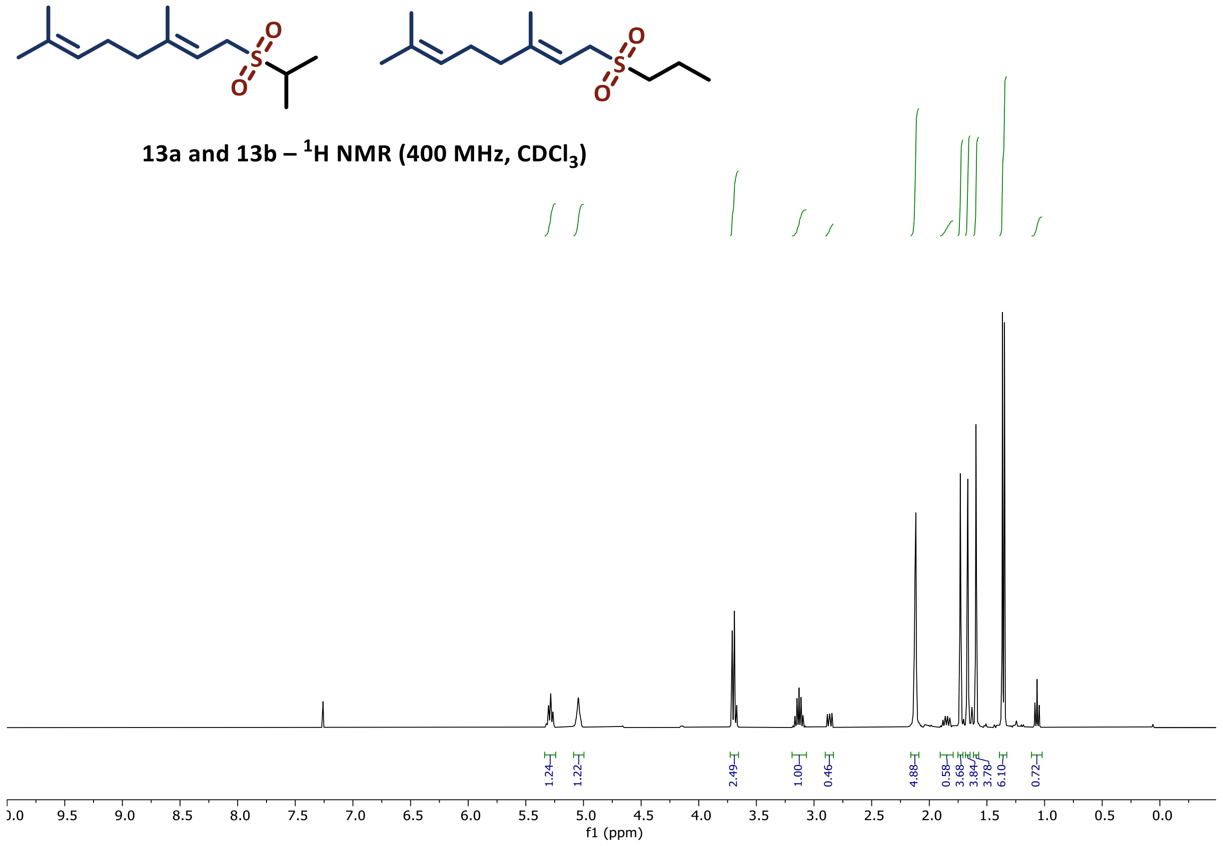

13a and 13b – ^{1}H NMR (400 MHz, $CDCl_3$)

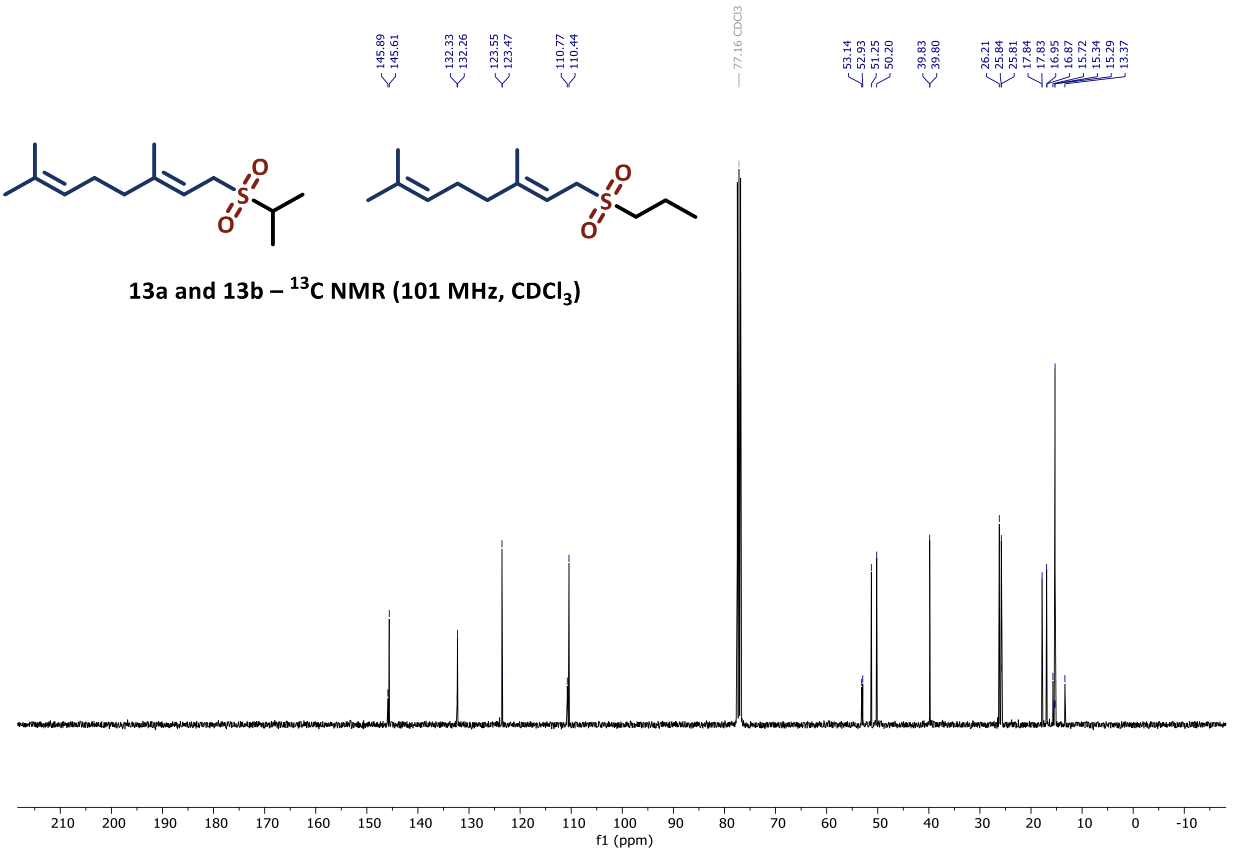

13a and 13b – ^{13}C NMR (101 MHz, $CDCl_3$)

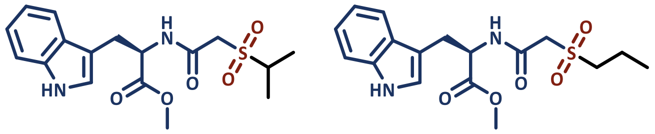

14a and 14b – ^{1}H NMR (400 MHz, CDCl$_3$)

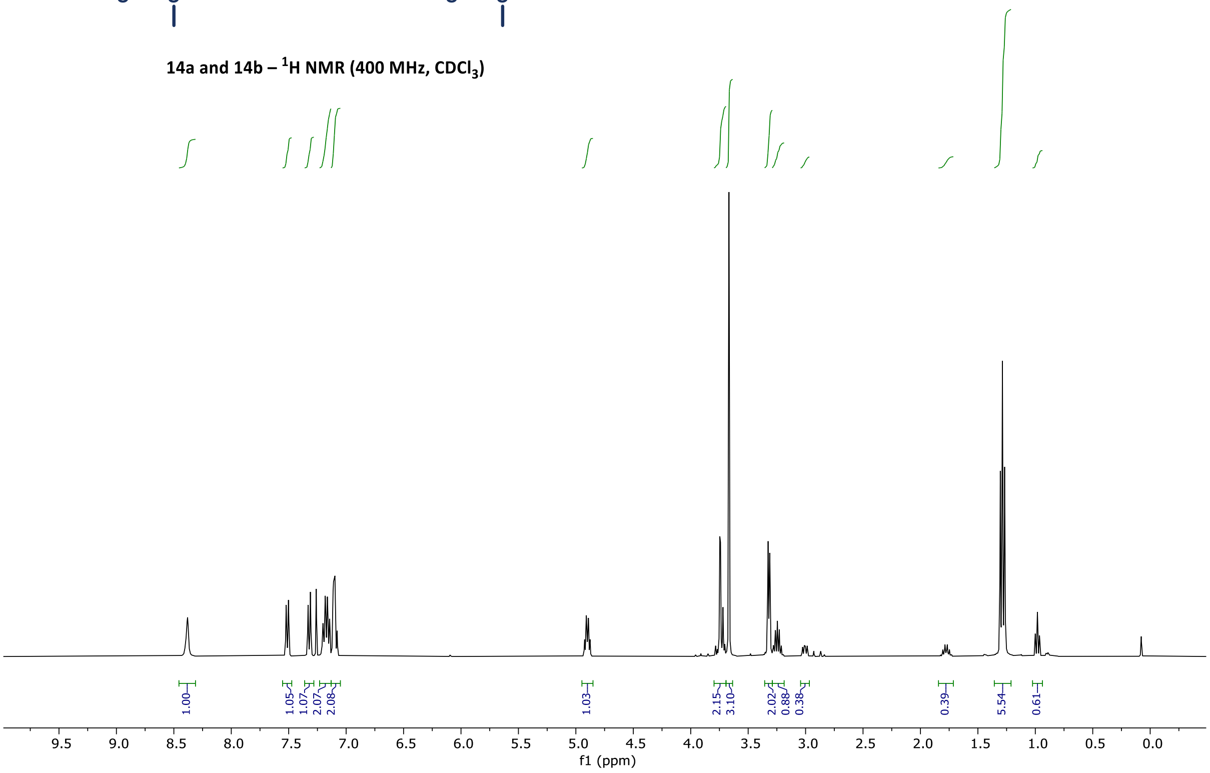

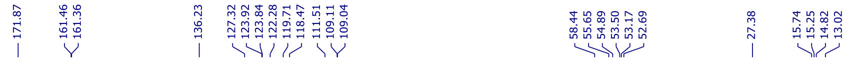

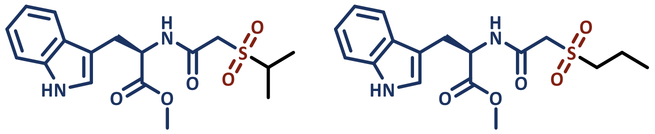

14a and 14b – ^{13}C NMR (101 MHz, CDCl$_3$)

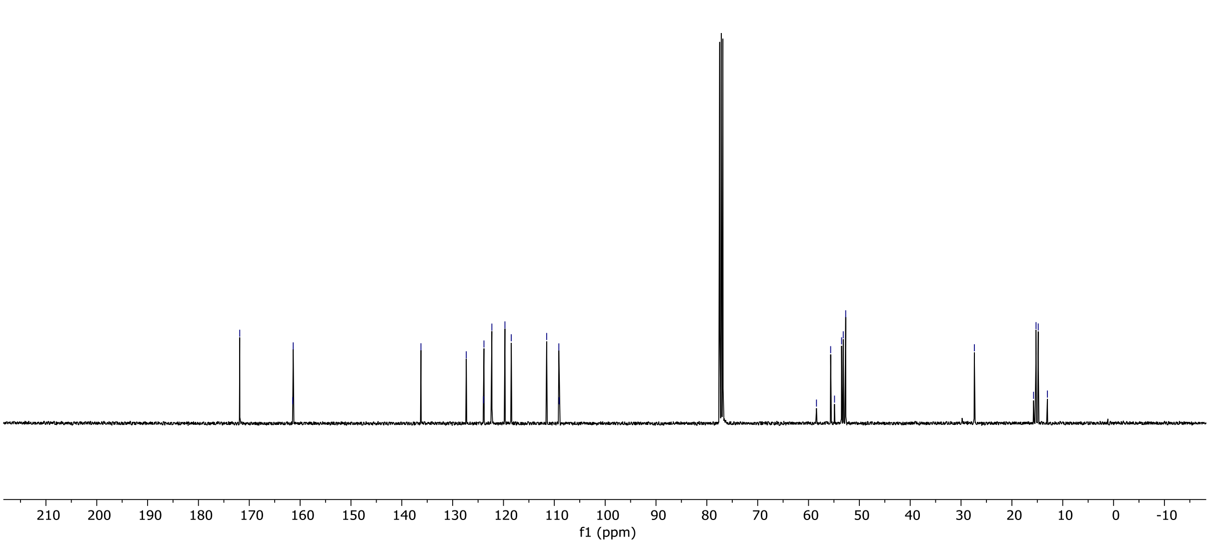

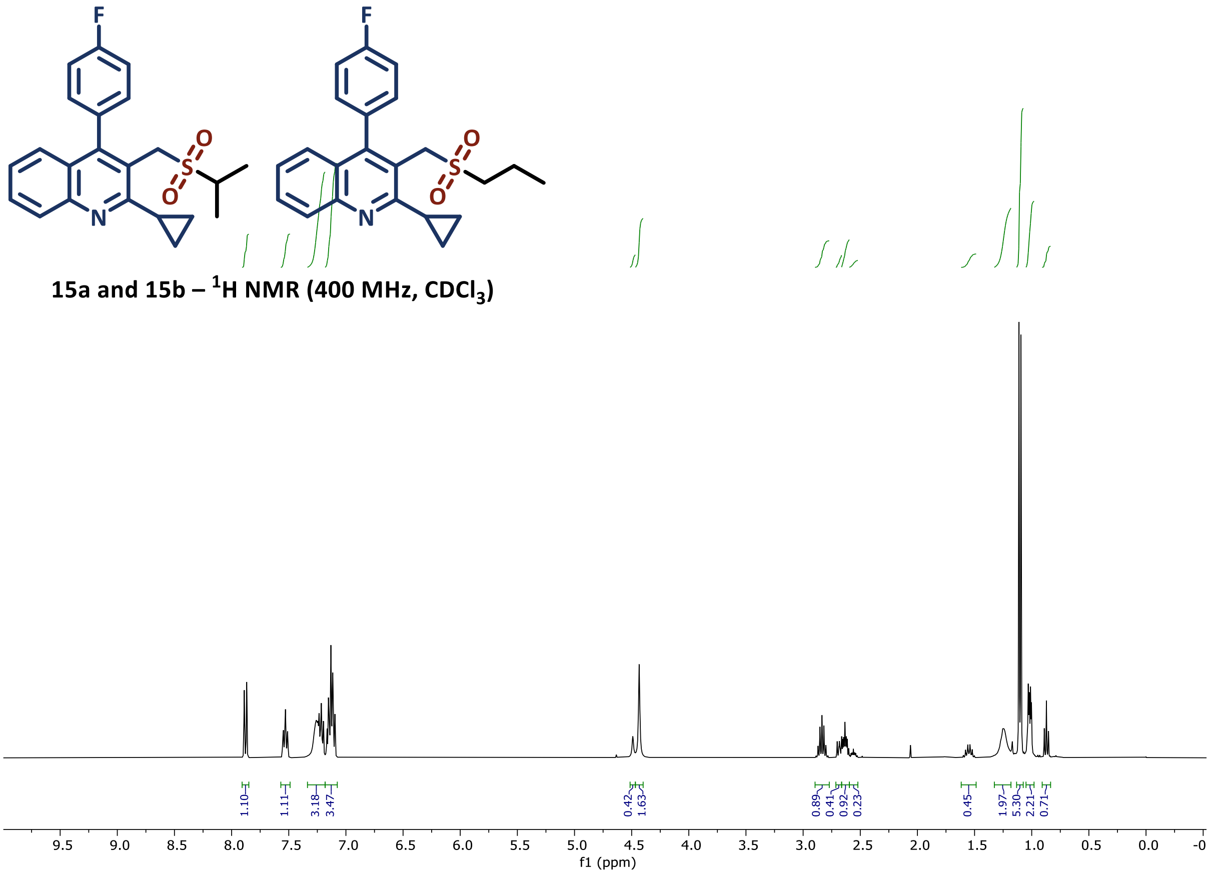

15a and 15b – ^{1}H NMR (400 MHz, $CDCl_3$)

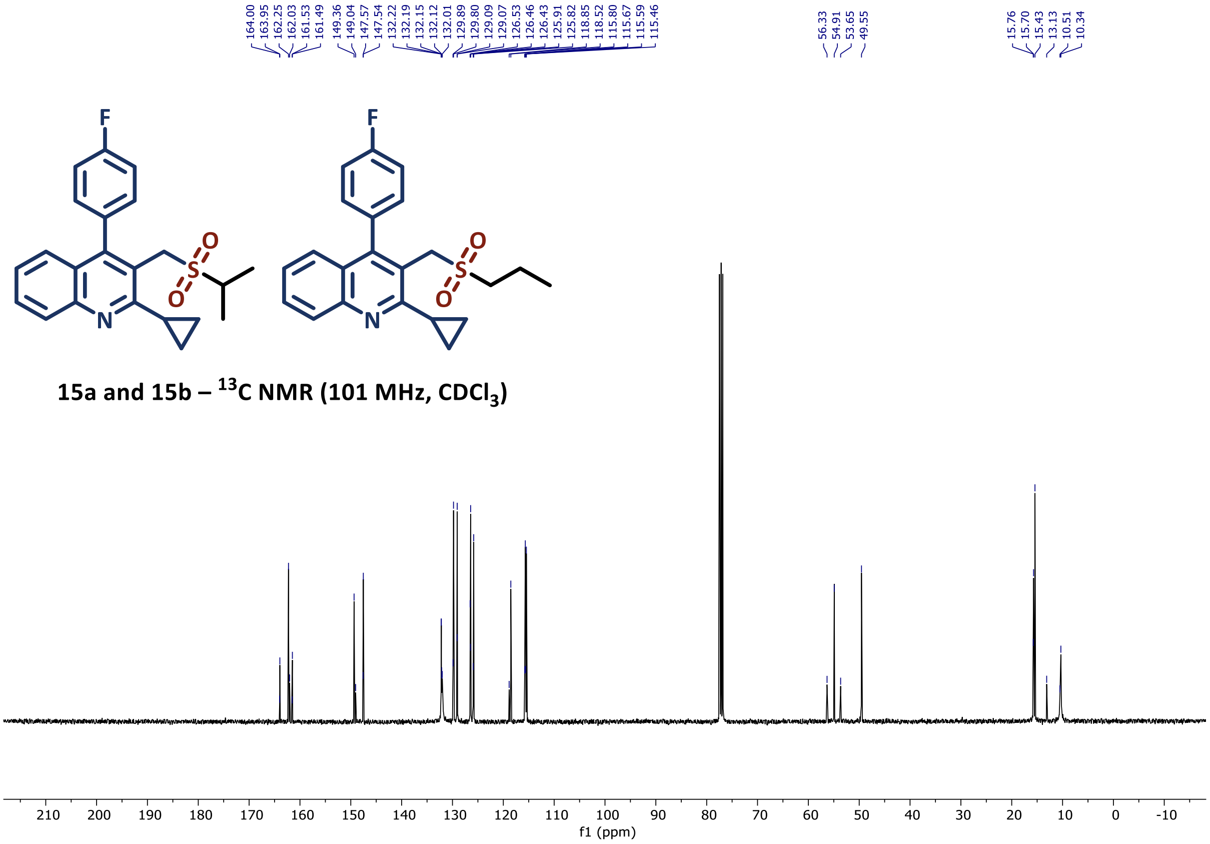

15a and 15b – ^{13}C NMR (101 MHz, $CDCl_3$)

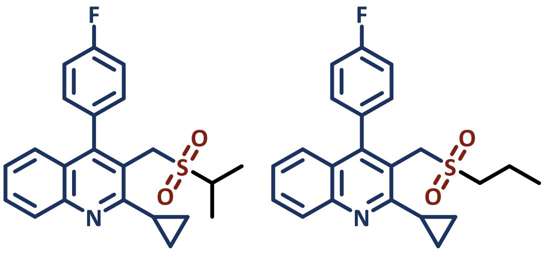

15a and 15b – ^{19}F NMR (282 MHz, $CDCl_3$)

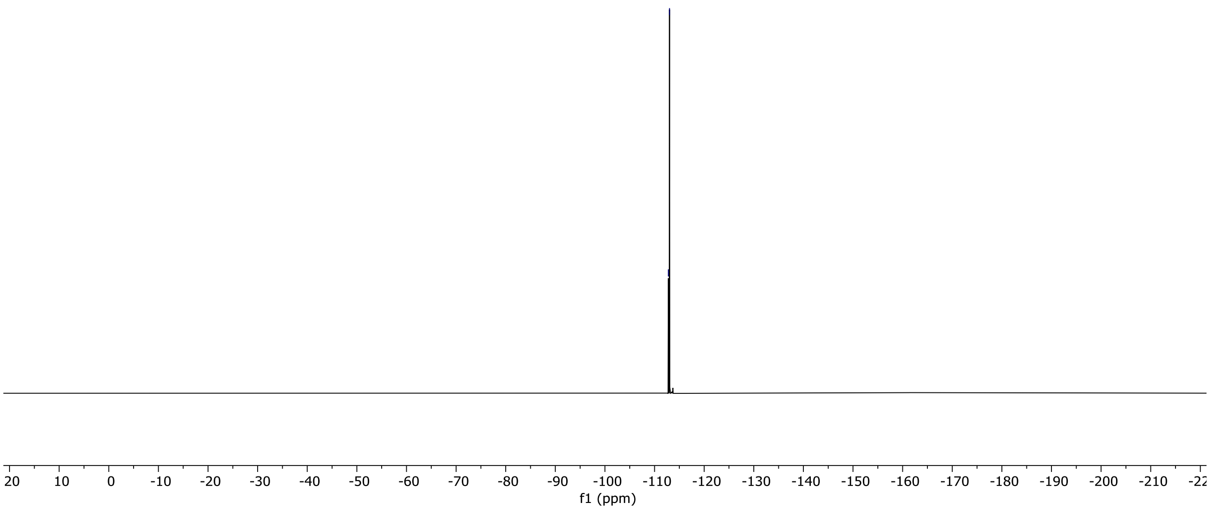

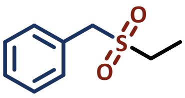

16 – ^{1}H NMR (400 MHz, $CDCl_3$)

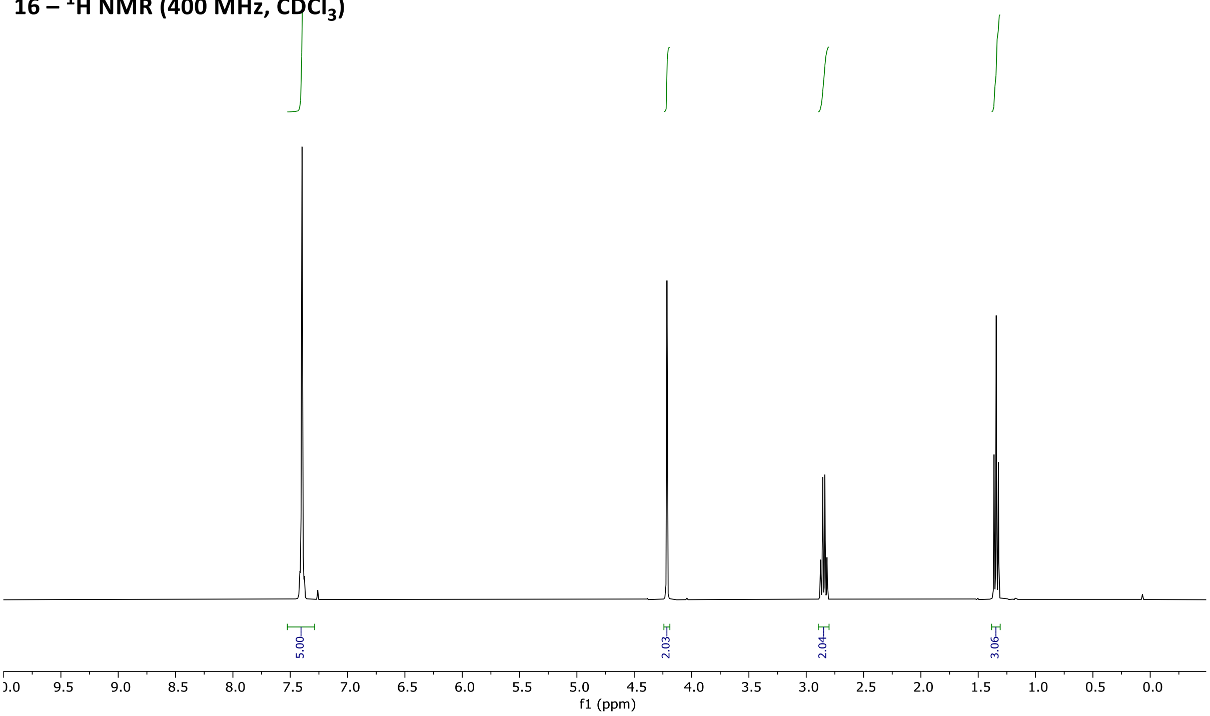

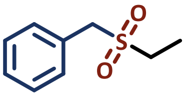

16 – ^{13}C NMR (101 MHz, $CDCl_3$)

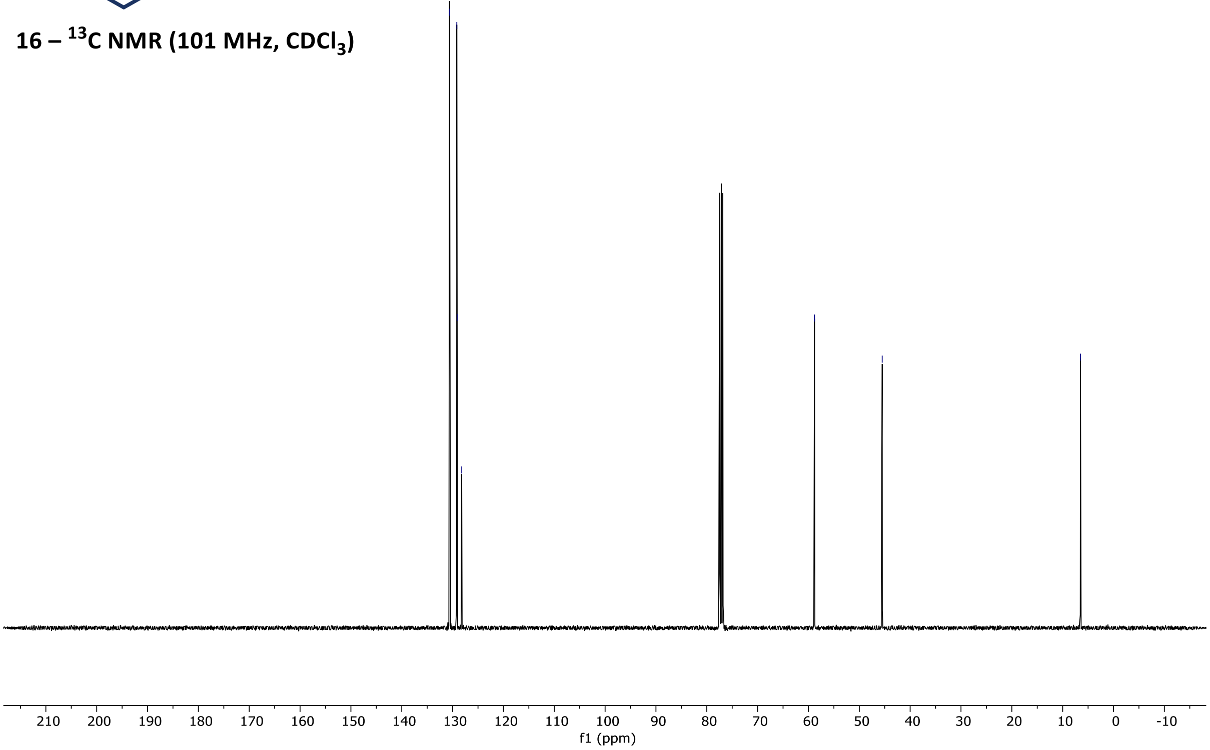

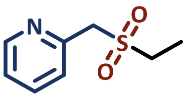

17 – ^{1}H NMR (300 MHz, $CDCl_3$)

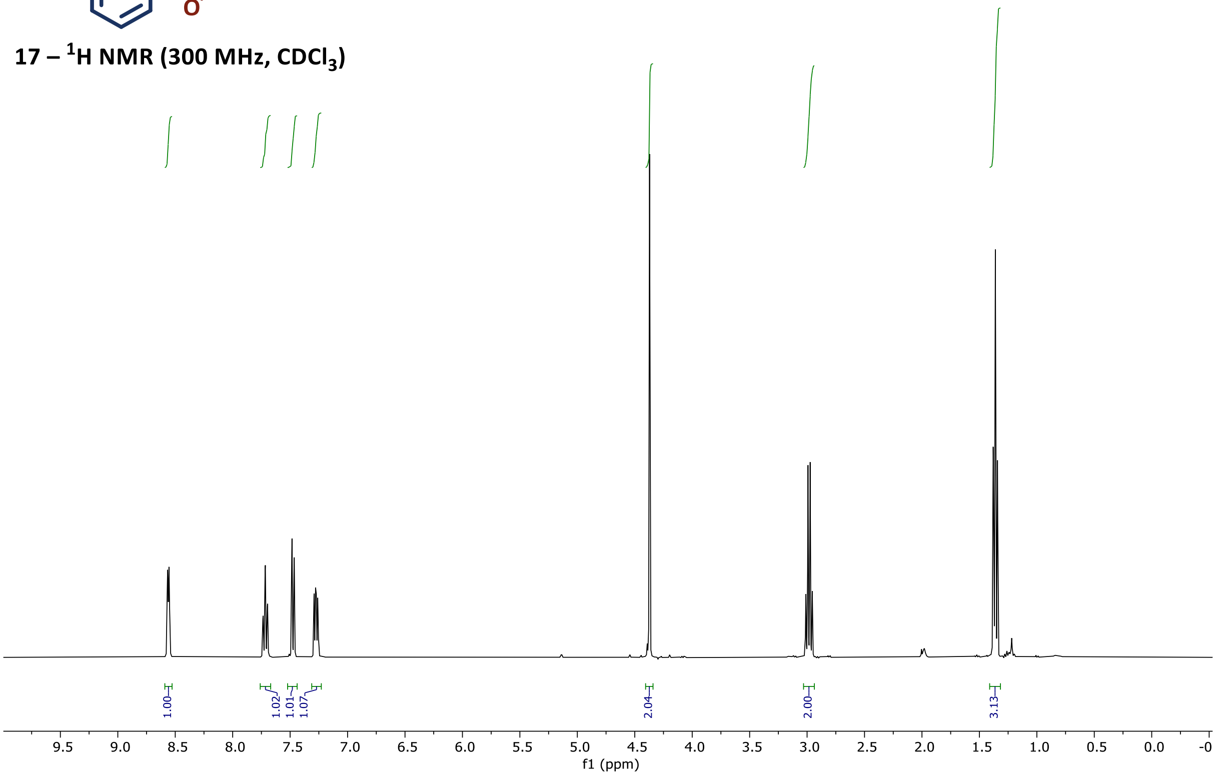

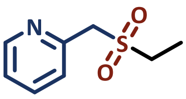

17 – ^{13}C NMR (75 MHz, $CDCl_3$)

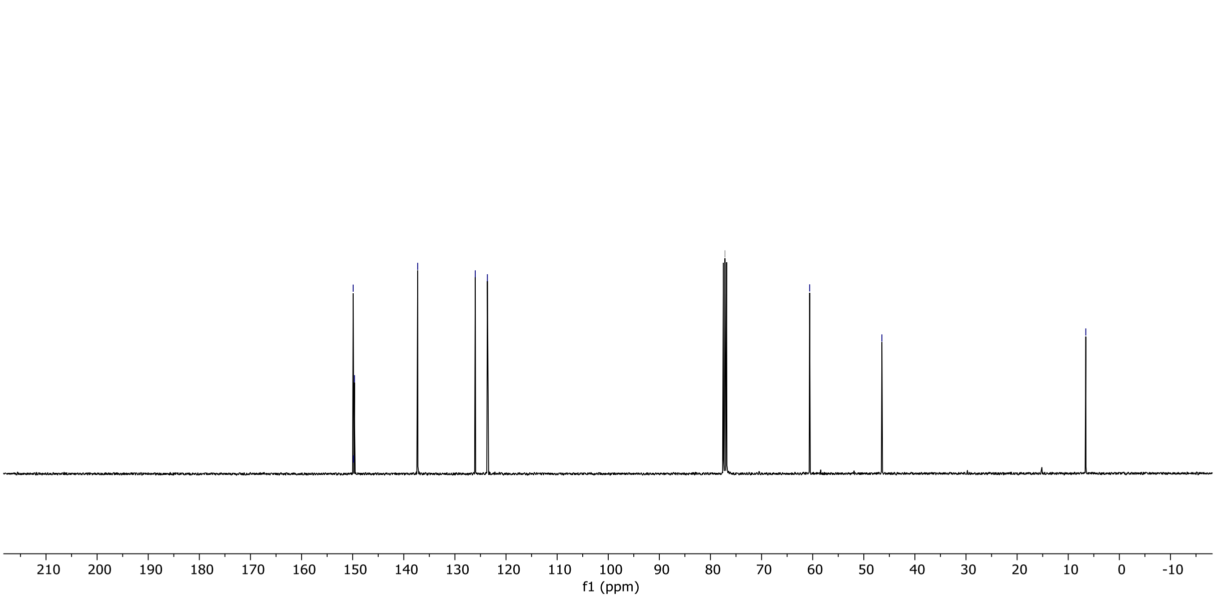

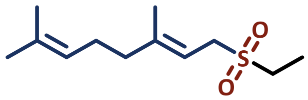

18 – ^{1}H NMR (300 MHz, CDCl$_3$)

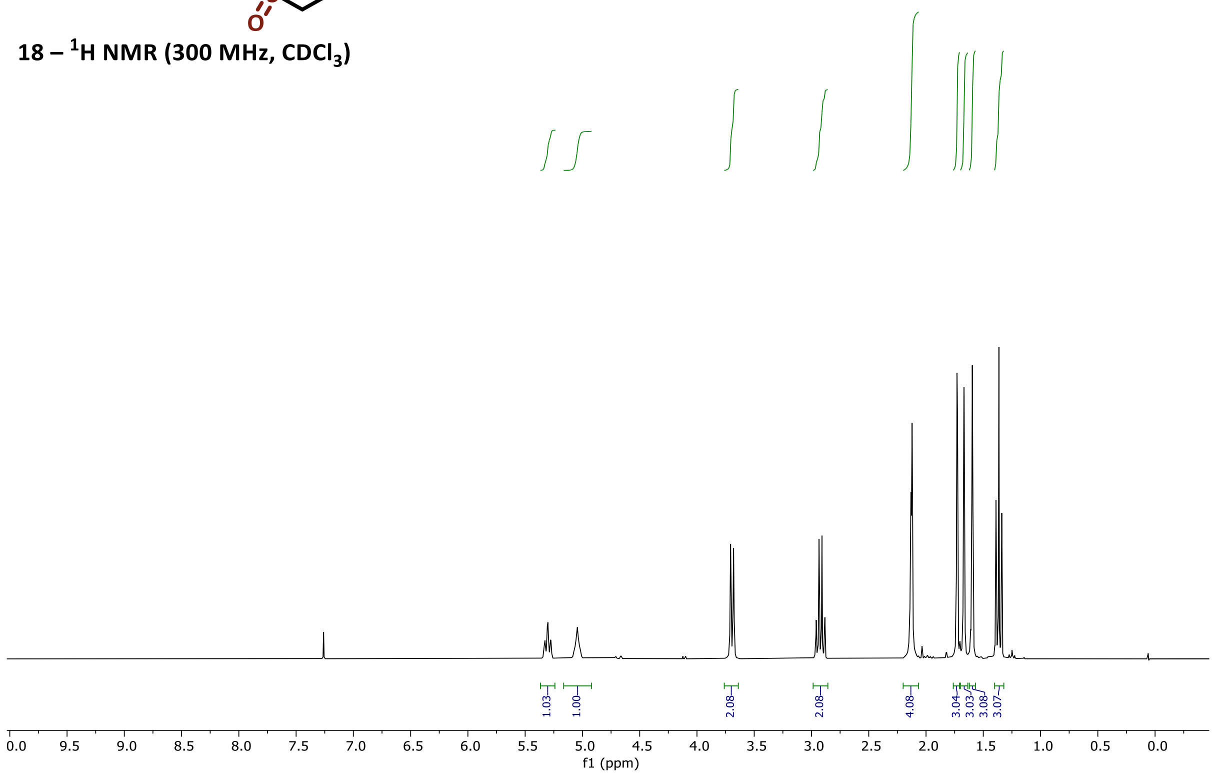

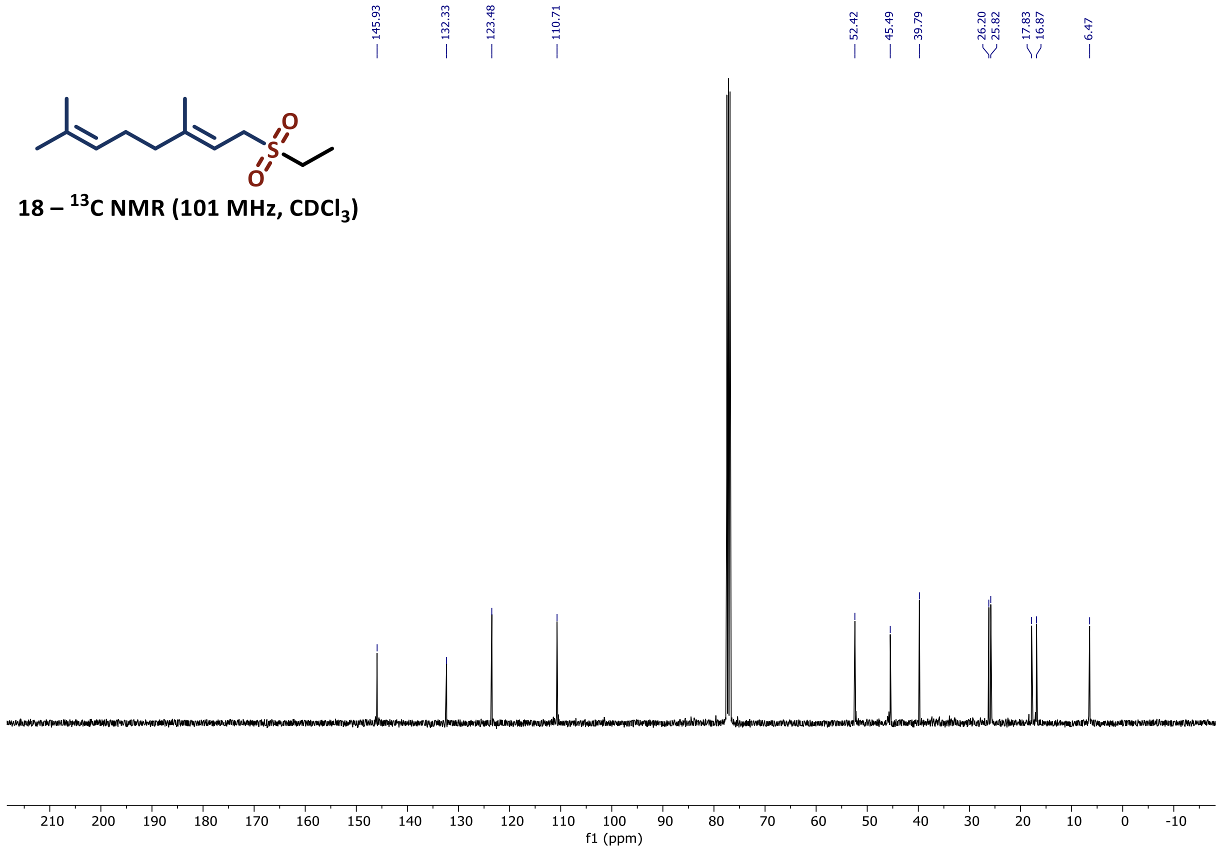

18 – ^{13}C NMR (101 MHz, CDCl$_3$)

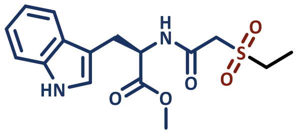

19 – ^{1}H NMR (300 MHz, $CDCl_3$)

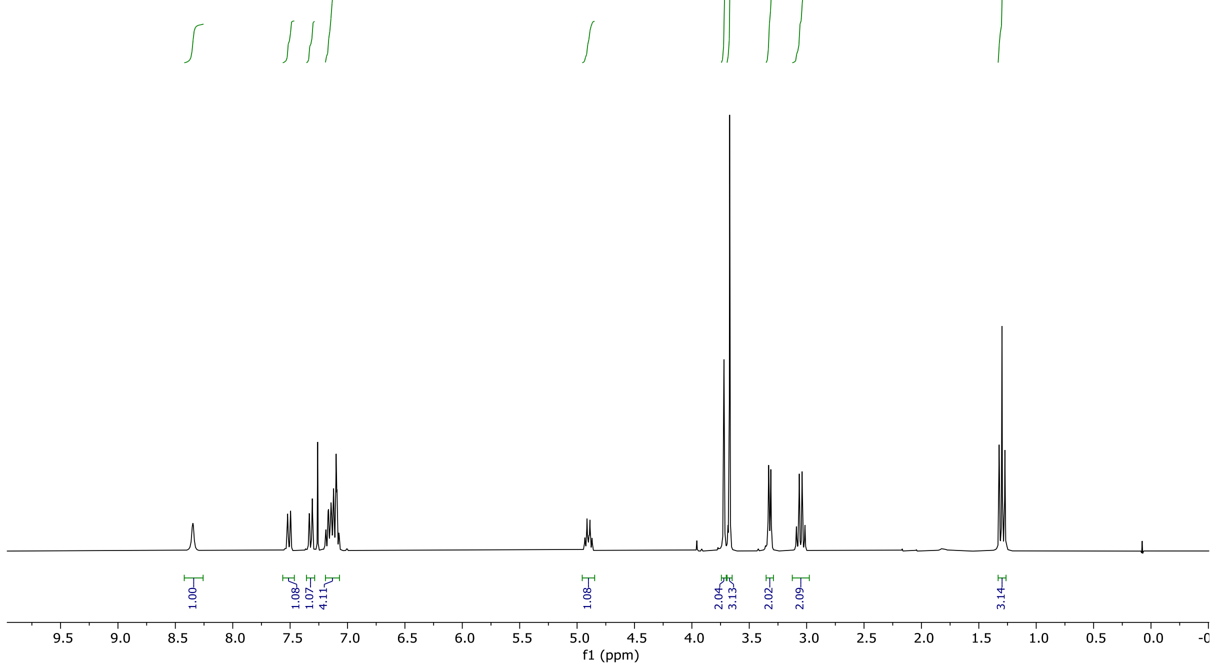

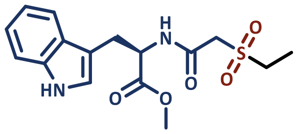

19 – ^{13}C NMR (75 MHz, $CDCl_3$)

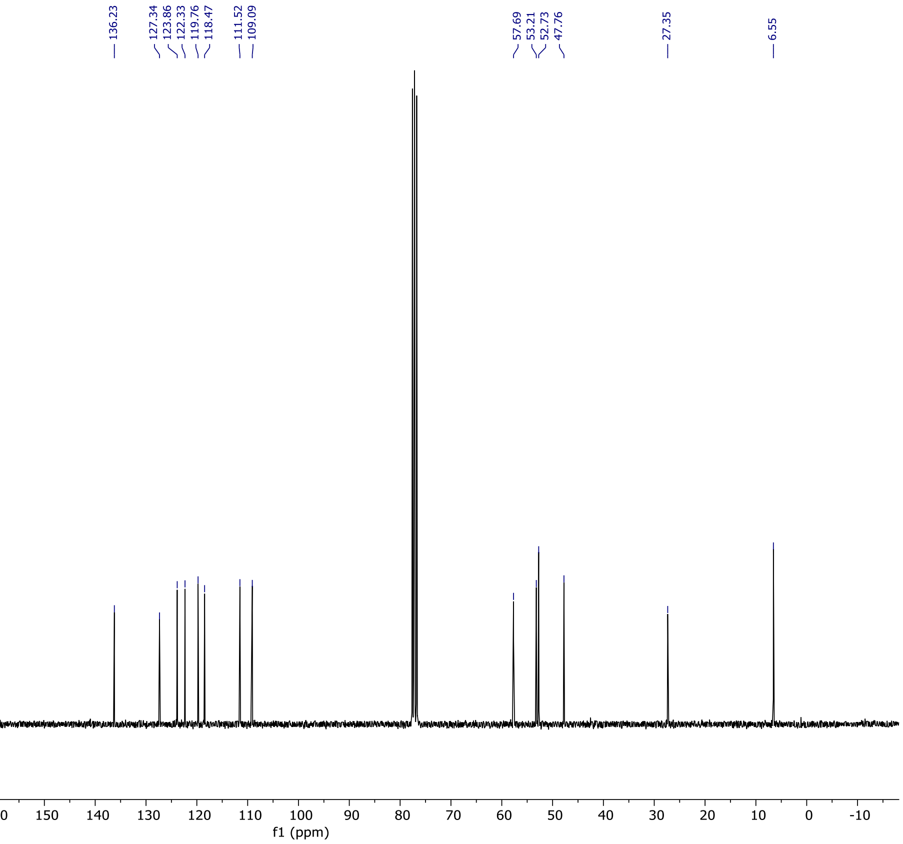

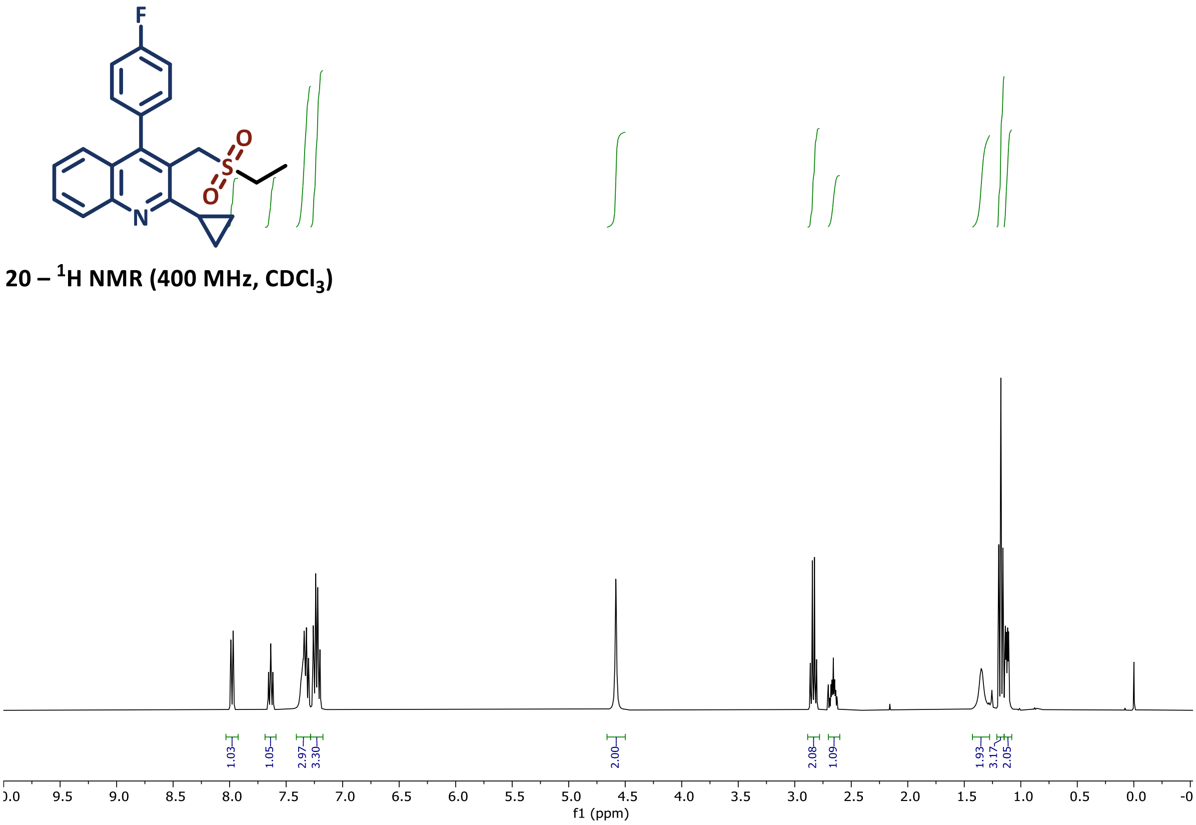

20 – ^{1}H NMR (400 MHz, $CDCl_3$)

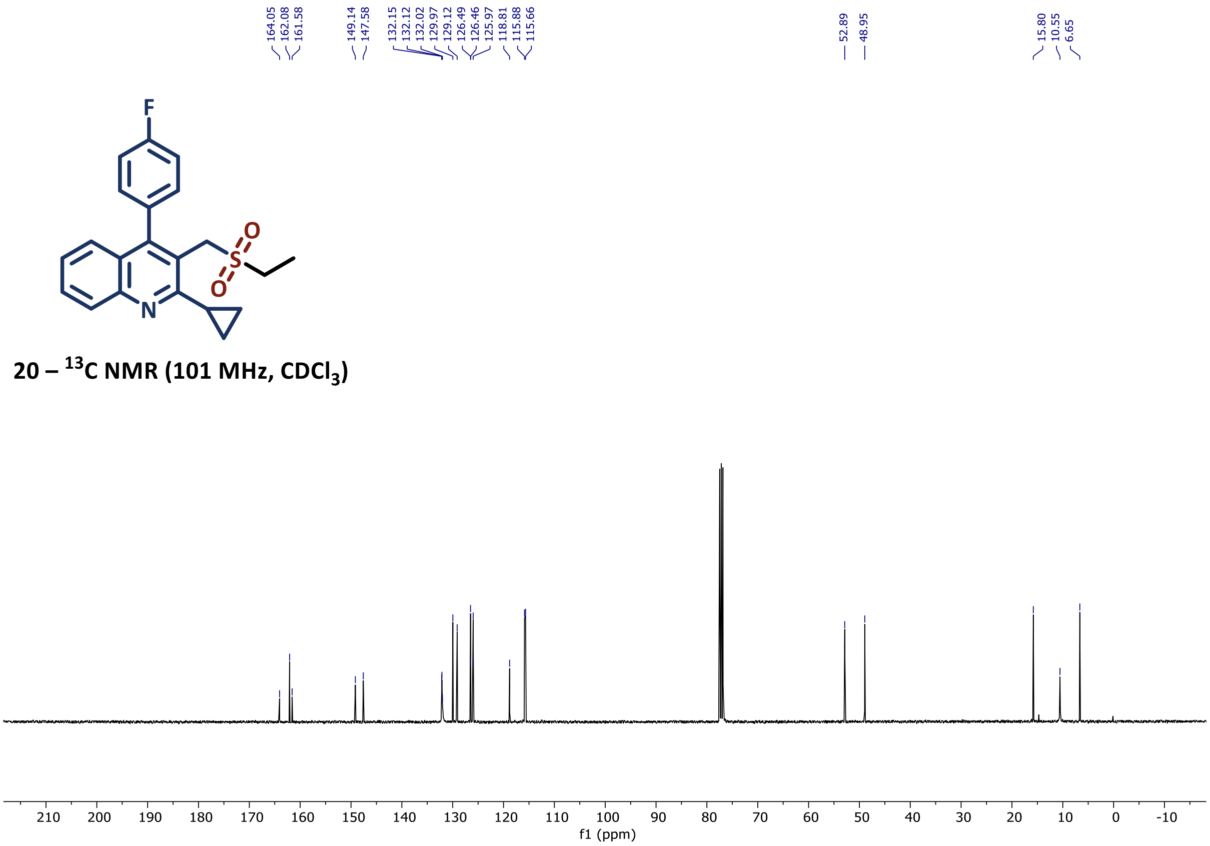

20 – ^{13}C NMR (101 MHz, $CDCl_3$)

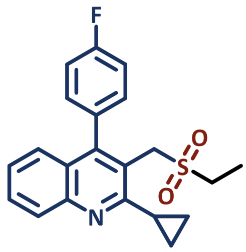

20 – ^{19}F NMR (282 MHz, $CDCl_3$)

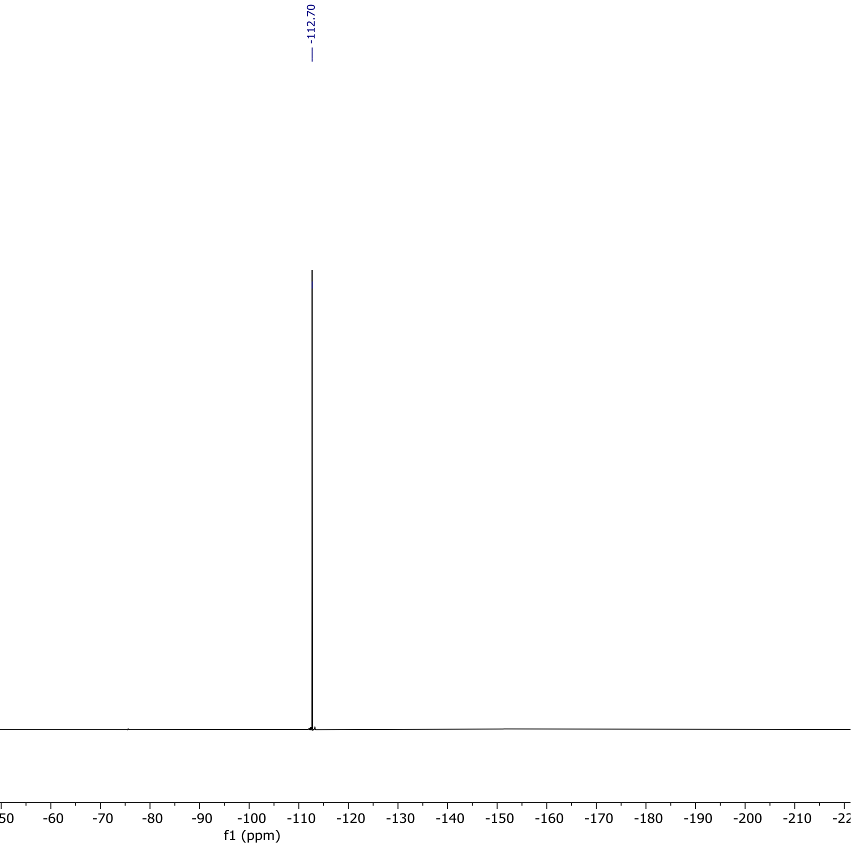

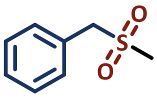

21 – ^{1}H NMR (400 MHz, $CDCl_3$)

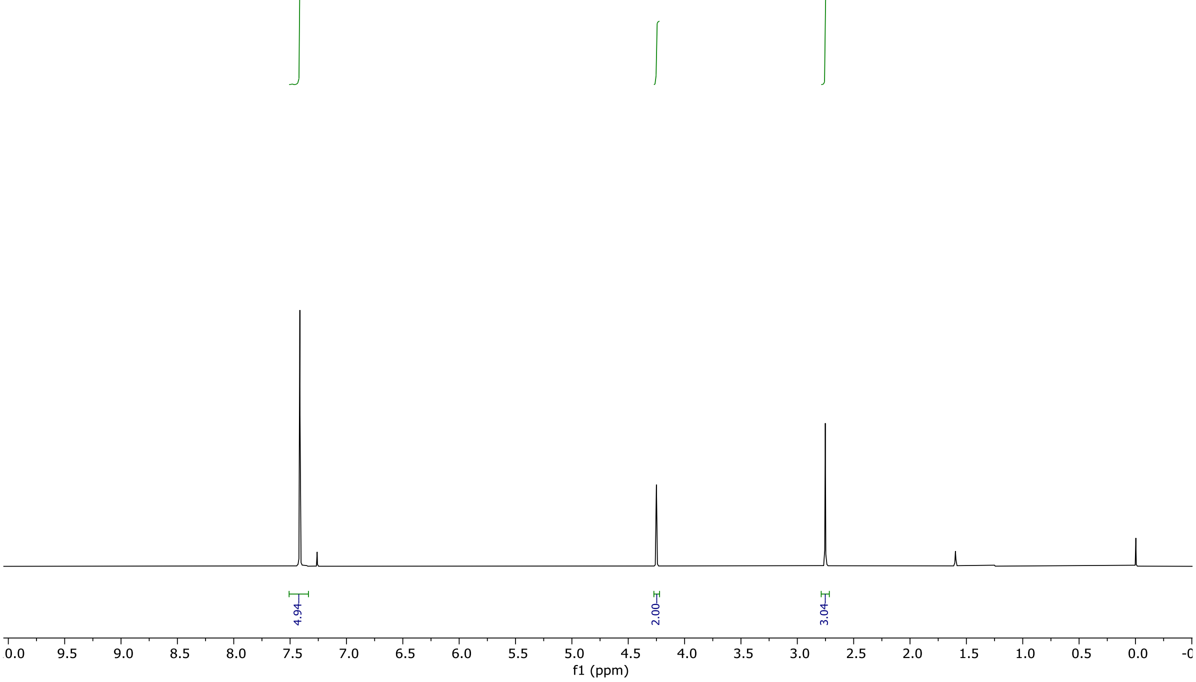

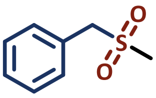

21 – ^{13}C NMR (101 MHz, $CDCl_3$)

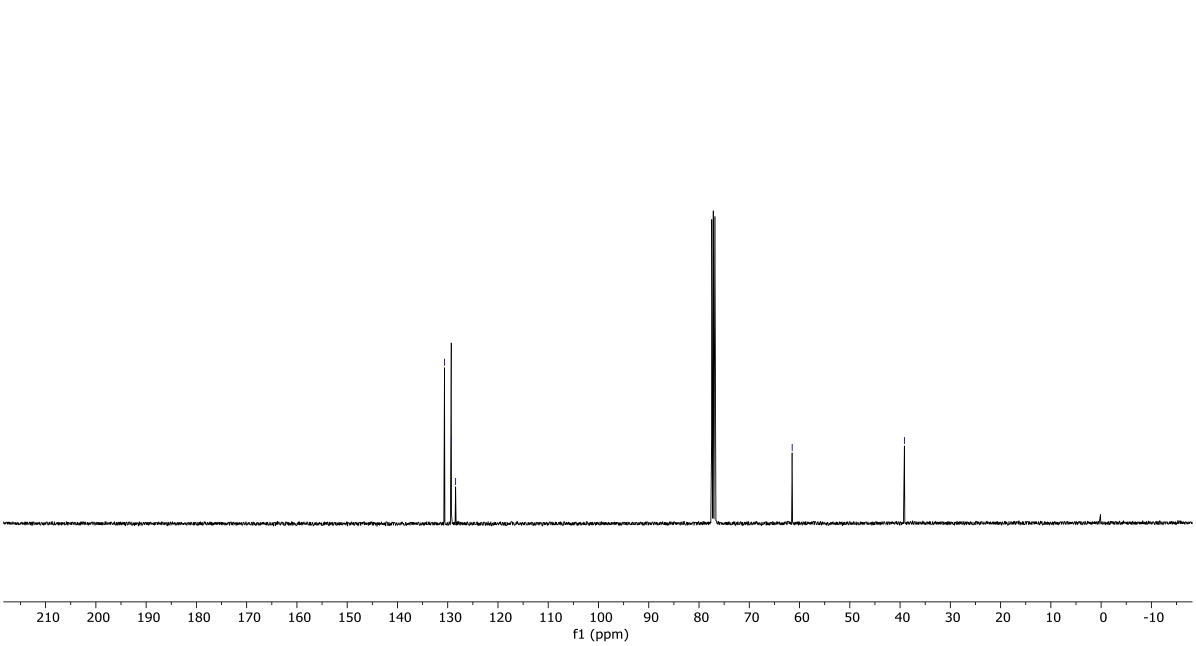

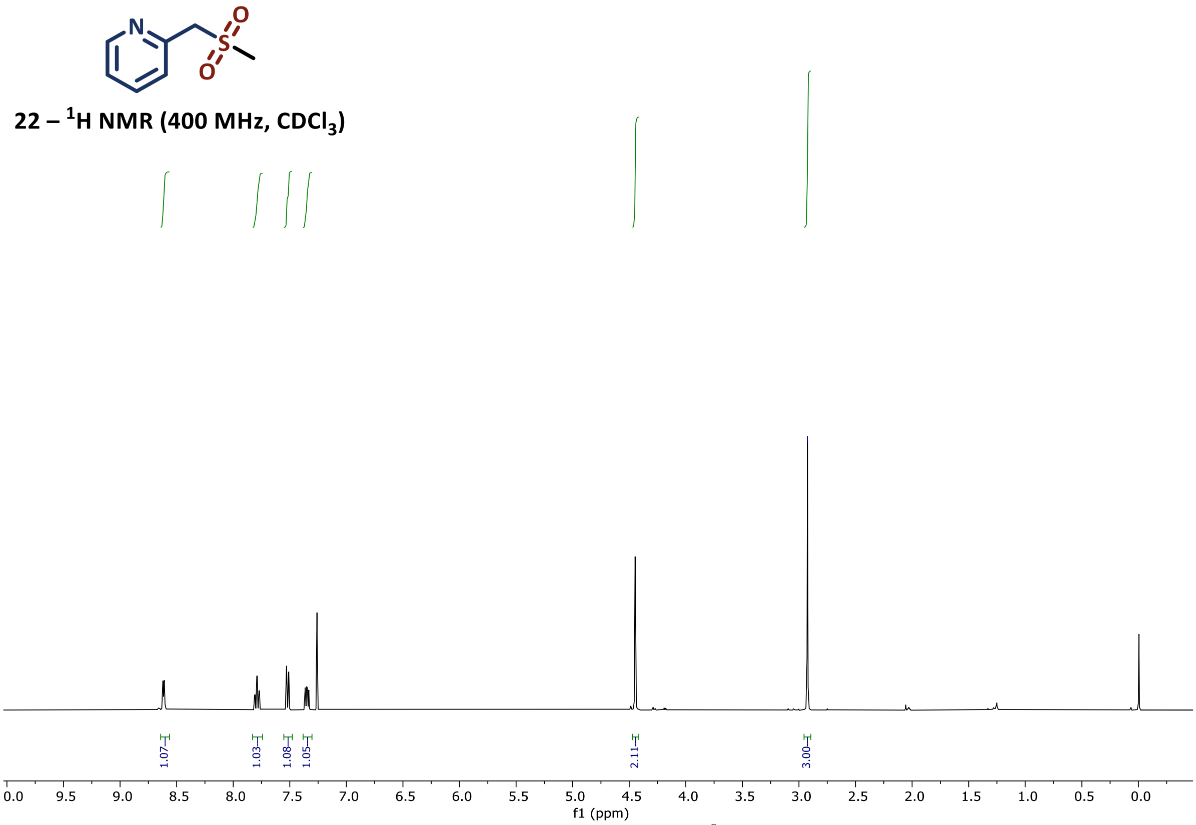

22 – ^{1}H NMR (400 MHz, $CDCl_3$)

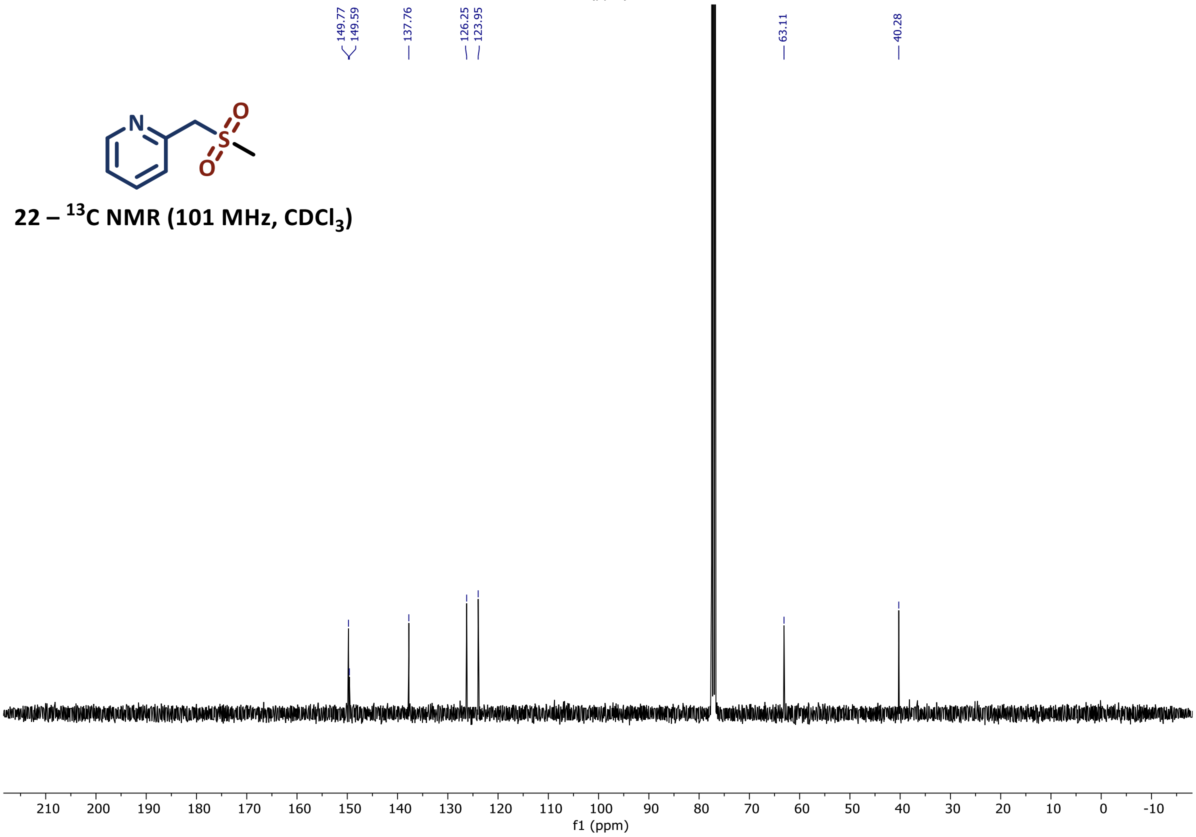

22 – ^{13}C NMR (101 MHz, $CDCl_3$)

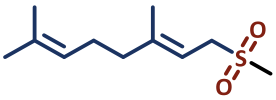

23 – ^{1}H NMR (300 MHz, $CDCl_3$)

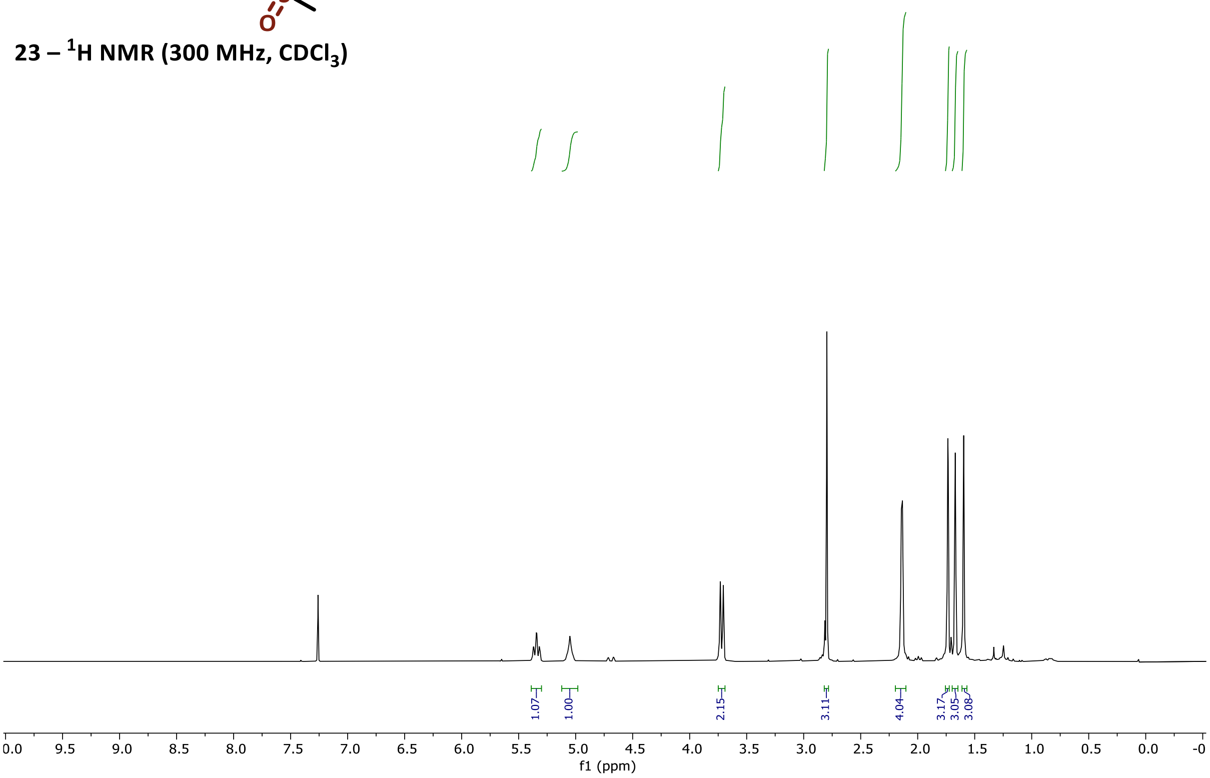

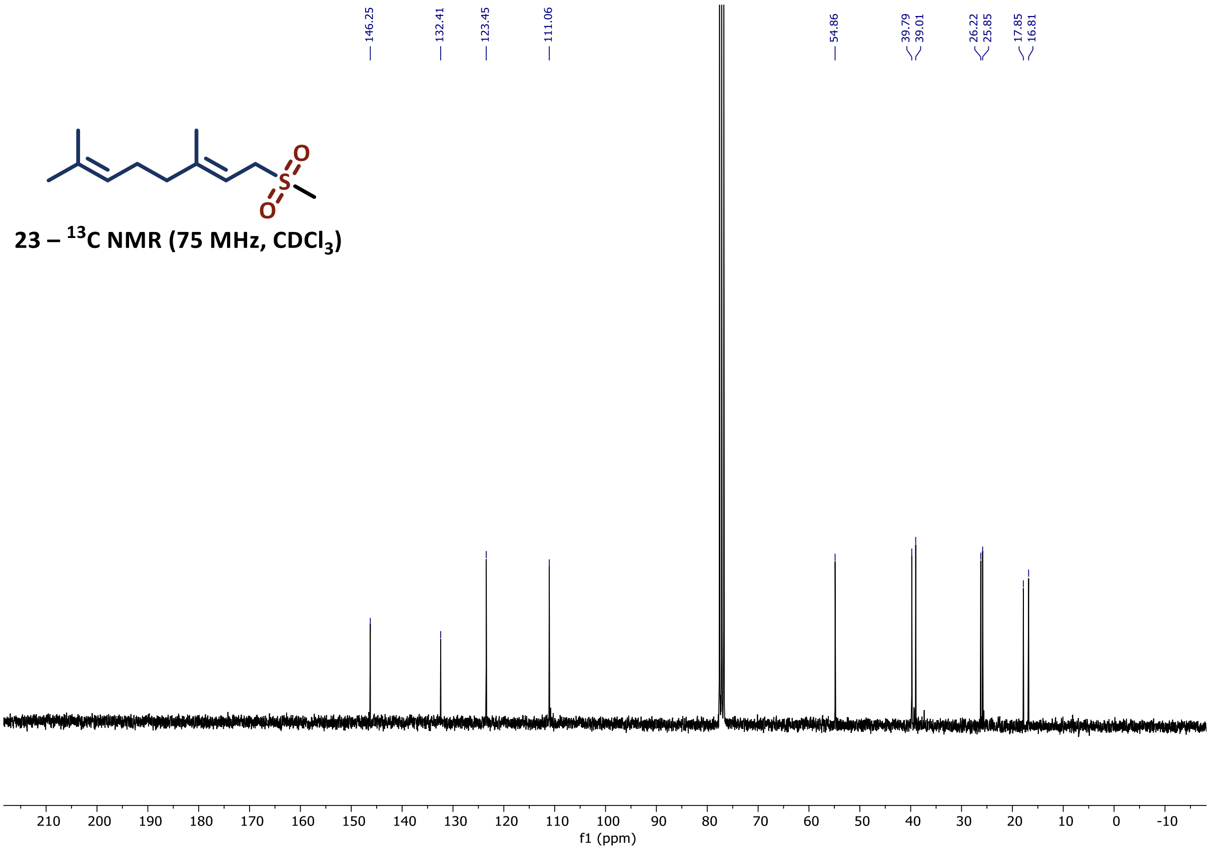

23 – ^{13}C NMR (75 MHz, $CDCl_3$)

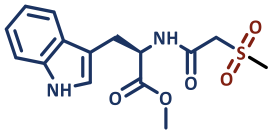

24 – ^{1}H NMR (300 MHz, $CDCl_3$)

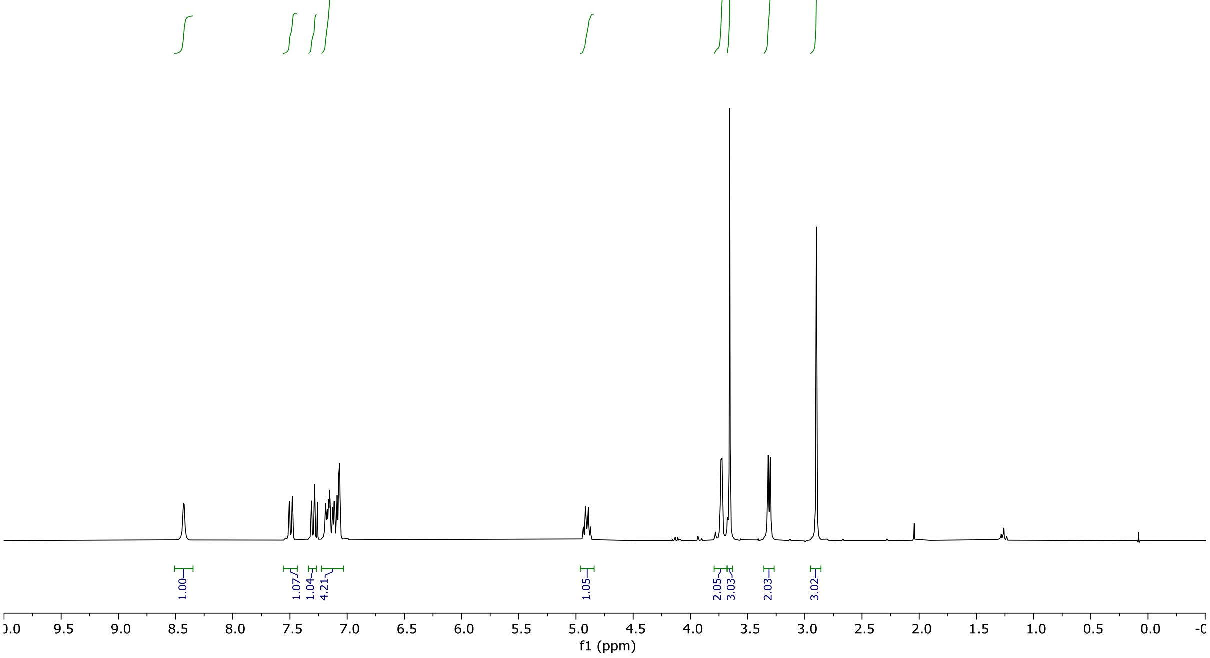

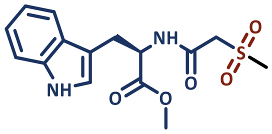

24 – ^{13}C NMR (101 MHz, $CDCl_3$)

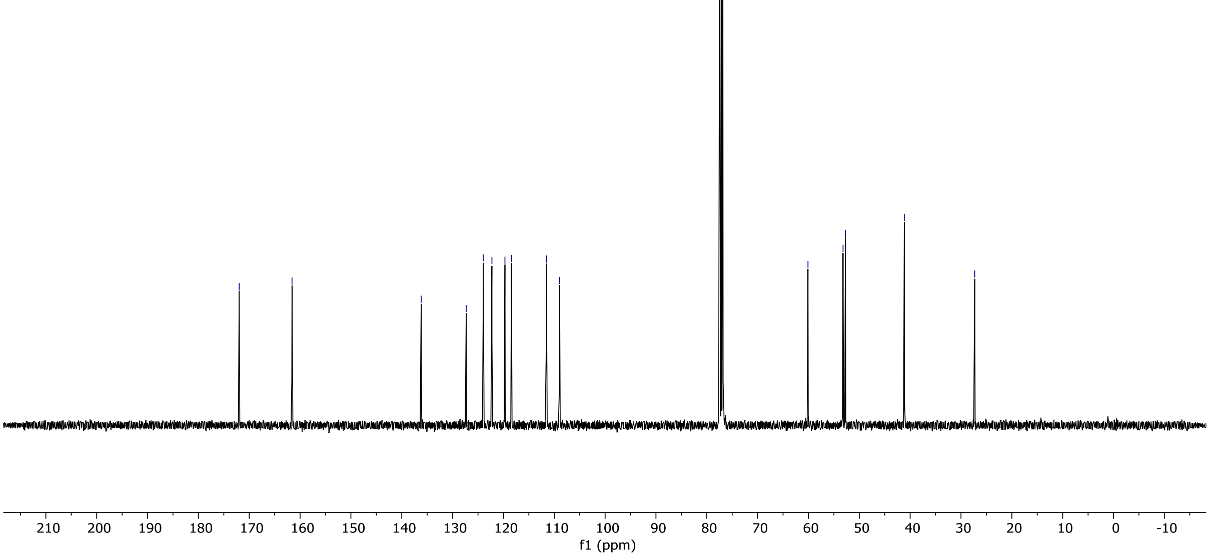

25 – ^{1}H NMR (300 MHz, $CDCl_3$)

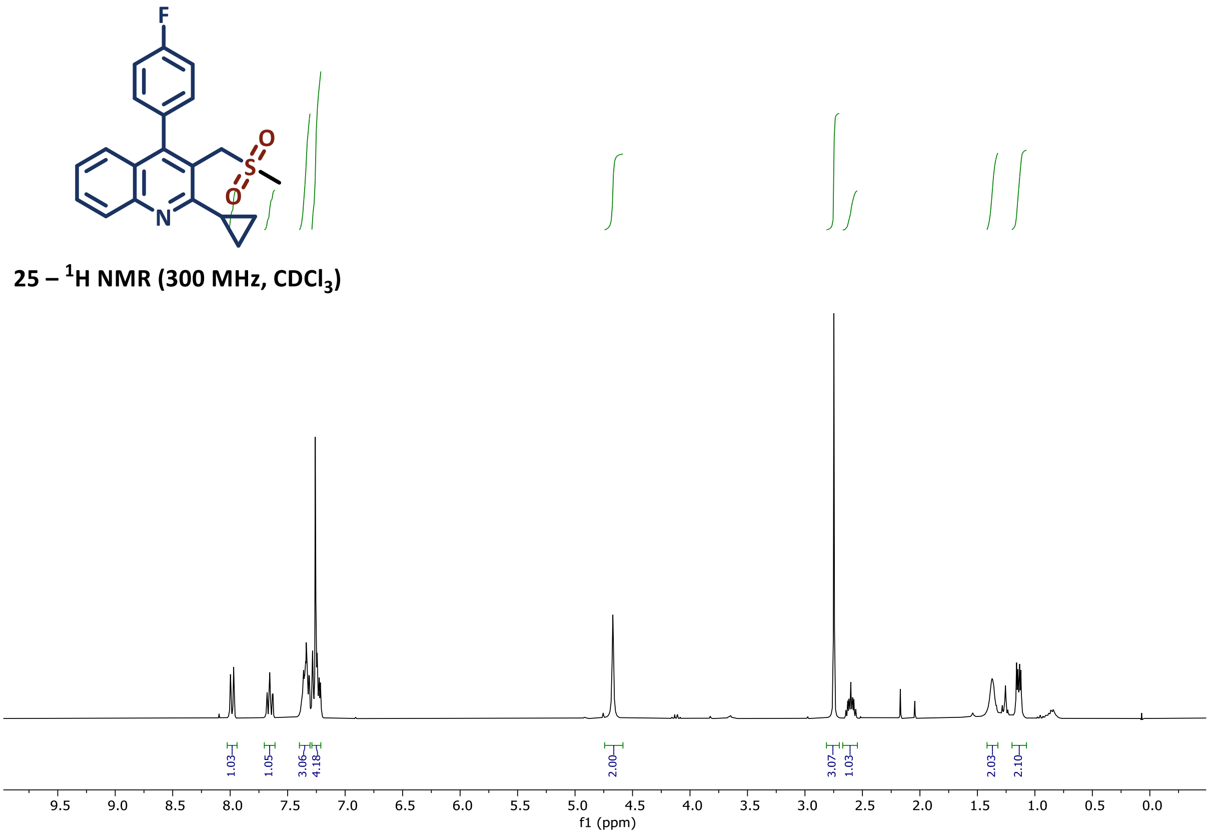

25 – ^{13}C NMR (101 MHz, $CDCl_3$)

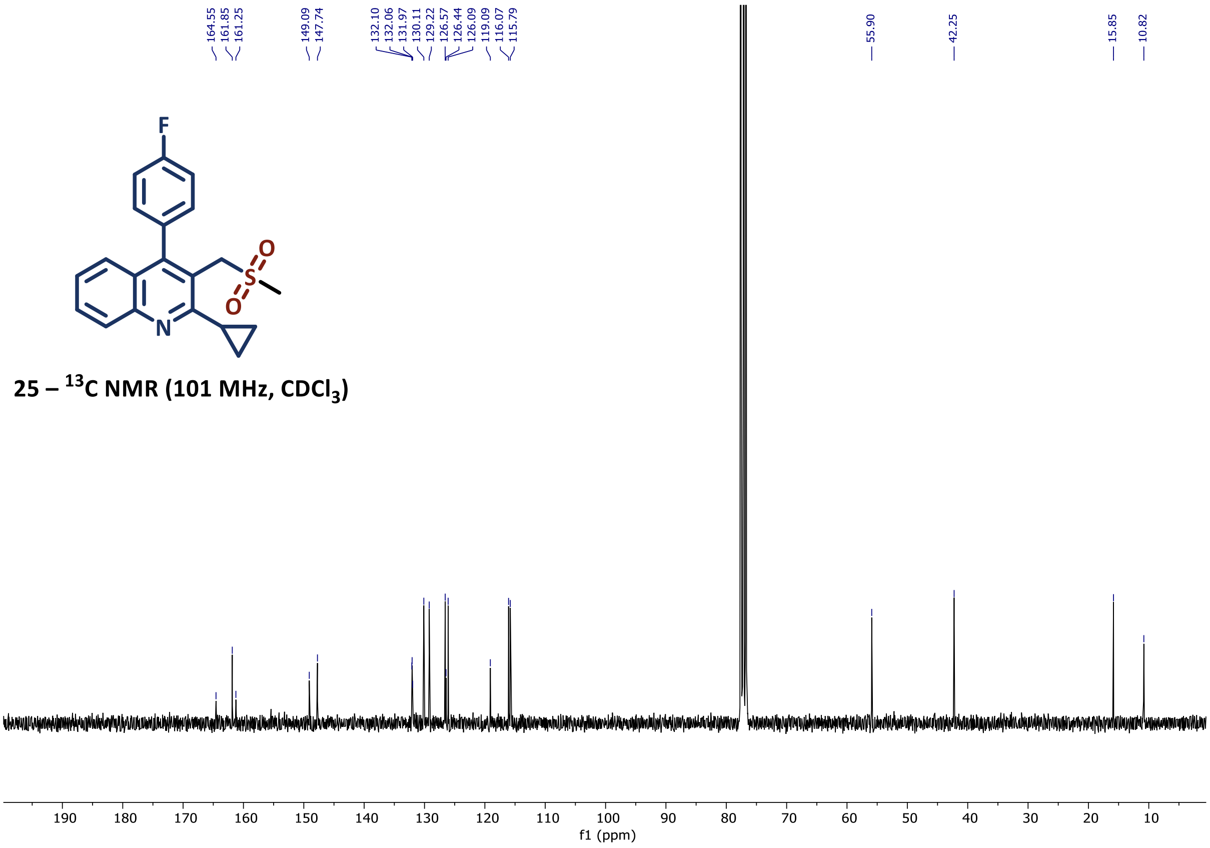

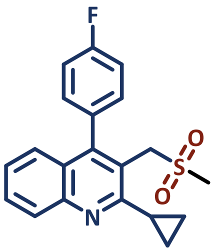

25 – ^{19}F NMR (282 MHz, CDCl$_3$)

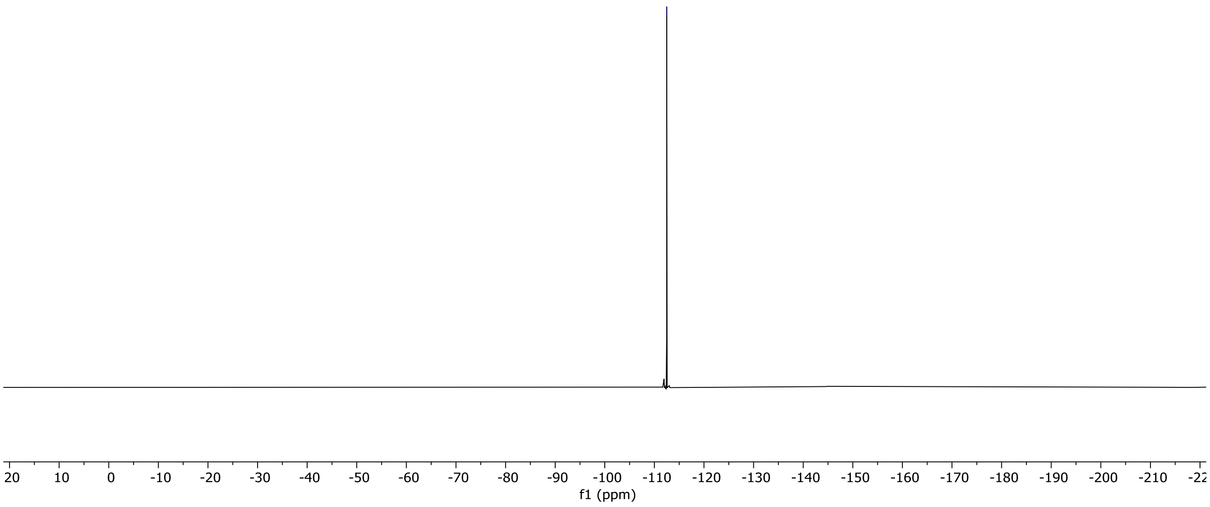

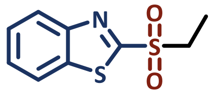

26 – ^{1}H NMR (400 MHz, $CDCl_3$)

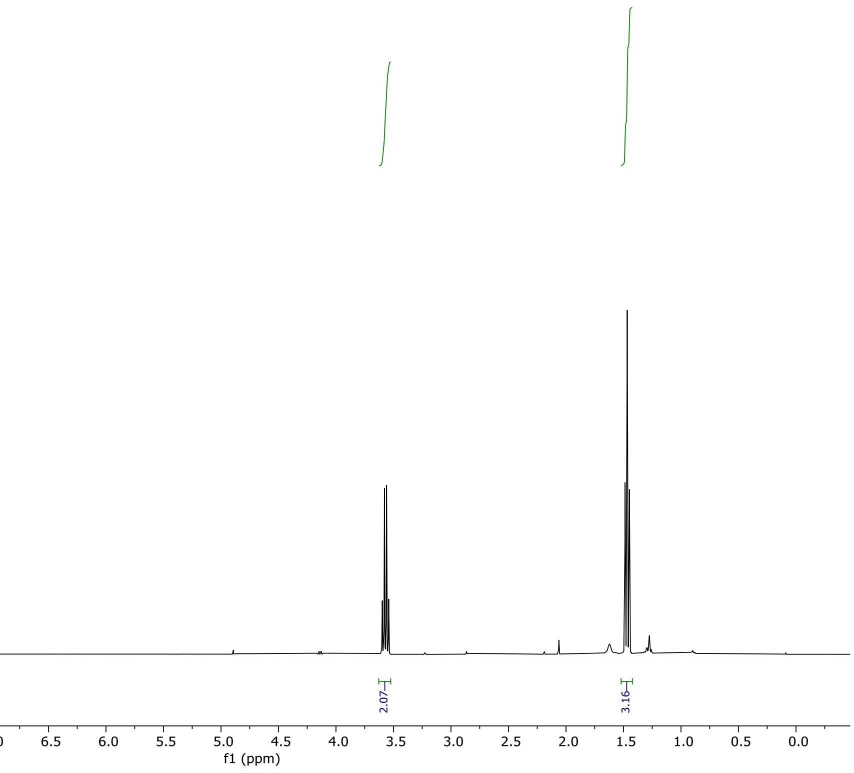

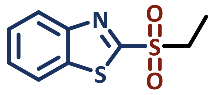

26 – ^{13}C NMR (101 MHz, $CDCl_3$)

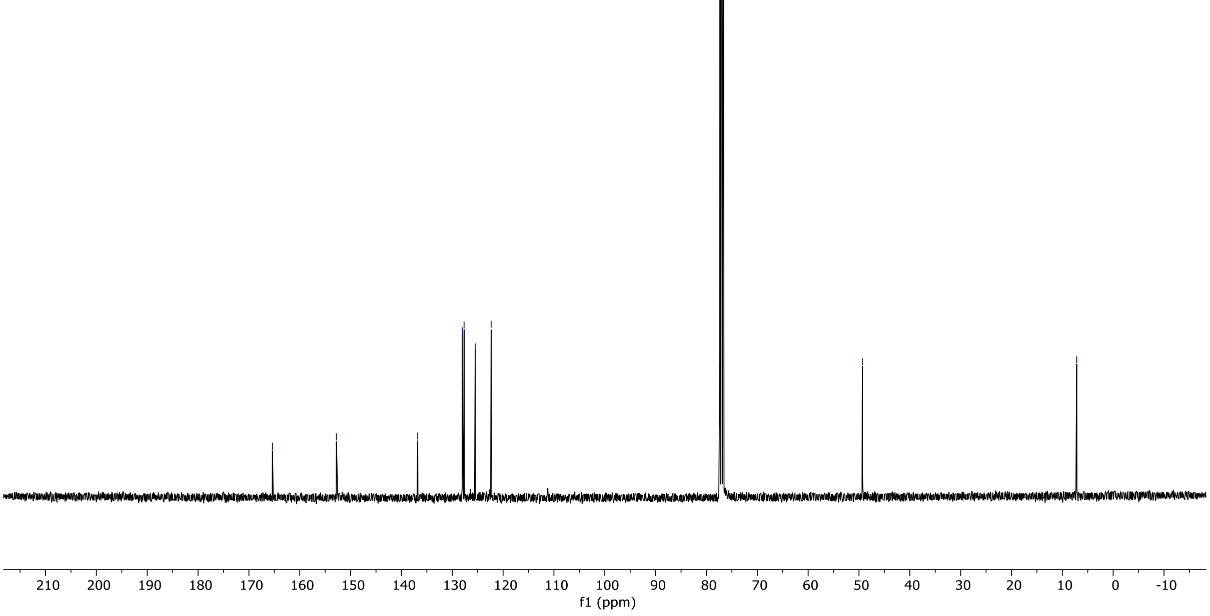

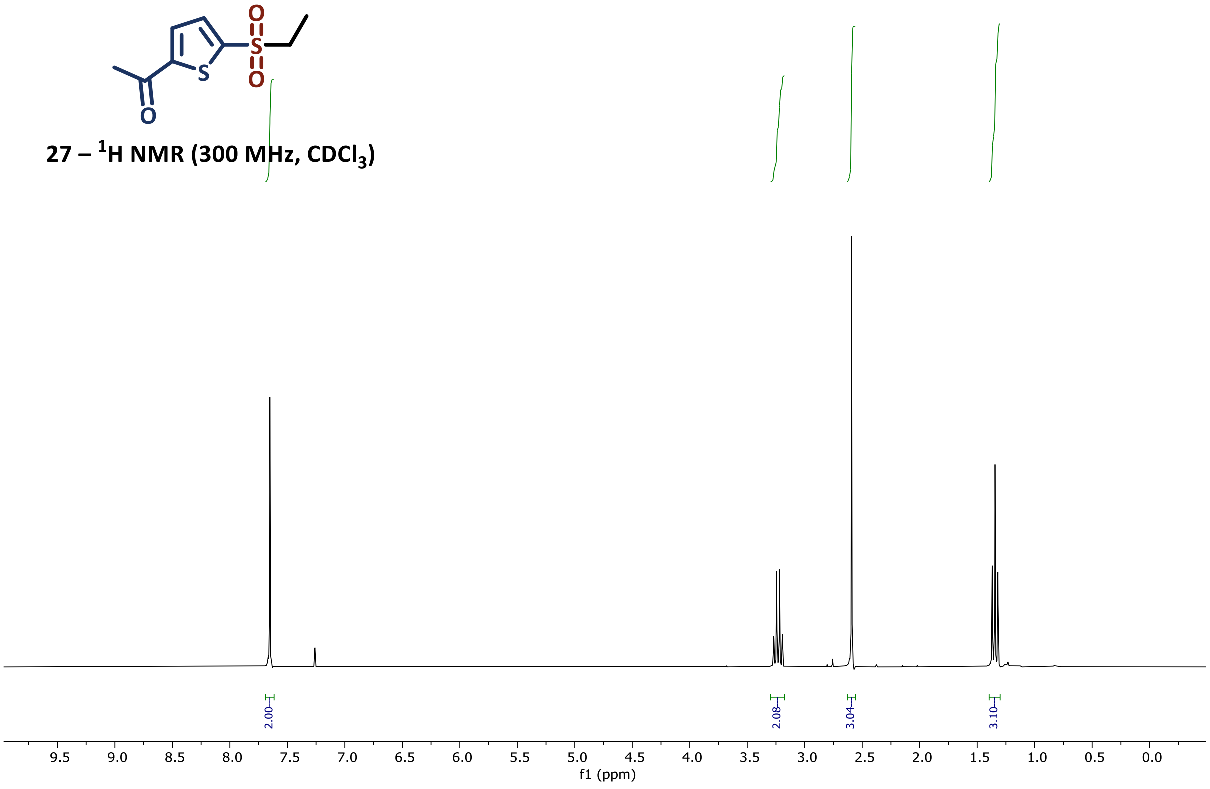

27 – ^{1}H NMR (300 MHz, $CDCl_3$)

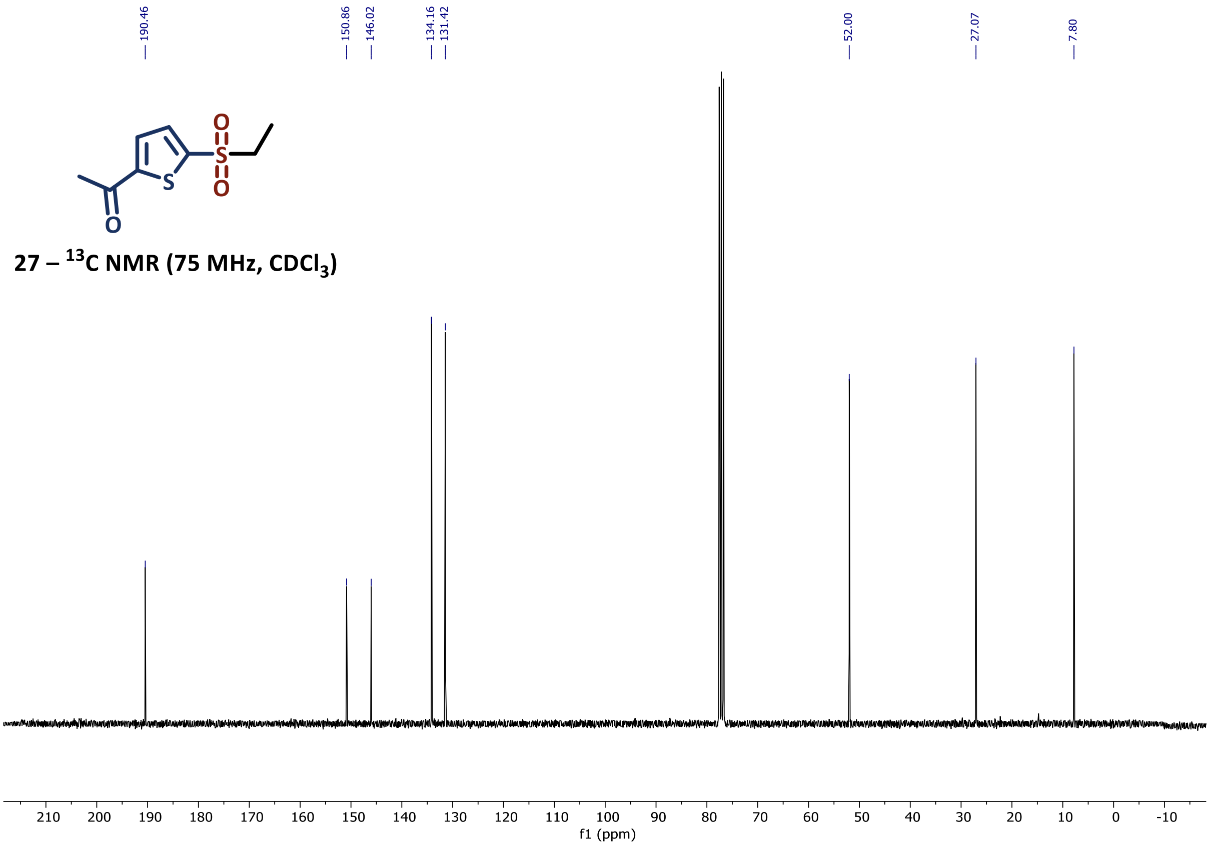

27 – ^{13}C NMR (75 MHz, $CDCl_3$)

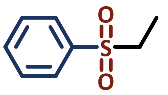

28 – ^{1}H NMR (400 MHz, $CDCl_3$)

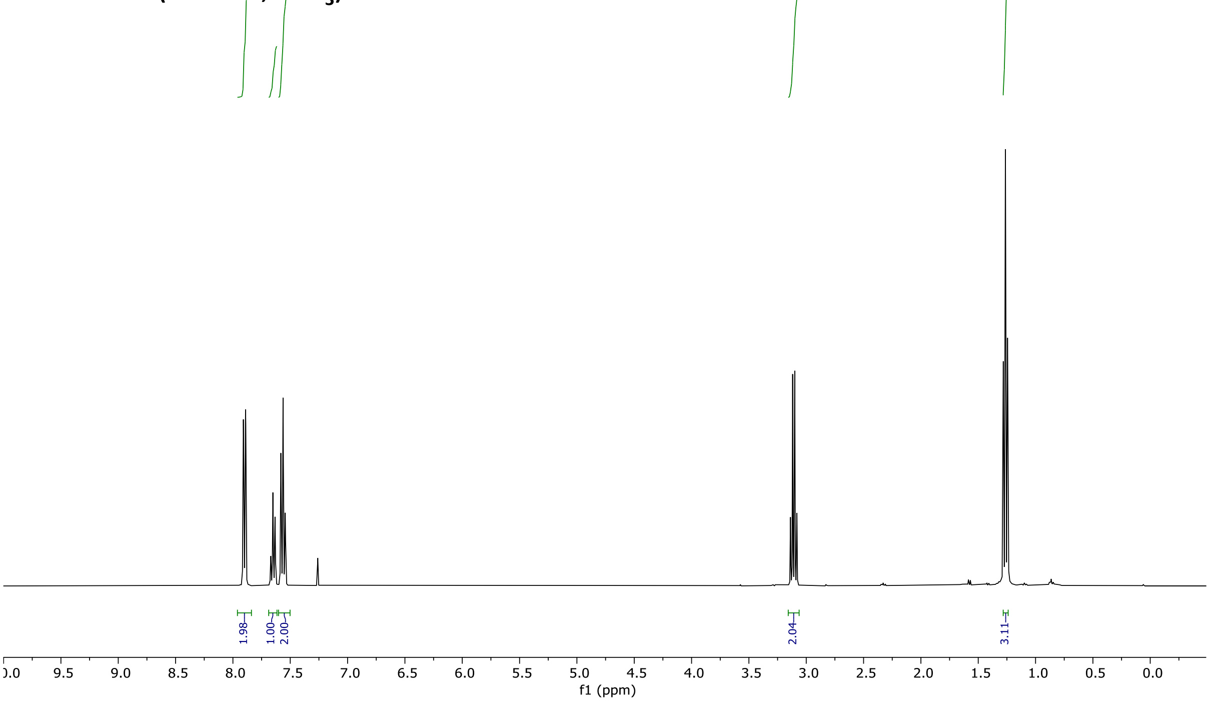

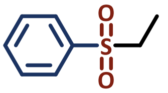

28 – ^{13}C NMR (101 MHz, $CDCl_3$)

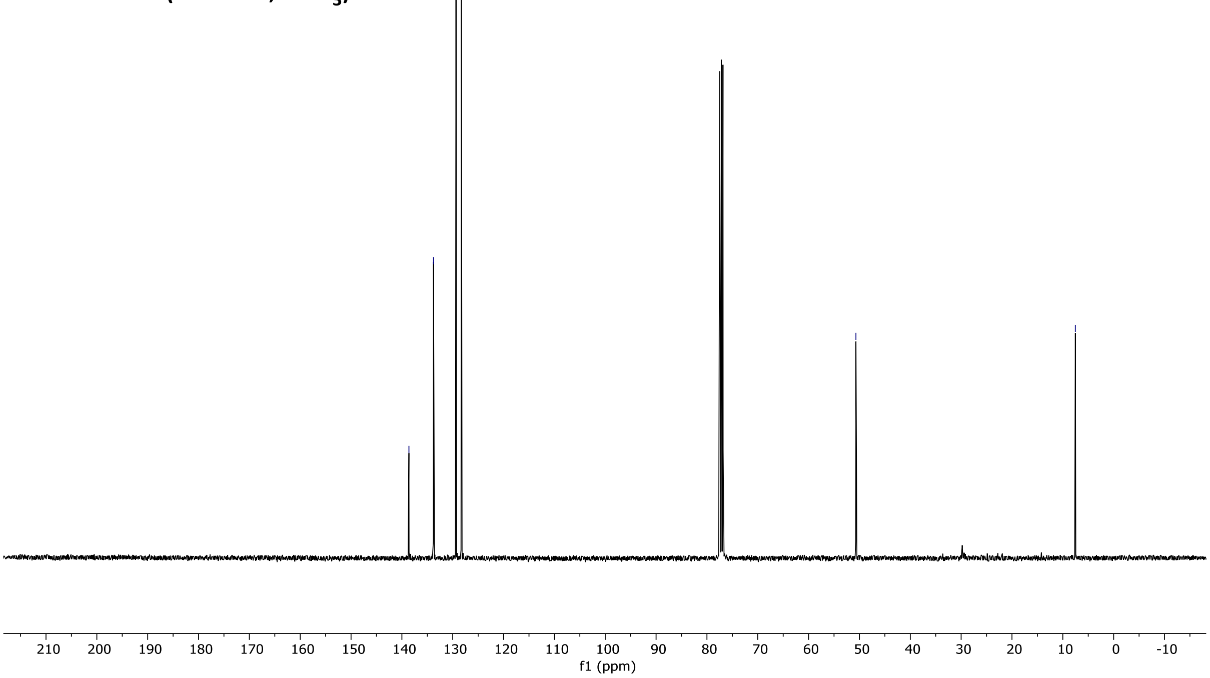

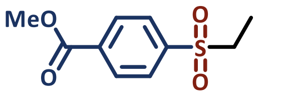

29 – ^{1}H NMR (400 MHz, $CDCl_3$)

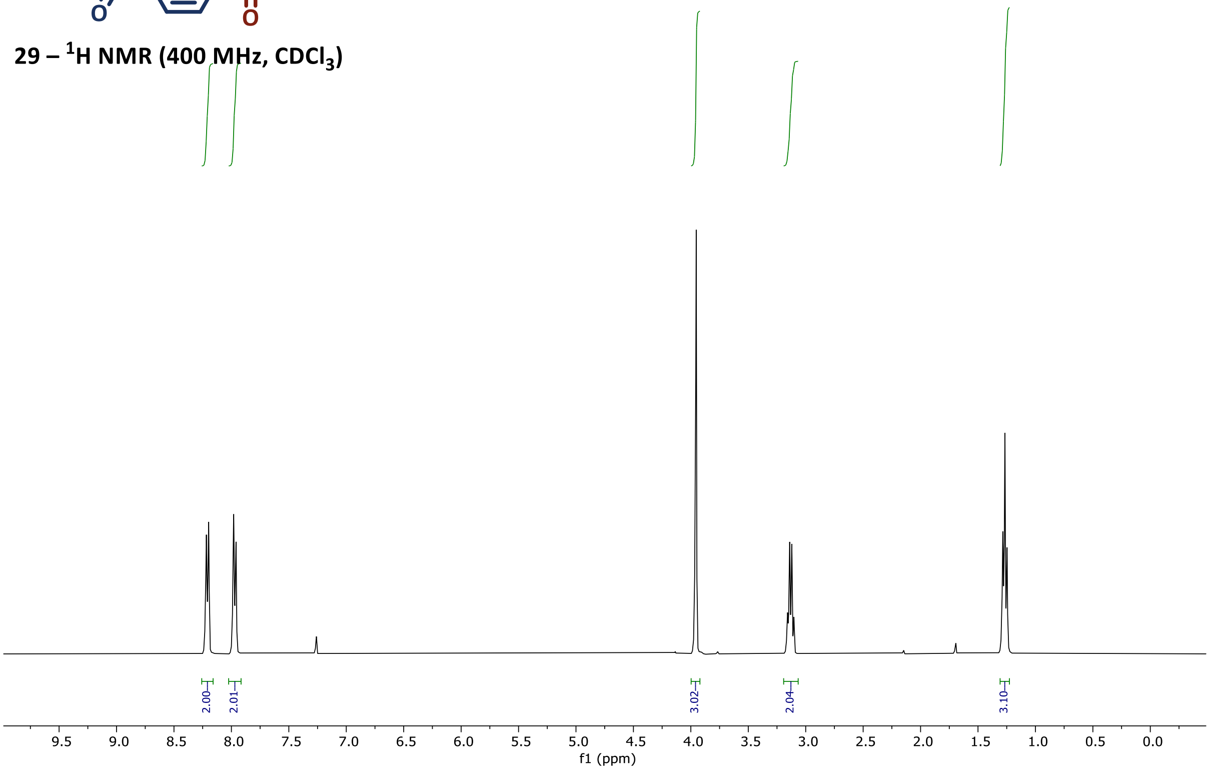

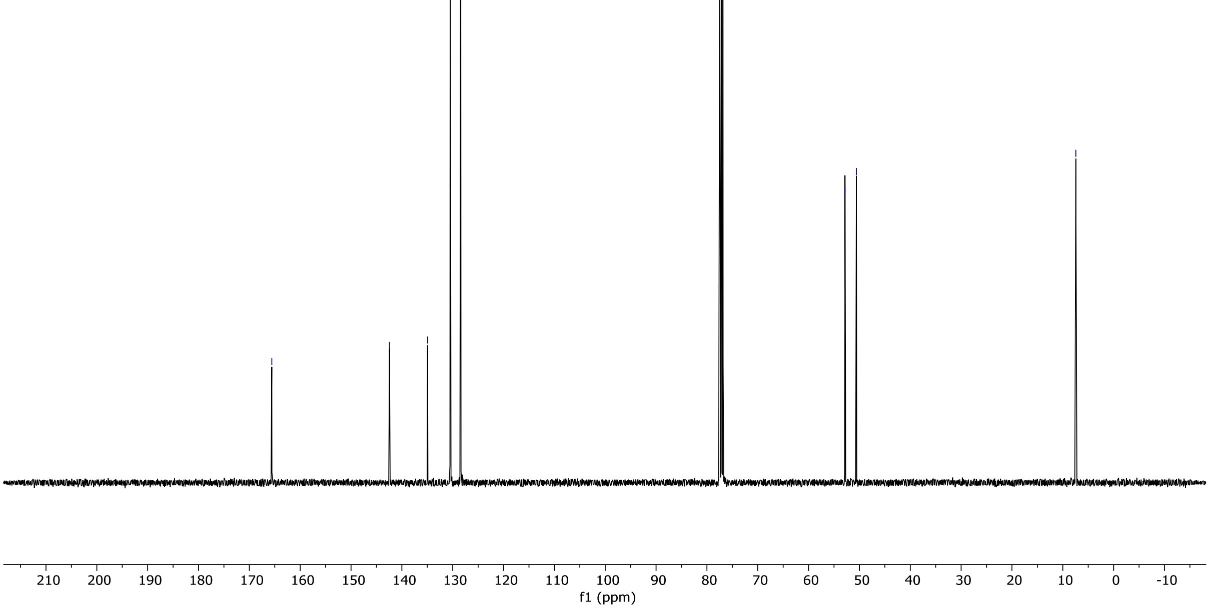

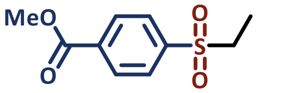

29 – ^{13}C NMR (101 MHz, $CDCl_3$)

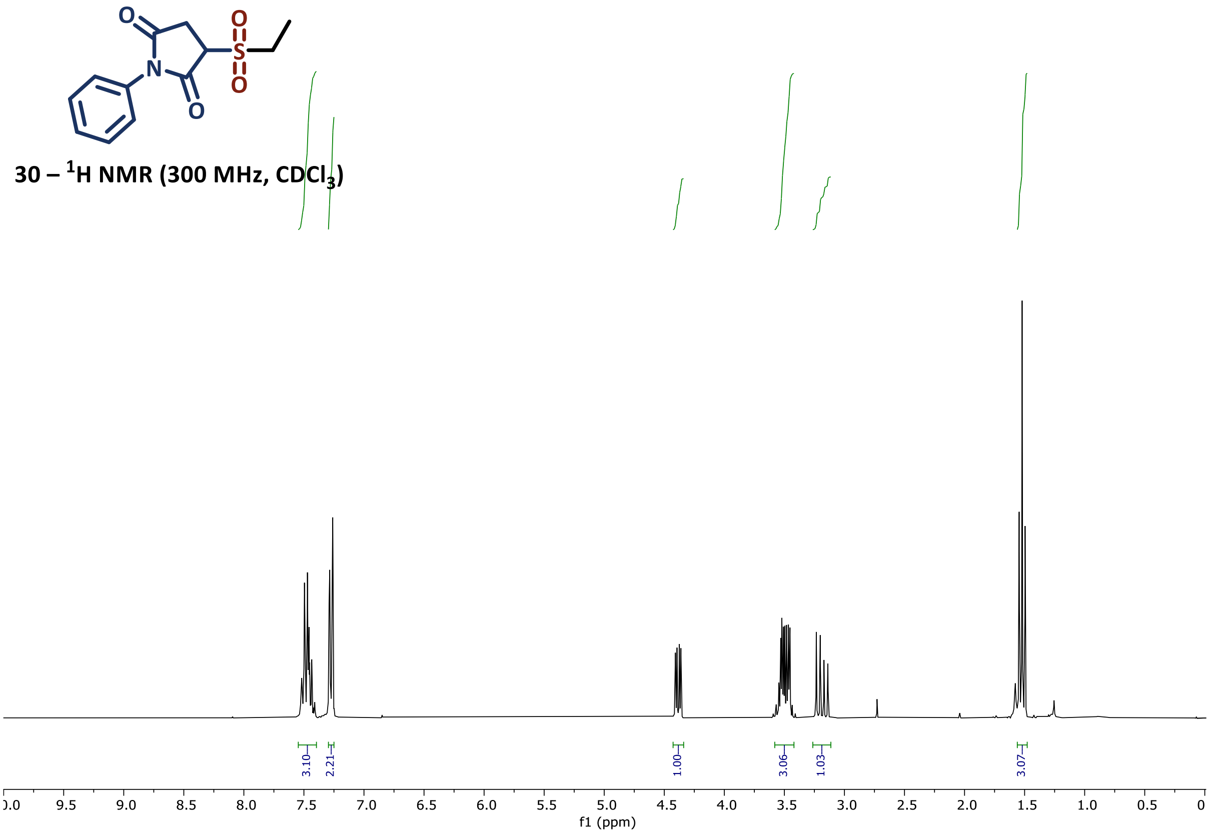
30 – ^{1}H NMR (300 MHz, $CDCl_3$)
3.10
2.21
1.00
3.06
1.03
3.07
f1 (ppm)

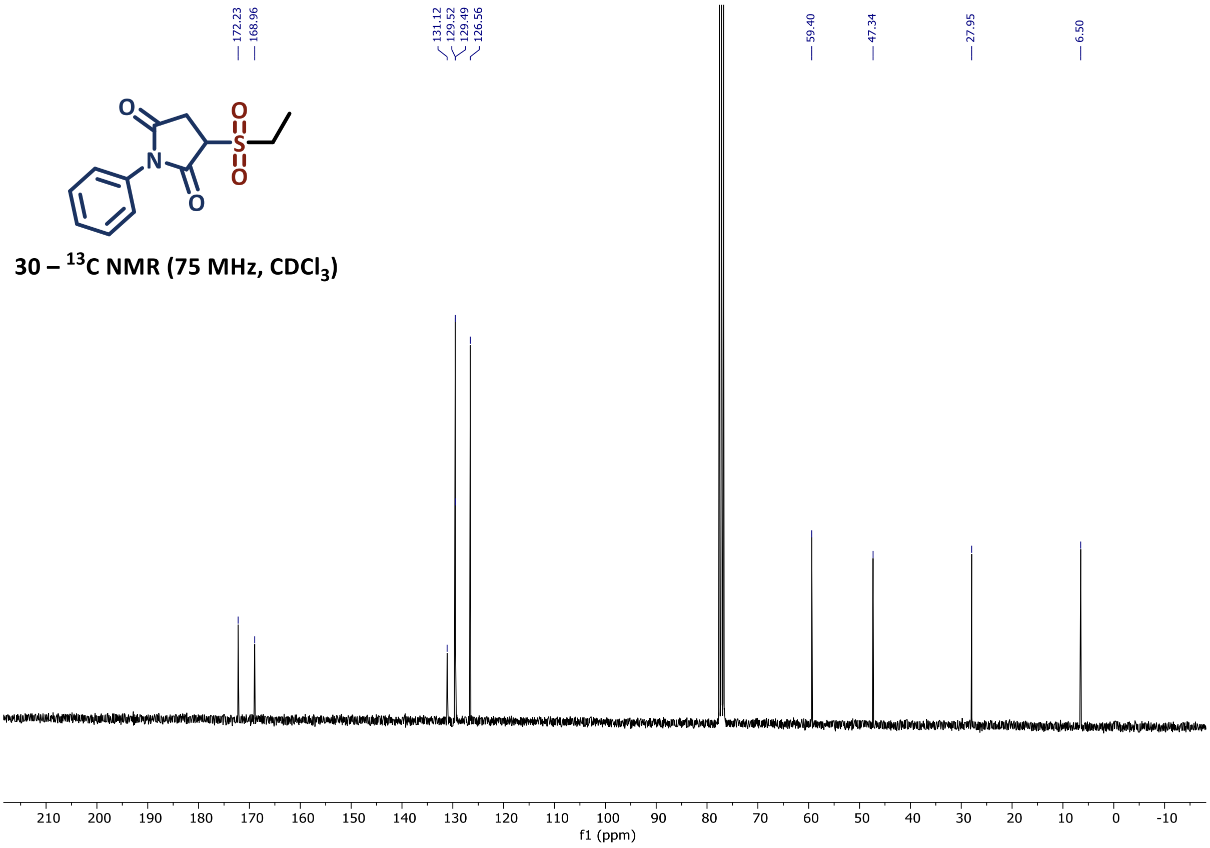
172.23
168.96
131.12
129.52
129.49
126.56
59.40
47.34
27.95
6.50
30 – ^{13}C NMR (75 MHz, $CDCl_3$)
f1 (ppm)

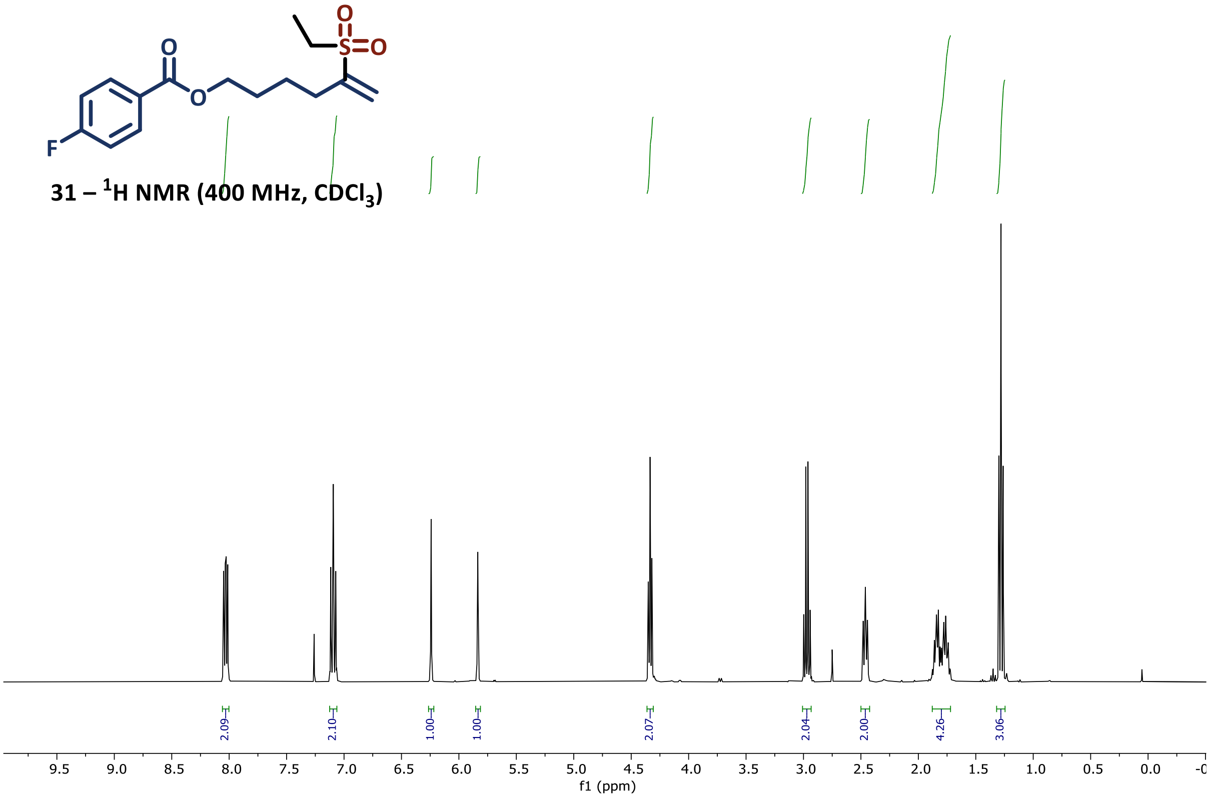

31 – ¹H NMR (400 MHz, CDCl$_3$)

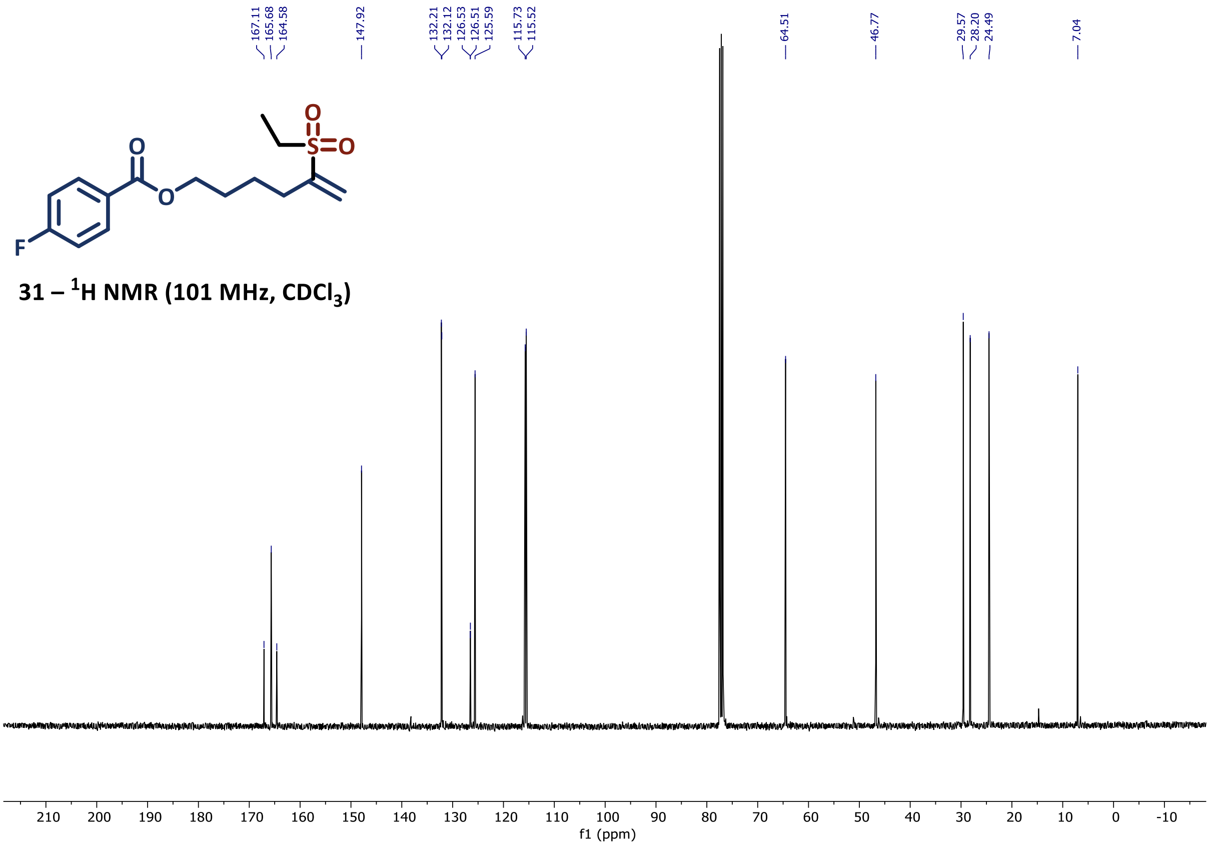

31 – ¹H NMR (101 MHz, CDCl$_3$)

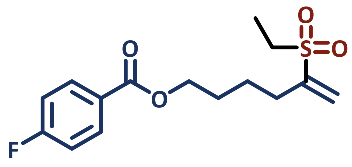

31 – ^{19}F NMR (282 MHz, $CDCl_3$)

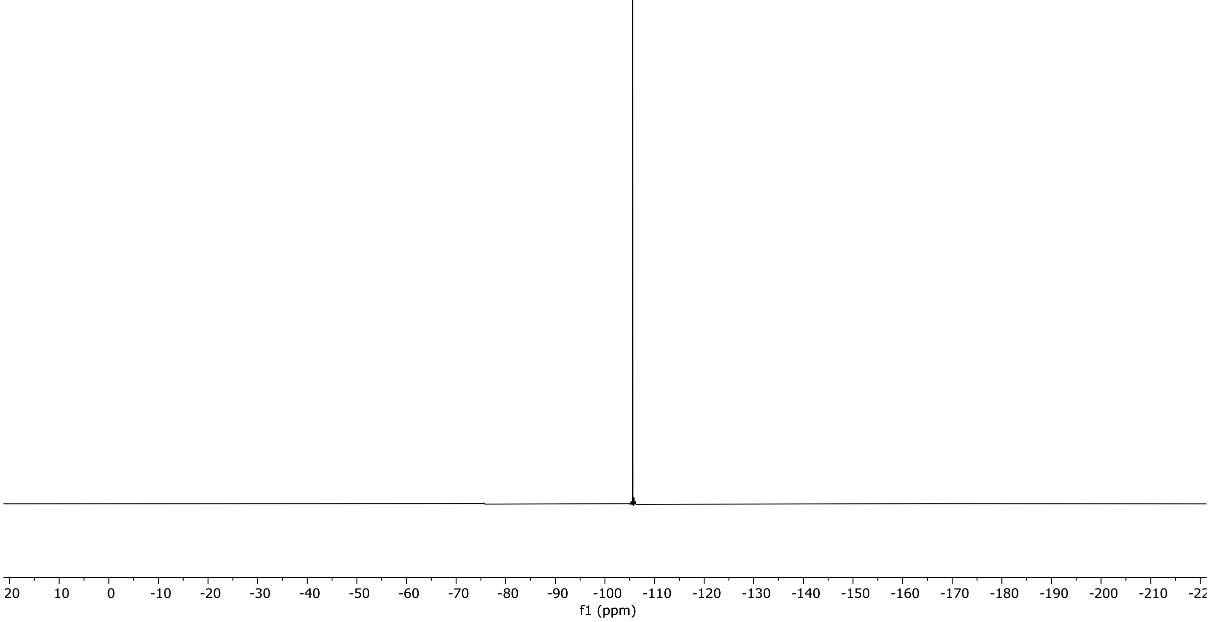

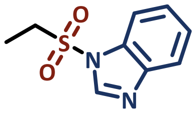

32 – ^{1}H NMR (300 MHz, $CDCl_3$)

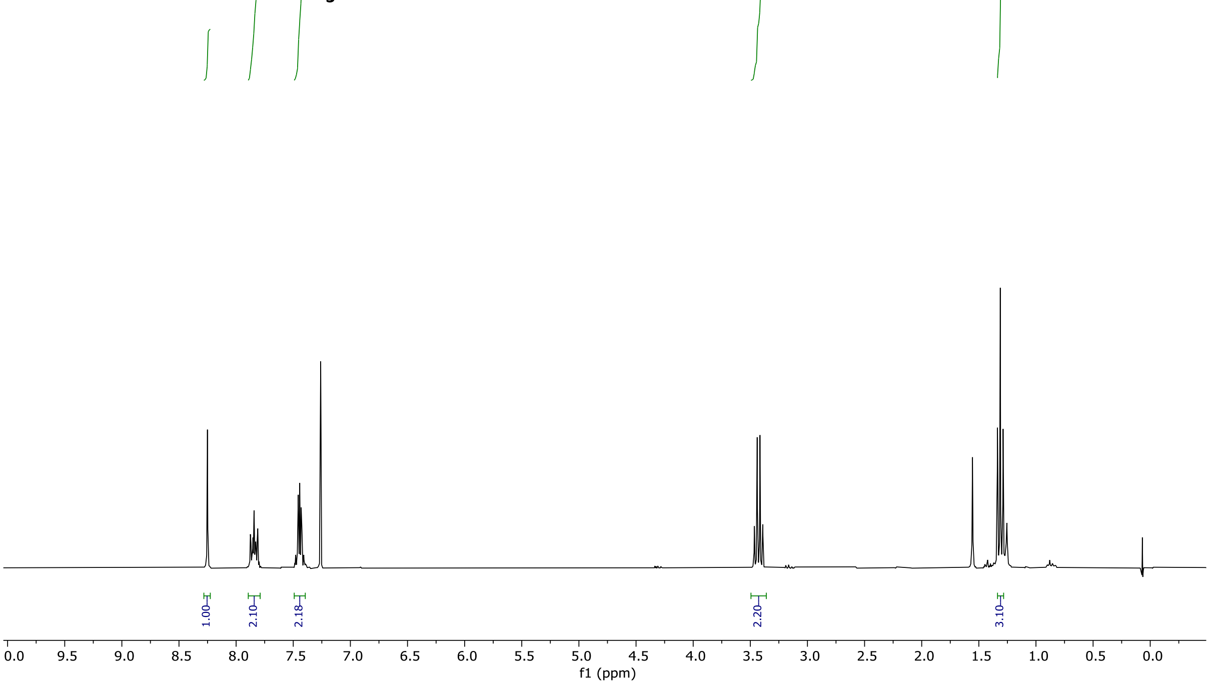

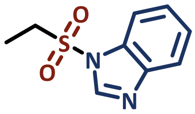

32 – ^{13}C NMR (101 MHz, $CDCl_3$)

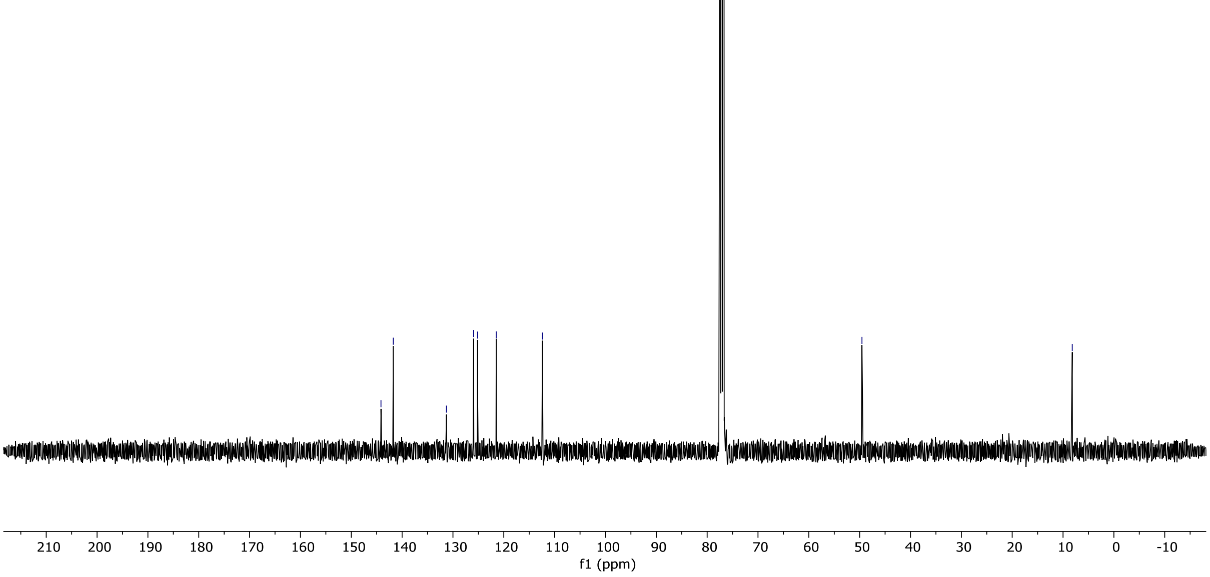

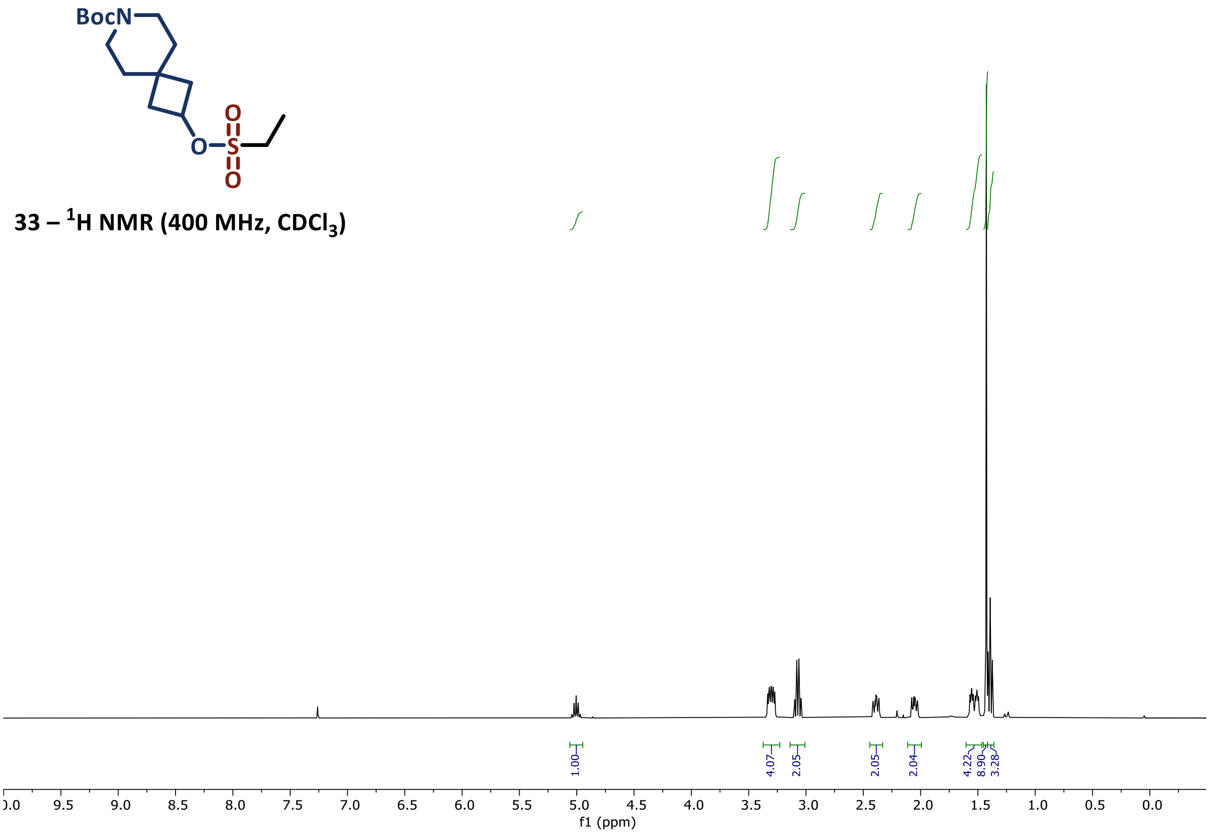

33 – ^{1}H NMR (400 MHz, $CDCl_3$)

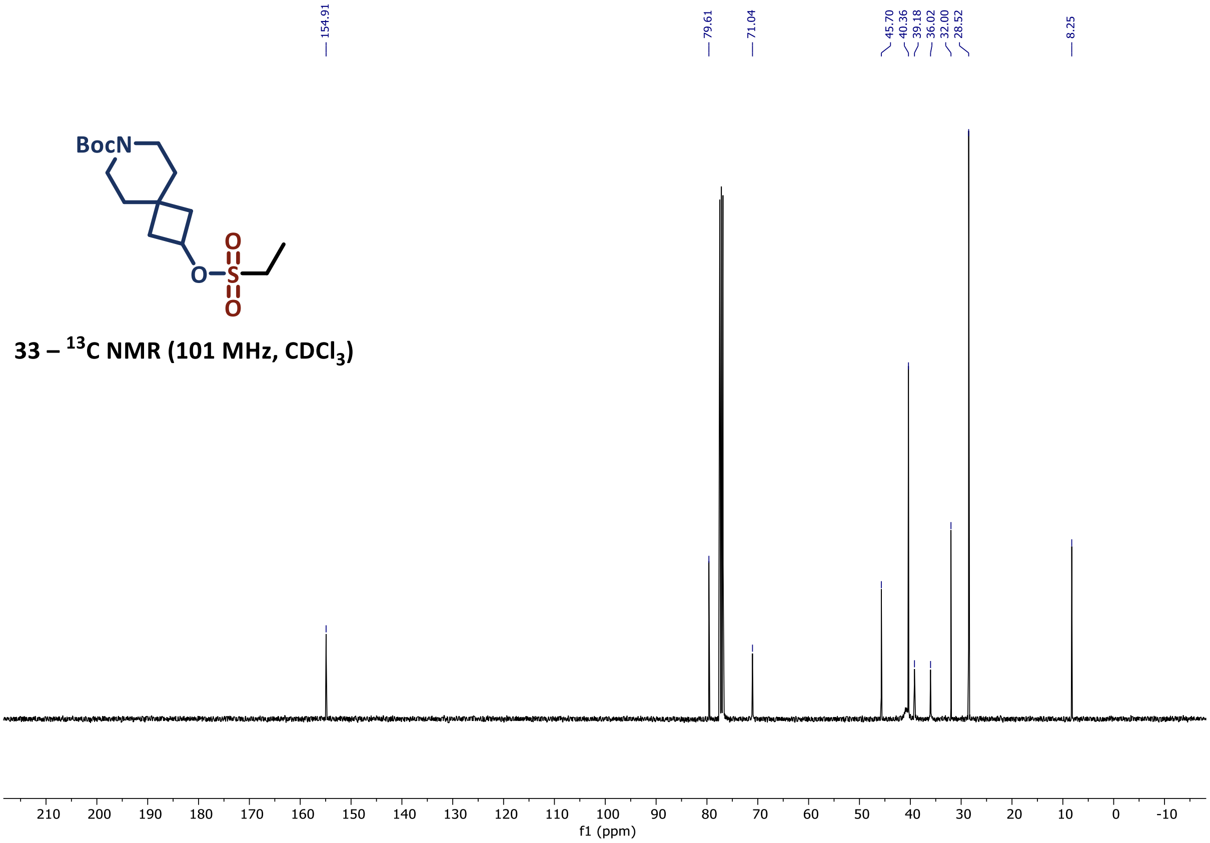

33 – ^{13}C NMR (101 MHz, $CDCl_3$)

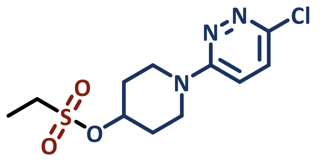

34 – ^{1}H NMR (300 MHz, $CDCl_3$)

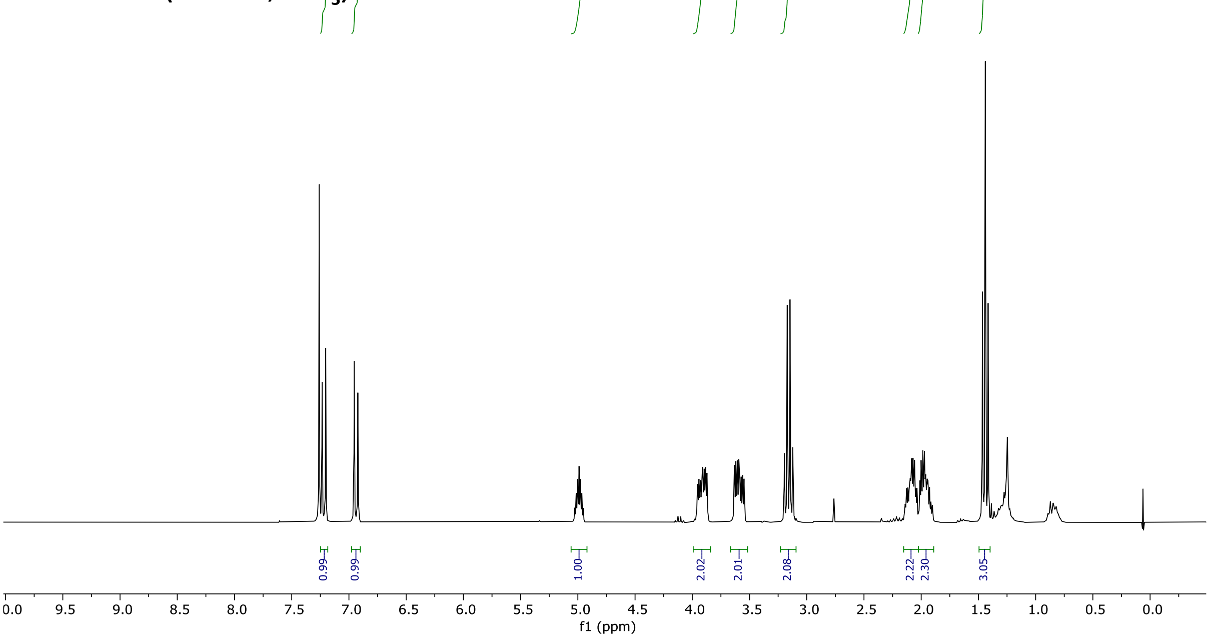

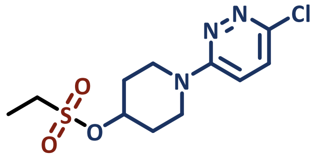

34 – ^{13}C NMR (101 MHz, $CDCl_3$)

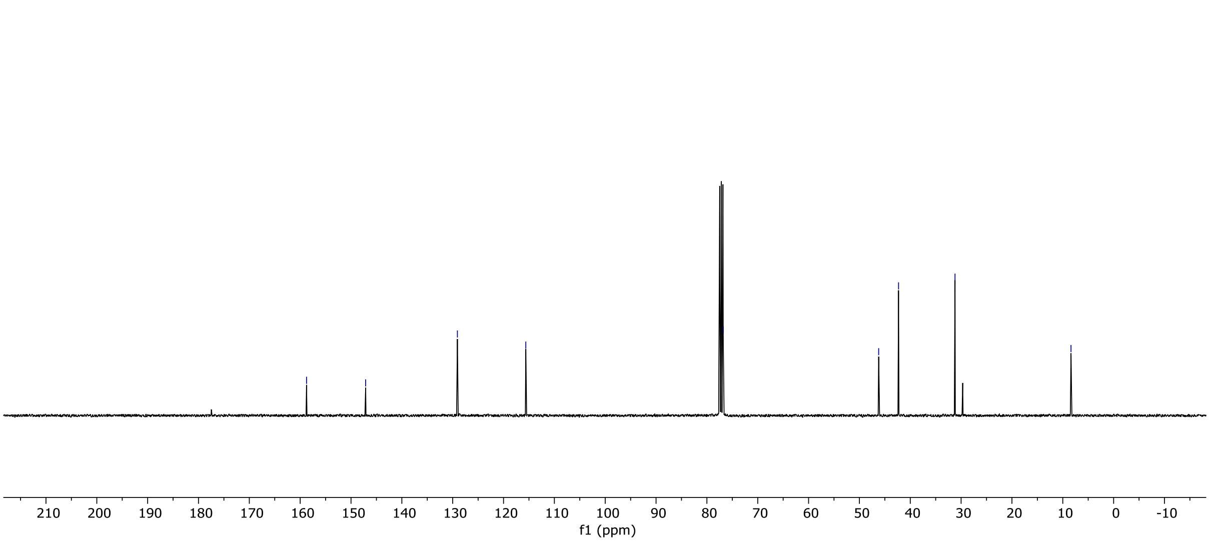

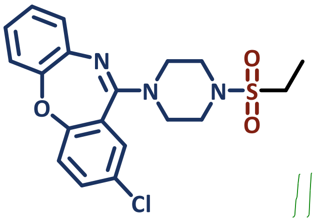

35 – ^{1}H NMR (400 MHz, $CDCl_3$)

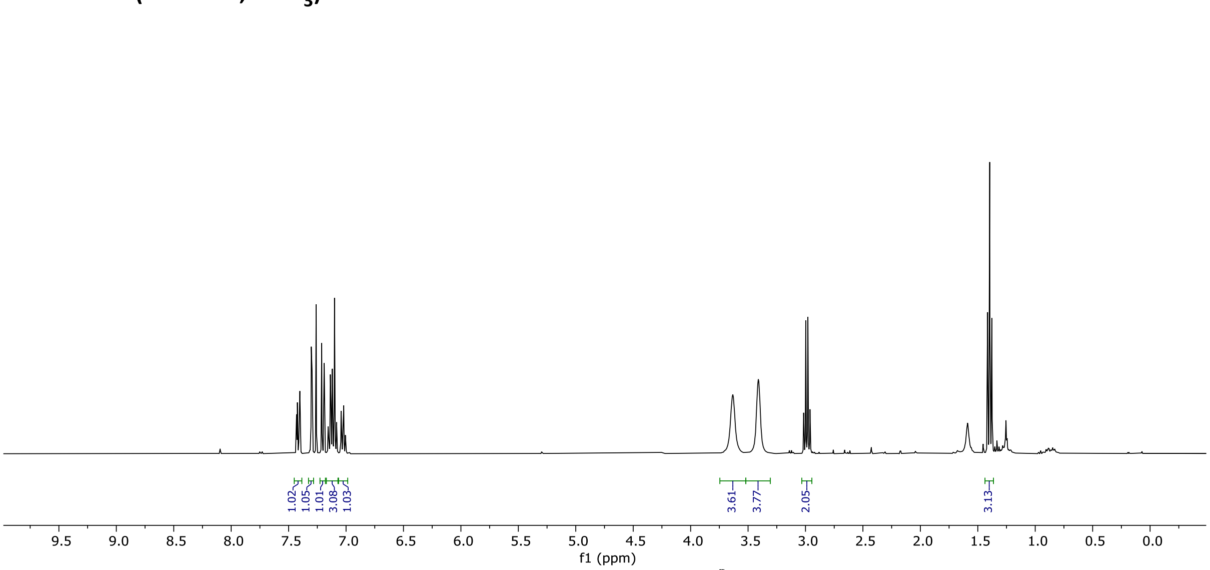

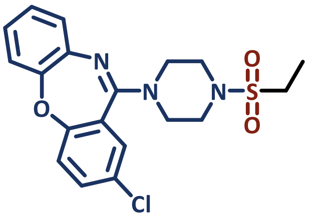

35 – ^{13}C NMR (101 MHz, $CDCl_3$)

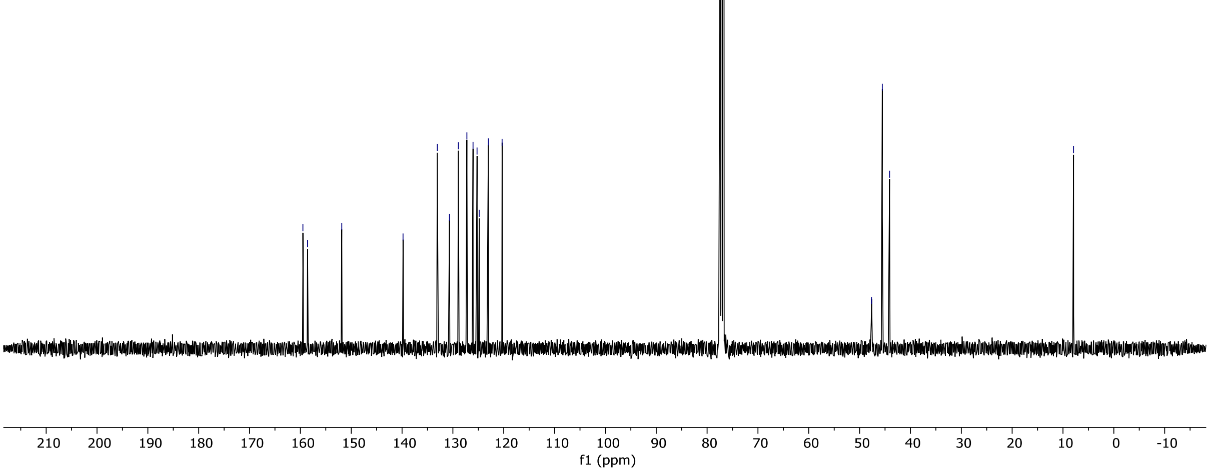

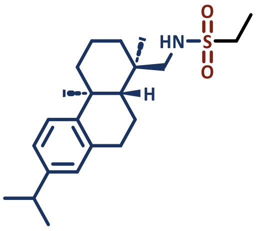

36 – ^{1}H NMR (400 MHz, $CDCl_3$)

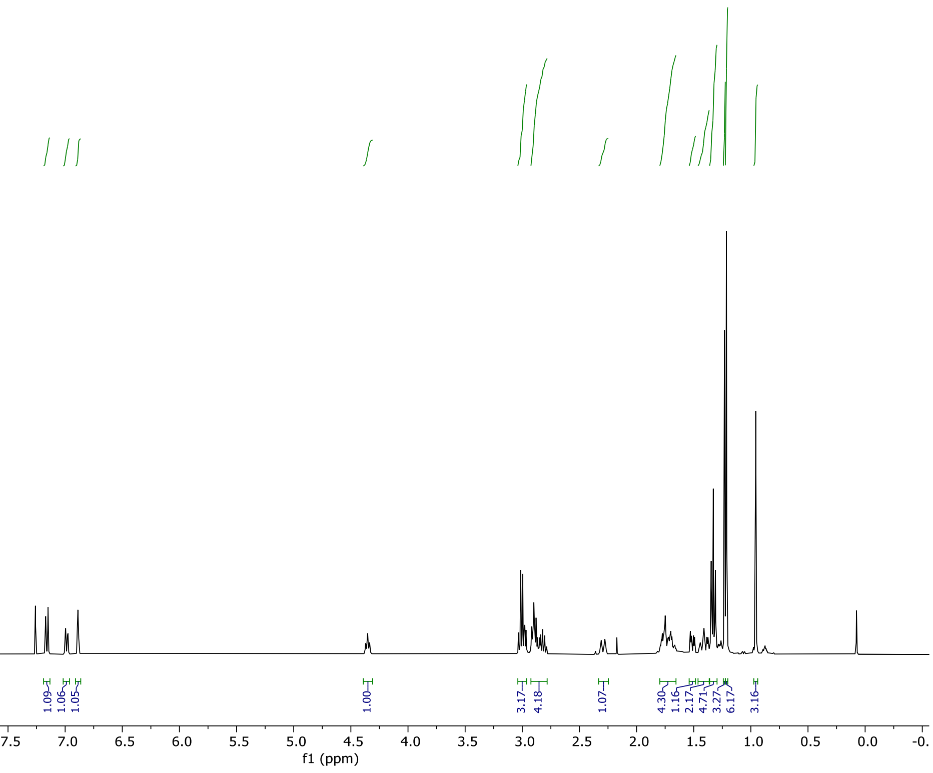

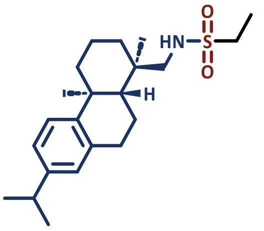

36 – ^{13}C NMR (101 MHz, $CDCl_3$)

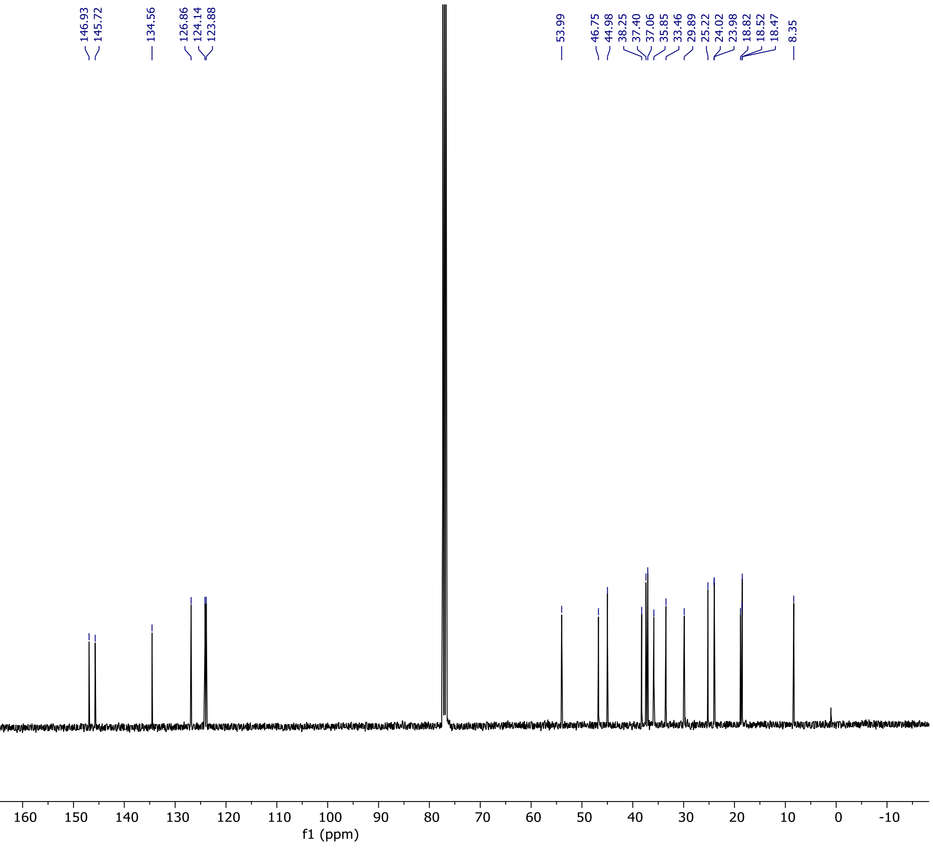

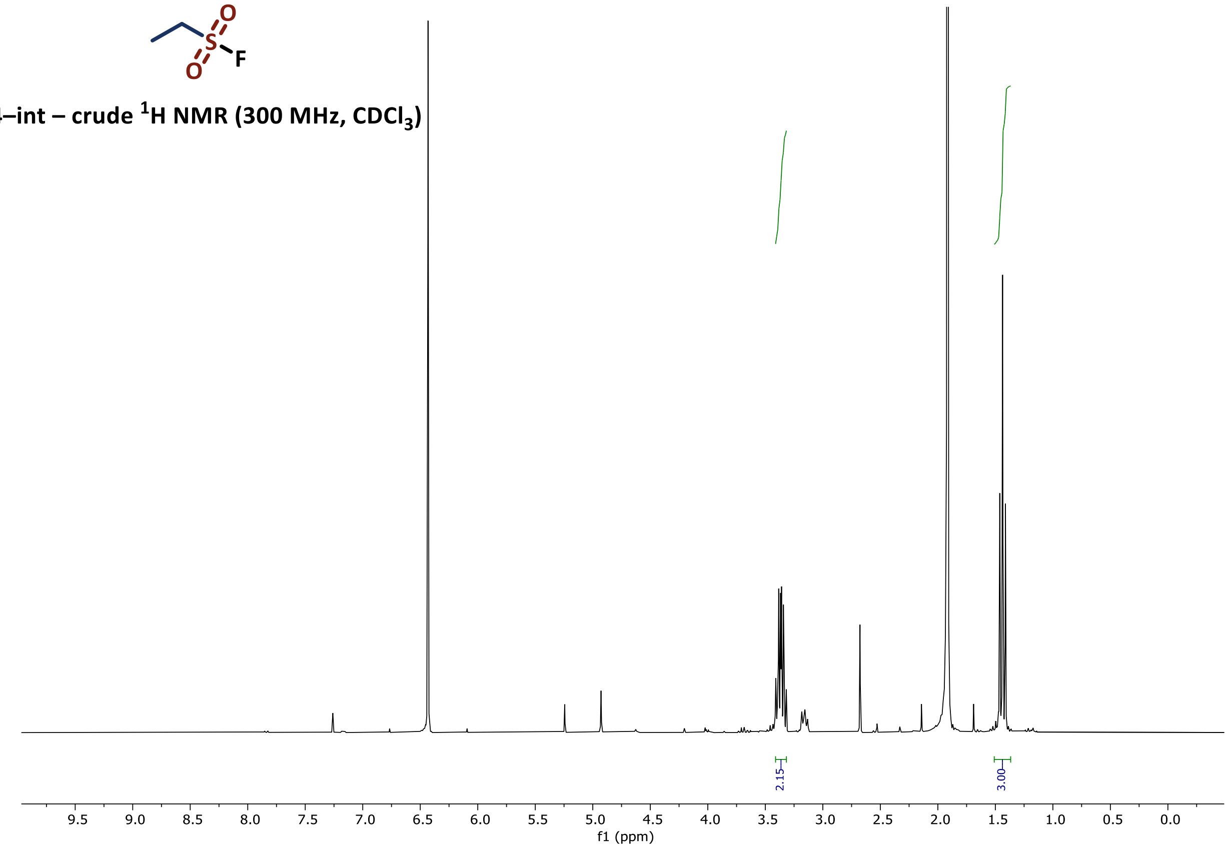

34–int – crude ^{1}H NMR (300 MHz, $CDCl_3$)

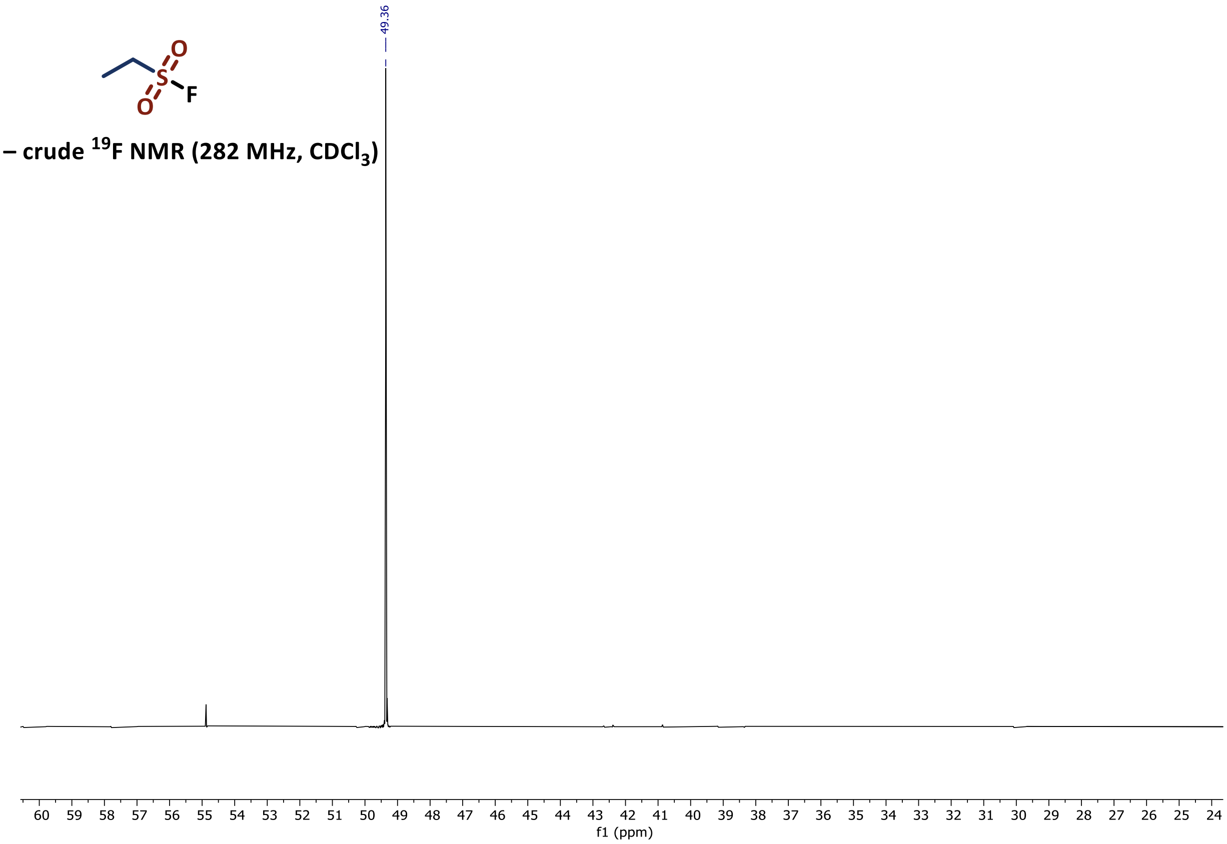

34–int – crude ^{19}F NMR (282 MHz, $CDCl_3$)

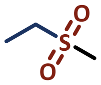

S5 – ^{1}H NMR (400 MHz, $CDCl_3$)

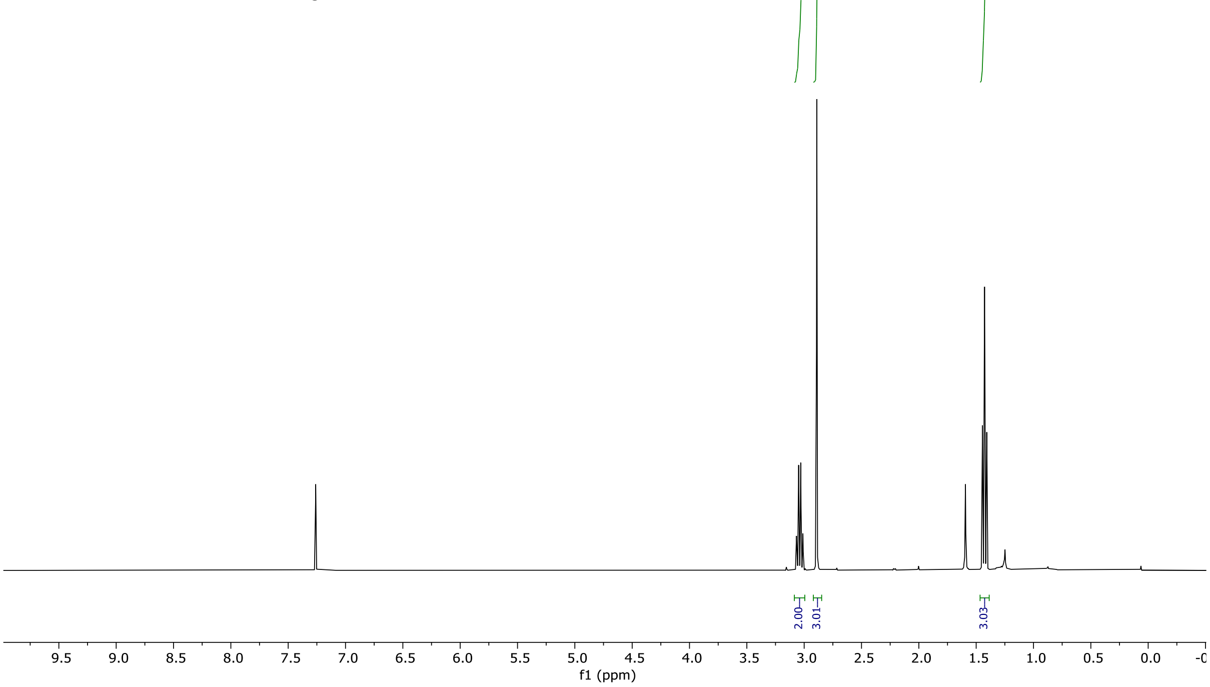

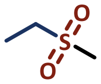

S5 – ^{13}C NMR (100 MHz, $CDCl_3$)

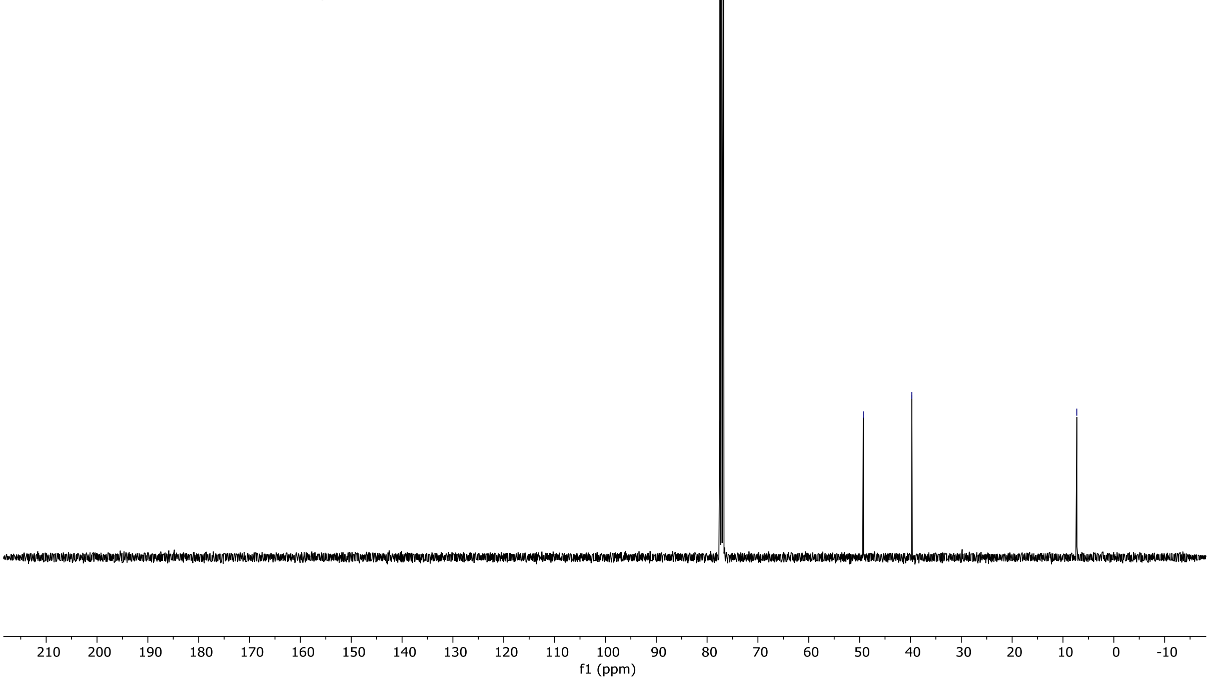

9. References

1. Blum, S. P.; Karakaya, T.; Schollmeyer, D.; Klapars, A.; Waldvogel, S. R. Metal-Free Electrochemical Synthesis of Sulfonamides Directly from (Hetero)arenes, SO2, and Amines. *Angew. Chem. Int. Ed.* **2021**, *60* (10), 5056-5062.
2. Wan, T., et al. Decatungstate-Mediated C(sp3)–H Heteroarylation via Radical-Polar Crossover in Batch and Flow. *Angew. Chem. Int. Ed.* **2021**, *60* (33), 17893-17897.
3. Sarver, P. J.; Bissonnette, N. B.; MacMillan, D. W. C. Decatungstate-Catalyzed C(sp3)–H Sulfinylation: Rapid Access to Diverse Organosulfur Functionality. *J. Am. Chem. Soc.* **2021**, *143* (26), 9737-9743.
4. Wan, T., et al. Accelerated and Scalable C(sp3)–H Amination via Decatungstate Photocatalysis Using a Flow Photoreactor Equipped with High-Intensity LEDs. *ACS Cent. Sci.* **2022**, *8* (1), 51-56.
5. Raymenants, F.; Masson, T. M.; Sanjosé-Orduna, J.; Noël, T. Efficient C(sp3)−H Carbonylation of Light and Heavy Hydrocarbons with Carbon Monoxide via Hydrogen Atom Transfer Photocatalysis in Flow. *Angew. Chem. Int. Ed.* **2023**, *62* (36), e202308563.
6. Laudadio, G., et al. C(sp3)–H functionalizations of light hydrocarbons using decatungstate photocatalysis in flow. *Science* **2020**, *369* (6499), 92-96.
7. Gu, D.; Harpp, D. N. The reaction of mercaptans with dimethyldioxirane. A facile synthesis of alkanesulfinic acids. *Tetrahedron Lett.* **1993**, *34* (1), 67-70.
8. Meyer, A. U.; Straková, K.; Slanina, T.; König, B. Eosin Y (EY) Photoredox-Catalyzed Sulfonylation of Alkenes: Scope and Mechanism. *Chemistry – A European Journal* **2016**, *22* (25), 8694-8699.
9. Spreider, P. A.; Breit, B. Palladium-Catalyzed Stereoselective Cyclization of in Situ Formed Allenyl Hemiacetals: Synthesis of Rosuvastatin and Pitavastatin. *Org. Lett.* **2018**, *20* (11), 3286-3290.
10. Yi, D.; Zhu, F.; Walczak, M. A. Glycosyl Cross-Coupling with Diaryliodonium Salts: Access to Aryl C-Glycosides of Biomedical Relevance. *Org. Lett.* **2018**, *20* (7), 1936-1940.
11. Deeming, A. S.; Russell, C. J.; Hennessy, A. J.; Willis, M. C. DABSO-Based, Three-Component, One-Pot Sulfone Synthesis. *Org. Lett.* **2014**, *16* (1), 150-153.
12. Fu, Y., et al. CuI catalyzed sulfonylation of organozinc reagents with sulfonyl halides. *Org. Biomol. Chem.* **2014**, *12* (25), 4295-4299.
13. Schevenels, F. T.; Shen, M.; Snyder, S. A. Isolable and Readily Handled Halophosphonium Pre-reagents for Hydro- and Deuteriohalogenation. *J. Am. Chem. Soc.* **2017**, *139* (18), 6329-6337.
14. Motiwala, H. F.; Kuo, Y.-H.; Stinger, B. L.; Palfey, B. A.; Martin, B. R. Tunable Heteroaromatic Sulfones Enhance in-Cell Cysteine Profiling. *J. Am. Chem. Soc.* **2020**, *142* (4), 1801-1810.
15. Yang, C., et al. Tetra-(tetraalkylammonium)octamolybdate catalysts for selective oxidation of sulfides to sulfoxides with hydrogen peroxide. *Green Chem.* **2009**, *11* (9), 1401-1405.
16. Wang, Y.; Zhang, F.; Wang, Y.; Pan, Y. Electrochemistry Enabled Nickel-Catalyzed Selective C−S Bond Coupling Reaction. *Eur. J. Org. Chem.* **2022**, *2022* (5), e202101462.
17. Deng, S.-H.; Zhao, S.-Y.; Huang, Y.-Y.; Chang, M.-R.; Dong, Z.-B. Glyoxylic Acid Monohydrate-Promoted Formation of the C–SO2 Bond Starting from Maleimides/Quinones and Sodium Sulfinates. *J. Org. Chem.* **2023**, *88* (22), 15925-15936.
